# Citation needed? Wikipedia bibliometrics during the first wave of the COVID-19 pandemic

**DOI:** 10.1093/gigascience/giab095

**Published:** 2022-01-12

**Authors:** Omer Benjakob, Rona Aviram, Jonathan Aryeh Sobel

**Affiliations:** Center for Research and Interdisciplinarity (CRI), Université de Paris, INSERM U1284, 8 bis Rue Charles V, 75004 Paris, France; The Cohn Institute for the History and Philosophy of Science and Ideas, Humanities Faculty, Tel Aviv University, Ramat Aviv, Tel Aviv 6997801, Israel; Center for Research and Interdisciplinarity (CRI), Université de Paris, INSERM U1284, 8 bis Rue Charles V, 75004 Paris, France; Department of Biomolecular Sciences, Weizmann Institute of Science, Rehovot 76100, Israel; Department of Biomolecular Sciences, Weizmann Institute of Science, Rehovot 76100, Israel; Department of Biomedical Engineering, Julius Silver Building, Technion-IIT, Technion City, Haifa 32000, Israel

**Keywords:** COVID-19, Wikipedia, infodemic, sources, bibliometrics, citizen science, open science

## Abstract

**Background:**

With the COVID-19 pandemic’s outbreak, millions flocked to Wikipedia for updated information. Amid growing concerns regarding an “infodemic,” ensuring the quality of information is a crucial vector of public health. Investigating whether and how Wikipedia remained up to date and in line with science is key to formulating strategies to counter misinformation. Using citation analyses, we asked which sources informed Wikipedia’s COVID-19–related articles before and during the pandemic’s first wave (January–May 2020).

**Results:**

We found that coronavirus-related articles referenced trusted media outlets and high-quality academic sources. Regarding academic sources, Wikipedia was found to be highly selective in terms of what science was cited. Moreover, despite a surge in COVID-19 preprints, Wikipedia had a clear preference for open-access studies published in respected journals and made little use of preprints. Building a timeline of English-language COVID-19 articles from 2001–2020 revealed a nuanced trade-off between quality and timeliness. It further showed how pre-existing articles on key topics related to the virus created a framework for integrating new knowledge. Supported by a rigid sourcing policy, this “scientific infrastructure” facilitated contextualization and regulated the influx of new information. Last, we constructed a network of DOI-Wikipedia articles, which showed the landscape of pandemic-related knowledge on Wikipedia and how academic citations create a web of shared knowledge supporting topics like COVID-19 drug development.

**Conclusions:**

Understanding how scientific research interacts with the digital knowledge-sphere during the pandemic provides insight into how Wikipedia can facilitate access to science. It also reveals how, aided by what we term its “citizen encyclopedists,” it successfully fended off COVID-19 disinformation and how this unique model may be deployed in other contexts.

## Introduction

Wikipedia has >130,000 different articles relating to health and medicine [1]. The website as a whole, and specifically its medical and health articles, such as those about diseases or drugs, are a prominent source of information for the general public [2]. Studies of readership and editorship of health-related articles reveal that medical professionals are active consumers of Wikipedia and make up roughly half of those involved in editing these articles in English [3,4]. Research conducted into the quality and scope of medical content deemed Wikipedia “a key tool for global public health promotion” [4,5]. Others have found that in terms of content errors Wikipedia is on par with academic and professional sources even in fields like medicine [6]. Meanwhile, a metastudy of Wikipedia’s medical content (specifically those articles overseen by the WikiProject Medicine, a volunteer-run group of editors that focuses on ensuring the quality of health-related articles) found it to be a prominent health information resource for experts and non-experts alike [7]. With the World Health Organization (WHO) labeling the COVID-19 pandemic an “infodemic” [8], and disinformation posing a public health threat, a closer examination of Wikipedia and its references during the pandemic is merited.

Wikipedia’s “COVID-19 pandemic” article was among the most viewed in 2020 [9]—with a peak interest during the first wave. Researchers from different disciplines have looked into citations in Wikipedia and performed bibliometric analyses of it—e.g., asking whether open-access papers are more likely to be cited in Wikipedia [10]. While anecdotal research has shown that Wikipedia and its academic references can mirror the growth of a scientific field [11], few have researched the coronavirus and Wikipedia. Research focused on Wikipedia and COVID-19 has shown both that traffic to Wikipedia’s coronavirus articles reflected public interest in the pandemic [12] and that these articles cite a representative sample of COVID-19 research [13]. However, to our knowledge, no research has yet focused on the bibliometrics of COVID-19 references on Wikipedia—be they popular or academic. These sources serve as the bridge between science and trusted facts on the one hand and public interest on the other. Examining their dynamics on Wikipedia is key for understanding the online knowledge ecosystem during a crucial phase of the pandemic and infodemic.

The aim of the present study was to provide a comprehensive bibliometric analysis of English Wikipedia’s COVID-19 articles during the pandemic’s first wave. To characterize the scientific literature as well as general media sources supporting the encyclopedia’s coverage of COVID-19 we performed citation analyses of the references used in Wikipedia’s coronavirus articles. We did this along 3 axes: the relevant articles’ references at the end of the first wave, their historical trajectory, and their network interaction with other Wikipedia articles on this topic.

## Material and Methods

Using citations as a metric for gauging the scientific character of Wikipedia articles along these 3 aforementioned axes allowed us to characterize the references and understand the pandemic’s effect on them. It also allowed us to ask what was the percentage of academic citations among any given article and what shifts they underwent during the period researched. This allowed us to gain a historical perspective on the scientific infrastructure supporting them, gauging the amount of time that passed between a scientific study’s publication and its being referenced on Wikipedia. Moreover we explored Wikipedia articles’ revisions (i.e., their edit history) and co-citations. This allowed us to gain insight into the representation of COVID-19 knowledge on Wikipedia and its growth since the creation of the digital encyclopedia in 2001 and up until 2020. Although predominantly qualitative, for some selected articles we also examined the different claims the citations were used to support at different stages, and reviewed some of the textual changes that articles underwent in the wake of the coronavirus outbreak, to provide anecdotal context for our findings.

### Corpus delimitation

Throughout the text, we used “articles” to denote Wikipedia entries and “papers” to denote academic studies referenced on Wikipedia articles. DOIs were used to identify academic sources among the references found within any given Wikipedia article. To delimit the corpus of Wikipedia COVID-19 articles containing DOIs, 2 different strategies were applied (Supplementary Fig. S1A). Every Wikipedia article affiliated with the official WikiProject COVID-19 (a volunteer-run task force overseeing >1,500 articles during the period analyzed) was scraped using an R package specifically developed for this study, WikiCitationHistoRy [14]. In combination with the WikipediR R package [15], which was used to retrieve the list of actual articles covered by the COVID-19 project, our WikiCitationHistoRy R package was used to extract DOIs from their text and thereby identify Wikipedia pages containing academic citations. Simultaneously, we also searched the EuroPMC database, using “COVID-19," “SARS-CoV2," and “SARS-nCoV19" as keywords to detect scientific studies published about this topic. Thus,  30,000 peer-reviewed papers, reviews, and preprints were retrieved. This set was compared to the DOI citations extracted from the entirety of the English Wikipedia dump of May 2020 (∼860,000 DOIs) using mwcite [16]. Thus, Wikipedia articles containing ≥1 DOI citation related to COVID-19 were identified—either from the EuroPMC search or through the specified Wikipedia project. The resulting “COVID-19 corpus” comprised a total of 231 Wikipedia articles, all related to COVID-19, which included ≥1 academic source. In this study, the term “corpus” describes this body of Wikipedia “articles,” and “sets” is used to describe a collection of “papers” (i.e., DOIs) and their related bibliographic information.

### DOI content analysis and set comparison

The analysis of DOIs led to the categorization of 3 DOI sets: (i) the COVID-19 Wikipedia set, (ii) the EuroPMC 30K search, and (iii) the Wikipedia dump of May 2020. For the dump and the COVID sets, the latency (see below) was computed, and for all 3 sets we retrieved their scientific citations count (the number of times the paper was cited in the scientific literature) and their Altmetric score, as well as the papers’ authors, publishers, journal, source type (preprint server or peer-reviewed publication), open-access status, title, and keywords. In addition, in the COVID-19 Wikipedia corpus the DOI set’s citation count on Wikipedia was also analysed to help gauge the importance of the sources within the online encyclopedia.

### Text mining, identifier extraction, and annotation

From the COVID-19 corpus, DOIs, PMIDs, ISBNs, and URLs (Supplementary Fig. S1B) were extracted using a set of regular expressions from our R package. Moreover WikiCitationHistoRy [14] allows the extraction of other sources such as tweets, press releases, reports, hyperlinks, and the protected status of Wikipedia pages (on Wikipedia, pages can be locked to public editing through a system of “protected” statuses). Subsequently, several statistics were computed for each Wikipedia article and information for each of their DOIs was retrieved using Altmetrics [17], CrossRef [18], and the EuroPMC [19] R packages.

### Visualizations and metrics

Our R package allows the retrieval of any Wikipedia article's content, both in the present—i.e., article text, size, reference count, and users—and in the past—i.e., timestamps, revision IDs, and the text of earlier versions. This package allows the retrieval of the relevant information in structured tables and helped support several data visualizations. Notably, 2 navigable visualizations were created for our corpus of Wikipedia articles: (i) A timeline [20] of article creation dates, which allows users to navigate through the growth of Wikipedia articles over time, and (ii) a network [21] linking Wikipedia articles based on their shared academic references. The package also includes a proposed metric to assess the scientific character of a Wikipedia article. This metric, called “Sci Score" (shorthand for “scientific score"), is defined as the ratio of academic as opposed to non-academic references that any Wikipedia article includes, as such:

(1) \begin{equation*}
\mathrm{ Sci}_{\mathrm{ Score}} = \frac{\# \mathrm{ DOI}}{\# \mathrm{ Reference}}
\end{equation*}

Our investigation also included an analysis of the latency [11] of any given DOI citation on Wikipedia. This metric is defined as the duration (in years) between the date of publication of a scientific paper and the date of introduction of the DOI into a specific Wikipedia article, as defined below:

(2) \begin{equation*}
\mathrm{ Latency} = \mathrm{ Date}_{\mathrm{ Wiki\ Introduction}}-\mathrm{ Date}_{\mathrm{ Publication}}
\end{equation*}

All visualizations and statistics were conducted using R statistical programming language (R version 3.5.0).

## Results

### COVID-19 Wikipedia articles: well-sourced but highly selective

We set out to characterize the representation of COVID-19–related research on Wikipedia. Because all factual claims on Wikipedia must be supported by “verifiable sources” [22], we focused on articles’ references to ask: What sources were used and what was the role of scientific papers in supporting coronavirus articles on Wikipedia? For this aim, we first identified the relevant Wikipedia articles related to COVID-19 (Supplementary Fig. S1A) as described in detail in the Material and Methods section. Then, we extracted relevant information such as identifiers (DOI, ISBN, PMID), references, and hyperlinks (Supplementary Fig. S1B).

From the perspective of Wikipedia, although there were >1.5k (1,695) COVID-19–related articles, only 149 had academic sources. We further identified an additional 82 Wikipedia articles that were not part of Wikipedia’s organic corpus of coronavirus articles but were in the Wikipedia dump and had ≥1 DOI reference from the EuroPMC database of >30,000 COVID-19–related papers (30,720) (Supplementary Fig. S1C). Together these 231 Wikipedia articles served as the main focus of our work because they form the scientific core of Wikipedia’s COVID-19 coverage. This DOI-filtered COVID-19 corpus included articles on scientific concepts, genes, drugs, and even notable people who fell ill with coronavirus. The articles ranged from “Severe acute respiratory syndrome-related coronavirus,” “Coronavirus packaging signal,” and “Acute respiratory distress syndrome” to “Charles, Prince of Wales,” “COVID-19 pandemic in North America,” and concepts with social interest like “Herd immunity,” “Wet market,” or even public figures like “Dr. Anthony Fauci.” This corpus included articles that had both scientific content–related topics of general public interest, e.g., the article for “Coronavirus,” the drugs “Chloroquine” and “Favipiravir,” and other non-scientific topics with wider social interest, like the article for “Social distancing” or “Shi Zhengli,” the virologist employed by the Wuhan Institute of Virology who earned public notoriety for her research into the origins of COVID-19.

Comparing the overall set of academic papers dealing with COVID-19 to those cited on Wikipedia we found that less than half a percent (0.42%) of all the academic papers related to coronavirus made it into Wikipedia (Supplementary Fig. S1C). Thus, our data reveals that Wikipedia was highly selective in regards to the existing scientific output dealing with COVID-19 (see Supplementary Dataset S1).

We next analyzed all the references included in the complete Wikipedia dump from May 2020, using mwcite [16] (Python package to extract references from Wikipedia dump). Thus, we could extract a total number of ∼2.68 million citations (2,686,881) comprising ISBNs, DOIs, arXiv, PMID, and PMC numbers (Supplementary Fig. S1D). Among the citations extracted were 860k DOIs and ∼38k preprint IDs from arXiv, ∼1.4% of all the citations in the dump, indicating that this server also contributes sources to Wikipedia alongside established peer-reviewed journals. These DOIs were used as a separate group that was compared with the EuroPMC 30k DOIs (30,720) and the extracted DOIs (2,626 unique DOIs) from our initial Wikipedia COVID-19 set in a subsequent analysis, thus forming the 3 aforementioned sets.

**Figure 1:**
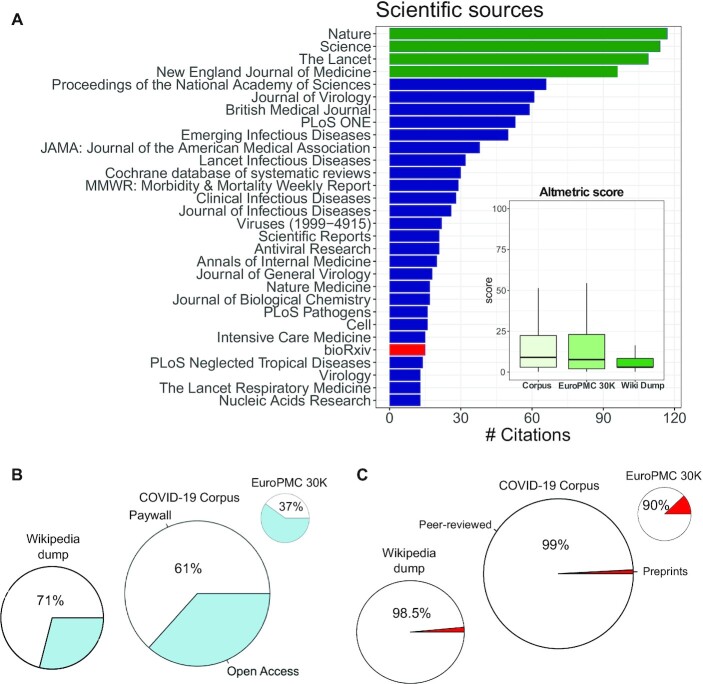
Characterization of scientific sources of the Wikipedia COVID-19 corpus. (A) Bar plot of the most cited academic sources. Top journals are highlighted in green and preprints are represented in red. *Bottom right:* Box plot of Altmetrics score of the 3 sets: the Wikipedia COVID-19 corpus, the EuroPMC COVID-19 search, and the full Wikipedia dump as of May 2020. Comparison of the occurrence of (B) open-access sources and (C) preprints (medRxiv and bioRxiv) in the 3 sets. Boxplots center indicates the median, and the bottom and top edges indicate the 25th and 75th percentiles; the wiskers extend 1.5 times the interquartile range.

Analysis of the journals and academic content from the set of 2,626 DOIs that were cited in the Wikipedia COVID-19 corpus revealed a strong bias towards high impact factor journals in both science and medicine. For example, *Nature*—which has an impact factor of >42—was among the top cited journals, alongside *Science, The Lancet*, and the *New England Journal of Medicine*; together these 4 comprised 13% of the overall academic references (Fig. 1A). The Cochrane Library database of systematic reviews was also among the most cited academic sources, likely because the WikiProject Medicine (WPM) and Cochrane have an official partnership. Notably, the papers cited were mostly those published in high impact factor journals, and were also found to have a higher Altmetric score compared to the overall average of papers cited in Wikipedia. In other words, the papers cited in Wikipedia’s COVID-19 articles were not just academically respected but were also popular—i.e., they were shared extensively on social media such as Twitter.

Importantly, more than one-third of the academic sources (39%) referenced in COVID-19 articles on Wikipedia were found to be open-access papers (Fig. 1B). The relation between open-access and paywalled academic sources is especially telling when compared to Wikipedia’s references writ large: ∼29% of all academic sources on Wikipedia are open-access, compared to 63% in the COVID-19–related scientific literature (i.e., in EuroPMC).

Remarkably, despite a surge in COVID-19 research being uploaded to preprint servers, we found that only a fraction of this new output was cited on Wikipedia: <1%, or 27 bioRxiv or medRxiv preprints were referenced (Fig. 1C, Supplementary Table S1). Among the COVID-19 preprints cited on Wikipedia was an early study on remdesivir [23], a study on the mortality rate of elderly individuals [24], research on COVID-19 transmission in Spain [25] and New York [26], and research into how Wuhan’s health system attempted to contain the virus [27]. This shows how non–peer-reviewed studies touched on medical, health, and social aspects of the virus—with 2 of the preprints, for example, focusing on the benefits of contact tracing [28,29]. The number of overall preprints was slightly lower than the general representation of preprints in Wikipedia (1.5%), but much lower than would be expected considering the fact that our academic database of EuroPMC papers had almost 3,700 preprints—12.3% of the ∼30,000 COVID-19–related papers in May 2020. Thus, in contrast to the high enrichment of preprints in COVID-19 research, Wikipedia’s editors overwhelmingly preferred peer-reviewed papers to preprints. In other words, Wikipedia generally cited preprints more often than it was found to do so on the topic of COVID-19, while COVID-19 articles cited open-access papers by 10% more (from 29% to 39%). Taken together with the bias towards high-impact journals, our data suggest that open-access papers contributed significantly to Wikipedia’s ability both to stay up to date and to maintain high academic standards, allowing editors to cite peer-reviewed research despite other alternatives being available.

**Figure 2:**
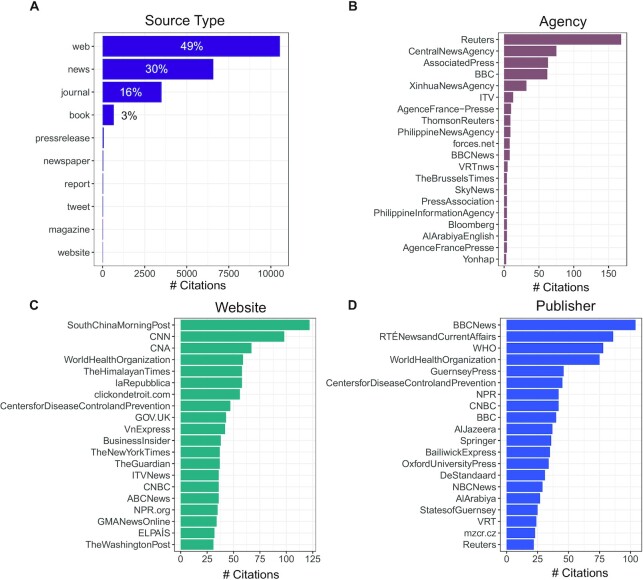
Top sources used in the Wikipedia COVID-19 corpus: A) source types, B) news agencies, C) websites, and D) publishers form the COVID-19 corpus sources (per Wikipedia’s citation template terminology). Several denominations for the same institution are present in the raw data which is highlighted here with the example of WHO and World Health Organization

We next focused on non-academic sources. Popular media, we found, played a substantial role in our corpus. More than 80% of all the references used in the COVID-19 corpus were non-academic, being either general media or websites (Fig. 2A). In fact, a mere 16% of the >21,000 references supporting the COVID-19 content were from academic journals. Among the general media sources used (Fig. 2B–D), there was a high representation for what is termed legacy media outlets, such as the New York Times and the BBC, alongside widely syndicated news agencies like Reuters and the Associated Press, and official sources like WHO.org and gov.UK. Among the most cited websites, for example, there was an interesting representation of local media outlets from countries hit early and hard by the virus, with the Italian La Repubblica and the South China Morning Postbeing among the most cited sites. The World Health Organization was one of the most cited publishers in the corpus of relevant articles, with >150 references.

### A scientific score for gauging scientific character

To distinguish between the roles that scientific research and popular media played, we created a “scientific score” for Wikipedia articles [1]. The metric is based on the ratio of academic as opposed to non-academic references that any article includes. This score attempts to rank the scientific character of any given Wikipedia article solely on the basis of its list of references. Ranging from 1 to 0, an article’s scientific score is calculated according to the ratio of its sources that are academic (i.e., contain DOIs), so that an article with 100% academic references will have a score of 1 while an article with none will have a score of 0. Technically, because all of our corpus of coronavirus-related Wikipedia articles had ≥1 academic source in the form of a DOI, their scientific scores will always be >0.

In effect, this score puts forth a metric for gauging the prominence of academic texts in any given article’s reference list. Of our 231 Wikipedia articles, 15 received a perfect scientific score of 1 (Supplementary Fig. S2A). High-scoring articles included the enzymes of “Furin” and “TMPRSS2”—whose inhibitor has been proposed as a possible treatment for COVID-19; “C30 Endopeptidase”—a group of enzymes also known as the “SARS coronavirus main proteinase”; and “SHC014-CoV”—a form of coronavirus that affects the Chinese rufous horseshoe bat.

In contrast to the articles on scientific topics and even biographical articles about scientists themselves, which both had high scientific scores, those with the lowest scores (Supplementary Fig. S2B) seemed to focus almost exclusively on social aspects of the pandemic or its immediate outcome. For example, the articles with the lowest scores dealt directly with the pandemic in a local context, including articles about the pandemic in Canada, North America, Indonesia, Japan, or even Jersey, to name a few. Others focused on different ramifications of the pandemic, e.g., the “Impact of the COVID-19 pandemic on the arts and cultural heritage” or “Travel restrictions related to the COVID-19 pandemic.” One of the articles with the lowest scientific scores was “Trump administration communication during the COVID-19 pandemic,” which made scarce use of coronavirus-related research to inform its content, citing a single academic paper (related to laws regulating quarantine) among its 244 footnotes.

### The price of remaining up to date on COVID-19

During the pandemic, there were tens of thousands of edits to the site, with thousands of new articles being created and scores of existing ones being re-edited and recast in the wake of new developments. Therefore, one could expect a rapid growth of articles on the topic, as well as a possible overall increase in the number of citations of all kinds. We sought to explore the temporal axis of Wikipedia’s coverage of the pandemic to see how COVID-19 articles and their academic references developed over time and were affected by the outbreak.

**Figure 3:**
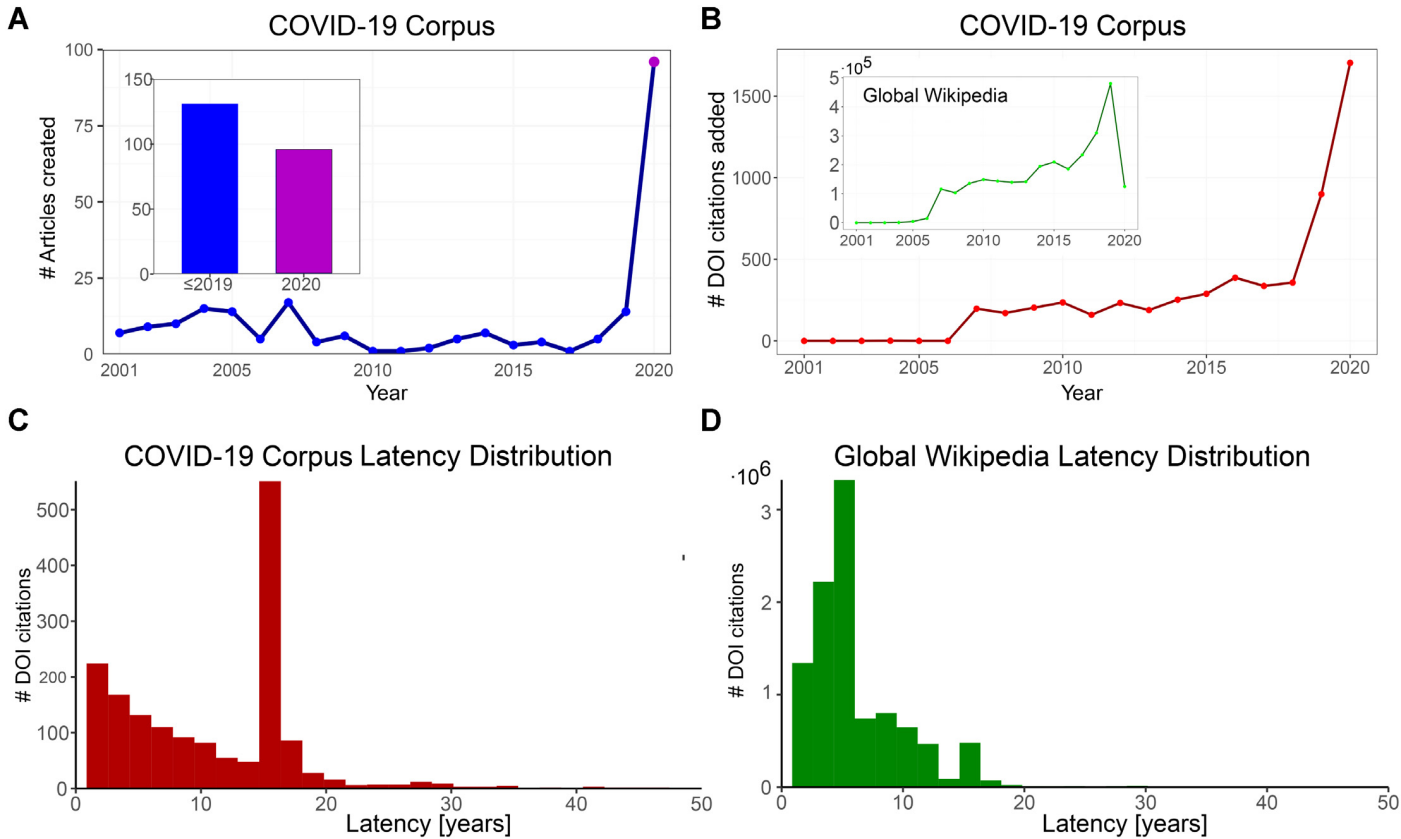
Historical perspective of the Wikipedia COVID-19 corpus. (A) COVID-19 article creation per year; inset: number of articles created before and after 2020. (B) Scientific citations added per year to the COVID-19 corpus and globally in Wikipedia (inset). Latency distribution of scientific papers (C) in the COVID-19 corpus and (D) the Wikipedia dump. See Supplementary Fig. S3 and in the GigaDB repository [54]. for an interactive version of the timeline.

First, we laid out our corpus of 231 articles across a timeline according to each article’s respective date of creation (Supplementary Fig. S3). An article count starting from 2001, when Wikipedia was first launched, and up until May 2020 shows that for many years there was a relatively steady growth in the number of articles that would become part of our corpus—until the pandemic hit, causing a massive peak at the start of 2020 (Fig. 3A). As the pandemic spread, the total number of Wikipedia articles dealing with COVID-19 and supported by scientific literature almost doubled—with a comparable number of articles being created before and after 2020 (134 and 97, respectively) (Fig. 3A, Supplementary Fig. S3).

The majority of the pre-2020 articles were created relatively early—between 2003 and 2006, likely linked to a general uptick in creation of articles on Wikipedia during this period. For example, the article for (the non-novel) “coronavirus” has existed since 2003, the article for the medical term “Transmission” and that of “Mathematical modeling of infectious diseases” from 2004, and the article for the “Coronaviridae” classification from 2005. Articles opened in this early period tended to focus on scientific concepts—e.g., those noted above or others like “Herd immunity.” Conversely, the articles created after the start of the pandemic during 2020 tended to be local or focused on the virus’s effects and social ramifications. Therefore, we collectively term the first group Wikipedia’s “scientific infrastructure” because they allowed new scientific information to be added into existing articles, while new ones focusing on the pandemic’s social significance were also being created.

The pre-pandemic articles tended to have a high scientific score—e.g., “Chloroquine,” which has been examined as a possible treatment for COVID-19. This article is one of many that underwent a shift in content in the wake of the pandemic, seeing both a surge in traffic and a surge in editorial activity (Supplementary Fig. S4). Per a subjective reading of this article’s content and the editorial changes it underwent during this period, much of the scientific content that was present before the pandemic was found to have remained intact, with new coronavirus-related information being integrated into the framework provided by existing content. The same occurred with many social concepts retroactively affiliated with COVID-19. Among these we can note the articles for “Herd immunity,” “Social distancing,” and the “SARS conspiracy theory” that also existed prior to the outbreak and served as part of Wikipedia’s scientific infrastructure, allowing new information to be contextualized.

In addition to the dramatic increase in article creation during the pandemic, there was also an increase in the overall number of references in articles affiliated with COVID-19 on Wikipedia (Fig. 3B). In fact, the number of DOIs added to these articles increased almost 6-fold from 2020 on—from ∼250 to almost 1,500 citations. Although most of the citations added were not just academic ones, with URLs overshadowing DOIs as the leading type of citation added, the general increase in citations can be seen as indicative of scientific literature’s prominent role in COVID-19 when taking into account that general trend in Wikipedia: The growth rate of references on COVID-19 articles was generally static until the outbreak; but on Wikipedia writ large references were on a rise since 2006. The post-2020 surge in citations was thus both academic and non-academic (Supplementary Fig. S5A).

One could hypothesize that a rapid growth of articles dedicated to coronavirus would translate to an overall decrease in the presence of academic sources because Wikipedia can create newer articles faster than academic research can be published on current events.

Examining the date of publication of the peer-reviewed studies referenced on Wikipedia shows that new COVID-19 research was cited alongside papers from previous years and even the previous century, the oldest being a 1923 paper titled the “The Spread of Bacterial Infection. The Problem of Herd-Immunity.” [30]. Overall, among the papers referenced on Wikipedia were highly cited studies, some with thousands of citations (Supplementary Table S2), but most had relatively low citation counts (median citation count for a paper in the corpus was 5). Comparing between a paper’s date of publication and its citation count reveals there is a low anti-correlation (−0.2) but highly significant between the two (Pearson product-moment correlation test *P*-value < 10^−15^, Supplementary Fig. S5B). This suggests that on average older scientific papers have a higher citation count; unsurprisingly, the more time that has passed since publication, the bigger the chances a paper will be cited.

Comparing the pre- and post-2020 articles’ scientific scores reveals that on average, the new articles had a mean score of 0.14, compared to the pre-2020 group’s mean of 0.48 and the overall mean of 0.30 (Supplementary Fig. S5C). Reading the titles of the 2020 articles to glean their topic and reviewing their respective scientific score can also point to a generalization: the more scientific an article is in topic, the more scientific its references are—even during the pandemic. This means that despite the dilution at a general level during the first months of 2020, articles with scientific topics created during this period did not pay that heavy of an academic price to stay up to date.

How did Wikipedia manage to maintain the quality of academic sourcing throughout the first wave of the pandemic? One possible explanation is that among the academic papers added to Wikipedia in 2020 were also papers published prior to this year if not a long time before. To investigate this hypothesis we used the latency metric (namely, the lag between a paper’s publication and its integration into Wikipedia, see equation [2]). We found the mean latency of Wikipedia’s COVID-19 content to be 10.2 years (Fig. 3C), slower than Wikipedia’s overall mean of 8.7 (Fig. 3D). In fact, in the coronavirus corpus we observed a peak in latency of ∼17 years—with >500 citations being added to Wikipedia 17 years after their initial academic publication—almost twice as slow as Wikipedia’s average. Interestingly, this time frame corresponds to the SARS outbreak (SARS-CoV-1) in 2002–2004, which yielded a boost of scientific literature regarding coronaviruses. This suggests that while there was a surge in editing activity during this pandemic that saw papers published in 2020 added to the COVID-19 articles, a large and even prominent role was still permitted for older literature. Viewed in this light, older papers played a similar role to pre-pandemic articles, giving precedence to existing knowledge in ordering the integration of new knowledge on scientific topics.

Comparing the articles’ scientific score to their date of creation portrays Wikipedia’s scientific infrastructure and its dynamics during the pandemic (Supplementary Fig. S5C). It reveals that despite maintaining high academic standards, citing papers published in prestigious and high impact factor journals, the need to stay up to date with COVID-19 research did come at some cost: most of the highest scoring articles were ones created before the pandemic (especially during 2005–2010) and newer articles generally had a lower scientific score (Supplementary Fig. S5C).

### Networks of COVID-19 knowledge

**Figure 4:**
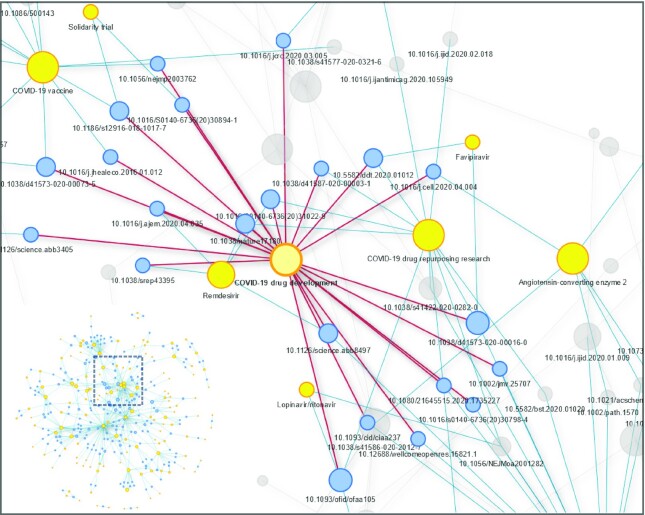
Network of articles–scientific papers (DOI) in the Wikipedia COVID-19 corpus. A network mapping scientific papers (with DOIs) cited in >1 article in the Wikipedia COVID-19 corpus was constructed. This network is composed of 454 edges, 179 DOIs (blue), and 136 Wikipedia articles (yellow). Nodes represent articles and their size is proportional to the number of connections. A zoom in on the cluster of Wikipedia articles dealing with COVID-19 drug development is depicted here for illustrative purposes. For clarity, edges marked in red indicate those connecting the DOIs cited directly in the “COVID-19 drug development” article and edges marked in blue indicate those connecting these DOIs to other articles citing them. See the GigaDB repository [54] for an interactive version of the network (see Supplementary Dataset S2).

To further investigate Wikipedia’s scientific sources and the infrastructure they provided, we built a network of Wikipedia articles linked together on the basis of their shared academic (DOI) sources. We filtered the list of papers (extracted DOIs) to keep those that were cited in ≥2 different Wikipedia articles and found 179 that fulfilled this criterion and were mapped to 136 Wikipedia articles in 454 different links (Fig. 4, Supplementary Data S2). This allowed us to map how scientific knowledge related to COVID-19 played a role not just in specific articles created during or prior to the pandemic but actually formed a web of knowledge that proved to be an integral part of Wikipedia’s scientific infrastructure. Similar to the timeline described above and as a subset of our COVID-19 corpus, Wikipedia articles belonging to this network included those dealing with people, institutions, regional outcomes of the pandemic, and scientific concepts—e.g., those regarding the molecular structure of the virus or the mechanism of infection (“C30 Endopeptidase," “Coronaviridae,” and “Airborne disease”). It also included a number of articles regarding the search for a potential drug to combat the virus or other possible interventions against it, with topics like social distancing, vaccine development, and drugs in current clinical trials.

Interestingly, we observed 6 prominent Wikipedia articles as key nodes in this network. These shared multiple citations with many other pages through DOI connections (nodes with an elevated degree). Of these 6 major nodes, 4 had a distinct and broad topic: “Coronavirus,” which focused on the virus writ large; “Coronavirus disease 2019,” which focused on the pandemic; and “COVID-19 drug repurposing research” and “COVID-19 drug development.” The first 2 articles were key players in how Wikipedia presented its coverage of the pandemic to readers: both were linked to from the main coronavirus article (“Coronavirus disease 2019"), which was placed on the English Wikipedia home page in a community-led process known as “In the News,” which showcases select articles on current events on the website’s home page. Later on, alongside this process led by the volunteers of the WikiProject COVID-19 task force, the Wikimedia Foundation (the WMF is the non-profit that oversees the Wikipedia project) also issued a directive to place a special banner referring to the “Coronavirus disease 2019” article on the top of every single article in English, driving millions to the article and to subsequent articles linking out from it. As noted, these articles—“Coronavirus disease 2019” and the articles linking out from it—were part of our DOI network. The fact that this central article shared citations with other articles that linked out from it, as described in our network, highlights the interconnecting role that academic citations played in Wikipedia’s COVID-19 coverage, allowing academic sources to support both popular and scientific articles and providing the public with access to high-quality sources in different contexts.

The 2 remaining nodes were similar and did not prove to be distinctly independent concepts but rather interrelated ones, with the articles for “Severe acute respiratory syndrome– related coronavirus” and “Severe acute respiratory syndrome coronavirus” each appearing as their own node despite their thematic overlap. It is also interesting to note that 4 of the 6 Wikipedia articles that served as the respective centers of these groups were locked to public editing as part of the protected page status (see Supplementary Data S3). These were all articles linked to the WPM or, at a later stage, to the specific offshoot project set up as a task force to deal with COVID-19.

The main themes that emerge from the network are those of COVID-19–related drugs and of the disease itself. Unlike articles relating to the effect of the pandemic, which as shown above were predominantly based on popular media, these 2 were topics that did require a scientific basis to be reliable. Reliability in this context is defined on Wikipedia by the WPM as accordance with its MEDRS policy—shorthand for “MEDical Reliable Sources.” The sourcing policy, which is Wikipedia’s most rigid, bans primary sources. Instead, MEDRS demands that medical and health claims cite meta-analysis or secondary sources that provide an overview of existing research or multiple–case-study clinical trials [31]. This policy is facilitated by the WPM’s aforementioned partnership with the Cochrane Library. The fact that popular articles like “Coronavirus disease 2019” or “COVID-19 drug development” shared academic citations with other articles underscores the important role that academic publications play on Wikipedia, creating the web of knowledge that our network describes. Furthermore, it highlights how the editing community’s centralized efforts (both articles were locked [Supplementary Dataset S3] and fell under the oversight of Wikipedia’s volunteer-run COVID-19 task force) allowed certain academic studies to find a role both in popular articles and in scientific articles linking out from them.

In our network analysis, an additional smaller group of nodes (with a lower degree) was also found. It had to do almost exclusively with China-related issues. As such, it exemplified how Wikipedia’s sourcing policy—which has an explicit bias towards peer-reviewed studies and is enforced exclusively by the community—may play a key role in fighting disinformation. For example, the academic paper that was most cited in Wikipedia’s COVID-19 articles was a paper published in *Nature* in 2020, titled “A pneumonia outbreak associated with a new coronavirus of probable bat origin” (Supplementary Table S3). This paper was referenced in 8 different Wikipedia articles, 2 among which dealt directly with scientific topics—“Angiotensin-converting enzyme 2” and “Severe acute respiratory syndrome coronavirus 2”—and 2 dealing with what can be termed parascientific terms linked to COVID-19—the “Wuhan Institute of Virology” and “Shi Zhengli.” This serves to highlight how contentious issues with a wide interest for the public—in this case, the origin of the virus—receive increased scientific support on Wikipedia, perhaps as a result of editors attempting to fend off misinformation supported by lesser, non-academic sources. Of the 5 most cited papers inside the COVID-19 corpus (Supplementary Table S3) 3 focused specifically on either bats or the virus’s animal origins, and another focused on its spread from Wuhan. Interestingly, 1 of the 27 preprints cited (Supplementary Table S1) was also the first study to suggest that the virus’s origin lay with bats [32].

Taken together with the previous findings regarding high-quality academic sources, centralized efforts in the form of locking articles did not just allow the enforcement of a rigid sourcing policy by the task force’s editors but also created a filtered knowledge funnel of sorts, which harnessed Wikipedia’s pre-existing infrastructure of articles, mechanisms, and policies to allow a regulated intake of new information as well as the creation of new articles, both based on existing research.

## Discussion

In the wake of the COVID-19 pandemic, characterizing scientific research on English-language Wikipedia and understanding the role that it plays is both important and timely. Millions of people—both medical professionals and the general public—read about health online [1]. Research has shown that traffic to Wikipedia articles follows topics covered in the news [33]—a dynamic that played out during the pandemic’s first wave [12]. Moreover, scientometric research has shown that academic research follows a similar pattern—with a surge of new studies during a pandemic followed by a decrease after it wanes [34]. During a pandemic the risk of disinformation on Wikipedia’s content is more severe, as it was during the Zika and SARS outbreaks [35]. Thus, throughout the COVID-19 pandemic, the threat was hypothetically increased because a surge in traffic to Wikipedia articles, research has found, often translates into an increase in vandalism [36]. Moreover, research into medical content on Wikipedia found that people who read health articles on the open encyclopedia are more likely to hover over, and thus possibly read, their references [37].

Particularly in the case of the coronavirus outbreak, the content on Wikipedia could have taken on potentially lethal consequences as the pandemic was deemed to be an "infodemic," and false information related to the virus was deemed a real threat to public health by the UN and WHO [8]. So far, most research into Wikipedia has revolved either around the quality, readership, or editorship of its health articles—or about references and sourcing in general. Meanwhile, research on Wikipedia and COVID-19 has focused almost exclusively on editing patterns and users' behaviours [12], or the representativity of academic citations [13]. Therefore, we deployed a comprehensive bibliometric analysis of COVID-19–related Wikipedia articles, focusing on articles’ text and sources, their growth over time, and their network relations.

Perhaps counterintuitively, we found that despite the traffic surge, these articles relied on high-quality sources, from both popular media and academic literature. Although the proportion of academic references in newly created articles did decrease in comparison to the period before the pandemic (resulting in a lower scientific score), we found that they still played a prominent role and that high editorial standards were generally maintained, utilizing several unique solutions that we herein attempt to outline and discuss.

One possible key to Wikipedia’s success had to do with the existence of centralized oversight mechanisms by the community of editors that could be quickly and efficiently deployed. In this case, the existence of the WPM—one of Wikipedia’s oldest community projects—and the formation of a specific COVID-19 task force in the form of WikiProject COVID-19 helped harness exiting editors and practices such as locking articles to safeguard quality across large swaths of articles and thus enforce a relatively unified sourcing policy on those dealing with both popular and scientific aspects of the virus.

In general, all factual claims on Wikipedia need to be supported by a verifiable source. Specifically, biomedical articles affiliated with the WPM are bound by a policy known as MEDRS (which requires meta-analysis or secondary sources for medical content [31]). However, the mere existence of this policy does not necessarily mean that it is respected. Our findings indicate that this policy, aided by the infrastructure provided by the community to enforce it, likely played a key role in regulating the quality of coronavirus articles. One mechanism used generally by the WPM to enforce the MEDRS sourcing standards and specifically deployed by the COVID-19 task force during the pandemic was locking articles to public editing (protected pages, Supplementary Dataset S3). This is a technique that is used to prevent vandalism on Wikipedia [38] and is commonly used when news events drive large amounts of new readers to specific Wikipedia articles, increasing the risk of substandard sources and content being added into the article by editors unversed in Wikipedia’s standards. This ad hoc measure of locking an article, deployed by a community vote on specific articles for specific amounts of time, prevents anonymous editors from being able to contribute directly to an article’s text and forces them to work through an experienced editor, thus ensuring editorial scrutiny. This measure is in line with our findings that many of the COVID-19 network central nodes were locked articles.

Another possible key to Wikipedia’s ability to maintain high quality sources during the pandemic was the existence of a specific infrastructure related directly to sourcing that could be enforced by the volunteer task force. The WPM has formed institutional-level partnerships to provide editors with access to reputable secondary sources that are in line with the MEDRS policy on medical and health topics—namely, through its cooperation with the Cochrane Library. The Cochrane Reviews database is available to Wikipedia’s medical editors, and it offers them access to systematic literature reviews and meta-analyses summarizing the results of multiple medical research studies [39]. As well as the existence of this database on medical content, the practice of providing access to high-quality sources was also deployed specifically in regards to coronavirus in the form a list of “trusted” sources provided to volunteers of the task force on the WikiProject COVID-19 project page. Alongside Cochrane studies, the WHO, for example, was given special status and preference [40]. This was evident in our results, with Cochrane sourcing being prominent, and the WHO being found to be among the most cited publishers on the COVID-19 articles. Also among the most cited scientific sources were others that were promoted by the task force as preferable sourcing for COVID-19 content: e.g., Science, Nature, and The Lancet. This indicates that the list of sources recommended by the task force were actually used by the volunteers and thus underscores the connection between our findings and the existence of a centralized community effort.

This was also true for non-academic sources: Among general media sources that the task force endorsed were Reuters and the New York Times, which were also prominently represented in our findings. Because each new edit to any locked COVID-19 article needed to be vetted by an experienced volunteer editor before it could go online within the body of an article’s text, the influx of new information being added was slowed down and regulated. Together with the source list, this allowed an especially strict sourcing policy to be rigorously implemented across thousands of articles. This was true despite the fact that there is no academic verification for volunteers—in fact, research suggests that less than half of Wikipedia’s editors focused on health and medical issues are medical professionals [3,4]—meaning that the task forces and its list of sources allowed non-experts to enforce academic-level standards.

This dynamic was also evident within articles with purely scientific content. Despite a deluge of preprints (both in general in recent years and specifically during the pandemic [41,42]), in our analysis, non–peer-reviewed academic sources did not play a key role in Wikipedia’s coronavirus content, while open-access papers did. Therefore, one could speculate that our finding that open-access papers were disproportionately cited may provide an explanation—with academic quality trumping speed, and editors opting against preprints and preferring published studies instead. Previous research has found that open-access papers are more likely to be cited on Wikipedia by 47% [10] and nearly one-third of the Wikipedia citations link to an open-access source [43]. Here we also saw that open access was prevalent in Wikipedia and even more so on COVID-19 articles. This, we suggest, allowed Wikipedia’s editors (expert or otherwise) to keep articles up to date without reverting to non–peer-reviewed academic content. This, one could suggest, was likely facilitated or at least aided by the decision by academic publications such as Nature and Science to lift their paywall and open public access to all of their COVID-19–related research papers, both past and present.

In addition to the communal infrastructure’s ability to regulate the addition of new information and maintain quality standards over time, another facet that we found to contribute to Wikipedia’s ability to stay accurate during the pandemic is what we term its scientific infrastructure. Research on Wikipedia articles’ content has shown that the initial structuring of information on a given article tends to dictate its development in later stages and that substantial reorganizations gradually decrease over time [44]. A temporal review of our articles and their citations showed that the best-sourced articles—those with the highest scientific score that formed the scientific backbone of Wikipedia’s COVID-19 content—were those created from 2005 and until 2010. These, we argue, formed Wikipedia’s scientific infrastructure, which regulated the intake of new knowledge into Wikipedia.

Our network analysis reflects the pivotal role that pre-existing content played in contextualizing the science behind many popular concepts or those made popular by the pandemic. Pre-existing content in the form of Wikipedia articles, policies, practices, and academic research served as a framework that helped regulate the deluge of new information, allowing newer findings to find a place within Wikipedia’s existing network of knowledge. Future work on this topic could focus on the question of whether this dynamic changed as 2020 progressed and, at a later time, on how contemporary peer-reviewed COVID-19–related research that was published during the pandemic’s subsequent waves would be integrated into these articles.

Previous research has suggested that in terms of content errors Wikipedia is on par with academic and professional sources even in fields like medicine [6]. A recent meta-analysis of studies about medical content on Wikipedia found that despite the prominent role that Wikipedia plays for the general public, health practitioners, patients, and medical students, the academic discourse around Wikipedia within the context of health is still limited [7]. This indicates that academic publications and scientists are lagging on embracing Wikipedia and its benefits. A change in this regard could help improve Wikipedia’s content and even introduce new editors with academic background into the fold, which would further improve quality and timeliness.

”Open” science practices that go beyond open access, for instance citizen scientists and open data, can also be translated to this and other contexts. In this regard, much like citizen scientists help support institutional science [45], Wikipedia’s editors may be regarded as citizen encyclopedists [11]. Viewed as such, Wikipedia’s citizen encyclopedists can play the same role communicating science that citizen scientists play in creating science. As previous citizen science projects have taught us [46], for that to work, citizens need scientists to provide the framework for non-expert contributions [47,48]. As this study indicates, a similar infrastructure can be seen to already exist on Wikipedia for encyclopedic (as opposed to scientific) work. Thus, should the cooperation between the scientific and Wikipedia communities increase, it could also be used for other contexts as well.

Our findings outline ways in which Wikipedia managed to fend off disinformation and stay up to date. With Facebook and other social media giants struggling to implement both technical and human-driven solutions against medical disinformation from the top down, it seems that Wikipedia’s dual use of established science and an open community of volunteers provides a possible model for how this can be achieved—a valuable goal during an infodemic. Some have already suggested that the U.S. Centers for Disease Control and Prevention should adopt Wikipedia’s model to help communicate medical knowledge [49]. In October 2020, the WHO and WMF announced that they would cooperate to make critical public health information available via an open licence. This means that in the near future, the quality of Wikipedia’s coverage of the pandemic will likely increase just as its role as central node in the network of knowledge transference to the general public becomes increasingly clear.

Wikipedia’s main advantage is in many ways its largest disadvantage: its open format, which allows a large community of editors of varying degrees of expertise to contribute. This can lead to large discrepancies in article quality and inconsistencies in the way editors add references to articles’ text [43]. We tried to address these limitations using technical solutions, such as regular expressions for extracting URLs, hyperlinks, DOIs, and PMIDs. In this study, which was limited to English, we retrieved most of our scientific literature metadata using Altmetrics [17,50], EuroPMC [19], and CrossRef [18] R APIs. However the content of the underlying databases is not always accurate, and at a technical level, this method was not without limitations. For example, we could not retrieve all of the extracted DOIs’ metadata. Moreover, information regarding open access (among others) varied with quality between the APIs [51]. In addition, our preprint analysis was mainly focused on MedRxiv and BioRxiv, which have the benefit of having a distinct DOI prefix. These collections make up the majority of preprints. However, others may also exist. Unfortunately, we found no better solution to annotate preprints from the extracted DOIs. Preprint servers do not necessarily use the DOI system [52] (i.e., ArXiv) and others share DOI prefixes with published papers (for instance the preprint server used by The Lancet). Moreover, we developed a parser for general citations on Wikipedia (categorized natively on the site as news outlets, websites, publishers), and we could not avoid redundant entries (i.e., “WHO”, “World Health Organisation”). In addition, our method to delimit the COVID-19 corpus focused on medical content (EuroPMC search) and may explain why we found predominately biomedical and health studies. Using DOI filtering on Wikipedia’s coronavirus articles should have equally led us to find papers from the social sciences—should those have been cited in this context. However, it seems that as these socially focused articles do not fall under the MEDRS sourcing policy, there was less if any use of academic studies, resulting in a low scientific score, thus further highlighting the importance of this policy in enforcing academic standards on the open encyclopedia’s articles.

Finally, as Wikipedia is constantly changing, some of our conclusions are bound to change. Our study is limited to focus on the pandemic’s first wave and its history on English Wikipedia alone, a crucial arena for examining the dynamics of knowledge online at a pivotal time frame. As these findings regarding the first wave were the result of a robust community effort that utilized English Wikipedia’s policies and mechanisms to safeguard existing content and regulate the creation of new content, it may be specific to English Wikipedia and its community. Nonetheless, it seems safe to speculate that at least on English Wikipedia, similar processes will continue to take place in the future as new textual additions are made to the open encyclopedia. In fact, one could speculate that as more time passes from the first wave, the newer post-pandemic articles that had low scientific scores will undergo a form of review and have their sources improved as newer research becomes more readily available. Studying the second wave—e.g., shifts in the scientific score over time—and understanding how encyclopedic content written during the first wave changed over the next year could be very instructive. Analyses of coronavirus articles indicated that at least on science, medical, and health topics—especially those in the news and driving public interest—Wikipedia’s methods for safeguarding its standards withstood the test. Perhaps as more academic research regarding the virus passes review and is published in 2021 and in the coming years, the ability of Wikipedia to reduce latency on this topic without having to compromise its scientific character will increase. Moreover, our findings hint that should journals open access to research in other fields, it may help Wikipedia cite even more peer-reviewed research instead of media sources or preprints. Thus, with the help of community enforcement, like that seen during the first wave of the pandemic, Wikipedia should be able to succeed in other fields as well.

In summary, our findings reveal a trade-off between timeliness and scientific character in regards to the scientific literature. Most of Wikipedia’s COVID-19 content was supported by references from highly trusted sources—but with the pandemic’s breakout, these were more from the general media than from academic publications. That Wikipedia’s COVID-19 articles were based on respected sources in both the academic and popular media was found to be true even as the pandemic and number of articles about it grew. Our investigation further demonstrates that despite a surge in preprints about the virus and their promise of cutting-edge information, Wikipedia preferred published studies, giving a clear preference to open-access studies. A temporal and network analysis of COVID-19 articles indicated that remaining up to date did come at a cost in terms of quality. It also showed how pre-existing content—both in the form of pre-pandemic articles and papers—helped regulate the flow of new information into existing articles. In future work, we hope the tools and methods developed here will be used to examine how these same articles fared over the entire span of 2020, as well as helping others use them for research into other topics on Wikipedia. We observed how Wikipedia used volunteer-editors to enforce rigid sourcing standards—and future work may continue to provide insight into how this unique method can be used to fight disinformation and to characterize the knowledge infrastructure in other arenas.

## Data Availability

All raw data and tables are available online through the Zenodo repository [53]. A beta version of the visualizations, their code, and the documentation from our R package are available on the GitHub repositories [14,20,21]. Supplementary information and datasets are available in the *GigaScience* GigaDB repository [54].

## Additional Files

**Supplementary Table S1**: Preprints cited within the Wikipedia COVID-19 Corpus.

**Supplementary Table S2**: Most cited scientific papers in the scientific literature within COVID-19 Wikipedia corpus.

**Supplementary Table S3**: Most cited scientific papers in COVID-19 Wikipedia corpus.

**Supplementary Figure S1**: Corpus identification and citation extraction pipeline. (A) Scheme of the corpus delimitation rationale and citation extraction. To delimit our corpus of Wikipedia articles containing DOI, we applied 2 different strategies. First we scraped every Wikipedia page from the COVID-19 Wikipedia project (∼3k pages) and we filtered them to keep only articles containing DOI citations (149 Wikipedia articles). For our second strategy, we searched the EuroPMC database for COVID-19, SARS-CoV2, SARS-nCoV19—yielding 30,000 scientific papers, reviews, and preprints. These were then compared to the citations extracted from the English Wikipedia dump of May 2020 (860,000 DOIs). Searching Wikipedia with the resulting set led us to identify an additional 91 Wikipedia articles containing ≥1 citation from the EuroPMC set. Taken together, from the resulting corpus of 231 Wikipedia articles, we extracted DOIs, PMIDs, ISBNs, websites, and URLs using a set of regular expressions, as described in the Materials and Methods section. Subsequently, we computed several statistics for each Wikipedia article and we retrieved Altmetrics, CrossRef, and EuroPMC information for each of their cited papers’ DOI. Finally, we produced tables of annotated citations and extracted information from each Wikipedia article such as books, websites, and newspapers. In addition, a timeline of Wikipedia articles and a network of Wikipedia articles linked by their shared scientific sources was produced. (B) Example of raw Wikipedia text from the “Social distancing” article, highlighted with several parsed items from a reference. Pink: a hyperlink to an image file; green: Wikipedia hyperlinks; purple: reference; yellow: citation type; dark green: citation title; red: citation date; orange: citation URL. (C) Overlap between DOIs from the Wikipedia dump and the 30k EuroPMC COVID-19–related scientific papers and preprints. (D) Number of extracted citations with mwcite from the English Wikipedia dump of May 2020.

**Supplementary Figure S2**: Articles from the Wikipedia COVID-19 corpus with (A) the highest and (B) lowest scientific scores. The scientific score was computed on the basis of the reference content of each article, as defined in the Material and Methods section.

**Supplementary Figure S3**: Timeline of the Wikipedia COVID-19 corpus articles, based on date of creation. See here for an interactive version of the timeline.

**Supplementary Figure S4**: Selected articles’ (A) page views and (B) edit counts during the first wave of COVID-19 pandemic (January–May 2020).

**Supplementary Figure S5**: Historical characterization of citations in the COVID-19 corpus. (A) Number of references on Wikipedia throughout time, parsed by different type of sources (DOI, ISBN, hyperlink, URL). (B) Number of citations in scientific literature as a function of the papers’ publication year. (C) Scientific score as a function of the creation date of Wikipedia article in the COVID-19 corpus.

**Supplementary Dataset 1**: Table of annotated DOI form europmc COVID-19 cited in Wikipedia.

**Supplementary Dataset 2**: Table of Wikipedia article-DOI network

**Supplementary Dataset 3**: Table of protected Wikipedia COVID-19 articles

## Abbreviations

API: Application Programming Interface; DOI: Digital Object Identifier; ISBN: International Standard Book Number; PMID: PubMed ID; URL: uniform resource locator; WHO: World Health Organization; WMF: Wikimedia Foundation; WPM: WikiProject Medicine.

## Competing Interests

Omer Benjakob is a journalist for Haaretz and has written about Wikipedia in the past.

## Funding

J.S. is a recipient of the Placide Nicod foundation, and R.A. is a recipient of the Azrieli Foundation fellowship. We are grateful for their financial support.

## Author's Contributions

J.S., O.B., and R.A. designed research; J.S., O.B., and R.A. performed research; J.S. analysed data and developed the software; and J.S., O.B., and R.A. wrote the paper. All authors approved the final version.

## Supplementary Material

giab095_GIGA-D-21-00139_Original_Submission

giab095_GIGA-D-21-00139_Revision_1

giab095_GIGA-D-21-00139_Revision_2

giab095_GIGA-D-21-00139_Revision_3

giab095_Response_to_Reviewer_Comments_Revision_2

giab095_Reviewer_1_Report_Original_Submission Dariusz Jemielniak -- 5/13/2021 Reviewed

giab095_Reviewer_2_Report_Original_Submission Dean Giustini -- 5/30/2021 Reviewed

giab095_Reviewer_3_Report_Original_Submission Daniel Mietchen -- 6/14/2021 Reviewed

giab095_Reviewer_3_Report_Revision_1 Daniel Mietchen -- 11/17/2021 Reviewed

giab095_Supplemental_Files
