# Supplementary material for: Citation needed? Wikipedia bibliometrics during the first wave of the COVID-19 pandemic
Source: Gigascience. 2022 Jan 12;11:giab095. doi: 10.1093/gigascience/giab095 (PMC8756189; doi:10.1093/gigascience/giab095)
Supplement: giab095_GIGA-D-21-00139_Original_Submission [file giab095_giga-d-21-00139_original_submission.pdf]

GigaScience

Citation needed? Wikipedia and the COVID-19 pandemic

--Manuscript Draft--

**Manuscript Number:**	GIGA-D-21-00139
**Full Title:**	Citation needed?<br>Wikipedia and the COVID-19 pandemic
**Article Type:**	Research
**Funding Information:**	
**Abstract:**	With the COVID-19 pandemic's outbreak at the beginning of 2020, millions across the world flocked to Wikipedia to read about the virus. Our study offers an in-depth analysis of the scientific backbone supporting Wikipedia's COVID-19 articles. Using references as a readout, we asked which sources informed Wikipedia's growing pool of COVID-19-related articles during the pandemic's first wave (January-May 2020). We found that coronavirus-related articles referenced trusted media sources and cited high-quality academic research. Moreover, despite a surge in preprints, Wikipedia's COVID-19 articles had a clear preference for open-access studies published in respected journals and made little use of non-peer-reviewed research uploaded independently to academic servers. Building a timeline of COVID-19 articles on Wikipedia from 2001-2020 revealed a nuanced trade-off between quality and timeliness, with a growth in COVID-19 article creation and citations, from both academic research and popular media. It further revealed how preexisting articles on key topics related to the virus created a framework on Wikipedia for integrating new knowledge. This "scientific infrastructure" helped provide context, and regulated the influx of new information into Wikipedia. Lastly, we constructed a network of DOI-Wikipedia articles, which showed the landscape of pandemic-related knowledge onWikipedia and revealed how citations create a web of scientific knowledge to support coverage of scientific topics like COVID-19 vaccine development. Understanding how scientific research interacts with the digital knowledge-sphere during the pandemic provides insight into how Wikipedia can facilitate access to science. It also sheds light on how Wikipedia successfully fended of disinformation on the COVID-19 and may provide insight into how its unique model may be deployed in other contexts.
**Corresponding Author:**	Jonathan Aryeh Sobel, Ph.D.<br>Technion Israel Institute of Technology<br>Haifa, ISRAEL
**Corresponding Author Secondary Information:**	
**Corresponding Author's Institution:**	Technion Israel Institute of Technology
**Corresponding Author's Secondary Institution:**	
**First Author:**	Omer Benjakob
**First Author Secondary Information:**	
**Order of Authors:**	Omer Benjakob
	Rona Aviram, Ph.D.
	Jonathan Aryeh Sobel, Ph.D.
**Order of Authors Secondary Information:**	
**Additional Information:**	
**Question**	**Response**
Are you submitting this manuscript to a	No

Powered by Editorial Manager® and ProduXion Manager® from Aries Systems Corporation

special series or article collection?	
**Experimental design and statistics**<br><br>Full details of the experimental design and statistical methods used should be given in the Methods section, as detailed in our Minimum Standards Reporting Checklist. Information essential to interpreting the data presented should be made available in the figure legends.<br><br>Have you included all the information requested in your manuscript?	Yes
**Resources**<br><br>A description of all resources used, including antibodies, cell lines, animals and software tools, with enough information to allow them to be uniquely identified, should be included in the Methods section. Authors are strongly encouraged to cite Research Resource Identifiers (RRIDs) for antibodies, model organisms and tools, where possible.<br><br>Have you included the information requested as detailed in our Minimum Standards Reporting Checklist?	Yes
**Availability of data and materials**<br><br>All datasets and code on which the conclusions of the paper rely must be either included in your submission or deposited in publicly available repositories (where available and ethically appropriate), referencing such data using a unique identifier in the references and in the "Availability of Data and Materials" section of your manuscript.<br><br>Have you have met the above requirement as detailed in our Minimum Standards Reporting Checklist?	Yes

Powered by Editorial Manager® and ProduXion Manager® from Aries Systems Corporation

Click here to access/download;Manuscript;BIORXIV-2021-433379v3-Sobel.pdf

Citation needed? Wikipedia and the COVID-19 pandemic

Omer Benjakob[1,*], **Rona Aviram**[2,*], **and Jonathan Sobel**[2,3,*]

[1]The Cohn Institute for the History and Philosophy of Science and Ideas, Tel Aviv University, Tel Aviv, Israel
[2]Weizmann Institute of Science, Rehovot, Israel
[3]Faculty of Biomedical Engineering, Technion-IIT, Haifa, Israel
[*]*These authors contributed equally to this work*

With the COVID-19 pandemic's outbreak at the beginning of 2020, millions across the world flocked to Wikipedia to read about the virus. Our study offers an in-depth analysis of the scientific backbone supporting Wikipedia's COVID-19 articles. Using references as a readout, we asked which sources informed Wikipedia's growing pool of COVID-19-related articles during the pandemic's first wave (January-May 2020). We found that coronavirus-related articles referenced trusted media sources and cited high-quality academic research. Moreover, despite a surge in preprints, Wikipedia's COVID-19 articles had a clear preference for open-access studies published in respected journals and made little use of non-peer-reviewed research uploaded independently to academic servers. Building a timeline of COVID-19 articles on Wikipedia from 2001-2020 revealed a nuanced trade-off between quality and timeliness, with a growth in COVID-19 article creation and citations, from both academic research and popular media. It further revealed how preexisting articles on key topics related to the virus created a framework on Wikipedia for integrating new knowledge. This "scientific infrastructure" helped provide context, and regulated the influx of new information into Wikipedia. Lastly, we constructed a network of DOI-Wikipedia articles, which showed the landscape of pandemic-related knowledge on Wikipedia and revealed how citations create a web of scientific knowledge to support coverage of scientific topics like COVID-19 vaccine development. Understanding how scientific research interacts with the digital knowledge-sphere during the pandemic provides insight into how Wikipedia can facilitate access to science. It also sheds light on how Wikipedia successfully fended of disinformation on the COVID-19 and may provide insight into how its unique model may be deployed in other contexts.

COVID-19 | Wikipedia | Infodemic | sources

Correspondence:
omerbj@gmail.com
anorona@gmail.com
jsobel83@gmail.com

Introduction

Wikipedia has over 130,000 different articles relating to health and medicine (1). The website as a whole, and specifically its medical and health articles, like those about disease or drugs, are a prominent source of information for the general public (2). Studies of readership and editorship of health articles reveal that medical professionals are active consumers of Wikipedia and make up roughly half of those involved in editing these articles in English (3, 4). Research conducted into the quality and scope of medical content deemed Wikipedia "a key tool for global public health promotion." (4, 5).

With the WHO labeling the COVID-19 pandemic an "infodemic" (6), and disinformation potentially affecting public health, a closer examination of Wikipedia and its references during the pandemic is merited. Researchers from different disciplines have looked into citations in Wikipedia, for example, asking if open-access papers are more likely to be cited in Wikipedia (7). While anecdotal research has shown that Wikipedia and its academic references can mirror the growth of a scientific field (8), and initial research into the coronavirus has shown Wikipedia provides a representative sample of COVID-19 research (9), to our knowledge no research has focused on the role of popular media and academic sources on Wikipedia during the pandemic.

Here, we asked what role does scientific literature, as opposed to general media, play in supporting the encyclopedia's coverage of the COVID-19 as the pandemic spread. We investigated this question throughout an analyses of Wikipedia's coronavirus articles along three axes: the references used in the relevant articles, their historical growths, and their network interaction. Our findings reveal that Wikipedia's COVID-19 articles were mostly supported by highly trusted sources - both from general media and academic literature. We found, for example, that these articles made more use of open-access papers than the average Wikipedia article, and did not overly refer to preprints. A temporal analysis of the references used in these articles reveals a more nuanced dynamic: after the pandemic broke out we observed a drop in the overall percentage of academic references in a given coronavirus article, used here as a metric for gauging scientificness in what we term an article's *Scientific Score*. Importantly, a time-course/historical analysis of these articles, revealed a surge in editing activity following the pandemic breakout, with many contemporary academic papers added to the COVID-19 articles, alongside frequent use of much older literature. A network analysis of the COVID-19 articles and their academic references revealed how certain topics, generally those pertaining directly to science or health (for example, drug development) and not the pandemic or its outcome, were linked together through shared sources. Together, these analyses depict what we term Wikipedia's scientific infrastructure, which included both content (e.g. past articles) and organizational practices (e.g. rigid sourcing policy), that can shed light on online

mechanisms to fend off disinformation and maintain high standards across articles.

Material and Methods

Corpus Delimitation. To delimit the corpus of Wikipedia COVID19-articles containing Digital Object Identifier (DOI), two different strategies were applied. Every Wikipedia article affiliated with the official WikiProject COVID-19 task force (more than 1,500 pages during the period analyzed) was scraped using an R package specifically developed for this study, *WikiCitationHistoRy*. In combination with the *WikipediR* R package, which was used to retrieve the actual articles covered by the COVID-19 project, our *WikiCitationHistoRy* R package extracted DOIs from their text and thereby identified Wikipedia pages containing academic citations, termed "Wikipedia articles" in the present study. While "articles" is used for Wikipedia entries, "papers" is used to describe academic studies referenced on Wikipedia articles, and these papers were also guaged for their "citation" count in academic literature. Simultaneously, a search of EuroPMC using *COVID-19*, *SARS-CoV2*, *SARS-nCoV19* keywords was performed to detect scientific studies published about this topic. Thus, 30,000 peer-reviewed papers, reviews, and preprint studies were retrieved. This set was compared to the DOI citations extracted from the entirety of the English Wikipedia dump of May 2020 (860,000 DOIs) using *mwcite*. Thus, Wikipedia articles containing at least one DOI citation were identified - either from the EuroPMC search or through the specified Wikipedia project. The resulting "COVID-19 corpus" comprised a total of 231 Wikipedia articles related to COVID-19 and based on at least one academic source; while "corpus" describes the body of Wikipedia articles, "sets" is used to describe the bibliographic information relating to academic papers (like DOIs).

DOI Corpus Content Analysis and DOI Sets Comparison. The analysis of DOIs led to the categorization of three DOI sets: 1) the COVID-19 Wikipedia set, 2) the EuroPMC 30K search and 3) the Wikipedia dump of May 2020. For the dump and the COVID sets, the latency was computed (to gauge how much time had passed from an article's publication until it was cited on Wikipedia), and for all three sets we retrieved their articles' scientific citations count (the number of times the paper was cited in scientific literature), their Altmetric score, as well as the papers' authors, publishers, journal, source type (preprint server or peer-reviewed publication), open-access status (if relevant), title and keywords. In addition, in the COVID-19 Wikipedia corpus the DOI set's citation count were also analysed to help gauge academic quality of the sources.

Text Mining, Identifier Extraction and Annotation. From the COVID-19 corpus, DOIs, PMIDs, ISBNs, and URLs were extracted using a set of regular expressions from our R package, provided in the Table 1. Moreover *WikiCitationHistoRy* allows the extraction of other sources such as tweets, press release, reports, hyperlinks and the *protected* status of Wikipedia pages (on Wikipedia, pages can be locked to public editing through a system of "protected" statuses). Subsequently, several statistics were computed for each Wikipedia article and information for each of their DOI were retrieved using *Altmetrics*, *CrossRef* and *EuroPMC* R packages.

<table>
<tr><th>Citation type</th><th>Regular expression</th></tr>
<tr><td>doi</td><td>10\\.\\d{4,9}/[-._;()/:a-z0-9A-Z]+</td></tr>
<tr><td>isbn</td><td>(?<=(isbn|ISBN)\\s?[=:]?\\s?)\\d{1,5}-\\d{1,7}-\\d{1,5}-[\\dX]</td></tr>
<tr><td>url</td><td>http[s]?://(?:[a-zA-Z]|[0-9]|[$-_@.&+]|[!*\\(\\),]|(?:%[0-9a-fA-F][0-9a-fA-F]))+</td></tr>
<tr><td>tweet</td><td>\\{\\{cite tweet.*?\\}\\}</td></tr>
<tr><td>news</td><td>\\{\\{cite news.*?\\}\\}</td></tr>
<tr><td>journal</td><td>\\{\\{cite journal.*?\\}\\}</td></tr>
<tr><td>web</td><td>\\{\\{cite web.*?\\}\\}</td></tr>
<tr><td>pmid</td><td>(?<=(pmid|PMID)\\s?[=:]\\s?)\\d{5,9}</td></tr>
<tr><td>ref</td><td><ref>\\{\\{[c|C]ite.*?\\}\\}</ref></td></tr>
</table>

. **Table 1** Regular expressions for each citation type.

Package Visualisations and Statistics. Our R package was developed in order to retrieve any Wikipedia article and its content, both in the present - i.e article text, size, citation count and users - and in the past - i.e. timestamps, revision IDs and the text of earlier versions. This package allows the retrieval of the relevant information in structured tables and helped support several visualisations for the data. Notably, two navigable visualisations were created and are available for any set of Wikipedia articles: 1) A timeline of article creation dates which allows users to navigate through the growth of Wikipedia articles related to a certain topic over time, and 2) a network linking Wikipedia articles based on their shared academic references. The package also includes a proposed metric to assess the scientificness of a Wikipedia article. This metric, called *Sci Score*, is defined by the ratio of academic as opposed to non-academic references any Wikipedia article includes, as such:

$$Sci_{Score} = \frac{\#DOI}{\#Reference} \tag{1}$$

Our investigation, as noted, also included an analysis into the latency (8) of any given DOI citation on Wikipedia. This metric is defined as the duration (in years) between the date of publication of a scientific paper and the date of introduction of the DOI into a specific Wikipedia article as defined below:

$$Latency = Date_{WikiIntroduction} - Date_{Publication} \tag{2}$$

Data and Code Availability Statement. Every table and raw data are available online through the ZENODO

repository with DOI: 10.5281/zenodo.3901741
Every visualisation and statistics were completed using R statistical programming language (R version 3.5.0). The code from our R package is available in the Github repositories:
https://github.com/jsobel1/WikiCitationHistoRy
https://github.com/jsobel1/Wiki_COVID-19_interactive_network
https://github.com/jsobel1/Interactive_timeline_wiki_COVID-19

Results

COVID-19 Wikipedia Articles: Well-Sourced but Highly Selective. We set out to characterize the representation of COVID-19-related research on Wikipedia. As all factual claims on Wikipedia must be supported by "verifiable sources" (10), we focused on articles' references to ask: What sources were used and what was the role of scientific papers about COVID-19 in supporting coronavirus articles on Wikipedia? For this aim, we identified the relevant Wikipedia articles related to COVID-19 (Supplementary figure S1A). We did this using a two-pronged approach (as described in detail in the methods section): firstly, searching Wikipedia's categories of coronavirus-related articles (i.e. articles directly affiliated with WikiProject COVID-19 or marked with the community-created COVID-19 template) for those that included at least one academic paper among their list of references. Secondly, we searched English Wikipedia's general body of articles' references (i.e. the dump of all of Wikipedia's articles' references) to find papers cited within the EuroPMC database of COVID-19 research (Supplementary figure S1A)). This approach allowed us to both filter out articles deemed to be about COVID-19 by the community but not based on any scientific research, and find articles that may not be labeled as being about COVID-19 but still relevant for our research as they include related research.

From the perspective of Wikipedia, though there were over 1.5K (1,695 pages) COVID-19 related articles, only 149 had academic sources. We further identified an additional 82 Wikipedia articles that were not part of Wikipedia's organic set of coronavirus articles, but had at least one DOI references from the EuroPMC database - which consisted of over 30,000 papers (30,720) S1B). Together these 231 Wikipedia articles served as the main focus of our work as they form the scientific core of Wikipedia's COVID-19 coverage. This DOI-filtered COVID-19 corpus included articles on scientific concepts, genes, drugs and even notable people who fell ill with coronavirus. The articles ranged from "Severe acute respiratory syndrome-related coronavirus", "Coronavirus packaging signal" and "Acute respiratory distress syndrome", to "Charles Prince of Whales", "COVID-19 pandemic in North America," and concepts with social interest like "Herd immunity", "Social distancing", "Wet market" or even public figures like "Dr. Anthony Fauci".

It is interesting to note that these articles included the article for "Coronavirus", the drugs "Chloroquine" and "Favipiravir," and other less scientific articles with wider social interest, like the article for "Social distancing" and "Shi Zhengli", the virologist employed by the Wuhan Institute of Virology and who earned public notoriety for her research into the origins of COVID-19. However, comparing the overall corpus of academic papers dealing with COVID-19 to those cited on Wikipedia we found that less than half a percent (0.42%) of all the academic articles related to coronavirus made it into Wikipedia (Supplementary figure S1C). Thus, our data reveals Wikipedia was highly selective in regards to the existing scientific output dealing with COVID-19 (See supplementary table (1)).

We next analyzed the citation content from the complete Wikipedia dump from May 2020, using *mwcite*. Thus, we could extract a total number of about 2.68 million citations (2,686,881) comprising ISBNs, DOIs, arXiv, PMID and PMC numbers (Supplementary figure S1D). Among the citations extracted were 860K DOIs and about 38K preprints IDs from arXiv, about 1.4 percent of all the citations in the dump, indicating that the server hosting non-reviewed studies does contribute sources to Wikipedia alongside established peer-review journals. These DOIs were used as a separate group that was compared with the EuroPMC 30K DOIs (30,720) and the extracted DOIs (2,626 unique DOI) from our initial Wikipedia COVID-19 set in subsequent analysis. In line with existing research (1, 8), an analysis of the journals and academic content from the 2,626 DOIs that were cited in the Wikipedia COVID-19 corpus reveals a strong bias towards high impact factor journals in both science and medicine. For example, *Nature* - which has an impact factor of over 42 - was among the top cited journals, alongside *Science*, *The Lancet* and the *New England Journal of Medicine*; together these four comprised 13 percent of the overall academic references (Figure 1A). Notably, the papers cited tended to not just to come from high impact factor journals, but also have a higher Altmetric score compared to the overall average of papers cited in Wikipedia in general and those scarped from the official dump. In other words, the papers cited on Wikipedia were not just academically respected but were also popular - i.e. they were shared extensively on social media such as Twitter and Facebook.
Most importantly perhaps, we also found that more than a third of the academic sources (39%) on COVID-19 articles on Wikipedia were open-access papers (Figure 1B). The relation between open-access and paywalled academic sources is especially interesting when compared to Wikipedia's references writ large: About 29 percent of all academic sources on Wikipedia are open-access, compared to 63 percent in the COVID-19-related scientific literature (i.e. in EuroPMC).
In the last decade, a new type of scientific media has emerged - preprints. These are non-peer-reviewed studies uploaded independently by researchers themsleves to online archives like

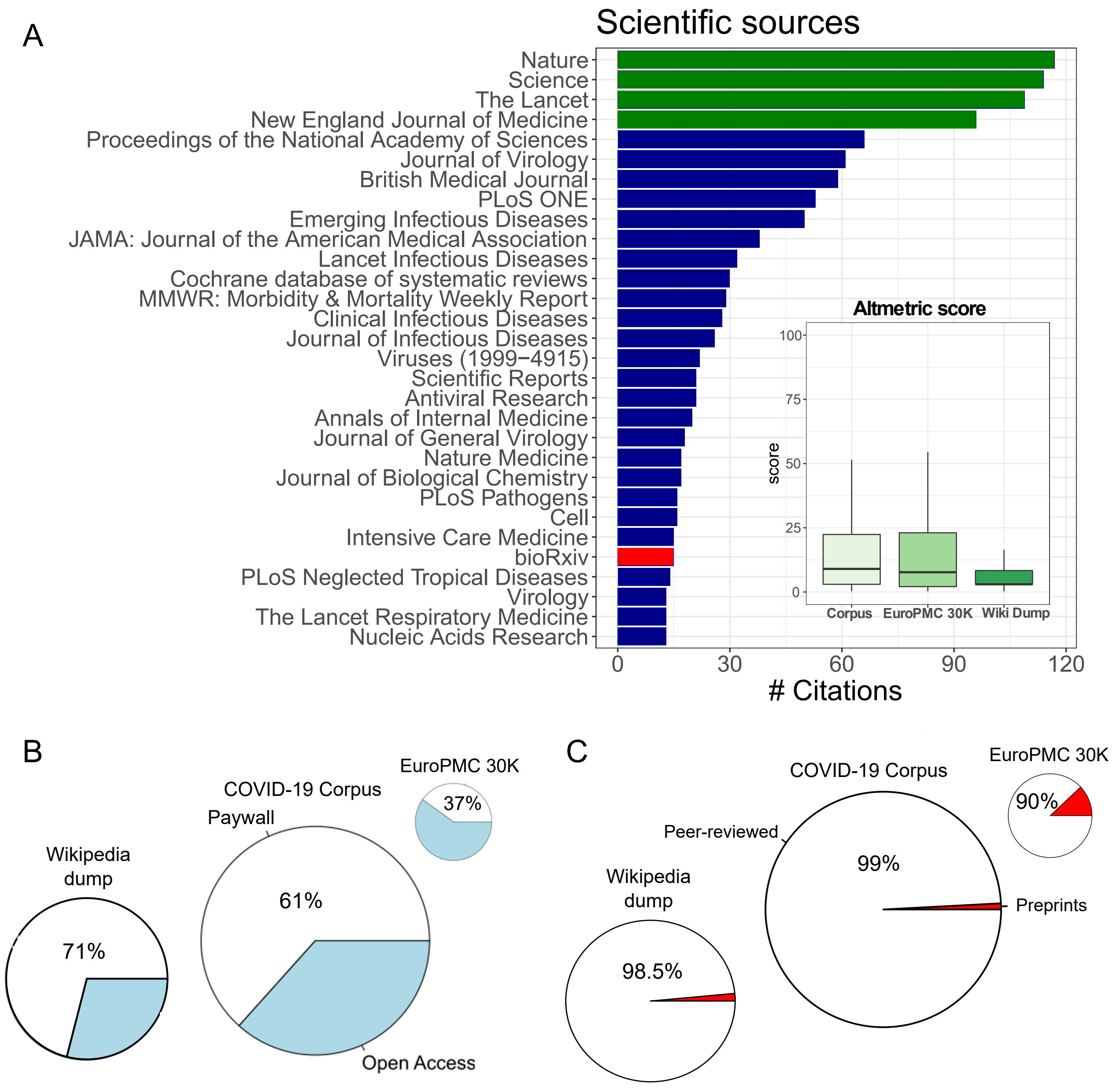

Figure 1. Wikipedia COVID-19 Corpus of scientific sources reveals a greater fraction of open-access papers as well as a higher impact in Altmetric score. A) Bar plot of the most trusted academic sources. Top journals are highlighted in green and preprints are represented in red. Bottom right: boxplot of the distribution Altmetrics score in Wikipedia COVID-19 corpus - the dump from May 2020, the COVID-19 Corpus and the scientific sources from the Europmc COVID-19 search. B) Fraction of open-access sources, C) fraction of preprints from BioRxiv and MedRxiv.

BioRxiv - the largest preprint database in life sciences and MedRxiv for medical research. Both of these digital archives saw a surge in COVID-19 related research during the pandemic (11). We therefore examined how this trend manifested on Wikipedia. Remarkably, despite a surge in COVID-19 research being uploaded to preprint servers, we found that only a fraction of this new output was cited in Wikipedia - less than 1 percent, or 27 (Figure 1C, Table S1) preprints referenced on Wikipedia. Among the preprints that were cited on Wikipedia was an early study on *Remdesivir* (12), a study on the mortality rate of elderly individuals (13), research on COVID-19 transmission in Spain (14) and New York (15), and research into how Wuhan's health system managed to eventually contain the virus (16), showing how non-peer-reviewed studies touched on medical, health and social aspects of the virus. The later was especially prevalent with two of the preprints focusing on the benefits of contact tracing (17, 18). The number of overall preprints was in line with the general representation of preprints in Wikipedia (1.5%), but lower than would be expected considering the fact that our academic database on EuroPMC had almost 3,700 preprints - 12.3 percent of the roughly 30,000 COVID-19 related papers in May 2020. Thus, in contrast to the high enrichment of preprints in COVID-19 research, Wikipedia's editors over-

whelmingly preferred peer-reviewed papers to preprints. In other words, Wikipedia generally cites preprints more than it was found to on the topic of COVID-19, while COVID-19 articles cited open-access paper by more 10 % (from 29 % to 39 %). Taken together with the bias towards high-impact journals, our data suggest that this contributed significantly to Wikipedia's ability to stay both up to date and to maintain high academic standards, allowing editors to cite peer-reviewed research despite other alternatives being available.

Examining the date of publication of the peer-reviewed studies referenced on Wikipedia (see Table S3) shows that new COVID-19 research was cited alongside papers from previous years and even the previous century, the oldest being a 1923 paper titled the "The Spread of Bacterial Infection. The Problem of Herd-Immunity."(19). Overall, among the papers referenced on Wikipedia were highly cited studies, some with thousands of citations, but most had low citation counts (median of citation count for a paper in the corpus was 5). Comparing between a paper's date of publication and its citation count reveals there is low anti-correlation (-0.2) but highly significant between the two (Pearson's product-moment correlation test p-value $< 10^{-15}$, Figure S3A). This suggest that on average older scientific papers have a higher citation count; unsurprisingly, the more time that has passed since publication, the bigger the chances a paper will being cited.

Due to the high-selectivity of Wikipedia editors in terms of citing academic research on COVID-19 articles, we also focused on non-academic sources: Popular media, we found, played a substantial role in our corpus: Over 80 percent of all the references used in the COVID-19 corpus were non-academic, being either general media or websites (Figure 2A). In fact, a mere 13 percent of the over 21,000 references supporting the COVID-19 content were from academic journals. Among the general media sources used (Figure 2B-D), there was a high representation for what is termed legacy media outlets, like the *New York Times* and the *BBC*, alongside widely syndicated news agencies like *Reuters* and the *Associated Press*, and official sources like *WHO.org* and *gov.UK*. Among the most cited websites, for example, there was an interesting representation of local media outlets from countries hit early and hard by the virus, with the Italian *La Repubblica* and the Chinese *South China Post* being among the most cited sites. The World Health Organization was one of the most cited publisher in the corpus of relevant articles, with 204 references. This can be attributed to the centralized nature of the Wikipedia community's response to the outbreak: In addition to concerted efforts by members of WikiProject Medicine - which strives for an especially rigorous sourcing policy on all biomedical articles, demanding only secondary sources from - a special COVID-19 "*Wiki project*" was set up at the beginning of March 2020 offering editors a list of "trusted" sources to use (20) - first and foremost was the WHO website.

Scientific Score. To distinguish between the role scientific research and popular media played, we created a "scientific score" for Wikipedia articles. The metric is based on the ratio of academic as opposed to non-academic references any article includes (Supplementary Figure S2). This score attempts to rank the *scientificness* of any given Wikipedia article based solely on its list of references. Ranging from 1 to 0, an article's scientific score is calculated according to the ratio of its sources that are academic (with DOIs), so that an article with a score of 1 will have 100 percent academic references, while that with none will have a score of zero. Technically, as all of our corpus of coronavirus-related Wikipedia articles had at least one academic source (DOI), their scientific scores will be greater than zero.

In effect, this score puts forth a metric for gauging the prominence of academic texts in any given article's reference list - or lack thereof. Out of our 231 Wikipedia articles, 15 received a perfect scientific score of 1 (Supplementary Figure S2A). High scientific score Wikipedia articles included the articles for the enzymes of "Furin" and "TMPRSS2" - whose inhibitor has been proposed as a possible treatment for COVID-19; "C30 Endopeptidase" - a group of enzymes also known as the "SARS coronavirus main proteinase"; and "SHC014-CoV" - a form of COVID-19 that affects the Chinese rufous horseshoe bat.

In contrast to the articles with scientific topics and even those for scientists, which had high scientific score, those with the lowest scores (Supplementary Figure S2B) seemed to focus almost exclusively on social aspects of the pandemic and its immediate outcome. For example, the articles with the lowest scores dealt directly with the pandemic in a hyper-local context, including articles about the pandemic in Canada, North America, Indonesia, Japan or even Jersey, to name a few. Others focused on different aspects of the pandemic, for example the "Impact of the COVID-19 pandemic on the arts and cultural heritage" or "Travel restrictions related to the COVID-19 pandemic". One of the articles with the lowest scientific score was the "Trump administration communication during the COVID-19 pandemic" which made scarce use of coronavirus-related research to inform its content, citing a single academic paper related to laws of quarantine among its 244 footnotes.

The Price of Remaining Up to Date on COVID-19. During the pandemic, there were over tens of thousands of edits to the site, with thousands of new articles being created and scores of existing ones being re-edited and recast in wake of new developments. Therefore, one could expect a rapid growth of articles on the topic, as well as a possible overall increase in the number of citations of all kinds. We sought to explore the temporal axis of Wikipedia's coverage of the pandemic to see how coverage of COVID-19 developed, namely, what were the dynamics of the growth of COVID-19 articles and their academic references.

First, we laid out our corpus of 231 articles across a timeline according to each article's respective date of creation. An article count starting from 2001, when Wikipedia was first launched, and up until May 2020, shows that for many years

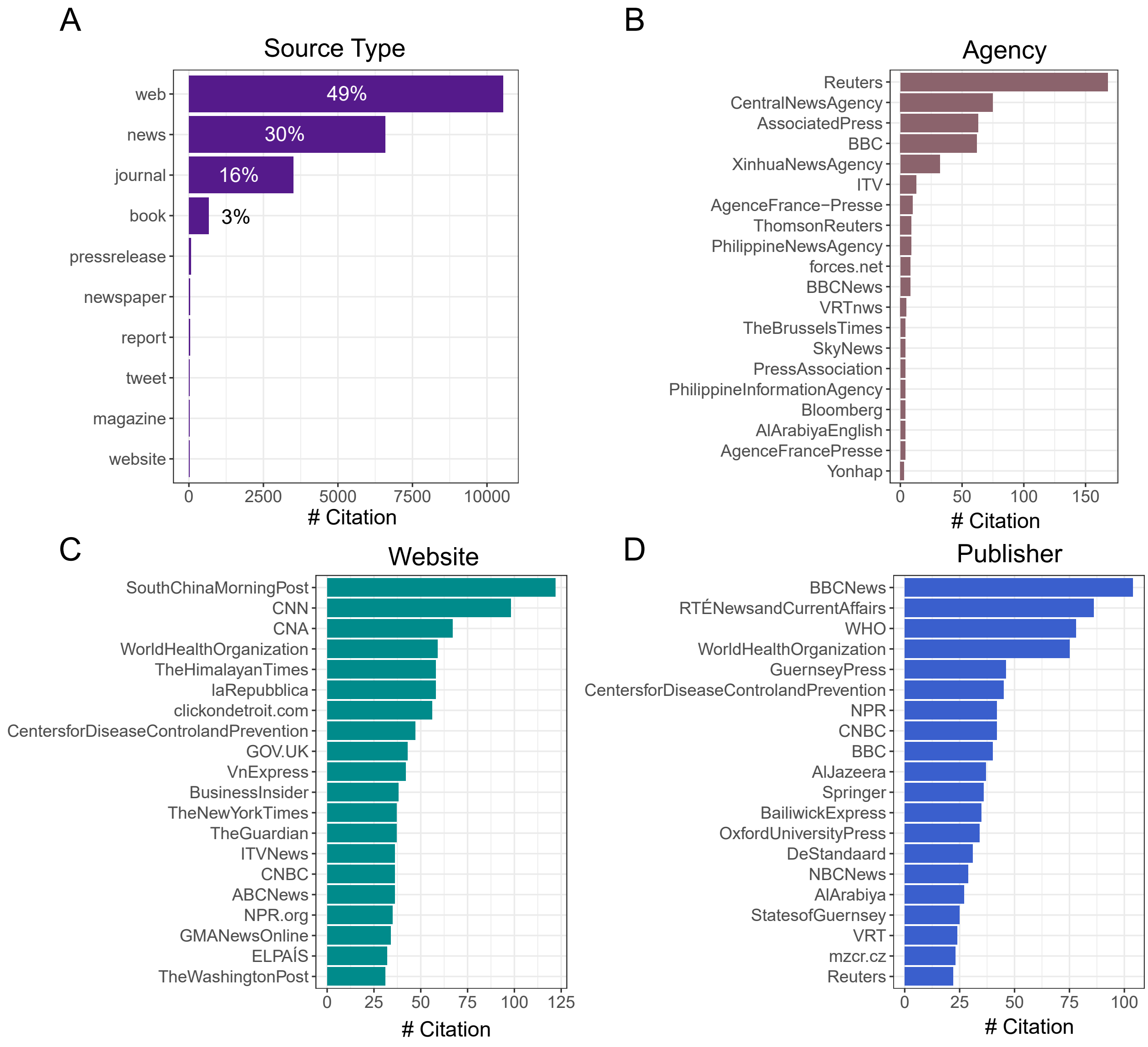

Figure 2. Wikipedia COVID-19 Corpus: Non-scientific sources mostly referred to websites or news media outlets considered highly respected and deemed to be trusted sources, including official sources like the WHO. A) source types extracted from the COVID-19 corpus of Wikipedia articles B) most cited news agencies, C) most cited websites and D) most cited publisher form the COVID-19 Corpus sources

there was a relatively steady growth in the number of articles that would become part of our corpus - until the pandemic hit, causing a massive peak at the start of 2020 (Figure 3A). As the pandemic spread, the total number of Wikipedia articles dealing with COVID-19 and supported by scientific literature almost doubled - with an near equal number of articles being created after 2020 than the entire time before (Figure 3A,B) (from 134 before 2020 compared to 97 in 2020).

The majority of the pre-2020 articles were created relatively early - between 2003 and 2006, likely linked to a general uptick in creation of articles on Wikipedia during this period. For example, the article for (the non-novel) “coronavirus” has existed since 2003, the article for the medical term “Transmission” and that of “Mathematical modeling of infectious diseases” from 2004, and the article for the “Coronaviridae” classification from 2005. Articles opened in this early period tended to focus on scientific concepts - for example those noted above or others like "Herd immunity". Conversely, the articles created post-pandemic during 2020 tended to be hyper-local or hyper-focused on the virus’ effects. Therefore, we collectively term the first group Wikipedia’s scientific infrastructure, as they allowed new information related to science to be added into existing articles, alongside the creation of new ones focusing on the pandemic’s actual ramifications.

Articles like “Chloroquine”, which has been examined as a possible treatment for COVID-19, underwent a shift in content in wake of the pandemic, seeing both a surge in traffic and a surge in editorial activity (Supplementary Figure S4). However, per a subjective reading of its content and the editing it underwent during this period, much of their scientific

content that was present pre-pandemic remained intact, with new coronavirus-related information being integrated into the existing content. The same occurred with many social concepts retroactively affiliated with COVID-19. Among these we can note the articles for “Herd immunity”, “Social distancing” and the “SARS conspiracy theory” that also existed prior to the outbreak and served as part of Wikipedia’s scientific infrastructure, allowing new information to be contextualized.

In addition to the dramatic rise in article creations during the pandemic, there was also a rise in those overall number of references affiliated with COVID-19 articles on Wikipedia (Figure 3C). In fact, the number of overall references in our articles grew almost six-fold post-2020 - from roughly 250 to almost 1,500 citations. Though the citations added were not just academic ones, with URLs overshadowing DOIs as the leading type of citation added, the general rise in citations can be seen as indicative of scientific literature’s prominent role in COVID-19 when taking into account that general trend in Wikipedia: The growth rate of references on COVID-19 articles was generally static until the outbreak; but on Wikipedia writ large references were on a rise since 2006. The post-2020 surge in citations was both academic and non-academic (Supplementary Figure S3B).

One could hypothesize that a rapid growth in the number articles dedicated to coronavirus would translate to an overall decrease in the presence of academic sources, as Wikipedia can create newer articles faster than academic research can be published on current events. Comparing the pre- and post-2020 articles’ scientific score reveals that on average, the new articles had a mean score of 0.14, compared to the pre-2020 group’s mean of 0.48 and the overall average of 0.3. Reading the titles of the 2020 articles to glean their topic and reviewing their respective scientific score can also point to a generalization: the more scientific an article is in topic, the more scientific its references are - even during the pandemic. This means that despite the dilution at a general level during the first month of 2020, articles with scientific topics that were created during this period did not pay that heavy of an academic price to stay up to date.

How is that Wikipedia managed to maintain academic sourcing on new and old articles about coronavirus as the pandemic was happening? One possible explanation is that among the academic papers added to Wikipedia in 2020 were also papers published prior to this year (Supplementary Figure S3A). We found the mean latency of Wikipedia’s COVID-19 content to be 10.2 years (Figure 3D), slower than Wikipedia’s overall mean of 8.7 (Figure 3E). In fact, in the coronavirus corpus we observed a peak in latency of 17 years - with over 500 citations being added to Wikipedia 17 years after their initial academic publication - almost twice as slow as Wikipedia’s average. Interestingly, this time frame corresponds to the SARS pandemic in 2003, which yielded a boost of scientific literature regarding the virus. This suggests that while there was a surge in editing activity during this pandemic that saw papers published in 2020 added to the COVID-19 articles, a large and even prominent role was still permitted for older literature. Viewed in this light, older papers played a similar role to pre-pandemic articles, giving precedence to existing knowledge in ordering the integration new knowledge on scientific topics.

Comparing the articles’ scientific score to their date of creation portrays Wikipedia’s scientific infrastructure and its dynamics during the pandemic (Supplementary Figure S3C). It reveals that despite maintaining high academic standards, citing papers published in prestigious and high-impact-factor journals, the need to stay up to date with COVID-19 research did come at some cost: most of the highest scoring articles were ones created pre-pandemic (mostly during 2005-2010) and newer articles had a lower scientific score (Supplementary Figure S3C). Since it is edited in real time, new articles based on popular sources could be created to respond to the pandemic as it happen. Indeed the majority of articles which were opened in 2020, those hyper-focused and hyper-localized articles detailing the pandemics effects, appeared to depend more on general media (Supplementary Figure S2A vs Supplementary Figure S2B). On the other hand, "Wikipedia is not a newspaper" (21) and does maintain a rigid sourcing policy, due to its encyclopedic nature and its reliance on academic sources for medical and health content. This seems to have been maintained though the overall ratio of academic versus non-academic literature shifted as the number of articles grew, indicating a decrease in scientificness over time.

Networks of COVID-19 Knowledge. To further investigate Wikipedia’s scientific sources and its infrastructure, we built a network of Wikipedia articles linked together based on their shared academic (DOI) sources. We filtered the list of papers (extracted DOIs) in order to keep those which were cited in at least two different Wikipedia articles, and found 179 that fulfilled this criteria, mapped to 136 Wikipedia articles in 454 different links (Figure 4, supplementary data (2)). This allowed us to map how scientific knowledge related to COVID-19 played a role not just in specific articles created during or prior to the pandemic, but actually formed a web of knowledge that proved to be an integral part of Wikipedia’s scientific infrastructure. Similar to the timeline described earlier, Wikipedia articles belonging to this network included those dealing with people, institutions, regional outcomes of the pandemic as well as scientific concepts, for example those regarding the molecular structure of the virus or the mechanism of infection ("C30 Endopeptidase", "Coronaviridae", and "Airborne disease"). It also included a number of articles regarding the search for a potential drug to combat the virus or other possible interventions against it (articles on topics like social distancing, vaccine development and drugs in current clinical trials).

Interestingly, we observed six prominent Wikipedia articles emerge in this network. These shared multiple citations with many other pages through DOI connections (nodes with an elevated degree). Four of these six so-called major nodes had a distinct and broad topic: “Coronavirus,” which focused on the virus writ large; “Coronavirus disease 2019”, which focused on the pandemic; and “COVID-19 drug repurposing

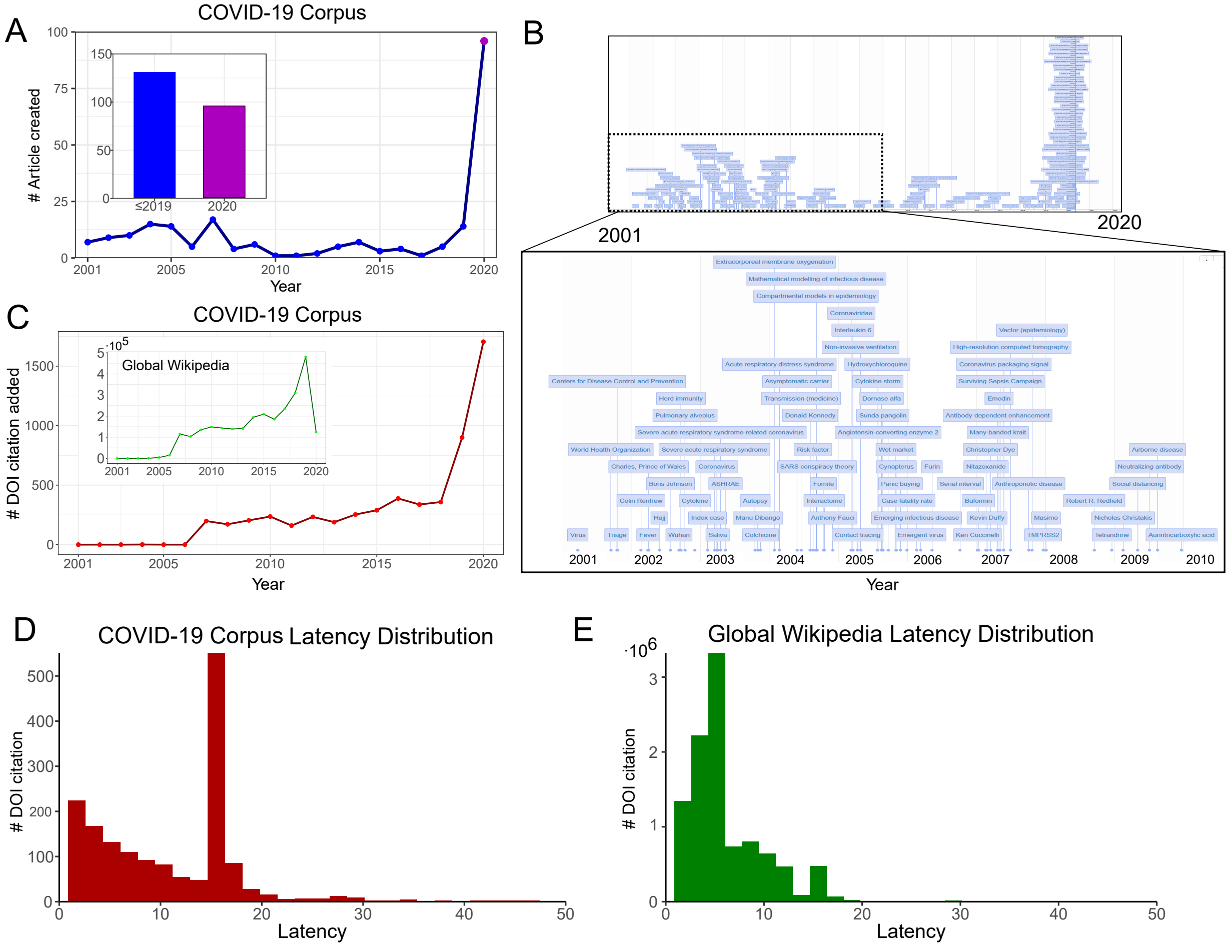

Figure 3. Historical perspective of the Wikipedia COVID-19 corpus outlining the growth of COVID-19 on the encyclopedia. A) COVID-19 article creation per year and number of articles created before the pandemic compared to the first five months of the pandemic. B) Timeline of the creation date of COVID-19 Wikipedia articles. C) Scientific citation added per year in the COVID-19 category and globally in Wikipedia. D) Latency distribution of scientific literature in the COVID-19 corpus and E) latency distribution of scientific literature in the Wikipedia dump. See here for an interactive version of the timeline.

research" and "COVID-19 drug development." The first two articles were key players in Wikipedia's coverage of the pandemic: both were linked to from the main coronavirus article ("COVID-19 pandemic") which was placed on the English Wikipedia's homepage, and later on, in a special banner located on the top of every single article in English, driving millions to the article and subsequent ones like those in our network.

The two remaining nodes were similar and did not prove to be distinctly independent concepts, but rather interrelated ones, with the articles for "Severe acute respiratory syndrome–related coronavirus" and "Severe acute respiratory syndrome coronavirus" each appearing as their own node despite their thematic connection. It is also interesting to note that four of the six Wikipedia articles that served as the respective centers of these groups of nodes were locked to public editing as part of the protected page status (see supplementary data (3)) and these were all articles linked to the WikiProject Medicine or, at a later stage, to the specific offshoot project set up to deal with COVID-19.

Two main themes that emerge from the network is that of COVID-19 related drugs and of the disease itself (Figure 4). Unlike popular articles relating to the effect of the virus, which we have seen are predominantly based on popular media, with scientific media playing a relatively small role, these two are topics that require scientific basing to be able to be reliable. The prominence of articles like "Coronavirus disease 2019" or "COVID-19 drug development" - both of which were locked (supplementary data (3)) and fell under the auspices of the COVID-19 task force - in our network underscore the role academic media had in their references. Furthermore, it highlights the effects of the editing community's centralized efforts: for example, by allowing key studies to find a role both in popular articles reached from the main articles, but also to subsequent articles about more focused scientific topics that could be reached from these. These centralized efforts created a filtered knowledge funnel of sorts, which harnessed Wikipedia' preexisting infrastructure to allow a regu-

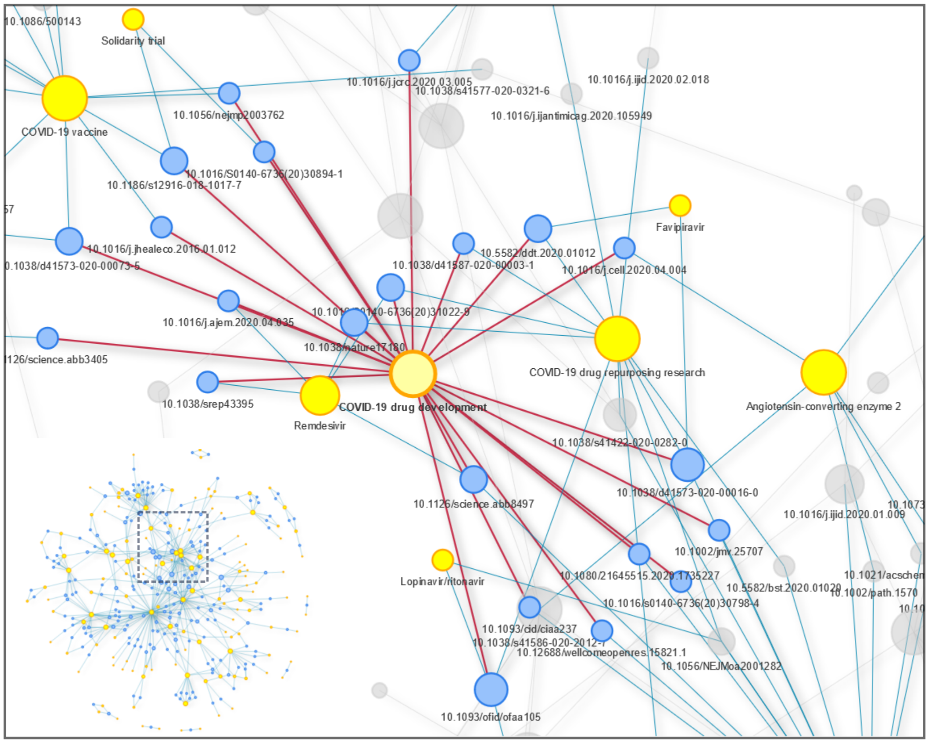

Figure 4. Wikipedia COVID-19 corpus article-scientific papers (DOI) network. The network mapping scientific papers cited in more than one article in the Wikipedia COVID-19 corpus was constructed using each DOI connecting at least two Wikipedia articles. This network is composed of 454 edges, 179 DOIs (Blue) and 136 Wikipedia articles (Yellow). A zoom in on the cluster of Wikipedia articles dealing with COVID-19 drug development is depicted with edges in red connecting the DOIs cited directly in the article and edges in blue connecting these DOIs to closely related articles citing the same DOIs. See here for an interactive version of the network.

lated intake of new information as well as the creation of new articles, both based on existing research.

In our network analysis, an additional smaller group of nodes (with a lower degree) had to do almost exclusively for China-related issues and exemplified how Wikipedia's sourcing policy - which has an explicit bias towards peer-reviewed studies and is enforced exclusively by the community - helps fight disinformation. For example, the academic paper that was most cited in Wikipedia's COVID-19 articles was a paper published in Nature in 2020, titled "A pneumonia outbreak associated with a new coronavirus of probable bat origin" (Table S2). This paper was referenced in eight different Wikipedia articles,among which two dealing directly with scientific topics - "Angiotensin-converting enzyme 2" and "Severe acute respiratory syndrome coronavirus 2" - and two dealing with what can be termed para-scientific terms linked to COVID-19 - the "Wuhan Institute of Virology" and "Shi Zhengli". This serves to highlight how contentious issues with a wide interest for the public - in this case, the origin of the virus - receive increased scientific support on Wikipedia, perhaps as result of editors attempting to fend off misinformation supported by lesser, non-academic sources - specifically media sources from China itself which as we have seen were present on Wikipedia. Of the five most cited papers (see Table S2) three focused specifically on either bats or the virus' animal origins, and another focusing on the spread from Wuhan, China. Interestingly, one of the 27 preprints cited in Wikipedia also included the first paper to suggest the virus' origin lay with bats was (22).

All in all, our findings reveal a trade off between quality and academic representativity in regards to scientific literature: most of Wikipedia's COVID-19 content was supported by highly trusted sources. Moreover, our analysis reveals that much of the scientific content on Wikipedia related to COVID-19 stands on the shoulders of what we termed "scientific infrastructure" - preexisting content in the form of either Wikipedia articles and existing academic research - that serves as the foundation for supporting the information deluge that followed the pandemic's outbreak.

Discussion

In the wake of COVID-19 pandemic, characterizing scientific research on Wikipedia and understanding the role it plays is both important and timely. Millions of people - both medical professionals and the general public - read about health online (1). Research has shown traffic to Wikipedia articles following those covered in the news (23). During a pandemic, as was during the Zika and SARS outbreaks (24), the risk of disinformation on Wikipedia's content is more severe. Thus, throughout the outbreak of the COVID-19 pandemic, the threat was hypothetically increased: as a surge in traffic to Wikipedia articles, research has found, often translates into an increase in vandalism (25). Moreover, research into medical content on Wikipedia found that people who read health articles on the open encyclopedia are more likely to hover over, or even read its references to learn more about the topic (26). Particularly in the case of the coronavirus outbreak, Wikipedia's role as such took on potentially lethal con-

sequences as the pandemic was deemed to be an *infodemic*, and false information related to the virus was deemed a real threat to public health by the UN and WHO (6). So far, most research into Wikipedia has revolved either around the quality, readership or editorship of health content on Wikipedia - or about references and sourcing in general. Meanwhile, research on Wikipedia and COVID-19 has focused almost exclusively on editing patterns and users behaviors, with a single study about research and coronavirus (9) focusing solely on the representativity of academic citations. Therefore, we set out to examine in a temporal, qualitative and quantitative manner, the role of references in articles linked directly to the pandemic as it broke.

Perhaps counter-intuitively, an analysis of our Wikipedia articles and their sources during and at the end of the first wave found that despite the traffic surge, Wikipedia's articles on COVID-19 relied on high quality sources from both popular and academic media. Though academic references did decrease in comparison to the period before the pandemic (i.e. lower scientific score), we found that academic sources still played a prominent role and that high editorial standards were generally maintained, utilizing several unique solutions which we will now attempt to draw and outline from our findings.

One possible key to Wikipedia's success has to do with the existence of centralized oversight mechanisms by the community of editors that could be quickly and efficiently deployed. In this case, the existence of the WikiProject Medicine, and the formation of a specific COVID-19 task force in the form of WikiProject COVID-19, helped safeguard quality across large swaths of articles and enforce a relatively unified sourcing policy on articles dealing with both popular and scientific aspects of the virus. All factual claims on Wikipedia need to be supported by a verifiable source. Biomedical articles affiliated with the WikiProject Medicine (WPM) are bound by a specific policy known as MEDRS (short hand for medical reliable source) which ban primary sources and demands meta-analysis or secondary sources that provide an overview. "Examples include literature reviews or systematic reviews found in medical journals, specialist academic or professional books, and medical guidelines or position statements published by major health organizations"(27). One mechanism used generally by the WikiProject Medicine to enforce the MEDRS sourcing standards and specifically deployed by the COVID-19 task force during the pandemic was locking articles to public editing (protected pages). This is a technique that is used to fight vandalism on Wikipedia (28) and is commonly used when news events drive readers to specific Wikipedia articles, increasing the risk of substandard sources being added into the article. The ad hoc measure of locking an article, deployed by a community vote on specific articles for specific amounts of time, prevents anonymous editors from being able to contribute directly to an article's text and forces them to work through an experienced editor, thus exposing both their potential vandalism and poorly sourced misinformation to editorial scrutiny. This measure is in line with our findings that many of the COVID-19 network central nodes were locked articles (Supp. data (3)).

Another possible key to Wikipedia's ability to maintain the WPM's MEDRS policy of high quality sources during the pandemic through centralized community mechanisms was the task force's list of "trusted" sources. The WHO, for example, was given special status and preference (20). This was evident in our results as the WHO was among the most cited publishers on the COVID-19 articles. Also among our most cited scientific sources were others promoted by the task force as preferable for sourcing scientific content: for example, *Science*, *Nature* and *The Lancet*. This indicates that sources recommended by the task force were actually used by volunteers and thus underscores the connection between our findings and the existence of a centralized effort by volunteers. Among general media sources that the task force endorsed were *Reuters* and the *New York Times* which were also represented prominently in our findings. As each new edit to any locked COVID-19 article needed to be vetted by the task force's volunteers before it could go online within the body of an article's text, thus slowing down the influx of information, the source list allowed an especially strict sourcing policy to be rigorously implemented across thousands of articles. This was true despite the fact that there is no academic verification for volunteers - in fact research suggests that less than half of the Wikipedia editors focused on health and medical issues are medical professionals (3, 4) - meaning that the task forces and its list of sources allowed non-experts to enforce academic-level standards. Even within scientific content, despite a deluge of preprints (both in general in recent years and specifically during the pandemic (11, 29)), in our analysis, non-peer-reviewed academic sources did not play a key role on Wikipedia's coronavirus content. In its stead, one could speculate that our finding that open-access papers were disproportionately highly cited may provide an explanation. Previous research has found open-access papers are more likely to be cited on Wikipedia by 47 percent (7) and nearly one-third of the Wikipedia citations link to an open-access source (30). Here we also saw that open-access was prevalent in Wikipedia and even more so on COVID-19 articles. This, we suggest, allowed Wikipedia's editors (expert or otherwise) to keep articles up to date without reverting to non-peer-reviewed academic content. This, one could suggest, was likely facilitated by the decision by academic publications' like *Nature* and *Science* to lift paywall and open public access to all of their COVID-19-related research papers, both past and present.

In addition to the communal infrastructure's ability to regulate the addition of new information and maintain quality standards over time, another facet we found to contribute in permitting Wikipedia to stay accurate during the pandemic is what we term its scientific infrastructure. Research on Wikipedia article's content has shown that the initial structuring of information on any given article tends to dictate its development in later stages, and that substantial reorganizations gradually decrease over time (31). A temporal review of our articles and their citations, showed that the best-sourced articles, the scientific backbone of Wikipedia's COVID-19

content, were those created from 2005 and until 2010. These, we argue, are part of Wikipedia's wider scientific infrastructure, which supported the intake of new knowledge into Wikipedia.

This could also be seen to be true about older academic papers. While the average research paper takes roughly 6-12 months to get published (32), in our analysis, Wikipedia has roughly a 8.7 year latency in citing articles. This means that while there was a surge in public interest and editing to Wikipedia about the COVID-19 pandemic, the mainstream output of scientific work on the virus predated the pandemic's outbreak to a great extent.

As our scientific score analysis shows, scientific articles that existed prior to the pandemic suffered only a minor decrees in scientificness during the first wave, despite the dilution at a general level during the first month of 2020. Scientific content stayed generally scientific during the pandemic and new content created during this period, though generally less scientific, still preferred referring to quality sources, perhaps due to oversight by the task force and Wikipedia's communal infrastructure as well as the opening of access to COVID-19 scientific papers. Our network shows the pivotal role preexisting content played in contextualizing the science behind many popular concepts or those made popular by the pandemic.

Preexisting content in the form of either Wikipedia articles and academic research served as a framework that helped regulate the deluge of new information, allowing newer findings to find a place within Wikipedia's existing network of knowledge. Future work on this topic could focus on the question of whether this dynamic changed as 2020 progressed and on how contemporary peer-reviewed COVID-19-related research that was published during the pandemic will be integrated into these articles in the near future.

Our findings outline ways in which Wikipedia managed to fight off disinformation and stay up to date. With Facebook and other social media giants struggling to implement both technical and human-driven solutions to disinformation from the top down, it seems Wikipedia dual usage of established science and a community of volunteers, provides a possible model for how this can be achieved - a valuable task during an infodemic. In October 2020, the WHO and Wikimedia, the foundation that oversees the Wikipedia project, announced they would cooperate to make critical public health information available. This means that in the near future, the quality of Wikipedia's coverage of the pandemic will very likely increase just as its role as central node in the network of knowledge transference to the general public becomes increasingly clear.

Wikipedia's main advantage is in many ways its largest disadvantage: its open format which allows a large community of editors of varying degrees of expertise to contribute. This can lead to large discrepancies in articles quality and inconsistencies in the way editors add references to articles' text (30). We tried to address these limitations using technical solutions, such as regular expressions for extracting URLs, hyprelinks, DOIs and PMIDs. In this study, we retrieved most of our scientific literature metadata using Altmetrics (33, 34), EuroPMC (35) and CrossRef (36) R APIs. However, this method was not without limitations and we could not, for example, retrieve all of the extracted DOIs metadata. Moreover, information regarding open access (among others) varied with quality between the APIs (37). In addition, our preprint analysis was mainly focused on MedRxiv and BioRxiv which have the benefit of having a distinct DOI prefix. Unfortunately, no better solution could be found to annotate preprints from the extracted DOIs. Preprint servers do not necessarily use the DOI system (38) (i.e ArXiv) and others share DOI prefixes with published paper (for instance the preprint server used by The Lancet). Moreover, we developed a parser for general citations (news outlets, websites, publishers), and we could not properly clean redundant entries (i.e "WHO", "World Health Organisation"). Finally, as Wikipedia is constantly changing, some of our conclusions are bound to change. Therefore, our study is focused on the pandemic's first wave and its history, crucial to examine the dynamics of knowledge online at a pivotal timeframe.

In summary, our findings reveal a trade off between timeliness and scientificness in regards to scientific literature: most of Wikipedia's COVID-19 content was supported by references from highly trusted sources - but more from the general media than from academic publications. That Wikipedia's COVID-19 articles were based on respected sources in both the academic and popular media was found to be true even as the pandemic and number of articles about it grew. Our investigation further demonstrates that despite a surge in preprints about the virus and their promise of cutting-edge information, Wikipedia preferred published studies, giving a clear preference to open-access studies. A temporal and network analysis of COVID-19 articles indicated that remaining up-to-date did come at a cost in terms of quality, but also showed how preexisting content helped regulate the flow of new information into existing articles. In future work, we hope the tools and methods developed here in regards to the first wave of the pandemic will be used to examine how these same articles fared over the entire span of 2020, as well as helping others use them for research into other topics on Wikipedia. We observed how Wikipedia used volunteers editors to enforce a rigid sourcing standards - and future work may continue to provide insight into how this unique method can be used to fight disinformation and to characterize the knowledge infrastructure in other arenas.

Acknowledgments

J.S. is a recipient of the Placide Nicod foundation, and R.A. is a recipient of the Azrieli Foundation fellowship. We are grateful for their financial support.

1. James M Heilman and Andrew G West. Wikipedia and medicine: quantifying readership, editors, and the significance of natural language. *Journal of medical Internet research*, 17 (3):e62, 2015.
2. Stacey M Lavsa, Shelby L Corman, Colleen M Culley, and Tara L Pummer. Reliability of wikipedia as a medication information source for pharmacy students. *Currents in Pharmacy Teaching and Learning*, 3(2):154–158, 2011.

3. Usaid K Allahwala, Aniket Nadkarni, and Deshan F Sebaratnam. Wikipedia use amongst medical students–new insights into the digital revolution. *Medical teacher*, 35(4):337–337, 2013.
4. James M Heilman, Eckhard Kemmann, Michael Bonert, Anwesh Chatterjee, Brent Ragar, Graham M Beards, David J Iberri, Matthew Harvey, Brendan Thomas, Wouter Stomp, et al. Wikipedia: a key tool for global public health promotion. *Journal of medical Internet research*, 13(1):e14, 2011.
5. Verena G Herbert, Andreas Frings, Herwig Rehatschek, Gisbert Richard, and Andreas Leithner. Wikipedia–challenges and new horizons in enhancing medical education. *BMC medical education*, 15(1):32, 2015.
6. WHO. Novel coronavirus (2019-ncov): situation report, 13, 2020.
7. Misha Teplitskiy, Grace Lu, and Eamon Duede. Amplifying the impact of open access: Wikipedia and the diffusion of science. *Journal of the Association for Information Science and Technology*, 68(9):2116–2127, 2017.
8. Omer Benjakob and Rona Aviram. A clockwork wikipedia: From a broad perspective to a case study. *Journal of Biological Rhythms*, 33(3):233–244, 2018.
9. Giovanni Colavizza. Covid-19 research in wikipedia. *Quantitative Science Studies*, pages 1–32, 2020.
10. Wikipedia. Wikipedia:core content policies. https://en.wikipedia.org/wiki/Wikipedia:Core_content_policies, 2020.
11. Nicholas Fraser, Liam Brierley, Gautam Dey, Jessica K Polka, Máté Pálfy, and Jonathon Alexis Coates. Preprinting a pandemic: the role of preprints in the covid-19 pandemic. *bioRxiv*, 2020.
12. Brandi N Williamson, Friederike Feldmann, Benjamin Schwarz, Kimberly Meade-White, Danielle P Porter, Jonathan Schulz, Neeltje Van Doremalen, Ian Leighton, Claude Kwe Yinda, Lizzette Pérez-Pérez, et al. Clinical benefit of remdesivir in rhesus macaques infected with sars-cov-2. *BioRxiv*, 2020.
13. John PA Ioannidis, Cathrine Axfors, and Despina G Contopoulos-Ioannidis. Population-level covid-19 mortality risk for non-elderly individuals overall and for non-elderly individuals without underlying diseases in pandemic epicenters. *medRxiv*, 2020.
14. Francisco Díez Fuertes, María Iglesias Caballero, Sara Monzón, Pilar Jiménez, Sarai Varona, Isabel Cuesta, Ángel Zaballos, Michael M Thomson, Mercedes Jiménez, Javier García Pérez, et al. Phylodynamics of sars-cov-2 transmission in spain. *bioRxiv*, 2020.
15. Ana S Gonzalez-Reiche, Matthew M Hernandez, Mitchell J Sullivan, Brianne Ciferri, Hala Alshammary, Ajay Obla, Shelcie Fabre, Giulio Kleiner, Jose Polanco, Zenab Khan, et al. Introductions and early spread of sars-cov-2 in the new york city area. *Science*, 2020.
16. WK Ming, J Huang, and CJP Zhang. Breaking down of the healthcare system: Mathematical modelling for controlling the novel coronavirus (2019-ncov) outbreak in wuhan, chinadoi: 10.1101/2020.01. 27.922443. *URL https://doi. org/10.1101% 2F2020*, 1:922443, 2020.
17. Justin D Silverman, Nathaniel Hupert, and Alex D Washburne. Using ili surveillance to estimate state-specific case detection rates and forecast sars-cov-2 spread in the united states. *medRxiv*, 2020.
18. M Kendall, M Parker, C Fraser, A Nurtay, C Wymant, D Bonsall, L Zhao, L Ferretti, and L Abeler-Dörner. Quantifying sars-cov-2 transmission suggests epidemic control with digital contact tracing. *Science*, 2020.
19. WWC Topley and GS Wilson. The spread of bacterial infection. the problem of herd-immunity. *Epidemiology & Infection*, 21(3):243–249, 1923.
20. Wikipedia. Wikipedia project covid-19: Reference sources. https://en.wikipedia.org/wiki/Wikipedia:WikiProject_COVID-19/Reference_sources, 2021.
21. Wikipedia. Wikipedia:wikipedia is not a newspaper. https://en.wikipedia.org/wiki/Wikipedia:Wikipedia_is_not_a_newspaper, 2021.
22. Peng Zhou, Xing-Lou Yang, Xian-Guang Wang, Ben Hu, Lei Zhang, Wei Zhang, Hao-Rui Si, Yan Zhu, Bei Li, Chao-Lin Huang, et al. Discovery of a novel coronavirus associated with the recent pneumonia outbreak in humans and its potential bat origin. *BioRxiv*, 2020.
23. Brian Keegan, Darren Gergle, and Noshir Contractor. Hot off the wiki: Structures and dynamics of wikipedia's coverage of breaking news events. *American Behavioral Scientist*, 57(5):595–622, 2013.
24. Nicholas Generous, Geoffrey Fairchild, Alina Deshpande, Sara Y Del Valle, and Reid Priedhorsky. Global disease monitoring and forecasting with wikipedia. *PLoS Comput Biol*, 10 (11):e1003892, 2014.
25. Qinyi Wu, Danesh Irani, Calton Pu, and Lakshmish Ramaswamy. Elusive vandalism detection in wikipedia: A text stability-based approach. In *Proceedings of the 19th ACM International Conference on Information and Knowledge Management*, CIKM '10, page 1797–1800, New York, NY, USA, 2010. Association for Computing Machinery. ISBN 9781450300995. doi: 10.1145/1871437.1871732.
26. Lauren A Maggio, Ryan M Steinberg, Tiziano Piccardi, and John M Willinsky. Meta-research: Reader engagement with medical content on wikipedia. *Elife*, 9:e52426, 2020.
27. Wikipedia. Wikipedia:identifying reliable sources (medicine). https://en.wikipedia.org/wiki/Wikipedia:Identifying_reliable_sources_(medicine), 2021.
28. Taha Yasseri, Robert Sumi, András Rung, András Kornai, and János Kertész. Dynamics of conflicts in wikipedia. *PloS one*, 7(6):e38869, 2012.
29. Darwin Y Fu and Jacob J Hughey. Meta-research: Releasing a preprint is associated with more attention and citations for the peer-reviewed article. *Elife*, 8:e52646, 2019.
30. Aida Pooladian and Ángel Borrego. Methodological issues in measuring citations in wikipedia: a case study in library and information science. *Scientometrics*, 113(1):455–464, 2017.
31. Amit Arjun Verma and S. Iyengar. Tracing the factoids: the anatomy of information re-organization in wikipedia articles. 2021.
32. Kendall Powell. Does it take too long to publish research? *Nature News*, 530(7589):148, 2016.
33. Roberta Kwok. Research impact: Altmetrics make their mark. *Nature*, 500(7463):491–493, 2013.
34. Karthik Ram. *rAltmetric: Retrieves altmerics data for any published paper from altmetrics.com*, 2012. R package version 0.3.
35. Maria Levchenko, Yuci Gou, Florian Graef, Audrey Hamelers, Zhan Huang, Michele Ide-Smith, Anusha Iyer, Oliver Kilian, Jyothi Katuri, Jee-Hyub Kim, et al. Europe pmc in 2017. *Nucleic acids research*, 46(D1):D1254–D1260, 2018.
36. Rachael Lammey. Using the crossref metadata api to explore publisher content. *Sci Ed*, 3 (3):109–11, 2016.
37. Christine Meschede and Tobias Siebenlist. Cross-metric compatability and inconsistencies of altmetrics. *Scientometrics*, 115(1):283–297, 2018.
38. Norman Paskin. Digital object identifier (doi®) system. *Encyclopedia of library and information sciences*, 3:1586–1592, 2010.

Supplementary information

Table 1. Preprints cited within the Wikipedia COVID-19 Corpus

title	doi	authorString	pubYear
Isolation and Characterization of 2019-nCoV-like Coronavirus from Malayan Pangolins	10.1101/2020.02.17.951335	Xiao K, Zhai J, Feng Y, Zhou N, Zhang X, Zou J, Li N, Guo Y, Li X, Shen X, Zhang Z, Shu F, Huang W, Li Y, Zhang Z, Chen R, Wu Y, Peng S, Huang M, Xie W, Cai Q, Hou F, Liu Y, Chen W, Xiao L, Shen Y.	2020
Evidence of recombination in coronaviruses implicating pangolin origins of nCoV-2019	10.1101/2020.02.07.939207	Wong MC, Javornik Cregeen SJ, Ajami NJ, Petrosino JF.	2020
Spike mutation pipeline reveals the emergence of a more transmissible form of SARS-CoV-2	10.1101/2020.04.29.069054	Korber B, Fischer W, Gnanakaran S, Yoon H, Theiler J, Abfalterer W, Foley B, Giorgi E, Bhattacharya T, Parker M, Partridge D, Evans C, Freeman T, de Silva T, LaBranche C, Montefiori D, on behalf of the Sheffield COVID-19 Genomics Group.	2020
Global profiling of SARS-CoV-2 specific IgG/ IgM responses of convalescents using a proteome microarray	10.1101/2020.03.20.20039495	Jiang H, Li Y, Zhang H, Wang W, Men D, Yang X, Qi H, Zhou J, Tao S.	2020
Novel coronavirus 2019-nCoV: early estimation of epidemiological parameters and epidemic predictions	10.1101/2020.01.23.20018549	Read JM, Bridgen JR, Cummings DA, Ho A, Jewell CP.	2020
Aerodynamic Characteristics and RNA Concentration of SARS-CoV-2 Aerosol in Wuhan Hospitals during COVID-19 Outbreak	10.1101/2020.03.08.982637	Liu Y, Ning Z, Chen Y, Guo M, Liu Y, Gali NK, Sun L, Duan Y, Cai J, Westerdahl D, Liu X, Ho K, Kan H, Fu Q, Lan K.	2020
Correlation Analysis Between Disease Severity and Inflammation-related Parameters in Patients with COVID-19 Pneumonia	10.1101/2020.02.25.20025643	Gong J, Dong H, Xia SQ, Huang YZ, Wang D, Zhao Y, Liu W, Tu S, Zhang M, Wang Q, Lu F.	2020
Estimation of COVID-2019 burden and potential for international dissemination of infection from Iran	10.1101/2020.02.24.20027375	Tuite AR, Bogoch I, Sherbo R, Watts A, Fisman DN, Khan K.	2020
Explaining national differences in the mortality of COVID-19: individual patient simulation model to investigate the effects of testing policy and other factors on apparent mortality.	10.1101/2020.04.02.20050633	Michaels JA, Stevenson MD.	2020
Saliva is more sensitive for SARS-CoV-2 detection in COVID-19 patients than nasopharyngeal swabs	10.1101/2020.04.16.20067835	Wyllie AL, Fournier J, Casanovas-Massana A, Campbell M, Tokuyama M, Vijayakumar P, Geng B, Muenker MC, Moore AJ, Vogels CBF, Petrone ME, Ott IM, Lu P, Lu-Culligan A, Klein J, Venkataraman A, Earnest R, Simonov M, Datta R, Handoko R, Naushad N, Sewanan LR, Valdez J, White EB, Lapidus S, Kalinich CC, Jiang X, Kim DJ, Kudo E, Linehan M, Mao T, Moriyama M, Oh JE, Park A, Silva J, Song E, Takahashi T, Taura M, Weizman O, Wong P, Yang Y, Bermejo S, Odio C, Omer SB, Dela Cruz CS, Farhadian S, Martinello RA, Iwasaki A, Grubaugh ND, Ko AI.	2020
Neutralizing antibody responses to SARS-CoV-2 in a COVID-19 recovered patient cohort and their implications	10.1101/2020.03.30.20047365	Wu F, Wang A, Liu M, Wang Q, Chen J, Xia S, Ling Y, Zhang Y, Xun J, Lu L, Jiang S, Lu H, Wen Y, Huang J.	2020
Estimation of SARS-CoV-2 Infection Prevalence in Santa Clara County	10.1101/2020.03.24.20043067	Yadlowsky S, Shah N, Steinhardt J.	2020
Population-level COVID-19 mortality risk for non-elderly individuals overall and for non-elderly individuals without underlying diseases in pandemic epicenters	10.1101/2020.04.05.20054361	Ioannidis JPA, Axfors C, Contopoulos-Ioannidis DG.	2020
Respiratory disease and virus shedding in rhesus macaques inoculated with SARS-CoV-2	10.1101/2020.03.21.001628	Munster VJ, Feldmann F, Williamson BN, van Doremalen N, Pérez-Pérez L, Schulz J, Meade-White K, Okumura A, Callison J, Brumbaugh B, Avanzato VA, Rosenke R, Hanley PW, Saturday G, Scott D, Fischer ER, de Wit E.	2020
Clinical benefit of remdesivir in rhesus macaques infected with SARS-CoV-2	10.1101/2020.04.15.043166	Williamson BN, Feldmann F, Schwarz B, Meade-White K, Porter DP, Schulz J, Doremalen Nv, Leighton I, Yinda CK, Pérez-Pérez L, Okumura A, Lovaglio J, Hanley PW, Saturday G, Bosio CM, Anzick S, Barbian K, Cihlar T, Martens C, Scott DP, Munster VJ, Wit Ed.	2020

Discovery of a novel coronavirus associated with the recent pneumonia outbreak in humans and its potential bat origin	10.1101/2020.01.22.914952	Zhou P, Yang X, Wang X, Hu B, Zhang L, Zhang W, Si H, Zhu Y, Li B, Huang C, Chen H, Chen J, Luo Y, Guo H, Jiang R, Liu M, Chen Y, Shen X, Wang X, Zheng X, Zhao K, Chen Q, Deng F, Liu L, Yan B, Zhan F, Wang Y, Xiao G, Shi Z.	2020
Breaking down of the healthcare system: Mathematical modelling for controlling the novel coronavirus (2019-nCoV) outbreak in Wuhan, China	10.1101/2020.01.27.922443	Ming W, Huang J, Zhang CJP.	2020
Introductions and early spread of SARS-CoV-2 in the New York City area	10.1101/2020.04.08.20056929	Gonzalez-Reiche AS, Hernandez MM, Sullivan M, Ciferri B, Alshammary H, Obla A, Fabre S, Kleiner G, Polanco J, Khan Z, Alburquerque B, van de Guchte A, Dutta J, Francoeur N, Melo BS, Oussenko I, Deikus G, Soto J, Sridhar SH, Wang Y, Twyman K, Kasarskis A, Altman DR, Smith M, Sebra R, Aberg J, Krammer F, Garcia-Sarstre A, Luksza M, Patel G, Paniz-Mondolfi A, Gitman M, Sordillo EM, Simon V, van Bakel H.	2020
Phylodynamics of SARS-CoV-2 transmission in Spain	10.1101/2020.04.20.050039	Díez-Fuertes F, Iglesias-Caballero M, Monzón S, Jiménez P, Varona S, Cuesta I, Zaballos Á, Thomson MM, Jiménez M, García Pérez J, Pozo F, Pérez-Olmeda M, Alcamí J, Casas I.	2020
Using ILI surveillance to estimate state-specific case detection rates and forecast SARS-CoV-2 spread in the United States	10.1101/2020.04.01.20050542	Silverman JD, Hupert N, Washburne AD.	2020
Quantifying SARS-CoV-2 transmission suggests epidemic control with digital contact tracing	10.1101/2020.03.08.20032946	Ferretti L, Wymant C, Kendall M, Zhao L, Nurtay A, Abeler-Dorner L, Parker M, Bonsall DG, Fraser C.	2020
Adoption and impact of non-pharmaceutical interventions for COVID-19	10.12688/wellcomeopenres.15808.	Imai N, Gaythorpe KA, Abbott S, Bhatia S, van Elsland S, Prem K, Liu Y, Ferguson NM.	2020
Aberrant pathogenic GM-CSF+ T cells and inflammatory CD14+CD16+ monocytes in severe pulmonary syndrome patients of a new coronavirus	10.1101/2020.02.12.945576	Zhou Y, Fu B, Zheng X, Wang D, Zhao C, qi Y, Sun R, Tian Z, Xu X, Wei H.	2020
SARS-CoV-2 invades host cells via a novel route: CD147-spike protein	10.1101/2020.03.14.988345	Wang K, Chen W, Zhou Y, Lian J, Zhang Z, Du P, Gong L, Zhang Y, Cui H, Geng J, Wang B, Sun X, Wang C, Yang X, Lin P, Deng Y, Wei D, Yang X, Zhu Y, Zhang K, Zheng Z, Miao J, Guo T, Shi Y, Zhang J, Fu L, Wang Q, Bian H, Zhu P, Chen Z.	2020
Functional assessment of cell entry and receptor usage for lineage B ß-coronaviruses, including 2019-nCoV	10.1101/2020.01.22.915660	Letko M, Munster V.	2020
Broad anti-coronaviral activity of FDA approved drugs against SARS-CoV-2 in vitro and SARS-CoV in vivo	10.1101/2020.03.25.008482	Weston S, Coleman CM, Haupt R, Logue J, Matthews K, Frieman MB.	2020
Global and Temporal Patterns of Submicroscopic Plasmodium falciparum Malaria Infection	10.1101/554311	Whittaker C, Slater H, Bousema T, Drakeley C, Ghani A, Okell L.	2019

Table 2. Most cited scientific papers in COVID-19 Wikipedia corpus

doi	Authors	OA	Journal	Year	Source	Title	Wiki	Sci.lit
10.1038/s41586-020-2012-7	Zhou P, Yang XL, Wang XG, Hu B, Zhang L, Zhang W, Si HR, Zhu Y, Li B, Huang CL, Chen HD, Chen J, Luo Y, Guo H, Jiang RD, Liu MQ, Chen Y, Shen XR, Wang X, Zheng XS, Zhao K, Chen QJ, Deng F, Liu LL, Yan B, Zhan FX, Wang YY, Xiao GF, Shi ZL.	Y	Nature	2020	MED	A pneumonia outbreak associated with a new coronavirus of probable bat origin.	8	940
10.3390/v11020174	Wong ACP, Li X, Lau SKP, Woo PCY.	Y	Viruses	2019	MED	Global Epidemiology of Bat Coronaviruses.	6	28
10.1016/j.ijid.2020.01.009	Hui DS, I Azhar E, Madani TA, Ntoumi F, Kock R, Dar O, Ippolito G, Mchugh TD, Memish ZA, Drosten C, Zumla A, Petersen E.	Y	Int J Infect Dis	2020	MED	The continuing 2019-nCoV epidemic threat of novel coronaviruses to global health - The latest 2019 novel coronavirus outbreak in Wuhan, China.	5	228
10.1016/j.jmii.2020.03.013	Lau H, Khosrawipour V, Kocbach P, Mikolajczyk A, Ichii H, Schubert J, Bania J, Khosrawipour T.	Y	J Microbiol Immunol Infect	2020	MED	Internationally lost COVID-19 cases.	5	5
10.1038/d41586-020-00548-w	Cyranoski D.	N	Nature	2020	MED	Mystery deepens over animal source of coronavirus.	5	8
10.1038/s41591-020-0820-9	Andersen KG, Rambaut A, Lipkin WI, Holmes EC, Garry RF.	Y	Nat Med	2020	MED	The proximal origin of SARS-CoV-2.	5	147
10.3390/v2081803	Woo PC, Huang Y, Lau SK, Yuen KY.	Y	Viruses	2010	MED	Coronavirus genomics and bioinformatics analysis.	5	109
10.1007/978-1-4939-2438-7$_1$	Fehr AR, Perlman S.	Y	Methods Mol Biol	2015	MED	Coronaviruses: an overview of their replication and pathogenesis.	4	195
10.1007/s00134-020-05991-x	Ruan Q, Yang K, Wang W, Jiang L, Song J.	Y	Intensive Care Med	2020	MED	Clinical predictors of mortality due to COVID-19 based on an analysis of data of 150 patients from Wuhan, China.	4	66
10.1038/d41573-020-00016-0	Li G, De Clercq E.	N	Nat Rev Drug Discov	2020	MED	Therapeutic options for the 2019 novel coronavirus (2019-nCoV).	4	105
10.1038/s41422-020-0282-0	Wang M, Cao R, Zhang L, Yang X, Liu J, Xu M, Shi Z, Hu Z, Zhong W, Xiao G.	Y	Cell Res	2020	MED	Remdesivir and chloroquine effectively inhibit the recently emerged novel coronavirus (2019-nCoV) in vitro.	4	474
10.1093/cid/ciaa149	To KK, Tsang OT, Chik-Yan Yip C, Chan KH, Wu TC, Chan JMC, Leung WS, Chik TS, Choi CY, Kandamby DH, Lung DC, Tam AR, Poon RW, Fung AY, Hung IF, Cheng VC, Chan JF, Yuen KY.	Y	Clin Infect Dis	2020	MED	Consistent detection of 2019 novel coronavirus in saliva.	4	94
10.1093/ofid/ofaa105	McCreary EK, Pogue JM.	Y	Open Forum Infect Dis	2020	MED	Coronavirus Disease 2019 Treatment: A Review of Early and Emerging Options.	4	7
10.1126/science.aba9757	Chinazzi M, Davis JT, Ajelli M, Gioannini C, Litvinova M, Merler S, Pastore Y Piontti A, Mu K, Rossi L, Sun K, Viboud C, Xiong X, Yu H, Halloran ME, Longini IM, Vespignani A.	Y	Science	2020	MED	The effect of travel restrictions on the spread of the 2019 novel coronavirus (COVID-19) outbreak.	4	69
10.1093/jtm/taaa030	Rocklöv J, Sjödin H, Wilder-Smith A.	Y	J Travel Med	2020	MED	COVID-19 outbreak on the Diamond Princess cruise ship: estimating the epidemic potential and effectiveness of public health countermeasures.	3	25
10.1101/2020.02.07.939207	Wong MC, Javornik Cregeen SJ, Ajami NJ, Petrosino JF.	N	NA	2020	PPR	Evidence of recombination in coronaviruses implicating pangolin origins of nCoV-2019	3	15
10.1101/2020.02.17.951335	Xiao K, Zhai J, Feng Y, Zhou N, Zhang X, Zou J, Li N, Guo Y, Li X, Shen X, Zhang Z, Shu F, Huang W, Li Y, Zhang Z, Chen R, Wu Y, Peng S, Huang M, Xie W, Cai Q, Hou F, Liu Y, Chen W, Xiao L, Shen Y.	N	NA	2020	PPR	Isolation and Characterization of 2019-nCoV-like Coronavirus from Malayan Pangolins	3	24
10.1111/j.1600-0668.2007.00469.x	Xie X, Li Y, Chwang AT, Ho PL, Seto WH.	N	Indoor Air	2007	MED	How far droplets can move in indoor environments–revisiting the Wells evaporation-falling curve.	3	167
10.1111/tmi.13383	Velavan TP, Meyer CG.	Y	Trop Med Int Health	2020	MED	The COVID-19 epidemic.	3	70
10.1126/science.1118391	Li W, Shi Z, Yu M, Ren W, Smith C, Epstein JH, Wang H, Crameri G, Hu Z, Zhang H, Zhang J, McEachern J, Field H, Daszak P, Eaton BT, Zhang S, Wang LF.	N	Science	2005	MED	Bats are natural reservoirs of SARS-like coronaviruses.	3	967

Table 3. Most cited scientific papers in the scientific literature within COVID-19 Wikipedia corpus

Title	Year	Journal	Authors	Citation Count
Understanding the Warburg effect: the metabolic requirements of cell proliferation.	2009	Science	Vander Heiden MG, Cantley LC, Thompson CB.	4927
The MIQE guidelines: minimum information for publication of quantitative real-time PCR experiments.	2009	Clin Chem	Bustin SA, Benes V, Garson JA, Hellemans J, Huggett J, Kubista M, Mueller R, Nolan T, Pfaffl MW, Shipley GL, Vandesompele J, Wittwer CT.	4809
Isolation of a cDNA clone derived from a blood-borne non-A, non-B viral hepatitis genome.	1989	Science	Choo QL, Kuo G, Weiner AJ, Overby LR, Bradley DW, Houghton M.	3672
Isolation of a T-lymphotropic retrovirus from a patient at risk for acquired immune deficiency syndrome (AIDS).	1983	Science	Barré-Sinoussi F, Chermann JC, Rey F, Nugeyre MT, Chamaret S, Gruest J, Dauguet C, Axler-Blin C, Vézinet-Brun F, Rouzioux C, Rozenbaum W, Montagnier L.	3016
The American-European Consensus Conference on ARDS. Definitions, mechanisms, relevant outcomes, and clinical trial coordination.	1994	Am J Respir Crit Care Med	Bernard GR, Artigas A, Brigham KL, Carlet J, Falke K, Hudson L, Lamy M, Legall JR, Morris A, Spragg R.	2904
Toll-like receptors.	2003	Annu Rev Immunol	Takeda K, Kaisho T, Akira S.	2872
The acute respiratory distress syndrome.	2000	N Engl J Med	Ware LB, Matthay MA.	2720
Network biology: understanding the cell's functional organization.	2004	Nat Rev Genet	Barabási AL, Oltvai ZN.	2697
Surviving sepsis campaign: international guidelines for management of severe sepsis and septic shock: 2012.	2013	Crit Care Med	Dellinger RP, Levy MM, Rhodes A, Annane D, Gerlach H, Opal SM, Sevransky JE, Sprung CL, Douglas IS, Jaeschke R, Osborn TM, Nunnally ME, Townsend SR, Reinhart K, Kleinpell RM, Angus DC, Deutschman CS, Machado FR, Rubenfeld GD, Webb SA, Beale RJ, Vincent JL, Moreno R, Surviving Sepsis Campaign Guidelines Committee including the Pediatric Subgroup.	2461
A comprehensive analysis of protein-protein interactions in Saccharomyces cerevisiae.	2000	Nature	Uetz P, Giot L, Cagney G, Mansfield TA, Judson RS, Knight JR, Lockshon D, Narayan V, Srinivasan M, Pochart P, Qureshi-Emili A, Li Y, Godwin B, Conover D, Kalbfleisch T, Vijayadamodar G, Yang M, Johnston M, Fields S, Rothberg JM.	2416

SI datasets

(1) Table of scientific paper form europmc COVID-19 cited in wikipedia

(2) Table of Wikipedia article-DOI network

(3) Table of protected wikipedia COVID-19 articles

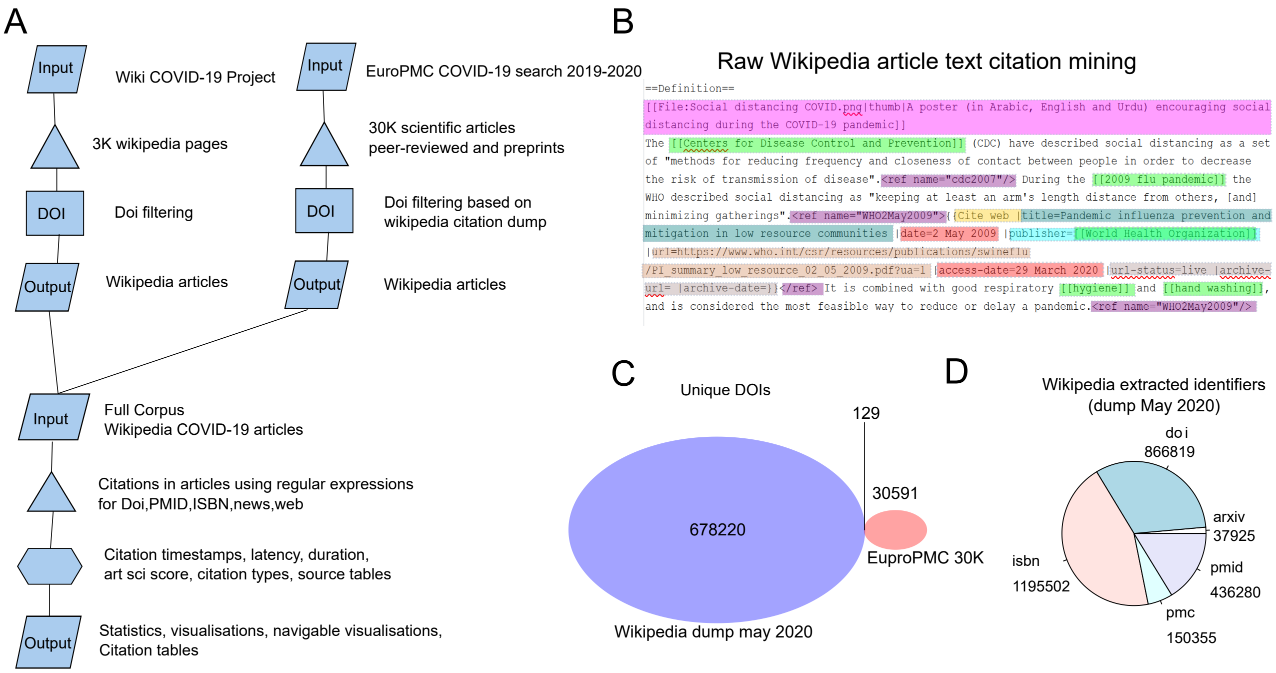

Figure S1. Corpus identification and citation extraction pipeline. A) Scheme of the Corpus delimitation rational and citation extraction. To delimit our corpus of Wikipedia articles containing Digital Object Identifier (DOI), we applied two different strategies. First we scraped every Wikipedia pages form the COVID-19 Wikipedia project (about 3K pages) and we filtered them to keep only page containing DOI citations (149 Wikipedia articles). For our second strategy, we made a search with EuroPMC on COVID-19, SARS-CoV2, SARS-nCoV19 (30,000 sci papers, reviews and preprints) and a selection on scientific papers form 2019 onwards that we compared to the Wikipedia extracted citations from the English Wikipedia dump of May 2020 (860'000 DOIs). This search led to 91 Wikipedia articles containing at least one citation of the EuroPMC search. Taken together, from our 231 Wikipedia articles corpus we extracted DOIs, PMIDs, ISBNs, websites and URLs using a set of regular expressions, as described in the methods. Subsequently, we computed several statistics for each Wikipedia article and we retrieved Atmetics, CrossRef and EuroPMC information for each DOI. Finally, our method allows to produce tables of citations annotated and extracted information in each Wikipadia articles such as books, websites, newspapers. In addition,a timeline of Wikipedia articles and a network of Wikipedia articles linked to scientific papers is built. B) Example of raw Wikipedia text from the social distancing article highlighted with several parsed items from a reference. pink: a hyperlink to an image file, green: Wikipedia hyperlinks, purple: reference, yellow: citation type, dark green: citation title, red: citation date, orange: citation URL. C) Overlap between DOI from the Wikipedia dump and the 30K EuroPMC COVID-19-related scientific articles and preprints D) number of extracted citations with *mwcite* from the English Wikipedia dump of May 2020.

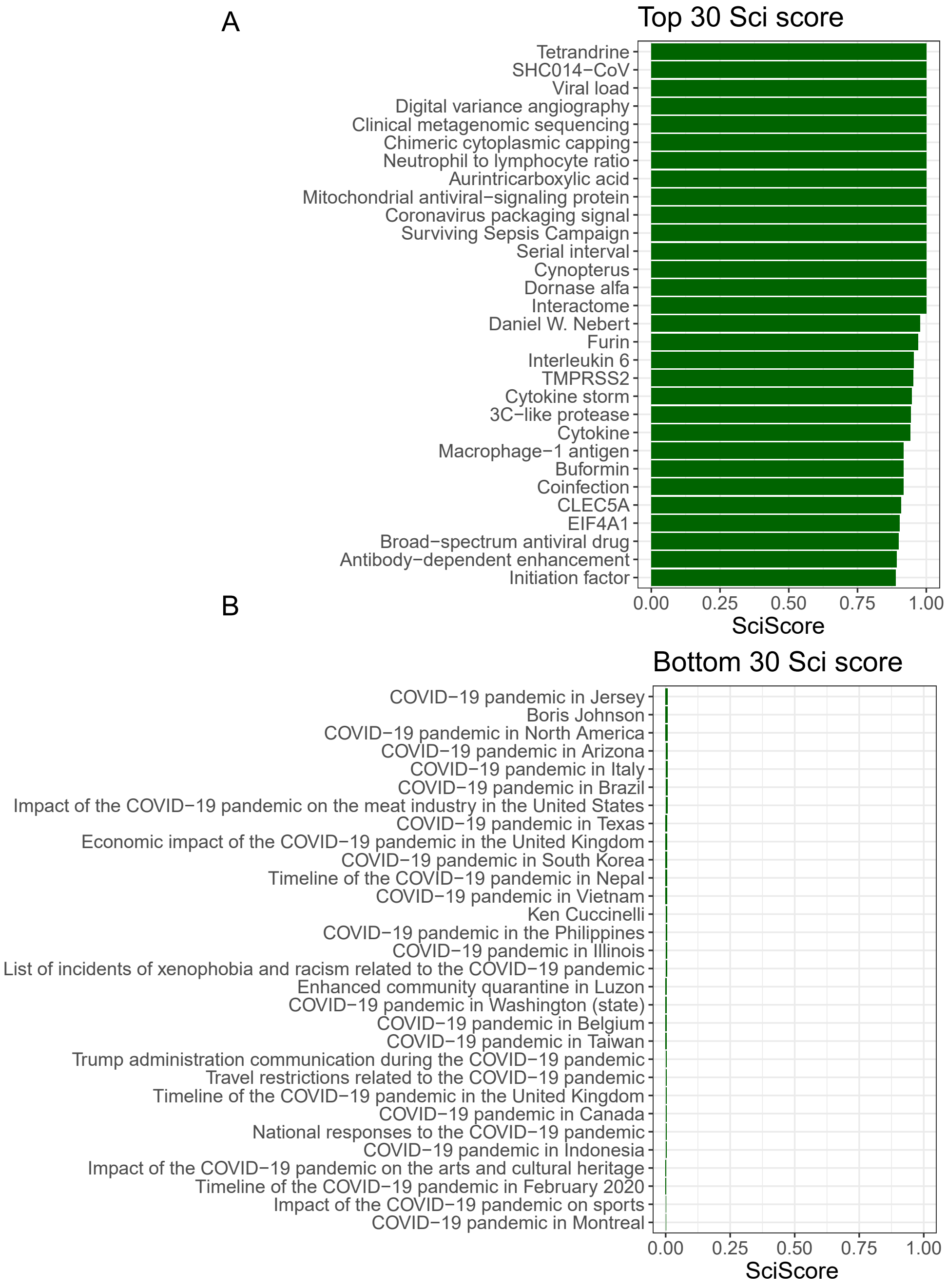

Figure S2. Top and bottom scientific score from Wikipedia article COVID-19 corpus. The scientific score was computed based on the reference content of each Wikipedia article from the COVID-19 corpus as defined in the methods section. A) Top 30 scientific article from the COVID-19 corpus. B) Bottom scientific article from the COVID-19 corpus.

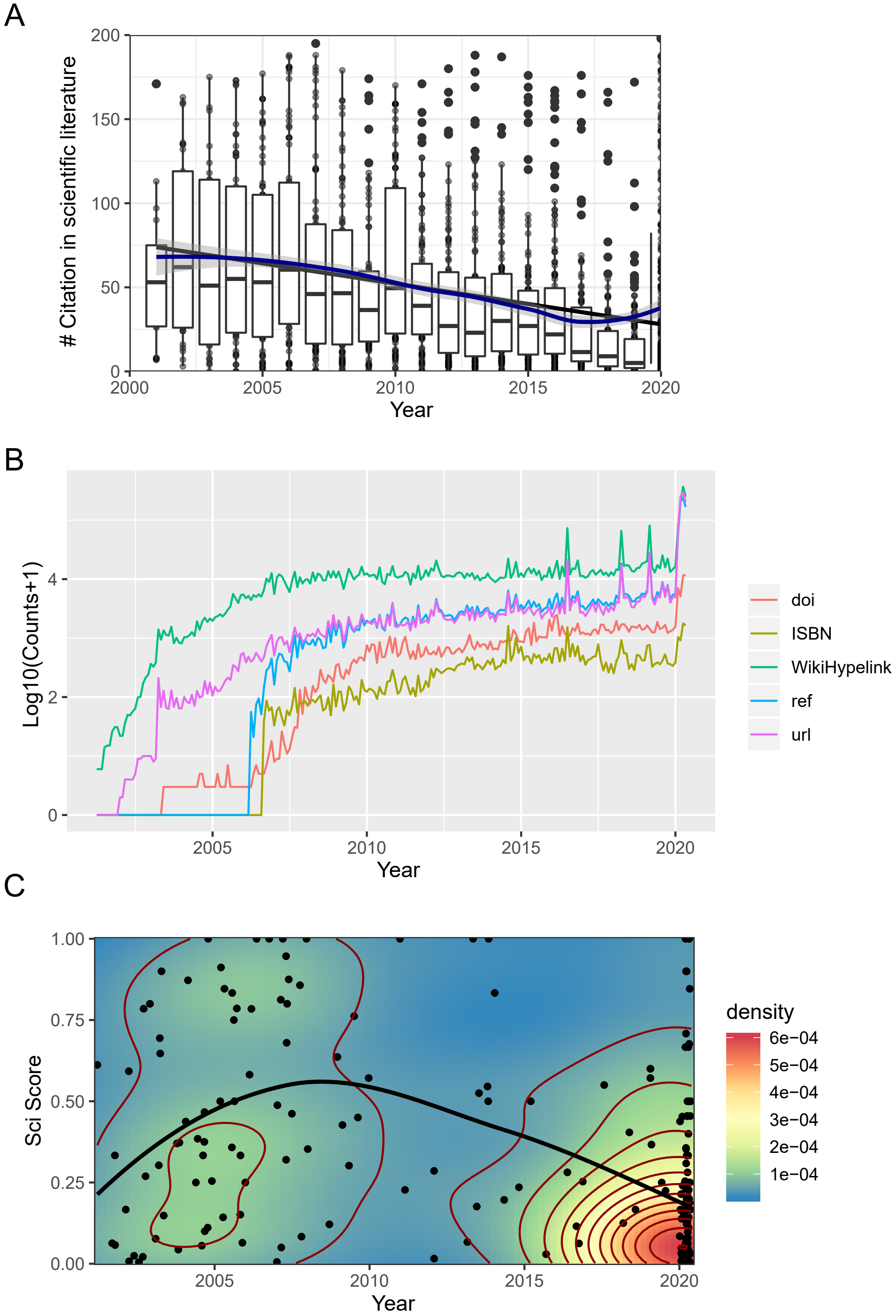

Figure S3. Historical perspective, citations count, citation type and scientific score of the COVID-19 corpus. A) Scientific literature citation count in function of the year of publication. B) Citation count in function of the year for different type of citation (doi, isbn, hyperlink, url). C) Scientific score in function of the creation date of wikipedia article.

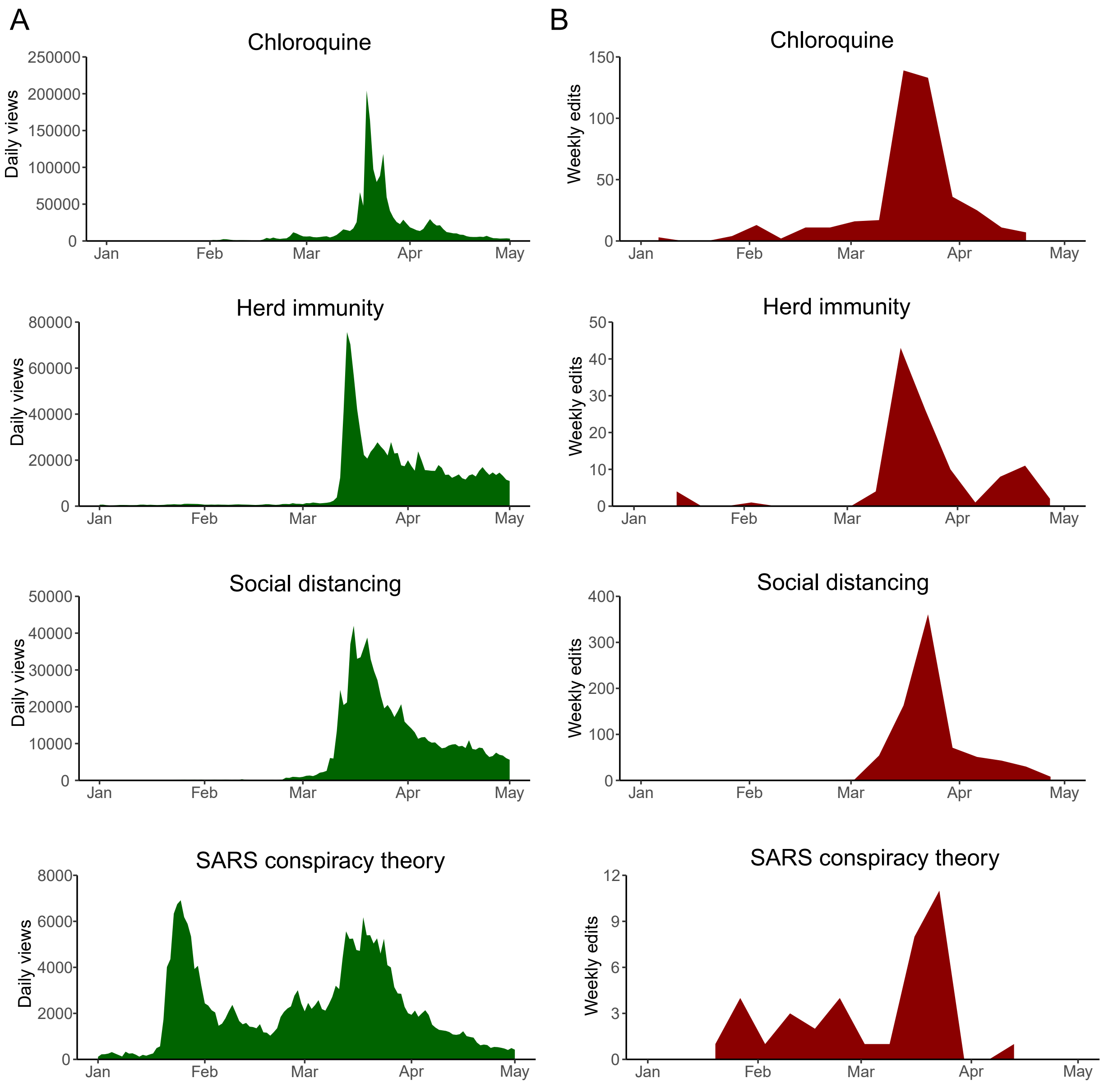

Figure S4. Wikipedia article page views and edits during COVID-19 pandemics. A) Daily page views and B) weekly edits for selected Wikipedia articles.

supp. data 2

Click here to access/download
Supplementary Material
Supp_data_Network.csv

supp. data 3

Click here to access/download
Supplementary Material
Supp_data_protected_corpus.csv

supp. data 1

Click here to access/download
Supplementary Material
supp_data_intesect_europmc_search_dump_doi_annotated.xlsx

Personal Cover Click here to access/download;Personal Cover;Cover_letter_GigaScience.docx

Submission of manuscript "Meta-Research: Citation needed? Wikipedia and the COVID-19 pandemic" by Benjakob, Aviram & Sobel

Dear editorial team,

Please consider for publication our manuscript "Citation needed? Wikipedia and the COVID-19 pandemic" in GigaScience.

With COVID-19 deemed an infodemic by the UN and WHO, and potential disinformation posing a threat to public health, Wikipedia has emerged as an unlikely source for accurate information about health and medicine online. Our study offers an in-depth analysis of the scientific backbone supporting Wikipedia's COVID-19 articles, using references as a readout. We found Wikipedia's editors preferred high quality sources - from both news media and from high impact factor academic journals, with a bias towards open access. Many of these respected sources were openly endorsed by the community of editors focused on safeguarding the quality of the virus articles. Our temporal analysis of the pandemic-related articles and their scientific references revealed that many articles based on scientific research were already in existence prior to the coronavirus' first wave (January-May 2020), while newer post-outbreak articles tended to be less scientific, showing a dynamic referencing pay off between scientificness and remaining up to date. By building a network of articles based on their shared sources, we show how Wikipedia's rigid sourcing policy for medical content - the same one that endorsed certain sources - manifested along large swathes of thematically related articles. Together, we claim all these created a "scientific infrastructure" that helped regulate the manner new information about the pandemic was integrated into Wikipedia and helped contextualize it along scientific lines.

This work is focused on the interface between academic research, popular media and the general public. We believe GigaScience will be the ideal journal for publication since it covers a particularly wide range of topics spanning these fields for a diverse readership. Moreover, GigaScience has also published a number of recent and important studies about Wikipedia, a field to which we hope our study may also contribute. GigaScience has placed open science at the heart of its scientific agenda and has helped push the open research into the academic discourse. As our paper is *about* open science and attempts uncovering the ways the open free online encyclopedia fights off disinformation by making scientific knowledge accessible to the wider public, we can think of no better home for our study.

Respectfully,

Dr. Jonathan Aryeh Sobel, Dr. Rona Aviram and Dr. Omer Benjakob
