# Supplementary material for: Citation needed? Wikipedia bibliometrics during the first wave of the COVID-19 pandemic
Source: Gigascience. 2022 Jan 12;11:giab095. doi: 10.1093/gigascience/giab095 (PMC8756189; doi:10.1093/gigascience/giab095)
Supplement: giab095_GIGA-D-21-00139_Revision_1 [file giab095_giga-d-21-00139_revision_1.pdf]

GigaScience

Citation needed? Wikipedia bibliometrics during the first wave of the COVID-19 pandemic.

--Manuscript Draft--

**Manuscript Number:**	GIGA-D-21-00139R1
**Full Title:**	Citation needed? Wikipedia bibliometrics during the first wave of the COVID-19 pandemic.
**Article Type:**	Research
**Funding Information:**	Azrieli Foundation — Dr. Rona Aviram<br>Placide Nicod Foundation — Dr. Jonathan Aryeh Sobel
**Abstract:**	Background<br><br>With the COVID-19 pandemic's outbreak, millions flocked to Wikipedia for updated information. Amid growing concerns regarding an "infodemic", ensuring the quality of information is a crucial vector of public health. Investigating if and how Wikipedia remained up to date and in line with science is key to formulating strategies to counter misinformation. Using citation analyses, we asked: which sources informed Wikipedia's COVID-19-related articles before and during the pandemic's first wave (January-May 2020).<br>Results<br><br>We found that coronavirus-related articles referenced trusted media sources and high-quality academic research. Moreover, despite a surge in COVID-19 preprints, Wikipedia had a clear preference for open-access studies published in respected journals and made little use of preprints. Building a timeline of English COVID-19 articles from 2001-2020 revealed a nuanced trade-off between quality and timeliness. It further showed how preexisting articles on key topics related to the virus created a framework for integrating new knowledge. Supported by a rigid sourcing policy, this "scientific infrastructure" facilitated contextualization and regulated the influx of new information. Lastly, we constructed a network of DOI-Wikipedia articles, which showed the shifting landscape of pandemic-related knowledge on Wikipedia and how academic citations create a web of shared knowledge supporting topics like COVID-19 vaccine development.<br>Conclusions<br><br>Understanding how scientific research interacts with the digital knowledge-sphere during the pandemic provides insight into how Wikipedia can facilitate access to science. It also reveals how, aided by what we term its "citizen encyclopedists", it successfully fended off COVID-19 disinformation and how this unique model may be deployed in other contexts.
**Corresponding Author:**	Jonathan Aryeh Sobel, Ph.D.<br>Technion Israel Institute of Technology<br>Haifa, ISRAEL
**Corresponding Author Secondary Information:**	
**Corresponding Author's Institution:**	Technion Israel Institute of Technology
**Corresponding Author's Secondary Institution:**	
**First Author:**	Omer Benjakob
**First Author Secondary Information:**	
**Order of Authors:**	Omer Benjakob<br>Rona Aviram, Ph.D.

Powered by Editorial Manager® and ProduXion Manager® from Aries Systems Corporation

	Jonathan Aryeh Sobel, Ph.D.
**Order of Authors Secondary Information:**	
**Response to Reviewers:**	GigaS Reviewer responses<br><br>Reviewer #1:<br><br>This is a very solid article on a timely topic. I also commend you for the thorough and meticulous methodology.<br><br>One thing that I believe you could amplify on is what would your proposed solution to the "trade off between timeliness and scientificness"? After all, Wikipedia relies on the sources that are reliable, verifiable, but foremostly... available. At the time when there are no academic journal articles published (yet) the chosen modus operandi does not appear to be a trade-off, it is basically the only logical solution. A trade-off would occur if the less valuable sources were not replaced when more academic ones appear, and this is not the case.<br><br>We thank the reviewers for their comments - specifically the need to clarify our proposed solution to the tradeoff we found between timeliness and scientificness; and the lack of some key references from our bibliography. Therefore, we have made the following two changes to our text:<br><br>In our results section, in the part discussing preprints and open-access (page 3, last paragraph; page 5, first paragraph) as well as in the discussion (page 10, fifth full paragraph) we explain that the speed of scientific publication is much slower than other types of sources.<br><br>Per your next comment, we have also elaborated on the cooperation between Wikipedia and the Cochrane database, to help explain the former's bias towards high-quality academic research (first in the results, on page 3 paragraph 5; and also in our discussion in page 10, third full paragraph).<br><br>In our discussion section (page 10 paragraphs 2-4, and page 11, paragraphs 1, 2 and 6), we have also now clarified this "trade off between timeliness and scientificness". We focus on how community mechanisms and official cooperation with Cochrane helped safeguard quality but also how in the future, will allow more academic sources to enter in and thus speculate a rise in the "scientificness" of the corpus.<br><br>I believe you should mention the fact that Wikipedia has an agreement with Cochrane database, which likely affects the popularity of this source.<br><br>The reviewer is correct, therefore we made the following changes to the text to include the fact that Wikipedia has an agreement with Cochrane database:<br><br>In the results, on page 3 paragraph 5, we note how Cochrane studies are among the most cited in our corpus - thus underscoring how such partnership directly helped support COVID-19 content; and also in our discussion in page 10, third full paragraph) we add that Wikipedia's "rigid sourcing policy" is supported by institutional cooperation like that it maintains with Cochrane.<br><br>On page 9 (third full paragraph), we expand on this, highlighting how Wikipedia's "medical reliable sources" policy (MEDRS) and in our aforementioned discussion discuss how it makes use of Cochrane as well as other institutional sources (e.g. WHO) to allow non-expert editors to enforce academic level sourcing policies and thus allow WP to maintain an explicit bias towards peer-reviewed studies for health content.<br><br>Additionally, I think that the literature review needs to be expanded. There are already some publications about Wikipedia and COVID-19, as well as about medical coverage on Wikipedia (some non-exhaustive references added below). Moreover, Wikipedia has been a topic covered in GigaScience and it would be reasonable to reflect on the

previous conversations in the journal in your publication.

We thank the reviewers for this rich list of sources that were missing from our work. We made the following changes to the text to incorporate them:

In our introduction (page 2, first full paragraph), we've expanded the line about “initial research into the coronavirus” to include Chrzanowski 2021 - which we have also added to our discussion to help contextualize public interest in the virus vis a vis Wikipedia (page 9, first paragraph of discussion). We've also updated the aforementioned line in the introduction to reference Colavizza 2020 (which was included in our bibliography as a preprint).

See: ”This research has shown both that traffic to Wikipedia's coronavirus articles reflected public interest in the pandemic (Chrzanowski 2021), and that these articles provide a representative sample of COVID-19 research (Colavizza 2020).

In our intro (page 2, middle of paragraph 1) and in our discussion (last paragraph starting on page 10), we've also added Jemielniak 2019 as well as Kagan 2020 (first paragraph of discussion, page 9) to add context on the relationship between academic journals and Wikipedia, and the later to help contextualize our scientometric findings regarding Wikipedia within the wider scientometric discourse on COVID.

In wake of comments from yourself and reviewer #3, we've also added a reference to a new preprint about editing patterns on WP during the pandemic (Keegan 2020, now in first paragraph of discussion, page 9) and a metastudy about research on Wikipedia's medical content; both have been added in our introduction and discussion to help situate our work within the growing corpus of research both on WP and medical content and research about it and coronavirus pandemic specifically.

Chrzanowski, J., Sołek, J., & Jemielniak, D. (2021). Assessing Public Interest Based on Wikipedia's Most Visited Medical Articles During the SARS-CoV-2 Outbreak: Search Trends Analysis. Journal of medical Internet research, 23(4), e26331.
Colavizza, G. (2020). COVID-19 research in Wikipedia. Quantitative Science Studies, 1-32.
Jemielniak, D. (2019). Wikipedia: Why is the common knowledge resource still neglected by academics?. GigaScience, 8(12), giz139.
Jemielniak, D., Masukume, G., & Wilamowski, M. (2019). The most influential medical journals according to Wikipedia: quantitative analysis. Journal of medical Internet Research, 21(1), e11429.
Kagan, D., Moran-Gilad, J., & Fire, M. (2020). Scientometric trends for coronaviruses and other emerging viral infections. GigaScience, 9(8), giaa085.

Reviewer #2:
This is a well-written manuscript. The methods are well-described. I've confined my comments to improving the reporting of your methods, some comments about the paper's structure, and a few about the readability of the figures and tables (which I think in general are too small, and difficult to read). Here are my main comments for your consideration as you work to improve your paper:

1) Title of manuscript - the title of your paper seems inadequate to me, and doesn't really convey its content. A more descriptive title that includes the idea of the "first wave" might be useful from my point of view as a reader who scans titles to see if I am interested. I'd recommend including words in the title that refer to your methods. What type of research is this - a quantitative analysis of citations? Title words say a lot about the robust nature of your methods. As you consider whether to keep your title as is, keep mind that title words will aid readers in understanding your research at a glance, and provide impetus to read your abstract (and one hopes the entire manuscript). These words will help researchers find the paper later as well via the Internet's many search engines (i.e., Google Scholar).

We thank the reviewer for the constructive suggestions on how to improve our title.

	Accordingly, we have renamed our manuscript to stress both the fact that the study focuses on the first wave of the pandemic and the bibliometric methods we used to understand it. The paper is now titled: “Citation needed? Wikipedia bibliometrics during the first wave of the COVID pandemic”.<br><br>2) Abstract - The abstract is well-written. Could the aims of your research be more obvious? and clearly articulated? How about using a statement such as "This research aims to" or similar? I also don't understand the sentence that begins with "Using references as a readout". What is meant by a "readout" in this context? Do you mean to read a print-out of references later? Lower down, you introduce the concept of Wikipedia's references as a "scientific infrastructure", and place it in quotations. Why is it in quotations? I wondered what the concept was on first reading it. A recurring web of papers in Wikipedia constitutes a set of core references - but would I call them a scientific infrastructure? Not sure; they are a mere sliver of the scientific corpus. Not sure I have any suggestions to clarify the use of this phrase.<br><br>We thank the reviewer for this feedback and have re-written our abstract to make our aims clearer and to make it better fit the GigaScience format. We’ve also dropped the word “readout” and swapped it for “metric” and apologize for the misleading terminology. We’ve also decided to drop the “scientific infrastructure” from the abstract and opted instead to explain what we mean by it: namely, the existence of rigid sourcing policy regarding medical and health content, institutional corporations (like WP’s partnership with Cochrane Network) to support it and and a specialized community of volunteers to enforce it; alongside the existence of articles relating to key scientific topics on coronavirus that predate the pandemic’s first wave and helped regulate the way new information was framed when it was integrated into WP during the pandemic).<br><br>Our new abstract reads as follows:<br>BACKGROUND: With the COVID-19 pandemic’s outbreak, millions flocked to Wikipedia for updated information. Amid growing concerns regarding an "infodemic", ensuring the quality of information is a crucial vector of public health. Investigating if and how Wikipedia remained up to date and in line with science is key to formulating strategies to counter misinformation. Using citation analyses, we asked: which sources informed Wikipedia’s COVID-19-related articles before and during the pandemic’s first wave (January-May 2020).<br>RESULTS: We found that coronavirus-related articles referenced trusted media sources and high-quality academic research. Moreover, despite a surge in COVID-19 preprints, Wikipedia had a clear preference for open-access studies published in respected journals and made little use of preprints. Building a timeline of English COVID-19 articles from 2001-2020 revealed a nuanced trade-off between quality and timeliness. It further showed how preexisting articles on key topics related to the virus created a framework for integrating new knowledge. Supported by a rigid sourcing policy, this "scientific infrastructure" facilitated contextualization and regulated the influx of new information. Lastly, we constructed a network of DOI-Wikipedia articles, which showed the shifting landscape of pandemic-related knowledge on Wikipedia and how academic citations create a web of shared knowledge supporting topics like COVID-19 vaccine development.<br>CONCLUSION: Understanding how scientific research interacts with the digital knowledge-sphere during the pandemic provides insight into how Wikipedia can facilitate access to science. It also reveals how, aided by what we term its "citizen encyclopedists", it successfully fended off COVID-19 disinformation and how this unique model may be deployed in other contexts.<br><br>3) Introduction - This is an excellent introduction to your paper, and it provides a lot of useful context and background. You make a case for positioning Wikipedia as a trusted source of information based on the highly selective literature cited by the entries. However, I would only caution that some COVID-19 entries cite excellent research but the content is contested, and vice versa. One suggestion I had for this section was the possibility of tying citizen science (part of open science) to the rise of Wikipedia's medwiki volunteers. Wikipedia provides all kinds of ways for citizens to get involved in science. As an open science researcher, I appreciated all of the open aspects you mention. Clearly, open access to Wikipedia in all languages is a driving force in

combatting misinformation generally, and the COVID "infodemic" specifically. I admit I struggled to understand the point of the section that begins, "Here, we asked what role does scientific literature, as opposed
to general media, play in supporting the encyclopedia's coverage of the COVID-19 as the pandemic spread." The opening sentence articulates your a priori research question, always welcome for readers. Would some of the information that follows in this section around your methods be better placed in the following section under the "Material and Methods"? I found it jarring to read that "....after the pandemic broke out we observed a drop in the overall percentage of academic references in a given coronavirus article, used here as a metric for gauging scientificness in what we term an article's Scientific Score." These two ideas are introduced again later, but I had no idea on reading them here what they signified or whether they were related to research you were building on. You might consider adding a parenthetical statement that they will be described later, and that the idea of a score is your own.

We thank the reviewer for their valuable suggestions and have made the following changes to the text:
We've deleted the line "we asked what role does scientific literature, as opposed to general media, play in supporting the encyclopedia's coverage of the COVID-19 as the pandemic spread" and relocated it as well the lines following "a temporal analysis" and "A network analysis" to the method section, which as our next response will explain we have significantly reworked in wake of your comments
In wake of next comment about the methods section needing a "preamble" to prepare readers, as well as completely rewriting the ending of our introduction section (first full paragraph on page 2), the methods section now also has an introductory graph of its own for that end (2nd full paragraph on page 2). It reads thus:
"Using citations as a metric for gauging the scientificness of Wikipedia articles along these three axes allowed us to provide a "scientific score" for them and ask: what shifts in scientificness did the COVID-19 articles undergo during the period researched. At the level of the citations inside any given Wikipedia article, we could provide a second metric, namely the latency which allowed us to get a historical perspective on the scientific infrastructure supporting them. Moreover our work explored Wikipedia's articles' versions history and co-citations, to gain an insight on COVID-19 knowledge and its growth since the creation of the digital encyclopedia in 2001 and up until 2020. Though predominantly quantitative, for some selected articles we also examined the different claims the citations were used to support, at different stages, and reviewed some of the textual changes that articles underwent in wake of the coronavirus outbreak, to provide anecdotal qualitative context to our findings."

Regarding the use of citizen science, we feel this is an important point - so much so that it is now part of the abstract's conclusions. Moreover, in the text, it was addressed in a reformulation of key parts of our discussion section. On page 11 (first full paragraph) we've added a paragraph about citizen science and attempt to tie the term "citizen encyclopedists" to Wikipedia's medwiki volunteers, thus more clearly linking between the open science discourse and citizen science research to our own findings. We've also cited past citizen science studies we ourselves have conducted (Benjakob & Aviram 2018; Sobel 2017).
Our new methods section introductory graph also now notes that we did not examine the manner the citations were actually represented in the text and ideally (were it not for length considerations0 we'd also add small cautionary note that though COVID-19 articles cite excellent academic research, we did not examine how they were represented in the text - and thus explain that WP article's content may be contested for technical or editorial or stylistic reasons by members of the community regardless of the quality of the sourcing behind it. Nonetheless, we feel our solution is an adequate response to your accurate point.

4) Material and methods - Your methods section might benefit from writing a preamble to prepare your readers. As already mentioned, consider taking some of the previous section and recasting it as an introduction to your methods. Consider adding some information to orient readers, and elaborating in a sentence or two about why identifying COVID-19 citations / information sources is an important activity. By the way, what is meant by this: "To delimit the corpus of Wikipedia articles containing

DOIs"? Do you mean "identify" Wikipedia articles with DOIs in their references? As I mentioned (apologies in advance for the repetition), it strikes me as odd that you don't refer to this research as a form of citation analysis (isn't that what it is?). Instead you characterize it as "citation counting". If your use of words has been intentional, is there a distinction you are making that I simply do not understand? Also: bibliometricians and/or scientometricians might wonder why you
avoid the phrase citation analysis. Further to your methods which are primarily quantitative and statistical - what are the qualitative methods used throughout the paper to analyze the data? How did you carry out this qualitative work? (On page 10, you state "we set out to examine in a temporal, qualitative and quantitative manner, the role of references in articles linked directly to the pandemic as it broke.") That part of your methods seems to be a bit under-developed, and may be worth reconsidering as you work to improve your reporting in the manuscript.

We thank the reviewer for their comments and have made the following changes to the text:
As noted, in wake of the comments regarding our introduction and methods section we've now re-edited both and created a new intro section for the methods (2nd full paragraph on page 2).
In the introduction (page 1 and first graph of page 2) and in the new preamble to the methods section we now explicitly label our work as a "citation analysis" in line with "bibliometrics and scientometrics" and have dropped "citation counting" from the text.
In the introduction section, ,we've now stressed why we are using footnotes and references as a "metric" (previously labeled as a "read out") for scientificness.
In the methods section, we no longer use the term "identify" and we textually clarify that we mean that academic papers are counted as all those references that have DOIs (see subsection "Corpus Delimitation" and "DOI Corpus Content Analysis and DOI Sets Comparison" on page 2, full paragraphs 3 and 4)
On page 10 we've dropped the noted line from ("we set out to examine in a temporal, qualitative and quantitative manner, the role of references in articles linked directly to the pandemic as it broke.") Instead we have clarified the three methods/perspectives we used (in the new intro to the methods section) to stress the quantitative aspect of our work and differentiate it from the qualitative work conducted (textual analysis of articles' and papers' titles, as well as some anecdotal comparison of articles wordings as part of our temporal and network analyses; noted in the methods section intro text.

5) Table 1. I am not sure what this table adds to the methods given it leads off your visuals. Do you really need it? It doesn't reveal anything to me and could be in a supplemental file. I also have difficulties in properly seeing table 1; perhaps you could make it larger and more readable?

We agree with the reviewer and have moved the table to supplementary files, and have enlarged the font for readability.

6) Figure 1. This is the most informative visual in the paper but it is hard to read and crowded. It deserves more space or the information it provides is not fully understood.

We apologize for the lack of clarity and have adjusted the figure to better visualize our analyses.

7) Figure 3. This is very bulky as a figure, although informative. Again, I'm not sure all of it needs inclusion. Perhaps select part of it, and include other parts in a supplement.

We thank the reviewer for this suggestion. This figure is highly important to convey the historical dynamics of this group of articles thus we moved the former figure 3 panel B in supplement (now figure S3) and we increased the size of the other panels. We believe all the panels complement each other and are necessary to comprehend the story.

7) Limitations - The paper does not adequately address its limitations. A more fulsome evaluation of limitations would be beneficial to me as a reader, as it would place your

Powered by Editorial Manager® and ProduXion Manager® from Aries Systems Corporation

work in a larger context. For example, consider asking whether the results are indicative of Wikipedia's other medical or scientific entries? Or are the results not generalizable at all? In other works, are they indicative of something very limited based on the timeframe that you examined? I found myself disagreeing with: "....the mainstream output of scientific work on the virus predated the pandemic's outbreak to a great extent". Is this still true? and what might its significance be now that we are in 2021? Would it be helpful to say that most of the foundational research re: the family of coronaviruses was published pre-2020, but entries about COVID-19 disease and treatment entries are now distinctly different in terms of papers cited, especially going forward. Wiki editors identify relevant papers over time but are not adept at identifying emerging evidence in my experience, or at incorporating important papers early; it's strange given that recency is one of its true calling cards. For me, the most confounding aspect of the infodemic is the constant shifts of evidence, and how to respond in a way that is prudent and evidence-based. As you point out, Wikipedia has a 8.7 year latency in citing highly relevant papers - and, it seem likely that many important COVID-19 papers were neglected in Wikipedia in the first wave especially about the disease. As you point out, this will form part of future research, which I hope you and your team will pursue.

We thank the review for this important point and have amended the text so it now has a special limitation section (page 11, paragraphs 3-7) that clarifies what can be generalized - both about coronavirus and generally - and what future work can be done in this regard. Though what happened in 2021 is currently beyond the scope of the paper, we do feel that there are many points that can be generalized and even exported to other contexts. This new clarified discussion includes and begins with the limitations already noted in the text, on page 11 (starting with “Wikipedia’s main advantage is in many ways…” and until “Therefore, our study is focused on the pandemic’s first wave and its history…”) and is followed by a new limitations paragraph that touches to both our study’s scope and the ability to generalize.

8) Reference 31 lacks a source: Amit Arjun Verma and S. Iyengar. Tracing the factoids: the anatomy of information reorganization in wikipedia articles. 2021.

We have amended the following footnote as such: Verma, Amit Arjun, Neeru Dubey, S. R. S. Iyengar, and Simran Setia. "Tracing the Factoids: the Anatomy of Information Re-organization in Wikipedia Articles." In Companion Proceedings of the Web Conference 2021, pp. 572-579. 2021.

Reviewer #3:

The present manuscript provides an overview of how the English Wikipedia incorporated COVID-19-related information during the first months of the ongoing COVID-19 pandemic.
It focuses on information supported by academic sources and considers how specific properties of the sources (namely their status with respect to open access and preprints) correlate with their incorporation into Wikipedia, as well as the role of existing content and policies in mediating that incorporation.

No aspect of the manuscript would justify a rejection but there are literally lots of opportunities for improvements, so "Major revision" appears to be the most appropriate recommendation at this point.

General comments

The main points that need to be addressed better:
(1) documentation of the computational workflows; JS:THIS IS FOR ME….
(2) adaptability of the Wikipedia approach to other contexts;
(3) descriptions of or references to Wikipedia workflows;
(4) linguistic presentation.

We thank the review both for their contribution to the WikiProject COVID-19 and for their detailed response. We took extra care not just to address the reviewer's

Powered by Editorial Manager® and ProduXion Manager® from Aries Systems Corporation

comments, but also the comments and feedback from the Wikipedia community itself to which they referred us. As our paper deals with the benefits of Wikipedia's model, it seems fitting to take into account the peer-review of our work as well as the citizen/crowd review conducted by the community of volunteer editors.
In wake of both we have made the following changes to the text (our response is proceeded by your elaborated comment):

Ad 1: while the code used for the analyses and for the visualizations seems to be shared rather comprehensively, it lacks sufficient documentation as to what was done in what order and what manual steps were involved. This makes it hard to replicate the findings presented here or to extend the analysis beyond the time frame considered by the authors.

We have updated the documentation on our computational methods in the Github repository of the project and we have added an introductory paragraph to our text's methods' section to clarify specific aspects - as requestedby both reviewer 2 and 3. The R package is still in active development and will be the object of another publication in 2022. Currently a beta version of the R package is available. The question of reproducibility is very important. The package documentation is designed to help users replicate some part of the analysis such as getting the text and the past versions of a given wikipedia page, extracting and parsing citations as well as annotating DOI or ISBN with title, authors, date, and more. However, the analysis made for this study is much more complex. Specifically, the code interacts with third party databases such as EuroPMC or Altmetric - the content of which evolves over time. For instance, it is not easily possible to get the citation count of a scientific publication two years ago. The information scraped is always the most recent one. In addition our study involved some analysis on the whole wikipedia dump. For this part I used mwcite (a python package to extract citations from the wikipedia dump) on a computing cluster as the size of the data was quite large. Doing the same analysis on a laptop would require weeks of computation. Unfortunately, the whole study is not fully replicable easily as the code was not designed for this purpose (i.e several scripts used on laptops and cluster, evolution of the information on 3rd party db, manual parts).

Ad 2: The authors allude to how pre-existing Wikipedia content and policies - which they nicely frame as Wikipedia's "scientific infrastructure" or "scientific backbone" - "may provide insight into how its unique model may be deployed in other contexts" but that potentially most transferable part of the manuscript - which would presumably be of interest to many of its readers - is not very well developed, even though that backbone is well described for Wikipedia itself.

In wake of this comment and others noted earlier on by yourself as well as reviewer #1, we've expanded our discussion page 11 (starting with "Wikipedia's main advantage is in many ways…" and until "Therefore, our study is focused on the pandemic's first wave and its history…") to include a part about the study focusing solely on English Wikipedia. We've also added a special limitation section that clarifies what aspects of the infrastructure can be generalized - both about coronavirus and generally - and what future work can be done in this regard.

To further stress the wider applicability of our findings, we also now note calls to have the CDC embarace Wikipedia-like processes to communicate information about disease (See paragraph 2 on page 11)

The remaining response we have not mapped onto yours, which is preserved as a list following our responses for the sake of clarity:
Re typos, spelling and grammar issues: We've fixed the noted typo (Whales vs "Charles, Prince of Wales"); and have also sent out the text for a professional proofreading
We've added the two references you note (as well as others noted by reviewer 1). The first is the preprint about editing patterns on WP during the pandemic (see Keegan 2020, now in first paragraph of discussion, page 9) and the second a metastudy about research on Wikipedia's medical content; both have been added in our intoro and our discussion to help situate our work within the growing corpus of research both on WP and medical content and research about it and coronavirus pandemic specifically.

Powered by Editorial Manager® and ProduXion Manager® from Aries Systems Corporation

In wake of the rich comments supplied by members of the Wikipedia community in the linked discussions, we have worked to correctly portray Wikipedia's workflows and processes, and have amended the following:

One comment we received had to do with terminology regarding what we termed a "banner" referring all those visiting WP articles on all topics during the first wave. We described this banner as being "official". However, that was misleading. The "official" banner has now been recast as two distinct initiatives/process: one led by the community (in the form of a temporary "In the News" banner, created by consensus vote by the WikiProject COVID-19) and the second a (contested) "executive order" (by the WMF) to have a permanent header banner on all articles in English (page 9, first full paragraph).

In the limitation section (paragraph 3-7, page 11), we added text to note that our focus was on medical content and therefore it is not surprising that little sources of this type were found in our corpus; however, by the same token, we also note and briefly discuss how (by using DOI filtering) one could have also easily found other topics as well, for instance social studies. As a result, we now note, there may also be room to include social research (for example studies on public health or policy) on social topics, too.

In this regard, we also added a line that the reason for the low score of many of the articles dealing with the outcome of the pandemic are that non-health topics are not bound by the MEDRS. This actually underscores our findings and reaffirms the need to also include academic sources on social topics.

One key insight we missed and the community flagged for us was the prevalence of coronavirus articles in WP's 50 top most viewed articles of 2020. In fact, the "COVID-19 pandemic" article which we focus on was actually WP's most read article in 2020, with 83,764,908 page views; the scientific article for "coronavirus" was 6th in 2020 (33,689,841) and COVID-19 pandemic by country and territory was 8th most popular (29,637,765) (cite: https://en.wikipedia.org/wiki/Wikipedia:Top_50_Report).

The list also provides key insights, for example the fact that traffic to these articles peaked during the first wave, the same period our study focuses on - see new addition to our intro (page 1 and first paragraph of page 2)

Reviewer 3 comments (continue list):

Ad 3: there is a good number of cases where the Wikipedia workflows are misrepresented (sometimes ever so slightly), and while many of these do not affect the conclusions, some actually do, and overall comprehension is hampered. I highlighted some of these cases, and others have been pointed out in community discussions, notably at https://eur01.safelinks.protection.outlook.com/?url=https%3A%2F%2Fen.wikipedia.org%2Fw%2Findex.php%3Ftitle%3DWikipedia_talk%3AWikiProject_COVID-19%26oldid%3D1028476999%23Review_of_Wikipedia%27s_coverage_of_COVID&data=04%7C01%7Cjsobel%40campus.technion.ac.il%7C2c478070cead41240ecb08d930a538af%7Cf1502c4cee2e411c9715c855f6753b84%7C1%7C0%7C637594309647239523%7CUnknown%7CTWFpbGZsb3d8eyJWIjoiMC4wLjAwMDAiLCJQIjoiV2luMzIiLCJBTiI6Ik1haWwiLCJXVCI6Mn0%3D%7C1000&sdata=CuaJVBu4Ujrp1uTjQtk%2ByCdB3SloS7c5kAKH24R7vrM%3D&reserved=0 and https://eur01.safelinks.protection.outlook.com/?url=http%3A%2F%2Fbluerasberry.com%2F2021%2F06%2Freview-of-paper-on-wikipedia-and-covid%2F&data=04%7C01%7Cjsobel%40campus.technion.ac.il%7C2c478070cead41240ecb08d930a538af%7Cf1502c4cee2e411c9715c855f6753b84%7C1%7C0%7C637594309647239523%7CUnknown%7CTWFpbGZsb3d8eyJWIjoiMC4wLjAwMDAiLCJQIjoiV2luMzIiLCJBTiI6Ik1haWwiLCJXVCI6Mn0%3D%7C1000&sdata=6IQRv2tp9JkkBPvRZJfqoHpk6MnxE%2BeT0fRMujbJmiY%3D&reserved=0 . Some resources particularly relevant to these parts of the manuscript have not been mentioned, be it scholarly ones likehttps://eur01.safelinks.protection.outlook.com/?url=https%3A%2F%2Farxiv.org%2Fabs%2F2006.08899&data=04%7C01%7Cjsobel%40campus.technion.ac.il%7C2c478070cead41240ecb08d930a538af%7Cf1502c4cee2e411c9715c855f6753b84%7C1%7C0%7C637594309647239523%7CUnknown%7CTWFpbGZsb3d8eyJWIjoiMC4wLjAwMDAiLCJQIjoiV2luMzIiLCJBTiI6Ik1haWwiLCJXVCI6Mn0%3D%7C1000&sdata=eMW3WIv4Qp%2FTOODcNblCjH2gWlxQZAV4NLFH%2FQXOaw8%3D&reserved=0 and https://eur01.safelinks.protection.outlook.com/?url=https%3A%2F%2Fdoi.org%2F10.1371%2Fjournal.pone.0228786&data=04%7C01%7Cjsobel%40campus.technion.a

c.il%7C2c478070cead41240ecb08d930a538af%7Cf1502c4cee2e411c9715c855f6753b84%7C1%7C0%7C637594309647239523%7CUnknown%7CTWFpbGZsb3d8eyJWIjoiMC4wLjAwMDAiLCJQIjoiV2luMzIiLCJBTiI6Ik1haWwiLCJXVCI6Mn0%3D%7C1000&sdata=oiHH1MLYvIBD7%2FUKA%2FiIYG99REY3NK1bkbgREAFGGYc%3D&reserved=0 or Wikimedia ones like https://eur01.safelinks.protection.outlook.com/?url=https%3A%2F%2Fen.wikipedia.org%2Fwiki%2FWikipedia_coverage_of_the_COVID-19_pandemic&data=04%7C01%7Cjsobel%40campus.technion.ac.il%7C2c478070cead41240ecb08d930a538af%7Cf1502c4cee2e411c9715c855f6753b84%7C1%7C0%7C637594309647239523%7CUnknown%7CTWFpbGZsb3d8eyJWIjoiMC4wLjAwMDAiLCJQIjoiV2luMzIiLCJBTiI6Ik1haWwiLCJXVCI6Mn0%3D%7C1000&sdata=z5mJrF6vPquVYBskQFkbYE%2BBrAkfyviYVFQ29apWQpY%3D&reserved=0 and https://eur01.safelinks.protection.outlook.com/?url=https%3A%2F%2Fcommons.wikimedia.org%2Fwiki%2FFile%3AWikimedia_Policy_Brief_-_COVID-19_-_How_Wikipedia_helps_us_through_uncertain_times.pdf&data=04%7C01%7Cjsobel%40campus.technion.ac.il%7C2c478070cead41240ecb08d930a538af%7Cf1502c4cee2e411c9715c855f6753b84%7C1%7C0%7C637594309647239523%7CUnknown%7CTWFpbGZsb3d8eyJWIjoiMC4wLjAwMDAiLCJQIjoiV2luMzIiLCJBTiI6Ik1haWwiLCJXVCI6Mn0%3D%7C1000&sdata=2UdB7PjBSY%2BrMxPdeMoRn4hJ5WJLv1KAx0OsKLSGu3c%3D&reserved=0 . Likewise
essentially missing - although this is a common feature in academic articles about Wikipedia - is a discussion of how valid the observations made for the English Wikipedia are in the context of other language versions (e.g. Hebrew). On that basis, it is understandable that no attempt is made to look beyond Wikipedia to see how coverage of the pandemic was handled in other parts of the Wikimedia ecosystem (e.g. Wikinews, Wikisource, Wikivoyage, Wikimedia Commons and Wikidata), but doing so might actually strengthen the above case for deployability of the Wikipedia approach in other contexts. Disclosure: I am closely involved with WikiProject COVID-19 on Wikidata too, e.g. as perhttps://eur01.safelinks.protection.outlook.com/?url=https%3A%2F%2Fdoi.org%2F10.5281%2Fzenodo.4028482&data=04%7C01%7Cjsobel%40campus.technion.ac.il%7C2c478070cead41240ecb08d930a538af%7Cf1502c4cee2e411c9715c855f6753b84%7C1%7C0%7C637594309647239523%7CUnknown%7CTWFpbGZsb3d8eyJWIjoiMC4wLjAwMDAiLCJQIjoiV2luMzIiLCJBTiI6Ik1haWwiLCJXVCI6Mn0%3D%7C1000&sdata=bavEHvLbsSQ8Ph8suaAtH7mBFktlcYc5UtL8seqXta4%3D&reserved=0 .

Ad 4: The relatively high number of linguistic errors - e.g. typos, grammar, phrasing and also things like internal references or figure legends - needlessly distracts from the value of the paper. The inclusion of figures - both via the text body and via the supplement - into the narrative is also sometimes confusing and would benefit from streamlining.

While GigaScience has technically asked me to review version 3 of the preprint (available via https://eur01.safelinks.protection.outlook.com/?url=https%3A%2F%2Fwww.biorxiv.org%2Fcontent%2F10.1101%2F2021.03.01.433379v3&data=04%7C01%7Cjsobel%40campus.technion.ac.il%7C2c478070cead41240ecb08d930a538af%7Cf1502c4cee2e411c9715c855f6753b84%7C1%7C0%7C637594309647239523%7CUnknown%7CTWFpbGZsb3d8eyJWIjoiMC4wLjAwMDAiLCJQIjoiV2luMzIiLCJBTiI6Ik1haWwiLCJXVCI6Mn0%3D%7C1000&sdata=nwPgaHDjGXnTYMC%2Bh8f5uJKU62pGB3rxjow%2FpHYFM%2BU%3D&reserved=0 and also via GigaScience's editorial system), that version was licensed incompatibly with publication in GigaScience, so I pinged the authors on this (via https://eur01.safelinks.protection.outlook.com/?url=https%3A%2F%2Ftwitter.com%2FEvoMRI%2Fstatus%2F1393114202349391872&data=04%7C01%7Cjsobel%40campus.technion.ac.il%7C2c478070cead41240ecb08d930a538af%7Cf1502c4cee2e411c9715c855f6753b84%7C1%7C0%7C637594309647239523%7CUnknown%7CTWFpbGZsb3d8eyJWIjoiMC4wLjAwMDAiLCJQIjoiV2luMzIiLCJBTiI6Ik1haWwiLCJXVCI6Mn0%3D%7C1000&sdata=oRdGGGxCbdF0x0dYqf7bj2NzOeZt1hW2gp6YQ1Q9rUE%3D&reserved=0), which resulted (with some small additional changes) in the creation of version 4 (available viahttps://eur01.safelinks.protection.outlook.com/?url=https%3A%2F%2Fwww.biorxiv.org%2Fcontent%2F10.1101%2F2021.03.01.433379v4&data=04%7C01%7Cjsobel

	%40campus.technion.ac.il%7C2c478070cead41240ecb08d930a538af%7Cf1502c4cee2e411c9715c855f6753b84%7C1%7C0%7C637594309647239523%7CUnknown%7CTWFpbGZsb3d8eyJWIjoiMC4wLjAwMDAiLCJQIjoiV2luMzIiLCJBTiI6Ik1haWwiLCJXVCI6Mn0%3D%7C1000&sdata=g%2BL57WahRKRMQ33YFWw7AvncDAAnLEjFaICVD0Q%2FFkI%3D&reserved=0) that I concentrated on in my review. Production of that version 4 - of which I eventually used both the PDF and the HTML, which became avaailable to me at different times - took a while, during which I had a first full read of the manuscript in version 3.<br><br>In an effort to explore how to make the peer review process more transparent than simply sharing the correspondence, I recorded myself while reading the manuscript for the second time, commenting on it live. These recordings are available viahttps://eur01.safelinks.protection.outlook.com/?url=https%3A%2F%2Fdoi.org%2F10.5281%2Fzenodo.4909923&data=04%7C01%7Cjsobel%40campus.technion.ac.il%7C2c478070cead41240ecb08d930a538af%7Cf1502c4cee2e411c9715c855f6753b84%7C1%7C0%7C637594309647239523%7CUnknown%7CTWFpbGZsb3d8eyJWIjoiMC4wLjAwMDAiLCJQIjoiV2luMzIiLCJBTiI6Ik1haWwiLCJXVCI6Mn0%3D%7C1000&sdata=8BMqAccH07SCR5joShENMVk2TdupXViA7Xz3YdUKTfQ%3D&reserved=0 .<br><br>In terms of specific comments, I annotated version 4 directly using Hypothes.is, and these annotations are available viahttps://eur01.safelinks.protection.outlook.com/?url=https%3A%2F%2Fvia.hypothes.is%2Fhttps%3A%2F%2Fwww.biorxiv.org%2Fcontent%2F10.1101%2F2021.03.01.433379v4.full&data=04%7C01%7Cjsobel%40campus.technion.ac.il%7C2c478070cead41240ecb08d930a538af%7Cf1502c4cee2e411c9715c855f6753b84%7C1%7C0%7C637594309647239523%7CUnknown%7CTWFpbGZsb3d8eyJWIjoiMC4wLjAwMDAiLCJQIjoiV2luMzIiLCJBTiI6Ik1haWwiLCJXVCI6Mn0%3D%7C1000&sdata=h8mhRueXII5yHz71x8qLIAPiAMxWapjEoSQad5fuW%2Fo%3D&reserved=0 .
**Additional Information:**	
**Question**	**Response**
Are you submitting this manuscript to a special series or article collection?	No
**Experimental design and statistics**<br><br>Full details of the experimental design and statistical methods used should be given in the Methods section, as detailed in our Minimum Standards Reporting Checklist. Information essential to interpreting the data presented should be made available in the figure legends.<br><br>Have you included all the information requested in your manuscript?	Yes
**Resources**<br><br>A description of all resources used, including antibodies, cell lines, animals and software tools, with enough information to allow them to be uniquely	Yes

Powered by Editorial Manager® and ProduXion Manager® from Aries Systems Corporation

identified, should be included in the Methods section. Authors are strongly encouraged to cite Research Resource Identifiers (RRIDs) for antibodies, model organisms and tools, where possible.<br><br>Have you included the information requested as detailed in our Minimum Standards Reporting Checklist?	
**Availability of data and materials**<br><br>All datasets and code on which the conclusions of the paper rely must be either included in your submission or deposited in publicly available repositories (where available and ethically appropriate), referencing such data using a unique identifier in the references and in the "Availability of Data and Materials" section of your manuscript.<br><br>Have you have met the above requirement as detailed in our Minimum Standards Reporting Checklist?	Yes

Powered by Editorial Manager® and ProduXion Manager® from Aries Systems Corporation

Click here to
access/download;Manuscript;Benjakob_etal_GigaScience_WI

OXFORD (GIGA)n SCIENCE

GigaScience, 2021, 1–24

doi: xx.xxxx/xxxx
Manuscript in Preparation
Research

RESEARCH

Citation needed? Wikipedia bibliometrics during the first wave of the COVID pandemic.

Omer Benjakob[1,*,§], Rona Aviram[2,†,§] and Jonathan Aryeh Sobel[2,3,‡,§]

[1]The Cohn Institute for the History and Philosophy of Science and Ideas, Tel Aviv University, Tel Aviv, Israel and [2]Weizmann Institute of Science, Rehovot, Israel and [3]Faculty of Biomedical Engineering, Technion-IIT, Haifa, Israel

[*]omerbj@gmail.com
[†]anorona@gmail.com
[‡]jsobel83@gmail.com
[§]Contributed equally.

Abstract

Background With the COVID-19 pandemic's outbreak, millions flocked to Wikipedia for updated information. Amid growing concerns regarding an "infodemic", ensuring the quality of information is a crucial vector of public health. Investigating if and how Wikipedia remained up to date and in line with science is key to formulating strategies to counter misinformation. Using citation analyses, we asked: which sources informed Wikipedia's COVID-19-related articles before and during the pandemic's first wave (January-May 2020).

Results We found that coronavirus-related articles referenced trusted media sources and high-quality academic research. Moreover, despite a surge in COVID-19 preprints, Wikipedia had a clear preference for open-access studies published in respected journals and made little use of preprints. Building a timeline of English COVID-19 articles from 2001-2020 revealed a nuanced trade-off between quality and timeliness. It further showed how preexisting articles on key topics related to the virus created a framework for integrating new knowledge. Supported by a rigid sourcing policy, this "scientific infrastructure" facilitated contextualization and regulated the influx of new information. Lastly, we constructed a network of DOI-Wikipedia articles, which showed the shifting landscape of pandemic-related knowledge on Wikipedia and how academic citations create a web of shared knowledge supporting topics like COVID-19 vaccine development.

Conclusions Understanding how scientific research interacts with the digital knowledge-sphere during the pandemic provides insight into how Wikipedia can facilitate access to science. It also reveals how, aided by what we term its "citizen encyclopedists", it successfully fended off COVID-19 disinformation and how this unique model may be deployed in other contexts.

Key words: COVID-19; Wikipedia; Infodemic; sources; bibliometrics; citizen science; open science

Introduction

Wikipedia has over 130,000 different articles relating to health and medicine [1]. The website as a whole, and specifically its medical and health articles, like those about disease or drugs, are a prominent source of information for the general public [2]. Studies of readership and editorship of health articles reveal that medical professionals are active consumers of Wikipedia and make up roughly half of those involved in editing these articles in English [3, 4]. Research conducted into the quality and scope of medical content deemed Wikipedia "a key tool for global public health promotion" [4, 5] and oth-

Compiled on: September 15, 2021.
Draft manuscript prepared by the author.

ers have found that in terms of content errors Wikipedia is on par with academic and professional sources even in fields like medicine [6]. Meanwhile, a metastudy of all the research about Wikipedia's health and medical content found it to be a prominent health information resource for experts and non-experts alike. [7]. With the WHO labeling the COVID-19 pandemic an "infodemic" [8], and disinformation threatening public health, a closer examination of Wikipedia and its references during the pandemic is merited. Wikipedia's "COVID-19 pandemic" article was among the most viewed in 2020 [9] - with a peak interest during the first wave. Researchers from different disciplines have looked into citations in Wikipedia and done bibliometric analyses of it - for example, asking if open-access papers are more likely to be cited in Wikipedia [10]. While anecdotal research has shown that Wikipedia and its academic references can mirror the growth of a scientific field [11], few have researched the coronavirus and Wikipedia. This research has shown both that traffic to Wikipedia's coronavirus articles reflected public interest in the pandemic [12], and that these articles provide a representative sample of COVID-19 research [13]. However, to our knowledge, no research has yet focused on the pandemic's "bibliometrics" on Wikipedia, and addressed the different dynamics regarding its sources - be they popular or academic - before and during the pandemic's first wave.

The aim of the present study is to provide a comprehensive bibliometric analyses of english Wikipedia's COVID-19 articles during this period. To characterize the scientific literature as well as general media sources supporting the encyclopedia's coverage of the COVID-19 as the pandemic spread we performed citation analyses of the references used in Wikipedia's coronavirus articles. We did this along three axes: the references used in the relevant articles at the end of the first wave, their historical trajectory, and their network interaction with Wikipedia articles on this topic.

Material and Methods

Using citations as a metric for gauging the scientificness of Wikipedia articles along these three axes allowed us to provide a "scientific score" (1) for them and ask: what shifts in scientificness did the COVID-19 articles undergo during the period researched. At the level of the citations inside any given Wikipedia article, we could provide a second metric, namely the latency (2) which allowed us to get a historical perspective on the scientific infrastructure supporting them. Moreover our work explored Wikipedia's articles' versions history and co-citations, to gain an insight on COVID-19 knowledge and its growth since the creation of the digital encyclopedia in 2001 and up until 2020. Though predominantly quantitative, for some selected articles we also examined the different claims the citations were used to support, at different stages, and reviewed some of the textual changes that articles underwent in wake of the coronavirus outbreak, to provide anecdotal qualitative context to our findings.

Corpus Delimitation

Digital Object Identifiers (DOIs) were used to identify academic sources among the references found within any given Wikipedia article. To delimit the corpus of Wikipedia COVID-19-articles containing DOIs, two different strategies were applied (Supplementary figure S1A). Every Wikipedia article affiliated with the official WikiProject COVID-19 task force (more than 1,500 pages during the period analyzed) was scraped using an R package specifically developed for this study, *WikiCitationHistoRy*. In combination with the *WikipediR* R package, which was used to retrieve the list of actual articles covered by the COVID-19 project, our *WikiCitationHistoRy* R package was used to extract DOIs from their text and thereby identified Wikipedia pages containing academic citations, termed "Wikipedia articles" in the present study. While "articles" is used for Wikipedia entries, "papers" is used to denote academic studies referenced on Wikipedia articles. Simultaneously, we also searched the EuroPMC database, using *COVID-19*, using *SARS-CoV2*, *SARS-nCoV19* as keywords to detect scientific studies published about this topic. Thus, 30,000 peer-reviewed papers, reviews, and preprint studies were retrieved. This set was compared to the DOI citations extracted from the entirety of the English Wikipedia dump of May 2020 (~860,000 DOIs) using *mwcite*. Thus, Wikipedia articles containing at least one DOI citation were identified - either from the EuroPMC search or through the specified Wikipedia project. The resulting "COVID-19 corpus" comprised a total of 231 Wikipedia articles - all related to COVID-19 and based on at least one academic source. In this study, the term "corpus" describes this body of Wikipedia "articles", and "sets" is used to describe "papers" and the bibliographic information relating to academic studies (i.e. DOIs).

DOI Corpus Content Analysis and DOI Sets Comparison

The analysis of DOIs led to the categorization of three DOI sets: 1) the COVID-19 Wikipedia set, 2) the EuroPMC 30K search and 3) the Wikipedia dump of May 2020. For the dump and the COVID sets, the latency was computed (to gauge how much time had passed from an academic paper's publication until it was cited on Wikipedia), and for all three sets we retrieved their scientific citations count (the number of times the paper was cited in scientific literature), their Altmetric score, as well as the papers' authors, publishers, journal, source type (preprint server or peer-reviewed publication), open-access status (if relevant), title and keywords. In addition, in the COVID-19 Wikipedia corpus the DOI set's citation count among wikipedia articles were also analysed to help gauge the importance of the sources.

Text Mining, Identifier Extraction and Annotation

From the COVID-19 corpus, DOIs, PMIDs, ISBNs, and URLs (Supplementary figure S1B) were extracted using a set of regular expressions from our R package. Moreover *WikiCitationHistoRy* allows the extraction of other sources such as tweets, press releases, reports, hyperlinks and the *protected* status of Wikipedia pages (on Wikipedia, pages can be locked to public editing through a system of "protected" statuses). Subsequently, several statistics were computed for each Wikipedia article and information for each of their DOI were retrieved using *Altmetrics* [14], *CrossRef* [15] and the *EuroPMC* [16] R packages.

Visualisations and Metrics

Our R package was developed in order to retrieve any Wikipedia article and its content, both in the present - i.e article text, size, reference count and users - and in the past - i.e. timestamps, revision IDs and the text of earlier versions. This package allows the retrieval of the relevant information in structured tables and helped support several visualisations for the data. Notably, two navigable visualisations were created and are available for any set of Wikipedia articles: 1) A timeline of article creation dates which allows users to navigate through the growth of Wikipedia articles related to a certain topic over time, and 2) a network linking Wikipedia articles based on their

shared academic references. The package also includes a proposed metric to assess the scientificness of a Wikipedia article. This metric, called *Sci Score* (shorthand for scientific score), is defined by the ratio of academic as opposed to non-academic references any Wikipedia article includes, as such:

$$Sci_{Score} = \frac{\#DOI}{\#Reference} \quad (1)$$

Our investigation also included an analysis of the latency [11] of any given DOI citation on Wikipedia. This metric is defined as the duration (in years) between the date of publication of a scientific paper and the date of introduction of the DOI into a specific Wikipedia article, as defined below:

$$Latency = Date_{WikiIntroduction} - Date_{Publication} \quad (2)$$

Data and Code Availability Statement

Every table and all of our raw data are available online through the ZENODO repository with DOI: 10.5281/zenodo.3901741 . Every visualisation and statistics were completed using R statistical programming language (R version 3.5.0). A beta version of the visualizations, their code and the documentation from our R package are available on the Github repositories:
https://github.com/jsobel1/WikiCitationHistoRy
https://github.com/jsobel1/Wiki_COVID-19_interactive_network
https://github.com/jsobel1/Interactive_timeline_wiki_COVID-19

Results

COVID-19 Wikipedia Articles: Well-Sourced but Highly Selective

We set out to characterize the representation of COVID-19-related research on Wikipedia. As all factual claims on Wikipedia must be supported by "verifiable sources" [17], we focused on articles' references to ask: What sources were used and what was the role of scientific papers in supporting coronavirus articles on Wikipedia? For this aim, we first identified the relevant Wikipedia articles related to COVID-19 (Supplementary figure S1A) as described in detail in the methods section. Then, we extracted relevant information such as identifiers (DOI, ISBN, PMID), references and hyperlinks (Supplementary figure S1B).

From the perspective of Wikipedia, though there were over 1.5K (1,695) COVID-19-related articles, only 149 had academic sources. We further identified an additional 82 Wikipedia articles that were not part of Wikipedia's organic set of coronavirus articles, but had at least one DOI reference from the EuroPMC database - which consisted of over 30,000 COVID-19 related papers (30,720) (Supplementary figureS1C). Together these 231 Wikipedia articles served as the main focus of our work as they form the scientific core of Wikipedia's COVID-19 coverage. This DOI-filtered COVID-19 corpus included articles on scientific concepts, genes, drugs and even notable people who fell ill with coronavirus. The articles ranged from "Severe acute respiratory syndrome-related coronavirus", "Coronavirus packaging signal" and "Acute respiratory distress syndrome", to "Charles, Prince of Wales", "COVID-19 pandemic in North America," and concepts with social interest like "Herd immunity", "Social distancing", "Wet market" or even public figures like "Dr. Anthony Fauci". This corpus included articles that were purely about scientific topics as well as those that had both scientific and social content and that were on topics with general interest to the public. For example, the article for "Coronavirus", the drugs "Chloroquine" and "Favipiravir," and other less scientific articles with wider social interest, like the article for "Social distancing" and "Shi Zhengli", the virologist employed by the Wuhan Institute of Virology and who earned public notoriety for her research into the origins of COVID-19.

Comparing the overall corpus of academic papers dealing with COVID-19 to those cited on Wikipedia we found that less than half a percent (0.42%) of all the academic papers related to coronavirus made it into Wikipedia (Supplementary figure S1C). Thus, our data reveals Wikipedia was highly selective in regards to the existing scientific output dealing with COVID-19 (See supplementary dataset (1)).

We next analyzed all the citations and references included in the complete Wikipedia dump from May 2020, using *mwcite*. Thus, we could extract a total number of about 2.68 million citations (2,686,881) comprising ISBNs, DOIs, arXiv, PMID and PMC numbers (Supplementary figure S1D). Among the citations extracted were 860K DOIs and about 38K preprints IDs from arXiv, about 1.4 percent of all the citations in the dump, indicating that the server hosting non-reviewed studies does contribute sources to Wikipedia alongside established peer-review journals. These DOIs were used as a separate group that was compared with the EuroPMC 30K DOIs (30,720) and the extracted DOIs (2,626 unique DOIs) from our initial Wikipedia COVID-19 set in a subsequent analysis.

An analysis of the journals and academic content from the 2,626 DOIs that were cited in the Wikipedia COVID-19 corpus reveals a strong bias towards high impact factor journals in both science and medicine. For example, *Nature* - which has an impact factor of over 42 - was among the top cited journals, alongside *Science*, *The Lancet* and the *New England Journal of Medicine*; together these four comprised 13 percent of the overall academic references (Figure 1A). The Cochrane database of systematic reviews was also among the most cited academic sources (WPM and Cochrane have an official partnership). Notably, the papers cited tended to not just to come from high impact factor journals, but also have a higher Altmetric score compared to the overall average of papers cited in Wikipedia in general. In other words, the papers cited on Wikipedia's COVID-19 articles were not just academically respected, but were also popular - i.e. they were shared extensively on social media such as Twitter and Facebook.

Most importantly perhaps, we also found that more than a third of the academic sources (39%) referenced in COVID-19 articles on Wikipedia were open-access papers (Figure 1B). The relation between open-access and paywalled academic sources is especially interesting when compared to Wikipedia's references writ large: About 29 percent of all academic sources on Wikipedia are open-access, compared to 63 percent in the COVID-19-related scientific literature (i.e. in EuroPMC).

Remarkably, despite a surge in COVID-19 research being uploaded to preprint servers, we found that only a fraction of this new output was cited on Wikipedia - less than 1 percent, or 27 (Figure 1C, Table S1) bioRxiv or medRxiv preprints were referenced. Among the preprints that were cited on Wikipedia was an early study on *Remdesivir* [18], a study on the mortality rate of elderly individuals [19], research on COVID-19 transmission in Spain [20] and New York [21], and research into how Wuhan's health system managed to eventually contain the virus [22], showing how non-peer-reviewed studies

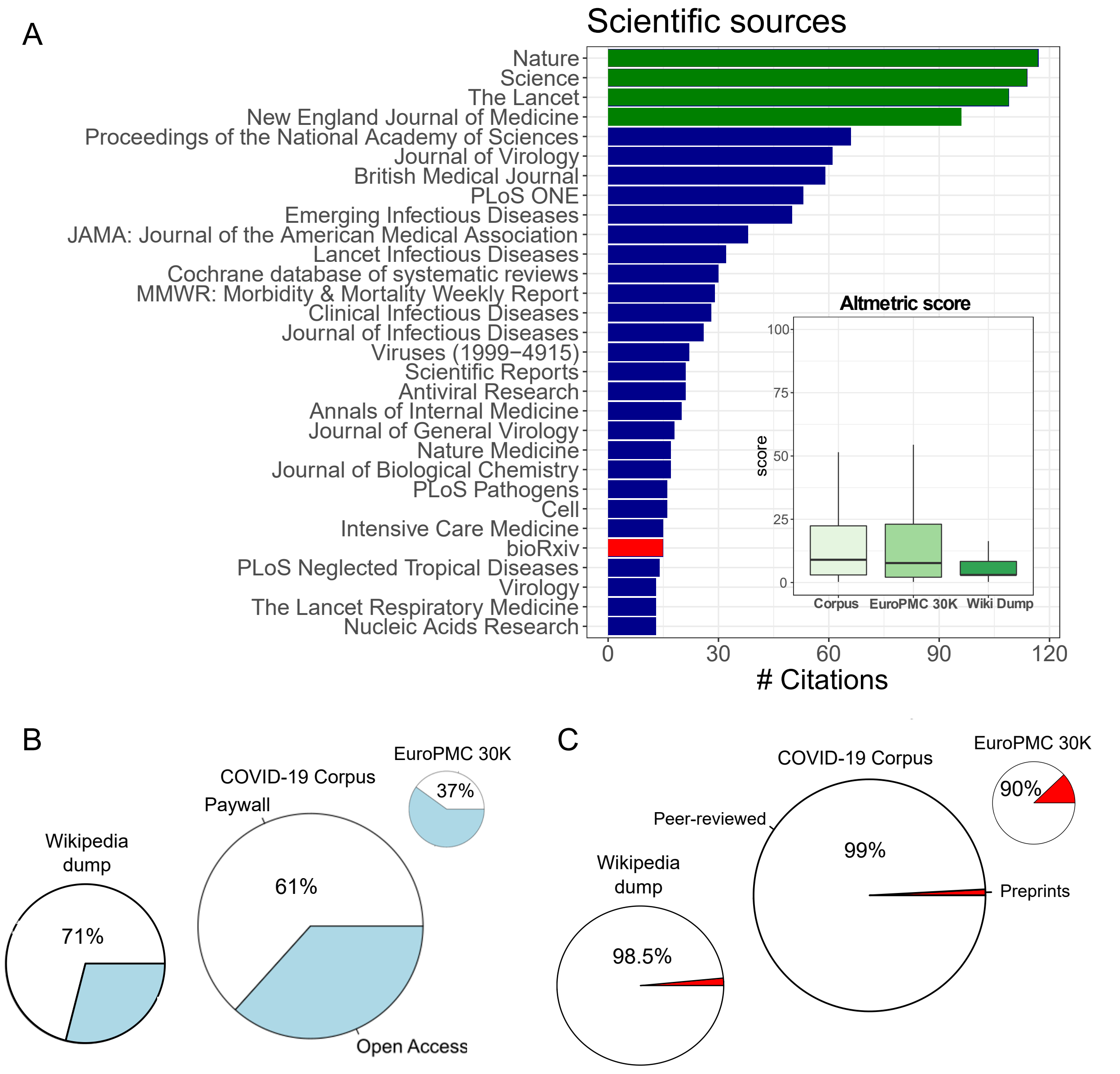

Figure 1. Wikipedia COVID-19 Corpus of scientific sources reveals a greater fraction of open-access papers as well as a higher impact in Altmetric score. A) Bar plot of the most trusted academic sources. Top journals are highlighted in green and preprints are represented in red. Bottom right: boxplot of the distribution Altmetrics score in Wikipedia COVID-19 corpus - the dump from May 2020, the COVID-19 Corpus and the scientific sources from the Europmc COVID-19 search. B) Fraction of open-access sources, C) fraction of preprints from bioRxiv and medRxiv.

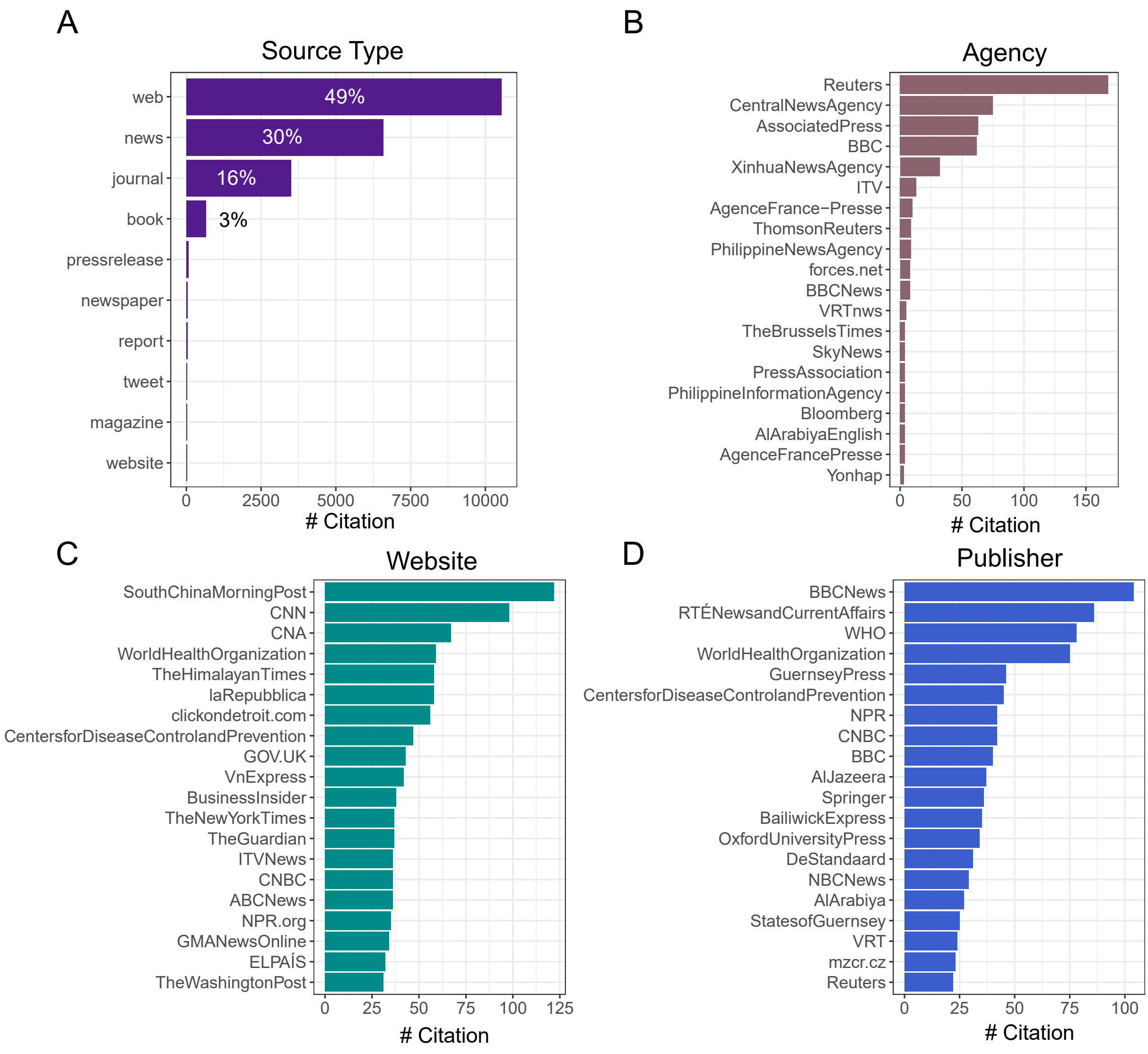

Figure 2. Wikipedia COVID-19 Corpus: Non-scientific sources mostly referred to websites or news media outlets considered highly respected and deemed to be trusted sources, including official sources like the WHO. A) source types extracted from the COVID-19 corpus of Wikipedia articles B) most cited news agencies, C) most cited websites and D) most cited publisher form the COVID-19 Corpus sources

touched on medical, health and social aspects of the virus. The later was especially prevalent with two of the preprints focusing on the benefits of contact tracing [23, 24]. The number of overall preprints was in line with the general representation of preprints in Wikipedia (1.5%), but lower than would be expected considering the fact that our academic database of EuroPMC papers had almost 3,700 preprints - 12.3 percent of the roughly 30,000 COVID-19 related papers in May 2020. Thus, in contrast to the high enrichment of preprints in COVID-19 research, Wikipedia's editors overwhelmingly preferred peer-reviewed papers to preprints. In other words, Wikipedia generally cites preprints more than it was found to on the topic of COVID-19, while COVID-19 articles cited open-access paper by more 10% (from 29% to 39%). Taken together with the bias towards high-impact journals, our data suggest that this contributed significantly to Wikipedia's ability to stay both up to date and to maintain high academic standards, allowing editors to cite peer-reviewed research despite other alternatives being available.

Due to the high selectivity of Wikipedia editors in terms of the percentage of COVID-19 academic research actually cited on Wikipedia's COVID-19 articles, we also focused on non-academic sources. Popular media, we found, played a substantial role in our corpus. Over 80 percent of all the references used in the COVID-19 corpus were non-academic, being either general media or websites (Figure 2A). In fact, a mere 16 percent of the over 21,000 references supporting the COVID-19 content were from academic journals. Among the general media sources used (Figure 2B-D), there was a high representation for what is termed legacy media outlets, like the *New York Times* and the *BBC*, alongside widely syndicated news agencies like *Reuters* and the *Associated Press*, and official sources like *WHO.org* and *gov.UK*. Among the most cited websites, for example, there was an interesting representation of local media outlets from countries hit early and hard by the virus, with the Italian *La Republica* and the Chinese *South China Post* being among the most cited sites. The World Health Organization was one of the most cited publisher in the corpus of relevant articles, more than 150 references.

Scientific Score

To distinguish between the role scientific research and popular media played, we created a “scientific score” for Wikipedia articles (1). The metric is based on the ratio of academic as opposed to non-academic references any article includes. This score attempts to rank the *scientificness* of any given Wikipedia article based solely on its list of references. Ranging from 1 to 0, an article’s scientific score is calculated according to the ratio of its sources that are academic (i.e. contain DOIs), so that an article with a score of 1 will have 100 percent academic references, while that with none will have a score of zero. Technically, as all of our corpus of coronavirus-related Wikipedia articles had at least one academic source in the form of a DOI, their scientific scores will always be greater than zero (Supplementary Figure S2, Supplementary Figure S5C).

In effect, this score puts forth a metric for gauging the prominence of academic texts in any given article’s reference list - or lack thereof. Out of our 231 Wikipedia articles, 15 received a perfect scientific score of 1 (Supplementary Figure S2A). High scientific score Wikipedia articles included the articles for the enzymes of “Furin” and “TMPRSS2” - whose inhibitor has been proposed as a possible treatment for COVID-19; “C30 Endopeptidase” - a group of enzymes also known as the “SARS coronavirus main proteinase”; and "SHC014-CoV" - a form of COVID-19 that affects the Chinese rufous horseshoe bat.

In contrast to the articles with scientific topics and even biographical articles about scientists themselves, which both had high scientific scores, those with the lowest scores (Supplementary Figure S2B) seemed to focus almost exclusively on social aspects of the pandemic and its immediate outcome. For example, the articles with the lowest scores dealt directly with the pandemic in a hyper-local context, including articles about the pandemic in Canada, North America, Indonesia, Japan or even Jersey, to name a few. Others focused on different aspects of the pandemic, for example the "Impact of the COVID-19 pandemic on the arts and cultural heritage" or "Travel restrictions related to the COVID-19 pandemic". One of the articles with the lowest scientific score was the "Trump administration communication during the COVID-19 pandemic" which made scarce use of coronavirus-related research to inform its content, citing a single academic paper (related to laws regulating quarantine) among its 244 footnotes.

The Price of Remaining Up to Date on COVID-19

During the pandemic, there were over tens of thousands of edits to the site, with thousands of new articles being created and scores of existing ones being re-edited and recast in wake of new developments. Therefore, one could expect a rapid growth of articles on the topic, as well as a possible overall increase in the number of citations of all kinds. We sought to explore the temporal axis of Wikipedia’s coverage of the pandemic to see how coverage of COVID-19 developed, namely, what were the dynamics of the growth of COVID-19 articles and their academic references.

First, we laid out our corpus of 231 articles across a timeline according to each article’s respective date of creation (Supplementary Figure S3). An article count starting from 2001, when Wikipedia was first launched, and up until May 2020, shows that for many years there was a relatively steady growth in the number of articles that would become part of our corpus - until the pandemic hit, causing a massive peak at the start of 2020 (Figure 3A). As the pandemic spread, the total number of Wikipedia articles dealing with COVID-19 and supported by scientific literature almost doubled - with a comparable number of articles being created after 2020 than the entire time before (Figure 3A, Supplementary FigureS3) (from 134 before 2020 compared to 97 in 2020).

The majority of the pre-2020 articles were created relatively early - between 2003 and 2006, likely linked to a general uptick in creation of articles on Wikipedia during this period. For example, the article for (the non-novel) “coronavirus” has existed since 2003, the article for the medical term “Transmission” and that of “Mathematical modeling of infectious diseases” from 2004, and the article for the “Coronaviridae” classification from 2005. Articles opened in this early period tended to focus on scientific concepts - for example those noted above or others like "Herd immunity". Conversely, the articles created post-pandemic during 2020 tended to be hyper-local or hyper-focused on the virus’ effects. Therefore, we collectively term the first group Wikipedia’s "scientific infrastructure", as they allowed new scientific information to be added into existing articles, alongside the creation of new ones focusing on the pandemic’s actual ramifications.

Examining the date of publication of the peer-reviewed studies referenced on Wikipedia shows that new COVID-19 research was cited alongside papers from previous years and even the previous century, the oldest being a 1923 paper titled the “The Spread of Bacterial Infection. The Problem of Herd-Immunity.” [25]. Overall, among the papers referenced on Wikipedia were highly cited studies, some with thousands of citations (Table 3), but most had relatively low citation counts (median of citation count for a paper in the corpus was 5). Comparing between a paper’s date of publication and its citation count reveals there is low anti-correlation (-0.2) but highly significant between the two (Pearson’s product-moment correlation test p-value $< 10^{-15}$, Figure S5A). This suggest that on average older scientific papers have a higher citation count; unsurprisingly, the more time that has passed since publication, the bigger the chances a paper will be cited.

The pre-pandemic articles tended to have a high scientific score - for example, “Chloroquine”, which has been examined as a possible treatment for COVID-19 - but also underwent a shift in content in wake of the pandemic, seeing both a surge in traffic and a surge in editorial activity (Supplementary Figure S4). However, per a subjective reading of this article’s content and the editing it underwent during this period, much of the scientific content that was present pre-pandemic remained intact, with new coronavirus-related information being integrated into the existing content. The same occurred with many social concepts retroactively affiliated with COVID-19. Among these we can note the articles for “Herd immunity”, “Social distancing” and the “SARS conspiracy theory” that also existed prior to the outbreak and served as part of Wikipedia’s scientific infrastructure, allowing new information to be contextualized.

In addition to the dramatic rise in article creation during the pandemic, there was also a rise in the overall number of references affiliated with COVID-19 articles on Wikipedia (Figure 3B). In fact, the number of added DOIs in our articles grew almost six-fold post-2020 - from roughly 250 to almost 1,500 citations. Though most of the citations added were not just academic ones, with URLs overshadowing DOIs as the leading type of citation added, the general rise in citations can be seen as indicative of scientific literature’s prominent role in COVID-19 when taking into account that general trend in Wikipedia: The growth rate of references on COVID-19 articles was generally

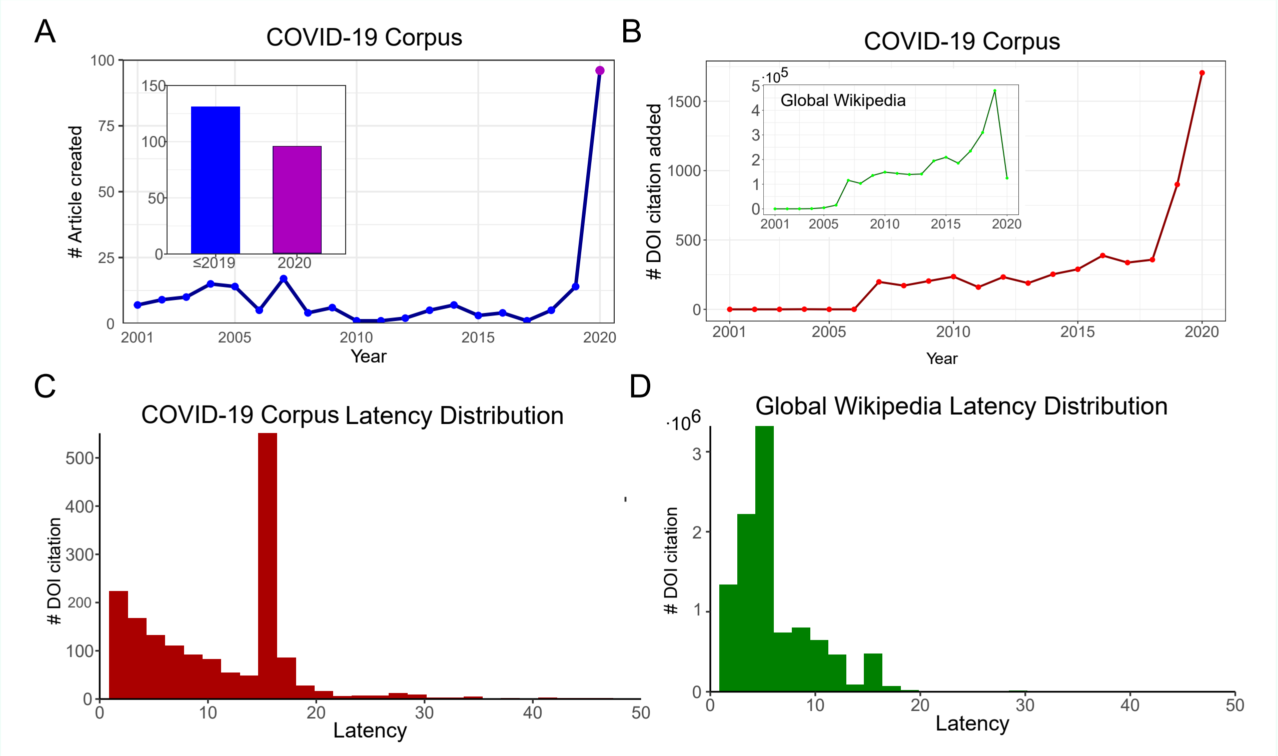

Figure 3. Historical perspective of the Wikipedia COVID-19 corpus outlining the growth of COVID-19 on the encyclopedia. A) COVID-19 article creation per year and number of articles created before the pandemic compared to the first five months of the pandemic. B) Scientific citation added per year in the COVID-19 category and globally in Wikipedia. C) Latency distribution of scientific literature in the COVID-19 corpus and D) latency distribution of scientific literature in the Wikipedia dump. See Supplementary FigureS3 and here for an interactive version of the timeline.

static until the outbreak; but on Wikipedia writ large references were on a rise since 2006. The post-2020 surge in citations was both academic and non-academic (Supplementary Figure S5B).

One could hypothesize that a rapid growth in the number articles dedicated to coronavirus would translate to an overall decrease in the presence of academic sources, as Wikipedia can create newer articles faster than academic research can be published on current events. Comparing the pre- and post-2020 articles' scientific score reveals that on average, the new articles had a mean score of 0.14, compared to the pre-2020 group's mean of 0.48 and the overall average of 0.3 (Supplementary Figure S5C). Reading the titles of the 2020 articles to glean their topic and reviewing their respective scientific score can also point to a generalization: the more scientific an article is in topic, the more scientific its references are - even during the pandemic. This means that despite the dilution at a general level during the first month of 2020, articles with scientific topics created during this period did not pay that heavy of an academic price to stay up to date.

How is that Wikipedia managed to maintain academic sourcing on new and old articles about coronavirus as the pandemic was happening? One possible explanation is that among the academic papers added to Wikipedia in 2020 were also papers published prior to this year if not a long time before. To investigate this hypothesis we used the latency metric (2). We found the mean latency of Wikipedia's COVID-19 content to be 10.2 years (Figure 3C), slower than Wikipedia's overall mean of 8.7 (Figure 3D). In fact, in the coronavirus corpus we observed a peak in latency of ~17 years - with over 500 citations being added to Wikipedia 17 years after their initial academic publication - almost twice as slow as Wikipedia's average. Interestingly, this time frame corresponds to the SARS outbreak (SARS-CoV-1) in 2002-2004, which yielded a boost of scientific literature regarding coronaviruses. This suggests that while there was a surge in editing activity during this pandemic that saw papers published in 2020 added to the COVID-19 articles, a large and even prominent role was still permitted for older literature. Viewed in this light, older papers played a similar role to pre-pandemic articles, giving precedence to existing knowledge in ordering the integration new knowledge on scientific topics.

Comparing the articles' scientific score to their date of creation portrays Wikipedia's scientific infrastructure and its dynamics during the pandemic (Supplementary Figure S5C). It reveals that despite maintaining high academic standards, citing papers published in prestigious and high impact factor journals, the need to stay up to date with COVID-19 research did come at some cost: most of the highest scoring articles were ones created pre-pandemic (mostly during 2005-2010) and newer articles had a lower scientific score (Supplementary Figure S5C).

Networks of COVID-19 Knowledge

To further investigate Wikipedia's scientific sources and its infrastructure, we built a network of Wikipedia articles linked together based on their shared academic (DOI) sources. We filtered the list of papers (extracted DOIs) in order to keep those which were cited in at least two different Wikipedia articles, and found 179 that fulfilled this criteria, mapped to 136 Wikipedia articles in 454 different links (Figure 4, supplementary data (2)). This allowed us to map how scientific knowledge related to COVID-19 played a role not just in specific articles created during or prior to the pandemic, but actually formed a web of knowledge that proved to be an inte-

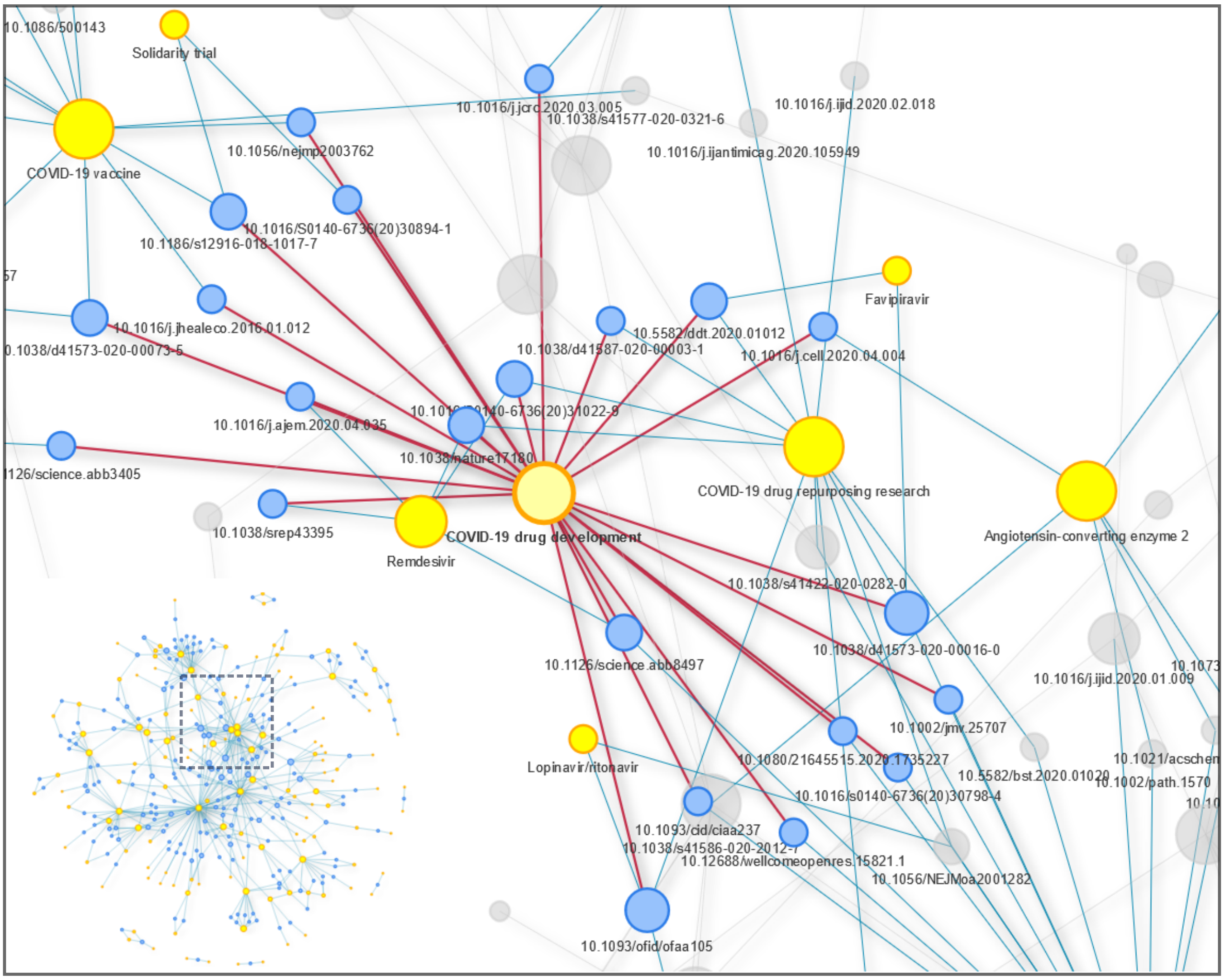

Figure 4. Wikipedia COVID-19 corpus article-scientific papers (DOI) network. The network mapping scientific papers cited in more than one article in the Wikipedia COVID-19 corpus was constructed using each DOI connecting at least two Wikipedia articles. This network is composed of 454 edges, 179 DOIs (Blue) and 136 Wikipedia articles (Yellow). A zoom in on the cluster of Wikipedia articles dealing with COVID-19 drug development is depicted with edges in red connecting the DOIs cited directly in the article and edges in blue connecting these DOIs to closely related articles citing the same DOIs. See here for an interactive version of the network. See Supplementary dataset (2).

gral part of Wikipedia's scientific infrastructure. Similar to the timeline described earlier and as a subset of our COVID-19 corpus, Wikipedia articles belonging to this network included those dealing with people, institutions, regional outcomes of the pandemic as well as scientific concepts, for example those regarding the molecular structure of the virus or the mechanism of infection ("C30 Endopeptidase", "Coronaviridae", and "Airborne disease"). It also included a number of articles regarding the search for a potential drug to combat the virus or other possible interventions against it (articles on topics like social distancing, vaccine development and drugs in current clinical trials).

Interestingly, we observed six prominent Wikipedia articles emerge in this network. These shared multiple citations with many other pages through DOI connections (nodes with an elevated degree). Four of these six so-called major nodes had a distinct and broad topic: "Coronavirus," which focused on the virus writ large; "Coronavirus disease 2019", which focused on the pandemic; and "COVID-19 drug repurposing research" and "COVID-19 drug development." The first two articles were key players in how Wikipedia presented its coverage of the pandemic to readers: both were linked to from the main coronavirus article ("COVID-19 pandemic") which was placed on the English Wikipedia's homepage in a community-led process known as "In the News" which showcases select articles on relevant topics on the website's homepage. Later on, alongside this community process led by the volunteers of the WikiProject COVID-19 task force, the WikiMedia Foundation also issued a directive to place a special banner referring to the "COVID-19 pandemic" article on the top of every single article in English, driving millions to the article and to subsequent articles linking out from it. These, too, were part of our network, showing how citations shared between articles can also coincide with inter-connectivity between the articles themselves.

The two remaining nodes were similar and did not prove to be distinctly independent concepts, but rather interrelated ones, with the articles for "Severe acute respiratory syndrome–related coronavirus" and "Severe acute respiratory syndrome coronavirus" each appearing as their own node despite their thematic connection. It is also interesting to note that four of the six Wikipedia articles that served as the respective centers of these groups of nodes were locked to public editing as part of the protected page status (see supplementary data (3)) and these were all articles linked to the WikiProject Medicine or, at a later stage, to the specific offshoot project set up to deal with COVID-19.

Two main themes that emerge from the network is that of COVID-19 related drugs and of the disease itself (Figure 4). Unlike popular articles relating to the effect of the virus, which we have seen are predominantly based on popular media, with scientific media playing a relatively small role, these two were topics that did require scientific basing to be able to be reliable according to the MEDRS policy - shorthand for "medical reliable sources, the sourcing policy is Wikipedia's most rigit and bans primary sources and instead demands meta-analysis or secondary sources that provide an overview of existing research and multiple-case-study clinical trials [26]. The prominence of articles like "Coronavirus disease 2019" or "COVID-19 drug development" - both of which were locked (supplementary dataset (3)) and fell under the auspices of the COVID-19 task force - in our network underscore the role academic media had in their references. Furthermore, it highlights the effects of the editing community's centralized efforts: for example, by allowing key studies to find a role both in popular articles reached from the main articles and in scientific articles linking out from them, thus creating the network we describe.

In our network analysis, an additional smaller group of nodes (with a lower degree) was also found. It had to do almost exclusively with China-related issues. As such, it exemplified how Wikipedia's sourcing policy - which has an explicit bias towards peer-reviewed studies and is enforced exclusively by the community - helps fight disinformation. For example, the academic paper that was most cited in Wikipedia's COVID-19 articles was a paper published in Nature in 2020, titled "A pneumonia outbreak associated with a new coronavirus of probable bat origin" (Table S2). This paper was referenced in eight different Wikipedia articles, two among which dealt directly with scientific topics - "Angiotensin-converting enzyme 2" and "Severe acute respiratory syndrome coronavirus 2" - and two dealing with what can be termed para-scientific terms linked to COVID-19 - the "Wuhan Institute of Virology" and "Shi Zhengli". This serves to highlight how contentious issues with a wide interest for the public - in this case, the origin of the virus - receive increased scientific support on Wikipedia, perhaps as result of editors attempting to fend off misinformation supported by lesser, non-academic sources - specifically media sources from China itself, which as we have seen were present on Wikipedia. Of the five most cited papers inside the COVID-19 corpus (Table S2) three focused specifically on either bats or the virus' animal origins, and another focused on its spread from Wuhan, China. Interestingly, one of the 27 preprints cited (Table S1) was also the first study to suggest the virus' origin lay with bats was [27].

Taken together with the previous findings, centralized efforts in the form of locking articles did not just allow the enforcement of a rigid sourcing policy but also created a filtered knowledge funnel of sorts, which harnessed Wikipedia' preexisting infrastructure of articles, mechanisms and policies to allow a regulated intake of new information as well as the creation of new articles, both based on existing research.

Discussion

In the wake of COVID-19 pandemic, characterizing scientific research on English-language Wikipedia and understanding the role it plays is both important and timely. Millions of people - both medical professionals and the general public - read about health online [1]. Research has shown traffic to Wikipedia articles follows topics covered in the news [28] - a dynamic which played out during the pandemic's first wave [12]. Moreover, scientometric research has shown that academic research follows a similar pattern - with a surge of new studies during a pandemic and then a decrease after it wanes [29]. During a pandemic, as was during the Zika and SARS outbreaks [30], the risk of disinformation on Wikipedia's content is more severe. Thus, throughout the outbreak of the COVID-19 pandemic, the threat was hypothetically increased: as a surge in traffic to Wikipedia articles, research has found, often translates into an increase in vandalism [31]. Moreover, research into medical content on Wikipedia found that people who read health articles on the open encyclopedia are more likely to hover over, or even read its references to learn more about the topic [32]. Particularly in the case of the coronavirus outbreak, Wikipedia's role as such took on potentially lethal consequences as the pandemic was deemed to be an *infodemic*, and false information related to the virus was deemed a real threat to public health by the UN and WHO [8]. So far, most research into Wikipedia has revolved either around the quality, readership or editorship of health content on Wikipedia - or about references and sourcing in general. Meanwhile, research on Wikipedia and

COVID-19 has focused almost exclusively on editing patterns and users behaviors [12], or the representativity of academic citations [13]. Therefore, we deployed a comprehensive bibliometric analyses of COVID-19-related Wikipedia articles – focusing on article's text and source, their growth over time and their network relations.

Perhaps counter-intuitively, we found that despite the traffic surge, these articles relied on high quality sources, from both popular media and academic literature. Though the proportion of academic references in newly created articles did decrease in comparison to the period before the pandemic (resulting in lower scientific score), we found that they still played a prominent role and that high editorial standards were generally maintained, utilizing several unique solutions which we will now attempt to outline and discuss based on our findings.

One possible key to Wikipedia's success had to do with the existence of centralized oversight mechanisms by the community of editors that could be quickly and efficiently deployed. In this case, the existence of the WikiProject Medicine – one of Wikipedia's oldest community projects – and the formation of a specific COVID-19 task force in the form of WikiProject COVID-19, helped harness exiting editors and practices like locking articles to safeguard quality across large swaths of articles and thus enforce a relatively unified sourcing policy on articles dealing with both popular and scientific aspects of the virus.

In general, all factual claims on Wikipedia need to be supported by a verifiable source. Specifically, biomedical articles affiliated with the WikiProject Medicine (WPM) are bound by a specific policy known as MEDRS (which requires meta-analysis or secondary sources for medical content [26]). However, the mere existence of this policy does not necessarily mean it is respected. However, our findings indicate that this policy, aided by the infrastructure provided by the community to enforce it, likely played a key role in regulating the quality of coronavirus articles. One mechanism used generally by the WPM to enforce the MEDRS sourcing standards and specifically deployed by the COVID-19 task force during the pandemic was locking articles to public editing (protected pages, supplementary dataset (3)). This is a technique that is used to prevent vandalism on Wikipedia [33] and is commonly used when news events drive large amounts of new readers to specific Wikipedia articles, increasing the risk of substandard sources being added into the article by editors unversed in Wikipedia's standards. This ad hoc measure of locking an article, deployed by a community vote on specific articles for specific amounts of time, prevents anonymous editors from being able to contribute directly to an article's text and forces them to work through an experienced editor, thus ensuring editorial scrutiny. This measure is in line with our findings that many of the COVID-19 network central nodes were locked articles.

Another possible key to Wikipedia's ability to maintain the WPM's MEDRS policy of high quality sources during the pandemic was the existence of a specific infrastructure related directly to sourcing. The WPM has formed institutional-level partnerships to provide editors with access to reputable secondary sources on medical and health topics – namely through its cooperation with the Cochrane Library. The Cochran Reviews' database is available to Wikipedia's medical editors and it offers them access to systematic literature reviews and meta-analyses summarizing the results of multiple medical research studies[34]. As well as the existence of this database on medical content, the practice of providing access to high-quality sources was also deployed specifically in regards to coronavirus in the form of the task force's list of "trusted" sources. Alongside Cochrane studies, the WHO, for example, was given special status and preference [35]. This was evident in our results as the WHO was among the most cited publishers on the COVID-19 articles. Also among the most cited scientific sources were others that were promoted by the task force as preferable sourcing for scientific content: for example, *Science*, *Nature* and *The Lancet*. This indicates that list of sources recommended by the task force were actually utilized by the volunteers and thus underscores the connection between our findings and the existence of a centralized community effort.

This was also true for non-academic sources: Among general media sources that the task force endorsed were *Reuters* and the *New York Times*, which were also prominently represented in our findings. As each new edit to any locked COVID-19 article needed to be vetted by an experienced volunteer from the task force before it could go online within the body of an article's text, the influx of new information being added was slowed down and regulated; the source list thus allowed an especially strict sourcing policy to be rigorously implemented across thousands of articles. This was true despite the fact that there is no academic verification for volunteers – in fact, research suggests that less than half of Wikipedia's editors focused on health and medical issues are medical professionals [3, 4] – meaning that the task forces and its list of sources allowed non-experts to enforce academic-level standards.

This dynamic was also evident within articles with purely scientific content. Despite a deluge of preprints (both in general in recent years and specifically during the pandemic [36, 37]), in our analysis, non-peer-reviewed academic sources did not play a key role on Wikipedia's coronavirus content, while open access papers did. Therefore, one could speculate that our finding that open-access papers were disproportionately cited may provide an explanation – with academic quality trumping speed, and editors opting against preprints and preferring published studies instead. Previous research has found open-access papers are more likely to be cited on Wikipedia by 47 percent [10] and nearly one-third of the Wikipedia citations link to an open-access source [38]. Here we also saw that open-access was prevalent in Wikipedia and even more so on COVID-19 articles. This, we suggest, allowed Wikipedia's editors (expert or otherwise) to keep articles up to date without reverting to non-peer-reviewed academic content. This, one could suggest, was likely facilitated or at least aided by the decision by academic publications' like *Nature* and *Science* to lift paywall and open public access to all of their COVID-19-related research papers, both past and present.

In addition to the communal infrastructure's ability to regulate the addition of new information and maintain quality standards over time, another facet we found to contribute in permitting Wikipedia to stay accurate during the pandemic is what we term its scientific infrastructure. Research on Wikipedia articles' content has shown that the initial structuring of information on a given article tends to dictate its development in later stages, and that substantial reorganizations gradually decrease over time [39]. A temporal review of our articles and their citations, showed that the best-sourced articles, those with the highest scientific score that formed the scientific backbone of Wikipedia's COVID-19 content, were those created from 2005 and until 2010. These, we argue, are part of Wikipedia's wider scientific infrastructure, which regulated the intake of new knowledge into Wikipedia.

Our network analysis reflects the pivotal role preexisting content played in contextualizing the science behind many popular concepts or those made popular by the pandemic. Preexist-

ing content in the form of Wikipedia articles, policies, practices, and academic research served as a framework that helped regulate the deluge of new information, allowing newer findings to find a place within Wikipedia's existing network of knowledge. Future work on this topic could focus on the question of whether this dynamic changed as 2020 progressed and, at a later time, on how contemporary peer-reviewed COVID-19-related research that was published during the pandemic's next waves would be integrated into these articles.

Previous research has suggested that in terms of content errors Wikipedia is on par with academic and professional sources even in fields like medicine [6]. A recent meta-analysis of studies about medical content on Wikipedia found that despite the prominent role Wikipedia plays for the general public, health practitioners, patients and medical students, the academic discourse around Wikipedia within the context of health is still limited [7]. This indicates that academic publications and scientists are lagging on embracing it and its benefits. Such a process could help improve Wikipedia's content and even introduce new editors with academic background into the fold, which would further improve quality and and timeliness.

Moreover, our findings suggest that "open" science - not just open access - may be key to understanding Wikipedia's mechanisms and how they can be translated to other contexts. In this regard, much like citizen scientists help support institutional science [40], Wikipedia's editors may be regarded as citizen encyclopedists [11]. Viewed as such, Wikipedia's citizen encyclopedists can play the same role communicating science that citizen scientists play in creating science. However, as previous citizen science projects have taught us [41], for that to work, citizens need scientists to provide the framework for non-expert contributions [42, 43]. As this study shows, a similar infrastructure can be seen to exist on Wikipedia for encyclopedic as opposed to scientific work. Thus, should the cooperation between the scientific and Wikipedia communities increase, it could be utilized for other contexts as well.

Our findings outline ways in which Wikipedia managed to fend off disinformation and stay up to date. With Facebook and other social media giants struggling to implement both technical and human-driven solutions to disinformation from the top down, it seems Wikipedia's dual usage of established science and a community of volunteers, provides a possible model for how this can be achieved - a valuable goal during an infodemic. Some have already suggested that the American Center for Disease Control should adopt Wikipedia's model to help communicate medical knowledge [44]. In October 2020, the WHO and Wikimedia, the foundation that oversees the Wikipedia project, announced they would cooperate to make critical public health information available. This means that in the near future, the quality of Wikipedia's coverage of the pandemic will very likely increase just as its role as central node in the network of knowledge transference to the general public becomes increasingly clear.

Wikipedia's main advantage is in many ways its largest disadvantage: its open format which allows a large community of editors of varying degrees of expertise to contribute. This can lead to large discrepancies in article quality and inconsistencies in the ways editors add references to articles' text [38]. We tried to address these limitations using technical solutions, such as regular expressions for extracting URLs, hyprelinks, DOIs and PMIDs. In this study, which was limited to English, we retrieved most of our scientific literature metadata using Altmetrics [45, 14], EuroPMC [16] and CrossRef [15] R APIs. However the content of the underlying databases is not always accurate, and at a technical level, this method was not without limitations. For example, we could not retrieve all of the extracted DOIs' metadata. Moreover, information regarding open access (among others) varied with quality between the APIs [46]. In addition, our preprint analysis was mainly focused on MedRxiv and BioRxiv which have the benefit of having a distinct DOI prefix. Unfortunately, we found no better solution to annotate preprints from the extracted DOIs. Preprint servers do not necessarily use the DOI system [47] (i.e ArXiv) and others share DOI prefixes with published paper (for instance the preprint server used by The Lancet). Moreover, we developed a parser for general citations (news outlets, websites, publishers), and we could not avoid redundant entries (i.e "WHO", "World Health Organisation").

In addition, our method to delimitate the COVID-19 corpus focused on medical content (EuroPMC search) and may explain why we found predominately biomedical and health studies. However, using DOI filtering on Wikipedia's coronavirus articles should have equally led us to find studies from the social sciences - should those have been used. However, it seems that as these socially focused articles do not fall under the MEDRS sourcing policy, there was less if any use of academic studies, resulting in a low scientific score, thus highlighting the importance of this policy in enforcing academic standards on the open encyclopedia's articles.

Finally, as Wikipedia is constantly changing, some of our conclusions are bound to change. Therefore, our study, though limited, is focused on the pandemic's first wave and its history on English Wikipedia alone, a crucial arena for examining the dynamics of knowledge online at this pivotal time frame. As these findings regarding the first wave were the result of a robust community effort that utilized English Wikipedia's policies and mechanisms to safeguard existing content and regulate the creation of new content, it may be specific to English Wikipedia and its community.

However, it seems safe to speculate that at least on English Wikipedia the processes will continue to take place in the future as new textual additions are made to the open encyclopedia. In fact, one could suggest that as more time passes from the first wave, the newer post-pandemic articles that had low scientific scores will undergo a review and have their sources improved as newer research becomes more readily available. Studying the second wave - for example, shifts in the scientific score overtime - and understanding how encyclopedic content written during the first wave changed over the next year could very instructive. Analyses of coronavirus articles indicated that at least on medical and health topics - especially those in the news and driving public interest - Wikipedia's methods for safeguarding its standards withstand the test. Perhaps as more academic research regarding the virus passes review and is published in 2021 and in the coming years, the ability of Wikipedia to reduce latency on this topic without having to compromise its scientificness will increase. Moreover, our findings hint that should journals open access to research in other fields, it may help Wikipedia cite even more peer reviewed research instead of media sources or preprints. Thus, with the help of community enforcement, like that seen during the first wave of the pandemic, Wikipedia should be able to succeed in other fields as well.

In summary, our findings reveal a trade off between timeliness and scientificness in regards to scientific literature: most of Wikipedia's COVID-19 content was supported by references from highly trusted sources - but more from the general media than from academic publications. That Wikipedia's COVID-19 articles were based on respected sources in both the academic and popular media was found to be true even as the pandemic

and number of articles about it grew. Our investigation further demonstrates that despite a surge in preprints about the virus and their promise of cutting-edge information, Wikipedia preferred published studies, giving a clear preference to open-access studies. A temporal and network analysis of COVID-19 articles indicated that remaining up-to-date did come at a cost in terms of quality, but also showed how preexisting content helped regulate the flow of new information into existing articles. In future work, we hope the tools and methods developed here in regards to the first wave of the pandemic will be used to examine how these same articles fared over the entire span of 2020, as well as helping others use them for research into other topics on Wikipedia. We observed how Wikipedia used volunteer-editors to enforce a rigid sourcing standards - and future work may continue to provide insight into how this unique method can be used to fight disinformation and to characterize the knowledge infrastructure in other arenas.

Acknowledgments

J.S. is a recipient of the Placide Nicod foundation, and R.A. is a recipient of the Azrieli Foundation fellowship. We are grateful for their financial support.

References

1. Heilman JM, West AG. Wikipedia and medicine: quantifying readership, editors, and the significance of natural language. Journal of medical Internet research 2015;17(3):e62.
2. Lavsa SM, Corman SL, Culley CM, Pummer TL. Reliability of Wikipedia as a medication information source for pharmacy students. Currents in Pharmacy Teaching and Learning 2011;3(2):154–158.
3. Allahwala UK, Nadkarni A, Sebaratnam DF. Wikipedia use amongst medical students–new insights into the digital revolution. Medical teacher 2013;35(4):337–337.
4. Heilman JM, Kemmann E, Bonert M, Chatterjee A, Ragar B, Beards GM, et al. Wikipedia: a key tool for global public health promotion. Journal of medical Internet research 2011;13(1):e14.
5. Herbert VG, Frings A, Rehatschek H, Richard G, Leithner A. Wikipedia–challenges and new horizons in enhancing medical education. BMC medical education 2015;15(1):32.
6. Jemielniak D. Wikipedia: Why is the common knowledge resource still neglected by academics? GigaScience 2019;8(12):giz139.
7. Smith DA. Situating Wikipedia as a health information resource in various contexts: A scoping review. PloS one 2020;15(2):e0228786.
8. WHO, Novel Coronavirus (2019-nCoV): situation report, 13. World Health Organization (WHO); 2020.
9. Wikipedia, Wikipedia:2020 Top 50 Report; 2020. https://en.wikipedia.org/wiki/Wikipedia:2020_Top_50_Report.
10. Teplitskiy M, Lu G, Duede E. Amplifying the impact of open access: Wikipedia and the diffusion of science. Journal of the Association for Information Science and Technology 2017;68(9):2116–2127.
11. Benjakob O, Aviram R. A Clockwork Wikipedia: From a Broad Perspective to a Case Study. Journal of Biological Rhythms 2018;33(3):233–244.
12. Chrzanowski J, Sołek J, Jemielniak D. Assessing Public Interest Based on Wikipedia's Most Visited Medical Articles During the SARS-CoV-2 Outbreak: Search Trends Analysis. Journal of medical Internet research 2021;23(4):e26331.
13. Colavizza G. COVID-19 research in Wikipedia. Quantitative Science Studies 2020;p. 1–32.
14. Ram K. rAltmetric: Retrieves altmerics data for any published paper from altmetrics.com; 2012, http://CRAN.R-project.org/package=rAltmetric, r package version 0.3.
15. Lammey R. Using the Crossref Metadata API to explore publisher content. Sci Ed 2016;3(3):109–11.
16. Levchenko M, Gou Y, Graef F, Hamelers A, Huang Z, Ide-Smith M, et al. Europe PMC in 2017. Nucleic acids research 2018;46(D1):D1254–D1260.
17. Wikipedia, Wikipedia:Core content policies; 2020. https://en.wikipedia.org/wiki/Wikipedia:Core_content_policies.
18. Williamson BN, Feldmann F, Schwarz B, Meade-White K, Porter DP, Schulz J, et al. Clinical benefit of remdesivir in rhesus macaques infected with SARS-CoV-2. BioRxiv 2020;.
19. Ioannidis JP, Axfors C, Contopoulos-Ioannidis DG. Population-level COVID-19 mortality risk for non-elderly individuals overall and for non-elderly individuals without underlying diseases in pandemic epicenters. medRxiv 2020;.
20. Fuertes FD, Caballero MI, Monzón S, Jiménez P, Varona S, Cuesta I, et al. Phylodynamics of SARS-CoV-2 transmission in Spain. bioRxiv 2020;.
21. Gonzalez-Reiche AS, Hernandez MM, Sullivan MJ, Ciferri B, Alshammary H, Obla A, et al. Introductions and early spread of SARS-CoV-2 in the New York City area. Science 2020;.
22. Ming W, Huang J, Zhang C. Breaking down of the healthcare system: Mathematical modelling for controlling the novel coronavirus (2019-nCoV) outbreak in wuhan, chinadoi: 10.1101/2020.01. 27.922443. URL https://doi org/101101% 2F2020 2020;1:922443.
23. Silverman JD, Hupert N, Washburne AD. Using ILI surveillance to estimate state-specific case detection rates and forecast SARS-CoV-2 spread in the United States. medRxiv 2020;.
24. Kendall M, Parker M, Fraser C, Nurtay A, Wymant C, Bonsall D, et al. Quantifying SARS-CoV-2 transmission suggests epidemic control with digital contact tracing. Science 2020;.
25. Topley W, Wilson G. The spread of bacterial infection. The problem of herd-immunity. Epidemiology & Infection 1923;21(3):243–249.
26. Wikipedia, Wikipedia:Identifying reliable sources (medicine); 2021. https://en.wikipedia.org/wiki/Wikipedia:Identifying_reliable_sources_(medicine).
27. Zhou P, Yang XL, Wang XG, Hu B, Zhang L, Zhang W, et al. Discovery of a novel coronavirus associated with the recent pneumonia outbreak in humans and its potential bat origin. BioRxiv 2020;.
28. Keegan B, Gergle D, Contractor N. Hot off the wiki: Structures and dynamics of Wikipedia's coverage of breaking news events. American Behavioral Scientist 2013;57(5):595–622.
29. Kagan D, Moran-Gilad J, Fire M. Scientometric trends for coronaviruses and other emerging viral infections. GigaScience 2020;9(8):giaa085.
30. Generous N, Fairchild G, Deshpande A, Del Valle SY, Priedhorsky R. Global disease monitoring and forecasting with Wikipedia. PLoS Comput Biol 2014;10(11):e1003892.
31. Wu Q, Irani D, Pu C, Ramaswamy L. Elusive Vandalism Detection in Wikipedia: A Text Stability-Based Approach. In: Proceedings of the 19th ACM International Conference on Information and Knowledge Management CIKM '10, New York, NY, USA: Association for Computing Machinery; 2010. p. 1797–1800. https://doi.org/10.1145/1871437.1871732.
32. Maggio LA, Steinberg RM, Piccardi T, Willinsky JM. Meta-Research: Reader engagement with medical content on

Wikipedia. Elife 2020;9:e52426.
33. Yasseri T, Sumi R, Rung A, Kornai A, Kertész J. Dynamics of conflicts in Wikipedia. PloS one 2012;7(6):e38869.
34. Joorabchi A, Doherty C, Dawson J. 'WP2Cochrane', a tool linking Wikipedia to the Cochrane Library: Results of a bibliometric analysis evaluating article quality and importance. Health Informatics Journal 2020;26(3):1881–1897.
35. Wikipedia, Wikipedia Project COVID-19: Reference sources; 2021. https://en.wikipedia.org/wiki/Wikipedia:WikiProject_COVID-19/Reference_sources.
36. Fu DY, Hughey JJ. Meta-Research: Releasing a preprint is associated with more attention and citations for the peer-reviewed article. Elife 2019;8:e52646.
37. Fraser N, Brierley L, Dey G, Polka JK, Pálfy M, Coates JA. Preprinting a pandemic: the role of preprints in the COVID-19 pandemic. bioRxiv 2020;.
38. Pooladian A, Borrego Á. Methodological issues in measuring citations in Wikipedia: a case study in Library and Information Science. Scientometrics 2017;113(1):455–464.
39. Verma AA, Dubey N, Iyengar SRS, Setia S. In: Tracing the Factoids: The Anatomy of Information Re-Organization in Wikipedia Articles New York, NY, USA: Association for Computing Machinery; 2021. p. 572–579. https://doi.org/10.1145/3442442.3452342.
40. Greshake Tzovaras B, Angrist M, Arvai K, Dulaney M, Estrada-Galiñanes V, Gunderson B, et al. Open Humans: A platform for participant-centered research and personal data exploration. GigaScience 2019;8(6):giz076.
41. Sobel J, Henry L, Rotman N, Rando G. BeerDeCoded: the open beer metagenome project. F1000Research 2017;6.
42. Conrad CC, Hilchey KG. A review of citizen science and community-based environmental monitoring: issues and opportunities. Environmental monitoring and assessment 2011;176(1):273–291.
43. McGowan ML, Choudhury S, Juengst ET, Lambrix M, Settersten RA, Fishman JR. "Let's pull these technologies out of the ivory tower": The politics, ethos, and ironies of participant-driven genomic research. BioSocieties 2017;12(4):494–519.
44. ;.
45. Kwok R. Research impact: Altmetrics make their mark. Nature 2013;500(7463):491–493.
46. Meschede C, Siebenlist T. Cross-metric compatability and inconsistencies of altmetrics. Scientometrics 2018;115(1):283–297.
47. Paskin N. Digital object identifier (DOI®) system. Encyclopedia of library and information sciences 2010;3:1586–1592.

Supplementary information

Table 1. Preprints cited within the Wikipedia COVID-19 Corpus

title	doi	authorString	pubYear
Isolation and Characterization of 2019-nCoV-like Coronavirus from Malayan Pangolins	10.1101/2020.02.17.951335	Xiao K, Zhai J, Feng Y, Zhou N, Zhang X, Zou J, Li N, Guo Y, Li X, Shen X, Zhang Z, Shu F, Huang W, Li Y, Zhang Z, Chen R, Wu Y, Peng S, Huang M, Xie W, Cai Q, Hou F, Liu Y, Chen W, Xiao L, Shen Y.	2020
Evidence of recombination in coronaviruses implicating pangolin origins of nCoV-2019	10.1101/2020.02.07.939207	Wong MC, Javornik Cregeen SJ, Ajami NJ, Petrosino JF.	2020
Spike mutation pipeline reveals the emergence of a more transmissible form of SARS-CoV-2	10.1101/2020.04.29.069054	Korber B, Fischer W, Gnanakaran S, Yoon H, Theiler J, Abfalterer W, Foley B, Giorgi E, Bhattacharya T, Parker M, Partridge D, Evans C, Freeman T, de Silva T, LaBranche C, Montefiori D, on behalf of the Sheffield COVID-19 Genomics Group.	2020
Global profiling of SARS-CoV-2 specific IgG/IgM responses of convalescents using a proteome microarray	10.1101/2020.03.20.20039495	Jiang H, Li Y, Zhang H, Wang W, Men D, Yang X, Qi H, Zhou J, Tao S.	2020
Novel coronavirus 2019-nCoV: early estimation of epidemiological parameters and epidemic predictions	10.1101/2020.01.23.20018549	Read JM, Bridgen JR, Cummings DA, Ho A, Jewell CP.	2020
Aerodynamic Characteristics and RNA Concentration of SARS-CoV-2 Aerosol in Wuhan Hospitals during COVID-19 Outbreak	10.1101/2020.03.08.982637	Liu Y, Ning Z, Chen Y, Guo M, Liu Y, Gali NK, Sun L, Duan Y, Cai J, Westerdahl D, Liu X, Ho K, Kan H, Fu Q, Lan K.	2020
Correlation Analysis Between Disease Severity and Inflammation-related Parameters in Patients with COVID-19 Pneumonia	10.1101/2020.02.25.20025643	Gong J, Dong H, Xia SQ, Huang YZ, Wang D, Zhao Y, Liu W, Tu S, Zhang M, Wang Q, Lu F.	2020
Estimation of COVID-2019 burden and potential for international dissemination of infection from Iran	10.1101/2020.02.24.20027375	Tuite AR, Bogoch I, Sherbo R, Watts A, Fisman DN, Khan K.	2020
Explaining national differences in the mortality of COVID-19: individual patient simulation model to investigate the effects of testing policy and other factors on apparent mortality.	10.1101/2020.04.02.20050633	Michaels JA, Stevenson MD.	2020
Saliva is more sensitive for SARS-CoV-2 detection in COVID-19 patients than nasopharyngeal swabs	10.1101/2020.04.16.20067835	Wyllie AL, Fournier J, Casanovas-Massana A, Campbell M, Tokuyama M, Vijayakumar P, Geng B, Muenker MC, Moore AJ, Vogels CBF, Petrone ME, Ott IM, Lu P, Lu-Culligan A, Klein J, Venkataraman A, Earnest R, Simonov M, Datta R, Handoko R, Naushad N, Sewanan LR, Valdez J, White EB, Lapidus S, Kalinich CC, Jiang X, Kim DJ, Kudo E, Linehan M, Mao T, Moriyama M, Oh JE, Park A, Silva J, Song E, Takahashi T, Taura M, Weizman O, Wong P, Yang Y, Bermejo S, Odio C, Omer SB, Dela Cruz CS, Farhadian S, Martinello RA, Iwasaki A, Grubaugh ND, Ko AI.	2020
Neutralizing antibody responses to SARS-CoV-2 in a COVID-19 recovered patient cohort and their implications	10.1101/2020.03.30.20047365	Wu F, Wang A, Liu M, Wang Q, Chen J, Xia S, Ling Y, Zhang Y, Xun J, Lu L, Jiang S, Lu H, Wen Y, Huang J.	2020
Estimation of SARS-CoV-2 Infection Prevalence in Santa Clara County	10.1101/2020.03.24.20043067	Yadlowsky S, Shah N, Steinhardt J.	2020
Population-level COVID-19 mortality risk for non-elderly individuals overall and for non-elderly individuals without underlying diseases in pandemic epicenters	10.1101/2020.04.05.20054361	Ioannidis JPA, Axfors C, Contopoulos-Ioannidis DG.	2020
Respiratory disease and virus shedding in rhesus macaques inoculated with SARS-CoV-2	10.1101/2020.03.21.001628	Munster VJ, Feldmann F, Williamson BN, van Doremalen N, Pérez-Pérez L, Schulz J, Meade-White K, Okumura A, Callison J, Brumbaugh B, Avanzato VA, Rosenke R, Hanley PW, Saturday G, Scott D, Fischer ER, de Wit E.	2020
Clinical benefit of remdesivir in rhesus macaques infected with SARS-CoV-2	10.1101/2020.04.15.043166	Williamson BN, Feldmann F, Schwarz B, Meade-White K, Porter DP, Schulz J, Doremalen Nv, Leighton I, Yinda CK, Pérez-Pérez L, Okumura A, Lovaglio J, Hanley PW, Saturday G, Bosio CM, Anzick S, Barbian K, Cihlar T, Martens C, Scott DP, Munster VJ, Wit Ed.	2020
Discovery of a novel coronavirus associated with the recent pneumonia outbreak in humans and its potential bat origin	10.1101/2020.01.22.914952	Zhou P, Yang X, Wang X, Hu B, Zhang L, Zhang W, Si H, Zhu Y, Li B, Huang C, Chen H, Chen J, Luo Y, Guo H, Jiang R, Liu M, Chen Y, Shen X, Wang X, Zheng X, Zhao K, Chen Q, Deng F, Liu L, Yan B, Zhan F, Wang Y, Xiao G, Shi Z.	2020

Breaking down of the healthcare system: Mathematical modelling for controlling the novel coronavirus (2019-nCoV) outbreak in Wuhan, China	10.1101/2020.01.27.922443	Ming W, Huang J, Zhang CJP.	2020
Introductions and early spread of SARS-CoV-2 in the New York City area	10.1101/2020.04.08.20056929	Gonzalez-Reiche AS, Hernandez MM, Sullivan M, Ciferri B, Alshammary H, Obla A, Fabre S, Kleiner G, Polanco J, Khan Z, Alburquerque B, van de Guchte A, Dutta J, Francoeur N, Melo BS, Oussenko I, Deikus G, Soto J, Sridhar SH, Wang Y, Twyman K, Kasarskis A, Altman DR, Smith M, Sebra R, Aberg J, Krammer F, Garcia-Sarstre A, Luksza M, Patel G, Paniz-Mondolfi A, Gitman M, Sordillo EM, Simon V, van Bakel H.	2020
Phylodynamics of SARS-CoV-2 transmission in Spain	10.1101/2020.04.20.050039	Díez-Fuertes F, Iglesias-Caballero M, Monzón S, Jiménez P, Varona S, Cuesta I, Zaballos Á, Thomson MM, Jiménez M, García Pérez J, Pozo F, Pérez-Olmeda M, Alcamí J, Casas I.	2020
Using ILI surveillance to estimate state-specific case detection rates and forecast SARS-CoV-2 spread in the United States	10.1101/2020.04.01.20050542	Silverman JD, Hupert N, Washburne AD.	2020
Quantifying SARS-CoV-2 transmission suggests epidemic control with digital contact tracing	10.1101/2020.03.08.20032946	Ferretti L, Wymant C, Kendall M, Zhao L, Nurtay A, Abeler-Dorner L, Parker M, Bonsall DG, Fraser C.	2020
Adoption and impact of non-pharmaceutical interventions for COVID-19	10.12688/wellcomeopenres.15808.1	Imai N, Gaythorpe KA, Abbott S, Bhatia S, van Elsland S, Prem K, Liu Y, Ferguson NM.	2020
Aberrant pathogenic GM-CSF+ T cells and inflammatory CD14+CD16+ monocytes in severe pulmonary syndrome patients of a new coronavirus	10.1101/2020.02.12.945576	Zhou Y, Fu B, Zheng X, Wang D, Zhao C, qi Y, Sun R, Tian Z, Xu X, Wei H.	2020
SARS-CoV-2 invades host cells via a novel route: CD147-spike protein	10.1101/2020.03.14.988345	Wang K, Chen W, Zhou Y, Lian J, Zhang Z, Du P, Gong L, Zhang Y, Cui H, Geng J, Wang B, Sun X, Wang C, Yang X, Lin P, Deng Y, Wei D, Yang X, Zhu Y, Zhang K, Zheng Z, Miao J, Guo T, Shi Y, Zhang J, Fu L, Wang Q, Bian H, Zhu P, Chen Z.	2020
Functional assessment of cell entry and receptor usage for lineage B ß-coronaviruses, including 2019-nCoV	10.1101/2020.01.22.915660	Letko M, Munster V.	2020
Broad anti-coronaviral activity of FDA approved drugs against SARS-CoV-2 in vitro and SARS-CoV in vivo	10.1101/2020.03.25.008482	Weston S, Coleman CM, Haupt R, Logue J, Matthews K, Frieman MB.	2020
Global and Temporal Patterns of Submicroscopic Plasmodium falciparum Malaria Infection	10.1101/554311	Whittaker C, Slater H, Bousema T, Drakeley C, Ghani A, Okell L.	2019

Table 2. Most cited scientific papers in COVID-19 Wikipedia corpus

doi	Authors	OA	Journal	Year	Source	Title	Wiki	Sci.lit
10.1038/s41586-020-2012-7	Zhou P, Yang XL, Wang XG, Hu B, Zhang L, Zhang W, Si HR, Zhu Y, Li B, Huang CL, Chen HD, Chen J, Luo Y, Guo H, Jiang RD, Liu MQ, Chen Y, Shen XR, Wang X, Zheng XS, Zhao K, Chen QJ, Deng F, Liu LL, Yan B, Zhan FX, Wang YY, Xiao GF, Shi ZL.	Y	Nature	2020	MED	A pneumonia outbreak associated with a new coronavirus of probable bat origin.	8	940
10.3390/v11020174	Wong ACP, Li X, Lau SKP, Woo PCY.	Y	Viruses	2019	MED	Global Epidemiology of Bat Coronaviruses.	6	28
10.1016/j.ijid.2020.01.009	Hui DS, I Azhar E, Madani TA, Ntoumi F, Kock R, Dar O, Ippolito G, Mchugh TD, Memish ZA, Drosten C, Zumla A, Petersen E.	Y	Int J Infect Dis	2020	MED	The continuing 2019-nCoV epidemic threat of novel coronaviruses to global health - The latest 2019 novel coronavirus outbreak in Wuhan, China.	5	228
10.1016/j.jmii.2020.03.013	Lau H, Khosrawipour V, Kocbach P, Mikolajczyk A, Ichii H, Schubert J, Bania J, Khosrawipour T.	Y	J Microbiol Immunol Infect	2020	MED	Internationally lost COVID-19 cases.	5	5
10.1038/d41586-020-00548-w	Cyranoski D.	N	Nature	2020	MED	Mystery deepens over animal source of coronavirus.	5	8
10.1038/s41591-020-0820-9	Andersen KG, Rambaut A, Lipkin WI, Holmes EC, Garry RF.	Y	Nat Med	2020	MED	The proximal origin of SARS-CoV-2.	5	147
10.3390/v2081803	Woo PC, Huang Y, Lau SK, Yuen KY.	Y	Viruses	2010	MED	Coronavirus genomics and bioinformatics analysis.	5	109
10.1007/978-1-4939-2438-7₁	Fehr AR, Perlman S.	Y	Methods Mol Biol	2015	MED	Coronaviruses: an overview of their replication and pathogenesis.	4	195
10.1007/s00134-020-05991-x	Ruan Q, Yang K, Wang W, Jiang L, Song J.	Y	Intensive Care Med	2020	MED	Clinical predictors of mortality due to COVID-19 based on an analysis of data of 150 patients from Wuhan, China.	4	66
10.1038/d41573-020-00016-0	Li G, De Clercq E.	N	Nat Rev Drug Discov	2020	MED	Therapeutic options for the 2019 novel coronavirus (2019-nCoV).	4	105
10.1038/s41422-020-0282-0	Wang M, Cao R, Zhang L, Yang X, Liu J, Xu M, Shi Z, Hu Z, Zhong W, Xiao G.	Y	Cell Res	2020	MED	Remdesivir and chloroquine effectively inhibit the recently emerged novel coronavirus (2019-nCoV) in vitro.	4	474
10.1093/cid/ciaa149	To KK, Tsang OT, Chik-Yan Yip C, Chan KH, Wu TC, Chan JMC, Leung WS, Chik TS, Choi CY, Kandamby DH, Lung DC, Tam AR, Poon RW, Fung AY, Hung IF, Cheng VC, Chan JF, Yuen KY.	Y	Clin Infect Dis	2020	MED	Consistent detection of 2019 novel coronavirus in saliva.	4	94
10.1093/ofid/ofaa105	McCreary EK, Pogue JM.	Y	Open Forum Infect Dis	2020	MED	Coronavirus Disease 2019 Treatment: A Review of Early and Emerging Options.	4	7
10.1126/science.aba9757	Chinazzi M, Davis JT, Ajelli M, Gioannini C, Litvinova M, Merler S, Pastore Y Piontti A, Mu K, Rossi L, Sun K, Viboud C, Xiong X, Yu H, Halloran ME, Longini IM, Vespignani A.	Y	Science	2020	MED	The effect of travel restrictions on the spread of the 2019 novel coronavirus (COVID-19) outbreak.	4	69
10.1093/jtm/taaa030	Rocklöv J, Sjödin H, Wilder-Smith A.	Y	J Travel Med	2020	MED	COVID-19 outbreak on the Diamond Princess cruise ship: estimating the epidemic potential and effectiveness of public health countermeasures.	3	25
10.1101/2020.02.07.939207	Wong MC, Javornik Cregeen SJ, Ajami NJ, Petrosino JF.	N	NA	2020	PPR	Evidence of recombination in coronaviruses implicating pangolin origins of nCoV-2019	3	15
10.1101/2020.02.17.951335	Xiao K, Zhai J, Feng Y, Zhou N, Zhang X, Zou J, Li N, Guo Y, Li X, Shen X, Zhang Z, Shu F, Huang W, Li Y, Zhang Z, Chen R, Wu Y, Peng S, Huang M, Xie W, Cai Q, Hou F, Liu Y, Chen W, Xiao L, Shen Y.	N	NA	2020	PPR	Isolation and Characterization of 2019-nCoV-like Coronavirus from Malayan Pangolins	3	24
10.1111/j.1600-0668.2007.00469.x	Xie X, Li Y, Chwang AT, Ho PL, Seto WH.	N	Indoor Air	2007	MED	How far droplets can move in indoor environments-revisiting the Wells evaporation-falling curve.	3	167
10.1111/tmi.13383	Velavan TP, Meyer CG.	Y	Trop Med Int Health	2020	MED	The COVID-19 epidemic.	3	70
10.1126/science.1118391	Li W, Shi Z, Yu M, Ren W, Smith C, Epstein JH, Wang H, Crameri G, Hu Z, Zhang H, Zhang J, McEachern J, Field H, Daszak P, Eaton BT, Zhang S, Wang LF.	N	Science	2005	MED	Bats are natural reservoirs of SARS-like coronaviruses.	3	967

Table 3. Most cited scientific papers in the scientific literature within COVID-19 Wikipedia corpus

Title	Year	Journal	Authors	Citation Count
Understanding the Warburg effect: the metabolic requirements of cell proliferation.	2009	Science	Vander Heiden MG, Cantley LC, Thompson CB.	4927
The MIQE guidelines: minimum information for publication of quantitative real-time PCR experiments.	2009	Clin Chem	Bustin SA, Benes V, Garson JA, Hellemans J, Huggett J, Kubista M, Mueller R, Nolan T, Pfaffl MW, Shipley GL, Vandesompele J, Wittwer CT.	4809
Isolation of a cDNA clone derived from a blood-borne non-A, non-B viral hepatitis genome.	1989	Science	Choo QL, Kuo G, Weiner AJ, Overby LR, Bradley DW, Houghton M.	3672
Isolation of a T-lymphotropic retrovirus from a patient at risk for acquired immune deficiency syndrome (AIDS).	1983	Science	Barré-Sinoussi F, Chermann JC, Rey F, Nugeyre MT, Chamaret S, Gruest J, Dauguet C, Axler-Blin C, Vézinet-Brun F, Rouzioux C, Rozenbaum W, Montagnier L.	3016
The American-European Consensus Conference on ARDS. Definitions, mechanisms, relevant outcomes, and clinical trial coordination.	1994	Am J Respir Crit Care Med	Bernard GR, Artigas A, Brigham KL, Carlet J, Falke K, Hudson L, Lamy M, Legall JR, Morris A, Spragg R.	2904
Toll-like receptors.	2003	Annu Rev Immunol	Takeda K, Kaisho T, Akira S.	2872
The acute respiratory distress syndrome.	2000	N Engl J Med	Ware LB, Matthay MA.	2720
Network biology: understanding the cell's functional organization.	2004	Nat Rev Genet	Barabási AL, Oltvai ZN.	2697
Surviving sepsis campaign: international guidelines for management of severe sepsis and septic shock: 2012.	2013	Crit Care Med	Dellinger RP, Levy MM, Rhodes A, Annane D, Gerlach H, Opal SM, Sevransky JE, Sprung CL, Douglas IS, Jaeschke R, Osborn TM, Nunnally ME, Townsend SR, Reinhart K, Kleinpell RM, Angus DC, Deutschman CS, Machado FR, Rubenfeld GD, Webb SA, Beale RJ, Vincent JL, Moreno R, Surviving Sepsis Campaign Guidelines Committee including the Pediatric Subgroup.	2461
A comprehensive analysis of protein-protein interactions in Saccharomyces cerevisiae.	2000	Nature	Uetz P, Giot L, Cagney G, Mansfield TA, Judson RS, Knight JR, Lockshon D, Narayan V, Srinivasan M, Pochart P, Qureshi-Emili A, Li Y, Godwin B, Conover D, Kalbfleisch T, Vijayadamodar G, Yang M, Johnston M, Fields S, Rothberg JM.	2416

SI datasets

(1) Table of scientific paper form europmc COVID-19 cited in wikipedia
(2) Table of Wikipedia article-DOI network
(3) Table of protected wikipedia COVID-19 articles

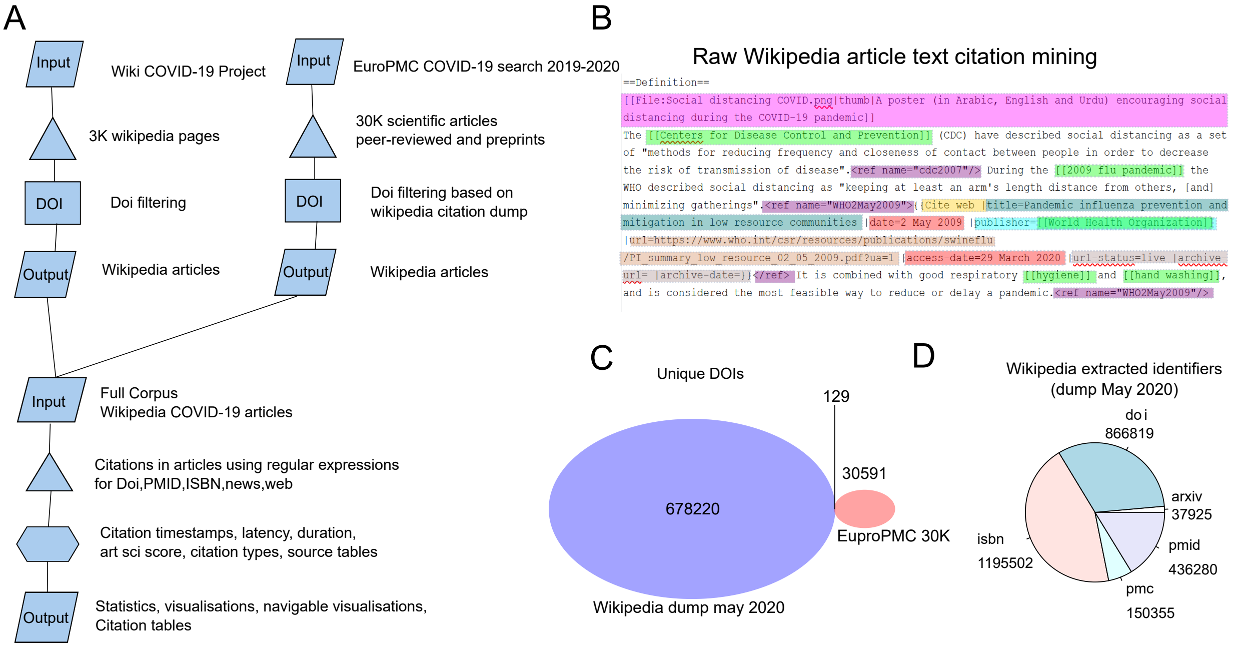

Figure S1. Corpus identification and citation extraction pipeline. A) Scheme of the Corpus delimitation rational and citation extraction. To delimit our corpus of Wikipedia articles containing Digital Object Identifier (DOI), we applied two different strategies. First we scraped every Wikipedia pages form the COVID-19 Wikipedia project (about 3K pages) and we filtered them to keep only page containing DOI citations (149 Wikipedia articles). For our second strategy, we made a search with EuroPMC on COVID-19, SARS-CoV2, SARS-nCoV19 (30,000 sci papers, reviews and preprints) and a selection on scientific papers form 2019 onwards that we compared to the Wikipedia extracted citations from the English Wikipedia dump of May 2020 (860'000 DOIs). This search led to 91 Wikipedia articles containing at least one citation of the EuroPMC search. Taken together, from our 231 Wikipedia articles corpus we extracted DOIs, PMIDs, ISBNs, websites and URLs using a set of regular expressions, as described in the methods. Subsequently, we computed several statistics for each Wikipedia article and we retrieved Atmetics, CrossRef and EuroPMC information for each DOI. Finally, our method allows to produce tables of citations annotated and extracted information in each Wikipadia articles such as books, websites, newspapers. In addition,a timeline of Wikipedia articles and a network of Wikipedia articles linked to scientific papers is built. B) Example of raw Wikipedia text from the social distancing article highlighted with several parsed items from a reference. pink: a hyperlink to an image file, green: Wikipedia hyperlinks, purple: reference, yellow: citation type, dark green: citation title, red: citation date, orange: citation URL. C) Overlap between DOI from the Wikipedia dump and the 30K EuroPMC COVID-19-related scientific articles and preprints D) number of extracted citations with *mwcite* from the English Wikipedia dump of May 2020.

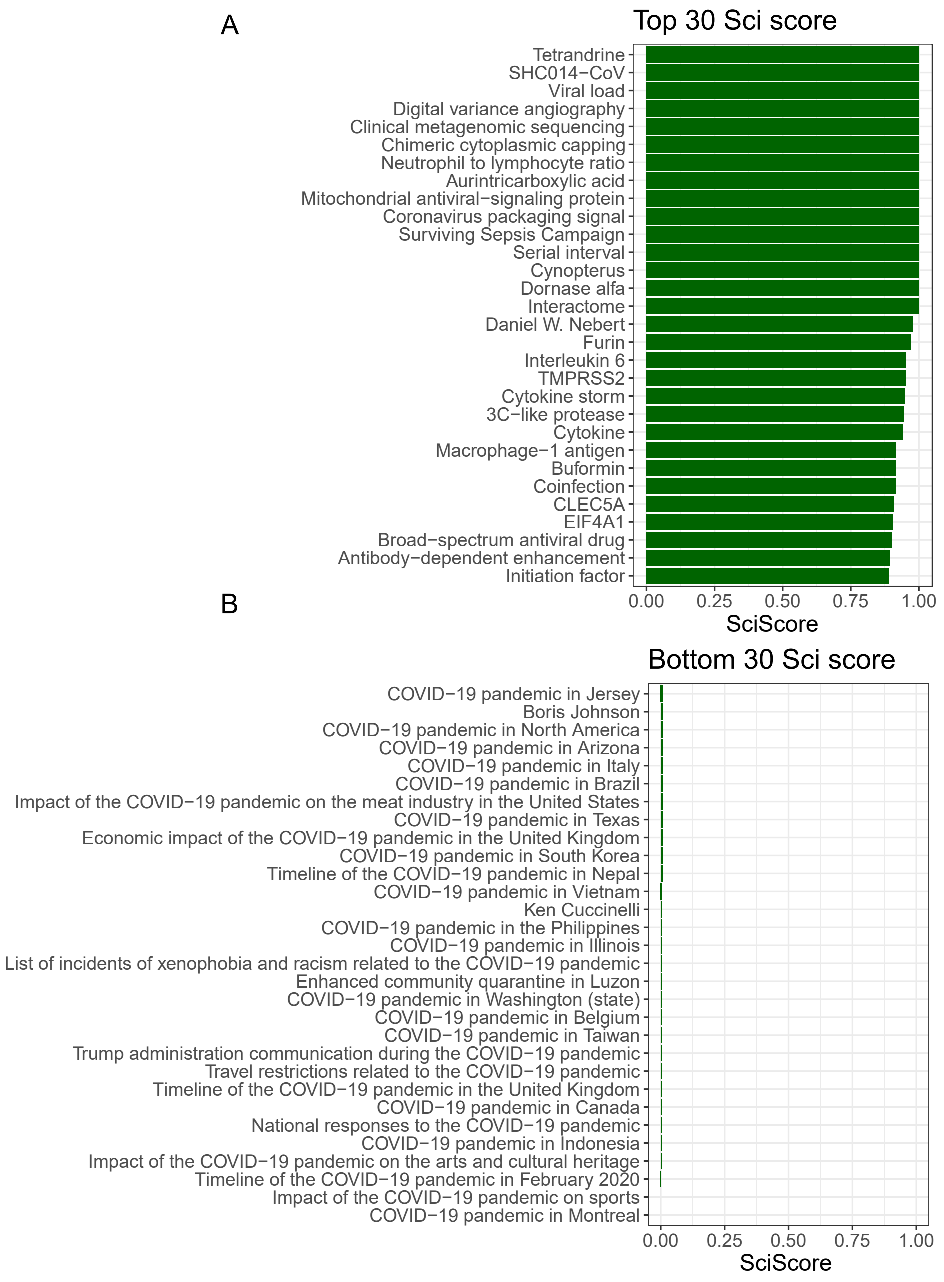

Figure S2. Top and bottom scientific score from Wikipedia article COVID-19 corpus. The scientific score was computed based on the reference content of each Wikipedia article from the COVID-19 corpus as defined in the methods section. A) Top 30 scientific article from the COVID-19 corpus. B) Bottom scientific article from the COVID-19 corpus.

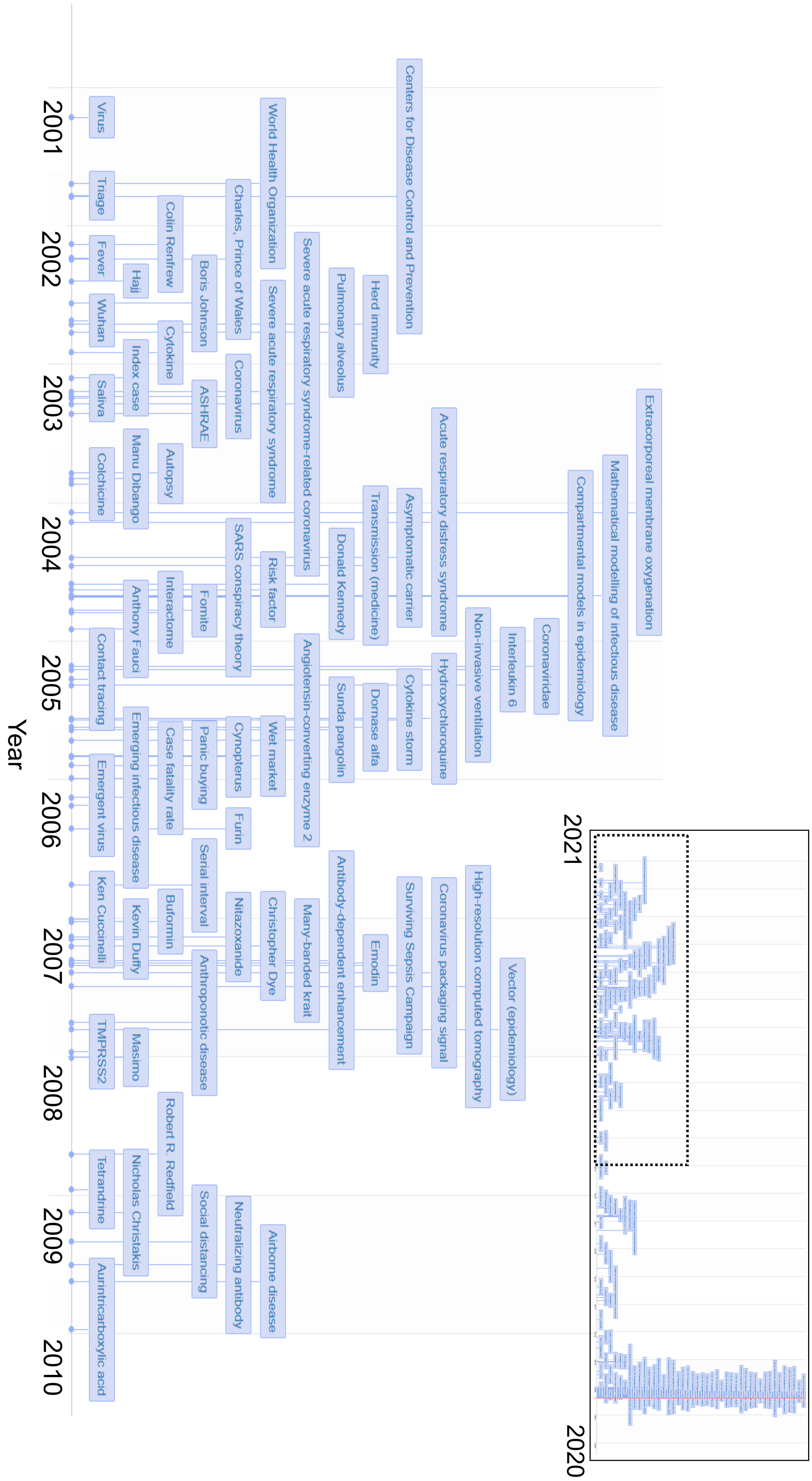

Figure S3. Timeline of the Wikipedia COVID-19 corpus. See here for an interactive version of the timeline.

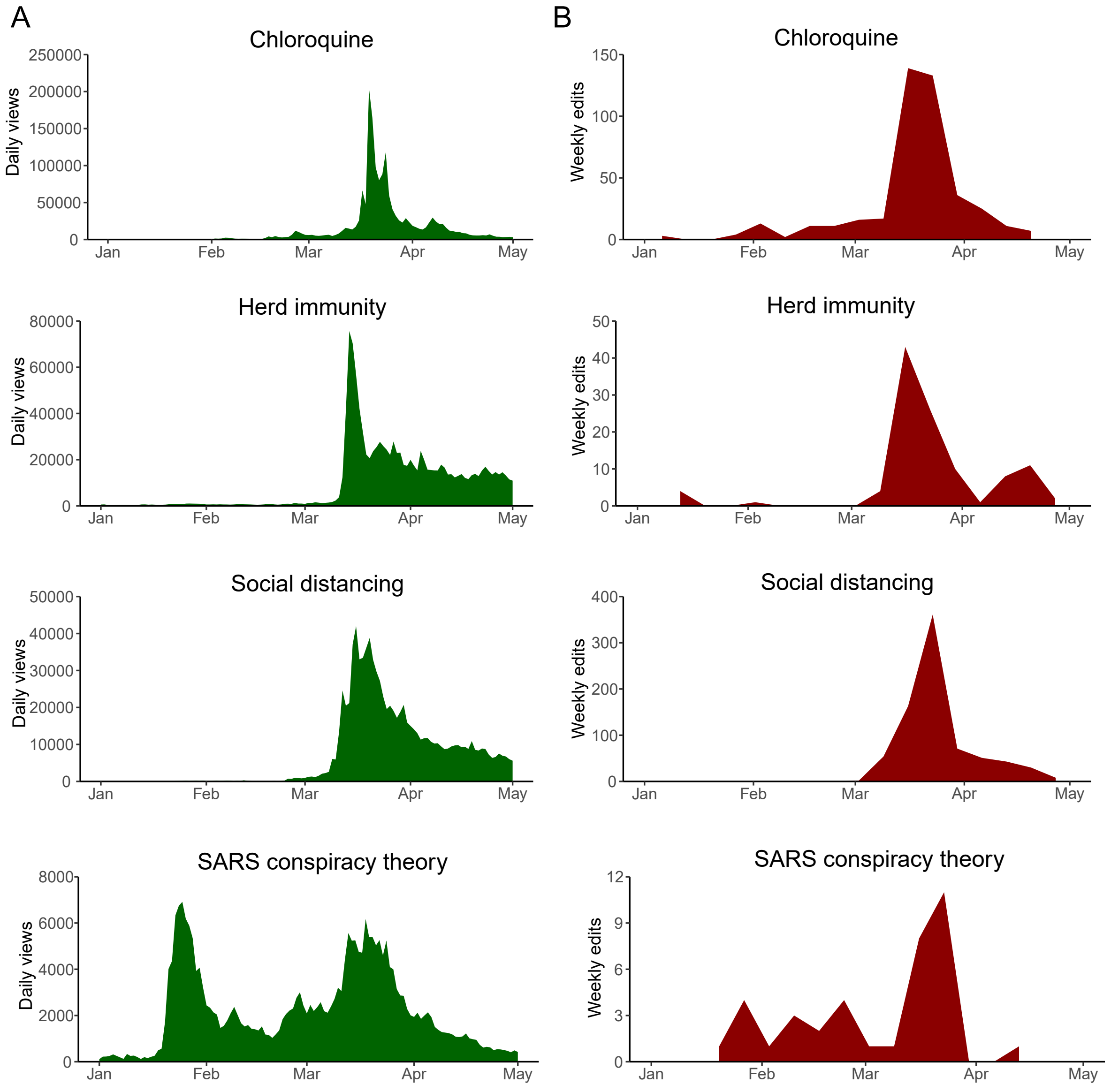

Figure S4. Wikipedia article page views and edits during COVID-19 pandemics. A) Daily page views and B) weekly edits for selected Wikipedia articles.

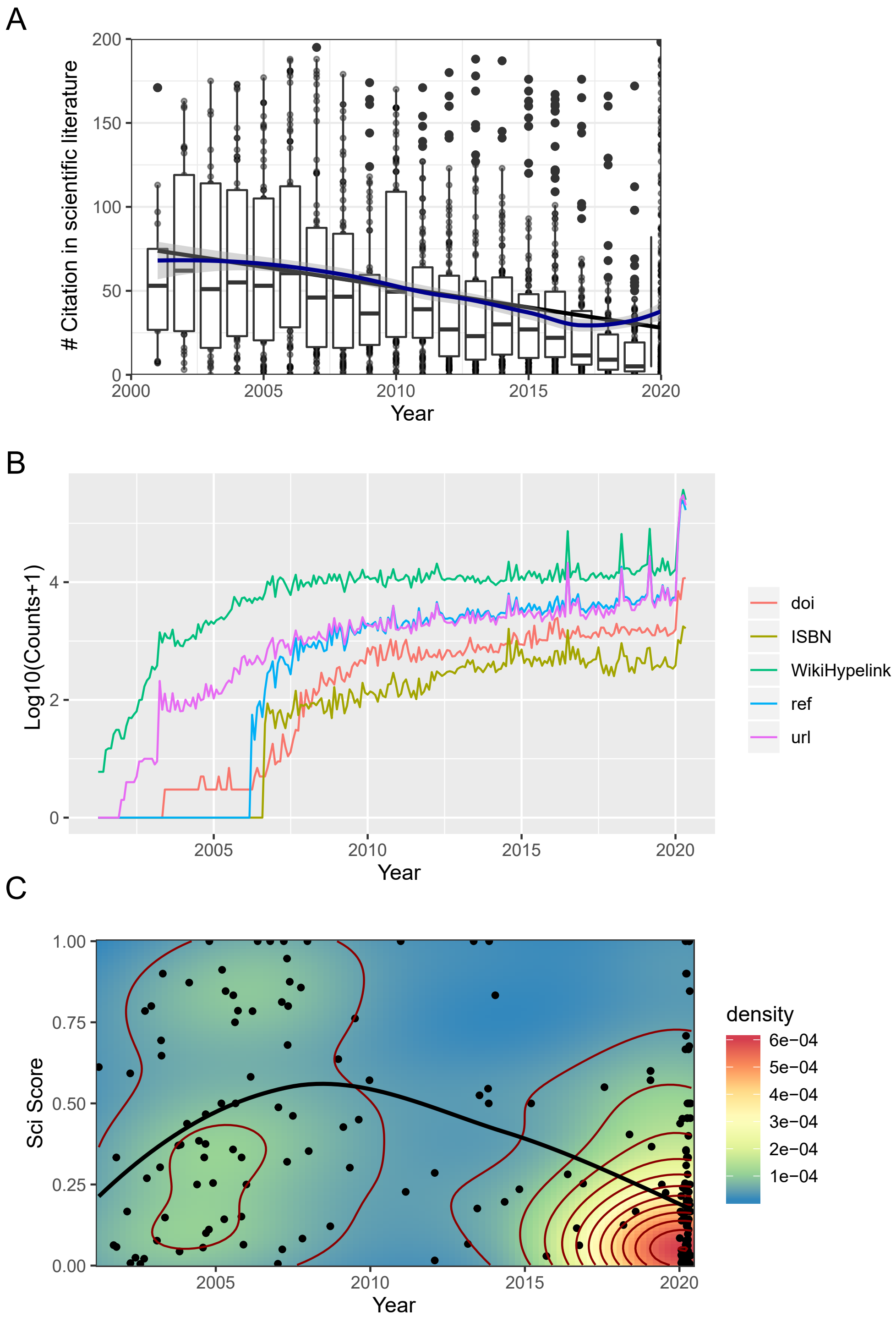

Figure S5. Historical perspective, citations count, citation type and scientific score of the COVID-19 corpus. A) Scientific literature citation count in function of the year of publication. B) Citation count in function of the year for different type of citation (doi, isbn, hyperlink, url). C) Scientific score in function of the creation date of wikipedia article.

supp. data 2

Click here to access/download
Supplementary Material
Supp_data_Network.csv

supp. data 3

Click here to access/download

Supplementary Material

Supp_data_protected_corpus.csv

supp. data 1

Click here to access/download
Supplementary Material
supp_data_intesect_europmc_search_dump_doi_annota
ted.xlsx

Click here to access/download;Personal Cover;Cover_gigascience.docx

Haifa, 16/09/2021

Dear editor,
We humbly resubmit for publication our manuscript, now titled: “Citation needed? Wikipedia bibliometrics during the first wave of the COVID pandemic.” The change in title, as well as the new abstract and reworked text, are the result of the highly instructive and detailed feedback we received from the reviewers.

We were pleased to read the positive responses from the reviewers and made intensive efforts to revise the text according to their suggestions: Simplifying our introduction section, writing an introductory paragraph to our methods section, reworking key figures, improving our documentation (though our R package is still in development), and clearly including terms that better describe our work for what it is - a bibliometric citation analysis of Wikipedia during the first wave of the pandemic.

Thanks to reviewer 1#, we have substantially expanded our bibliography to include a number of important studies published in GigaScience. These helped to contextualize our findings. We also clarified, in wake of the suggestions of reviewer #2, the term “scientific infrastructure” - a process that has helped make both our findings and our conclusions more coherent in terms of the factors that aided Wikipedia in maintaining such high-quality sources during the pandemic.

Aided by reviewer #3, we utilized Wikipedia’s model ourselves - addressing issues flagged for us by “Wikipedians” who also reviewed our work. Their perspective and comments clarified key internal processes and thus underscored the value Wikipedia’s model can have.

Moreover, reviewers #1 and #3 both asked us to clarify our proposed solution to the tradeoff between timeliness and scientificness. This issue has now been significantly addressed in our new and reworked discussion section. This section, for example, now offers a solution for how Wikipedia’s editors can make better use of preprints, utilizing them in a temporary manner but making sure to update them as new research is published.

The discussion now also includes an expanded “limitations paragraph” (as requested by reviewers #2 and #3) that touches on the shortcomings of our work in terms of scale and scope - but also explains how our conclusions can be generalized. In this regard, we offer the framework of citizen science and suggest viewing Wikipedia’s editors as citizen encyclopedists. This, alongside other mechanisms detailed in our revised discussion section, provides an infrastructure which makes Wikipedia well poised to play a bigger role in communicating science and fending off disinformation - be it about coronavirus or otherwise.

As the reviewers put it, alongside its value as a “thorough and meticulous” study, there were numerous “opportunities for improvements” throughout the text that merited a major revision. We couldn't agree more. And now that we have addressed these as well as others, we feel confident our work should find a place in your prestigious journal.

Respectfully yours,
Omer Benjakob, Dr. Rona Aviram and Dr. Jonathan Sobel
