# Supplementary material for: Citation needed? Wikipedia bibliometrics during the first wave of the COVID-19 pandemic
Source: Gigascience. 2022 Jan 12;11:giab095. doi: 10.1093/gigascience/giab095 (PMC8756189; doi:10.1093/gigascience/giab095)
Supplement: giab095_GIGA-D-21-00139_Revision_2 [file giab095_giga-d-21-00139_revision_2.pdf]

GigaScience

Citation needed? Wikipedia bibliometrics during the first wave of the COVID-19 pandemic.

--Manuscript Draft--

**Manuscript Number:**	GIGA-D-21-00139R2
**Full Title:**	Citation needed? Wikipedia bibliometrics during the first wave of the COVID-19 pandemic.
**Article Type:**	Research
**Funding Information:**	Azrieli Foundation — Dr. Rona Aviram<br>Placide Nicod Foundation — Dr. Jonathan Aryeh Sobel
**Abstract:**	Background<br>With the COVID-19 pandemic's outbreak, millions flocked to Wikipedia for updated information. Amid growing concerns regarding an "infodemic", ensuring the quality of information is a crucial vector of public health. Investigating if and how Wikipedia remained up to date and in line with science is key to formulating strategies to counter misinformation. Using citation analyses, we asked: which sources informed Wikipedia's COVID-19-related articles before and during the pandemic's first wave (January-May 2020).<br>Results<br>We found that coronavirus-related articles referenced trusted media outlets and high-quality academic sources. Regarding academic sources, Wikipedia was found to be highly selective in terms of what science was cited. Moreover, despite a surge in COVID-19 preprints, Wikipedia had a clear preference for open-access studies published in respected journals and made little use of preprints. Building a timeline of English COVID-19 articles from 2001-2020 revealed a nuanced trade-off between quality and timeliness. It further showed how preexisting articles on key topics related to the virus created a framework for integrating new knowledge. Supported by a rigid sourcing policy, this "scientific infrastructure" facilitated contextualization and regulated the influx of new information. Lastly, we constructed a network of DOI-Wikipedia articles, which showed the landscape of pandemic-related knowledge on Wikipedia and how academic citations create a web of shared knowledge supporting topics like COVID-19 drug development.<br>Conclusions<br>Understanding how scientific research interacts with the digital knowledge-sphere during the pandemic provides insight into how Wikipedia can facilitate access to science. It also reveals how, aided by what we term its "citizen encyclopedists", it successfully fended off COVID-19 disinformation and how this unique model may be deployed in other contexts.
**Corresponding Author:**	Jonathan Aryeh Sobel, Ph.D.<br>Technion Israel Institute of Technology<br>Haifa, ISRAEL
**Corresponding Author Secondary Information:**	
**Corresponding Author's Institution:**	Technion Israel Institute of Technology
**Corresponding Author's Secondary Institution:**	
**First Author:**	Omer Benjakob
**First Author Secondary Information:**	
**Order of Authors:**	Omer Benjakob<br>Rona Aviram, Ph.D.<br>Jonathan Aryeh Sobel, Ph.D.
**Order of Authors Secondary Information:**	

Powered by Editorial Manager® and ProduXion Manager® from Aries Systems Corporation

Response to Reviewers:	As required, we rearrange our data availability section to add the reference to the GigaDB repository and we made minor corrections to the main text. We arranged the order of some supplementary figure panels and tables to match better the flow of our main text.<br>Finally, per the suggestion of reviewer 3 we performed intense proofing of our main text.
Additional Information:	
Question	Response
Are you submitting this manuscript to a special series or article collection?	No
**Experimental design and statistics**<br><br>Full details of the experimental design and statistical methods used should be given in the Methods section, as detailed in our Minimum Standards Reporting Checklist. Information essential to interpreting the data presented should be made available in the figure legends.<br><br>Have you included all the information requested in your manuscript?	Yes
**Resources**<br><br>A description of all resources used, including antibodies, cell lines, animals and software tools, with enough information to allow them to be uniquely identified, should be included in the Methods section. Authors are strongly encouraged to cite Research Resource Identifiers (RRIDs) for antibodies, model organisms and tools, where possible.<br><br>Have you included the information requested as detailed in our Minimum Standards Reporting Checklist?	Yes
**Availability of data and materials**<br><br>All datasets and code on which the conclusions of the paper rely must be either included in your submission or deposited in publicly available repositories (where available and ethically	Yes

appropriate), referencing such data using a unique identifier in the references and in the “Availability of Data and Materials” section of your manuscript.<br><br>Have you have met the above requirement as detailed in our Minimum Standards Reporting Checklist?	

Powered by Editorial Manager® and ProduXion Manager® from Aries Systems Corporation

main tex file

Click here to access/download;LaTeX - Main Document (TeX file);main.tex

```
This is pdfTeX, Version 3.14159265-2.6-1.40.21 (TeX Live 2020/W32TeX)
(preloaded format=pdflatex 2020.5.12)  8 DEC 2021 01:53
entering extended mode
 restricted \write18 enabled.
 %&-line parsing enabled.
**main.tex
(./main.tex
LaTeX2e <2020-02-02> patch level 5
L3 programming layer <2020-05-05>

! LaTeX Error: File `oup-contemporary.cls' not found.

Type X to quit or <RETURN> to proceed,
or enter new name. (Default extension: cls)

Enter file name:
! Emergency stop.
<read *>

l.11 ^^M

*** (cannot \read from terminal in nonstop modes)


Here is how much of TeX's memory you used:
 22 strings out of 480681
 490 string characters out of 5908536
 236875 words of memory out of 5000000
 15943 multiletter control sequences out of 15000+600000
 532338 words of font info for 24 fonts, out of 8000000 for 9000
 1141 hyphenation exceptions out of 8191
 14i,0n,17p,95b,10s stack positions out of
5000i,500n,10000p,200000b,80000s
!  ==> Fatal error occurred, no output PDF file produced!
```

Manuscript

Click here to access/download;Manuscript;Benjakob_etal_GigaScience_WI

OXFORD (GIGA)^n SCIENCE

GigaScience, 2021, 1–24

doi: xx.xxxx/xxxx
Manuscript in Preparation
Research

RESEARCH

Citation needed? Wikipedia bibliometrics during the first wave of the COVID-19 pandemic.

Omer Benjakob[1,2,*,§], Rona Aviram[1,3,†,§] and Jonathan Aryeh Sobel[3,4‡,§]

[1]Center for Research and Interdisciplinarity (CRI), Université de Paris, INSERM U1284, Paris, France and [2]The Cohn Institute for the History and Philosophy of Science and Ideas, Tel Aviv University, Tel Aviv, Israel and [3]Weizmann Institute of Science, Rehovot, Israel and [4]Faculty of Biomedical Engineering, Technion-IIT, Haifa, Israel

[*]omerbj@gmail.com
[†]anorona@gmail.com
[‡]jsobel83@gmail.com
[§]Contributed equally.

Abstract

Background With the COVID-19 pandemic's outbreak, millions flocked to Wikipedia for updated information. Amid growing concerns regarding an "infodemic", ensuring the quality of information is a crucial vector of public health. Investigating if and how Wikipedia remained up to date and in line with science is key to formulating strategies to counter misinformation. Using citation analyses, we asked: which sources informed Wikipedia's COVID-19-related articles before and during the pandemic's first wave (January-May 2020).

Results We found that coronavirus-related articles referenced trusted media outlets and high-quality academic sources. Regarding academic sources, Wikipedia was found to be highly selective in terms of what science was cited. Moreover, despite a surge in COVID-19 preprints, Wikipedia had a clear preference for open-access studies published in respected journals and made little use of preprints. Building a timeline of English COVID-19 articles from 2001-2020 revealed a nuanced trade-off between quality and timeliness. It further showed how preexisting articles on key topics related to the virus created a framework for integrating new knowledge. Supported by a rigid sourcing policy, this "scientific infrastructure" facilitated contextualization and regulated the influx of new information. Lastly, we constructed a network of DOI-Wikipedia articles, which showed the landscape of pandemic-related knowledge on Wikipedia and how academic citations create a web of shared knowledge supporting topics like COVID-19 drug development.

Conclusions Understanding how scientific research interacts with the digital knowledge-sphere during the pandemic provides insight into how Wikipedia can facilitate access to science. It also reveals how, aided by what we term its "citizen encyclopedists", it successfully fended off COVID-19 disinformation and how this unique model may be deployed in other contexts.

Key words: COVID-19; Wikipedia; Infodemic; sources; bibliometrics; citizen science; open science

Introduction

Wikipedia has over 130,000 different articles relating to health and medicine [1]. The website as a whole, and specifically its medical and health articles, like those about diseases or drugs, are a prominent source of information for the general public [2]. Studies of readership and editorship of health-related articles reveal that medical professionals are active consumers of Wikipedia and make up roughly half of those involved in editing these articles in English [3, 4]. Research conducted into

Compiled on: December 8, 2021.
Draft manuscript prepared by the author.

the quality and scope of medical content deemed Wikipedia “a key tool for global public health promotion” [4, 5]. Others have found that in terms of content errors Wikipedia is on par with academic and professional sources even in fields like medicine [6]. Meanwhile, a metastudy of Wikipedia’s medical content (specifically those articles overseen by the WikiProject Medicine, a volunteer run group of editors which focuses on ensuring quality of health related articles) found it to be a prominent health information resource for experts and non-experts alike [7]. With the WHO labeling the COVID-19 pandemic an "infodemic" [8], and disinformation posing a public health threat, a closer examination of Wikipedia and its references during the pandemic is merited.

Wikipedia’s "COVID-19 pandemic" article was among the most viewed in 2020 [9] - with a peak interest during the first wave. Researchers from different disciplines have looked into citations in Wikipedia and done bibliometric analyses of it - for example, asking if open-access papers are more likely to be cited in Wikipedia [10]. While anecdotal research has shown that Wikipedia and its academic references can mirror the growth of a scientific field [11], few have researched the coronavirus and Wikipedia. Research focused on Wikipedia and COVID-19 has shown both that traffic to Wikipedia’s coronavirus articles reflected public interest in the pandemic [12], and that these articles cite a representative sample of COVID-19 research [13]. However, to our knowledge, no research has yet focused on the bibliometrics of COVID-19 references on Wikipedia - be they popular or academic. These sources serve as the bridge between science and trusted facts on the one hand, and public interest on the other. Examining their dynamics on Wikipedia is key for understanding the online knowledge ecosystem during a crucial phase of the pandemic and infodemic.

The aim of the present study was to provide a comprehensive bibliometric analyses of English Wikipedia’s COVID-19 articles during the pandemic’s first wave. To characterize the scientific literature as well as general media sources supporting the encyclopedia’s coverage of the COVID-19 we performed citation analyses of the references used in Wikipedia’s coronavirus articles. We did this along three axes: the relevant articles’ references at the end of the first wave, their historical trajectory, and their network interaction with other Wikipedia articles on this topic.

Material and Methods

Using citations as a metric for gauging the scientificness of Wikipedia articles along these three aforementioned axes allowed us to characterize the references and understand the pandemic’s effect on them. It also allowed us to ask what was the percentage of academic citations among any given article and what shifts they underwent during the period researched. This allowed us to gain an historical perspective on the scientific infrastructure supporting them, gauging the amount of time that passed between a scientific study’s publication and its being referenced on Wikipedia. Moreover we explored Wikipedia’s articles’ revisions (i.e their edit history) and co-citations. This allowed us to gain insight on the representation of COVID-19 knowledge on Wikipedia and its growth since the creation of the digital encyclopedia in 2001 and up until 2020. Though predominantly qualitative, for some selected articles we also examined the different claims the citations were used to support at different stages, and reviewed some of the textual changes that articles underwent in wake of the coronavirus outbreak, to provide anecdotal context for our findings.

Corpus Delimitation

Throughout the text, we used "articles" to denote Wikipedia entries, and "papers" for academic studies referenced on Wikipedia articles. Digital Object Identifiers (DOIs) were used to identify academic sources among the references found within any given Wikipedia article. To delimit the corpus of Wikipedia COVID-19-articles containing DOIs, two different strategies were applied (Supplementary figure S1A). Every Wikipedia article affiliated with the official WikiProject COVID-19 (a volunteer-run task force overseeing more than 1,500 articles during the period analyzed) was scraped using an R package specifically developed for this study, *WikiCitationHistoRy* [14]. In combination with the *WikipediR* R package [15], which was used to retrieve the list of actual articles covered by the COVID-19 project, our *WikiCitationHistoRy* R package was used to extract DOIs from their text and thereby identify Wikipedia pages containing academic citations. Simultaneously, we also searched the EuroPMC database, using *COVID-19*, using *SARS-CoV2*, *SARS-nCoV19* as keywords to detect scientific studies published about this topic. Thus, 30,000 peer-reviewed papers, reviews, and preprints were retrieved. This set was compared to the DOI citations extracted from the entirety of the English Wikipedia dump of May 2020 (~860,000 DOIs) using *mwcite* [16]. Thus, Wikipedia articles containing at least one DOI citation related to COVID-19 were identified - either from the EuroPMC search or through the specified Wikipedia project. The resulting "COVID-19 corpus" comprised a total of 231 Wikipedia articles - all related to COVID-19 which included at least one academic source. In this study, the term "corpus" describes this body of Wikipedia "articles", and "sets" is used to describe a collection of "papers" (i.e. DOIs) and their related bibliographic information.

DOI Content Analysis and Sets Comparison

The analysis of DOIs led to the categorization of three DOI sets: 1) the COVID-19 Wikipedia set, 2) the EuroPMC 30K search and 3) the Wikipedia dump of May 2020. For the dump and the COVID sets, the latency (see below) was computed, and for all three sets we retrieved their scientific citations count (the number of times the paper was cited in scientific literature), their Altmetric score, as well as the papers’ authors, publishers, journal, source type (preprint server or peer-reviewed publication), open-access status, title and keywords. In addition, in the COVID-19 Wikipedia corpus the DOI set’s citation count on Wikipedia were also analysed to help gauge the importance of the sources within the online encyclopedia.

Text Mining, Identifier Extraction and Annotation

From the COVID-19 corpus, DOIs, PMIDs, ISBNs, and URLs (Supplementary figure S1B) were extracted using a set of regular expressions from our R package. Moreover *WikiCitationHistoRy* [14] allows the extraction of other sources such as tweets, press releases, reports, hyperlinks and the *protected* status of Wikipedia pages (on Wikipedia, pages can be locked to public editing through a system of "protected" statuses). Subsequently, several statistics were computed for each Wikipedia article and information for each of their DOIs was retrieved using *Altmetrics* [17], *CrossRef* [18] and the *EuroPMC* [19] R packages.

Visualisations and Metrics

Our R package allows the retrieval of any Wikipedia articles' content, both in the present - i.e., article text, size, reference count and users - and in the past - i.e. timestamps, revision IDs and the text of earlier versions. This package allows the retrieval of the relevant information in structured tables and helped support several data visualisations. Notably, two navigable visualisations were created for our corpus of Wikipedia articles: 1) A timeline [20] of article creation dates which allows users to navigate through the growth of Wikipedia articles over time, and 2) a network [21] linking Wikipedia articles based on their shared academic references. The package also includes a proposed metric to assess the scientificness of a Wikipedia article. This metric, called *Sci Score* (shorthand for scientific score), is defined as the ratio of academic as opposed to non-academic references any Wikipedia article includes, as such:

$$Sci_{Score} = \frac{\#DOI}{\#Reference} \quad (1)$$

Our investigation also included an analysis of the latency [11] of any given DOI citation on Wikipedia. This metric is defined as the duration (in years) between the date of publication of a scientific paper and the date of introduction of the DOI into a specific Wikipedia article, as defined below:

$$Latency = Date_{WikiIntroduction} - Date_{Publication} \quad (2)$$

All visualisations and statistics were conducted using R statistical programming language (R version 3.5.0).

Data and Code Availability Statement

Every raw data and table are available online through the Zenodo repository [22]. A beta version of the visualizations, their code and the documentation from our R package are available on the Github repositories [14, 21, 20]. Supplementary information and datasets are available in the *GigaScience* GigaDB repository [23].

Results

COVID-19 Wikipedia Articles: Well-Sourced but Highly Selective

We set out to characterize the representation of COVID-19-related research on Wikipedia. As all factual claims on Wikipedia must be supported by "verifiable sources" [24], we focused on articles' references to ask: What sources were used and what was the role of scientific papers in supporting coronavirus articles on Wikipedia? For this aim, we first identified the relevant Wikipedia articles related to COVID-19 (Supplementary figure S1A) as described in detail in the methods section. Then, we extracted relevant information such as identifiers (DOI, ISBN, PMID), references and hyperlinks (Supplementary figure S1B).

From the perspective of Wikipedia, though there were over 1.5K (1,695) COVID-19-related articles, only 149 had academic sources. We further identified an additional 82 Wikipedia articles that were not part of Wikipedia's organic corpus of coronavirus articles, but had at least one DOI reference from the EuroPMC database of over 30,000 COVID-19 related papers (30,720) (Supplementary figure S1C). Together these 231 Wikipedia articles served as the main focus of our work as they form the scientific core of Wikipedia's COVID-19 coverage. This DOI-filtered COVID-19 corpus included articles on scientific concepts, genes, drugs and even notable people who fell ill with coronavirus. The articles ranged from "Severe acute respiratory syndrome-related coronavirus", "Coronavirus packaging signal" and "Acute respiratory distress syndrome", to "Charles, Prince of Wales", "COVID-19 pandemic in North America," and concepts with social interest like "Herd immunity", "Wet market" or even public figures like "Dr. Anthony Fauci". This corpus included articles that had both scientific and social content related topics of general public interest. For example, the article for "Coronavirus", the drugs "Chloroquine" and "Favipiravir," and others with wider social interest, like the article for "Social distancing" or "Shi Zhengli", the virologist employed by the Wuhan Institute of Virology and who earned public notoriety for her research into the origins of COVID-19.

Comparing the overall set of academic papers dealing with COVID-19 to those cited on Wikipedia we found that less than half a percent (0.42%) of all the academic papers related to coronavirus made it into Wikipedia (Supplementary figure S1C). Thus, our data reveals Wikipedia was highly selective in regards to the existing scientific output dealing with COVID-19 (See supplementary dataset (1)).

We next analyzed all the references included in the complete Wikipedia dump from May 2020, using *mwcite* [16] (python package to extract references from Wikipedia dump). Thus, we could extract a total number of about 2.68 million citations (2,686,881) comprising ISBNs, DOIs, arXiv, PMID and PMC numbers (Supplementary figure S1D). Among the citations extracted were 860K DOIs and about 38K preprints IDs from arXiv, about 1.4 % of all the citations in the dump, indicating that this server also contributes sources to Wikipedia alongside established peer-review journals. These DOIs were used as a separate group that was compared with the EuroPMC 30K DOIs (30,720) and the extracted DOIs (2,626 unique DOIs) from our initial Wikipedia COVID-19 set in a subsequent analysis, thus forming the three aforementioned sets.

Analysis of the journals and academic content from the set of 2,626 DOIs that were cited in the Wikipedia COVID-19 corpus revealed a strong bias towards high impact factor journals in both science and medicine. For example, *Nature* - which has an impact factor of over 42 - was among the top cited journals, alongside *Science*, *The Lancet* and the *New England Journal of Medicine*; together these four comprised 13 percent of the overall academic references (Figure 1A). The Cochrane Library database of systematic reviews was also among the most cited academic sources, likely since the WikiProject Medicine (WPM) and Cochrane have an official partnership. Notably, the papers cited were mostly those published in high impact factor journals, and were also found to have a higher Altmetric score compared to the overall average of papers cited in Wikipedia. In other words, the papers cited on Wikipedia's COVID-19 articles were not just academically respected, but were also popular - i.e. they were shared extensively on social media such as Twitter.

Importantly, more than a third of the academic sources (39%) referenced in COVID-19 articles on Wikipedia were found to be open-access papers (Figure 1B). The relation between open-access and paywalled academic sources is especially telling when compared to Wikipedia's references writ large: About 29 percent of all academic sources on Wikipedia are open-access, compared to 63 percent in the COVID-19-related scientific literature (i.e. in EuroPMC).

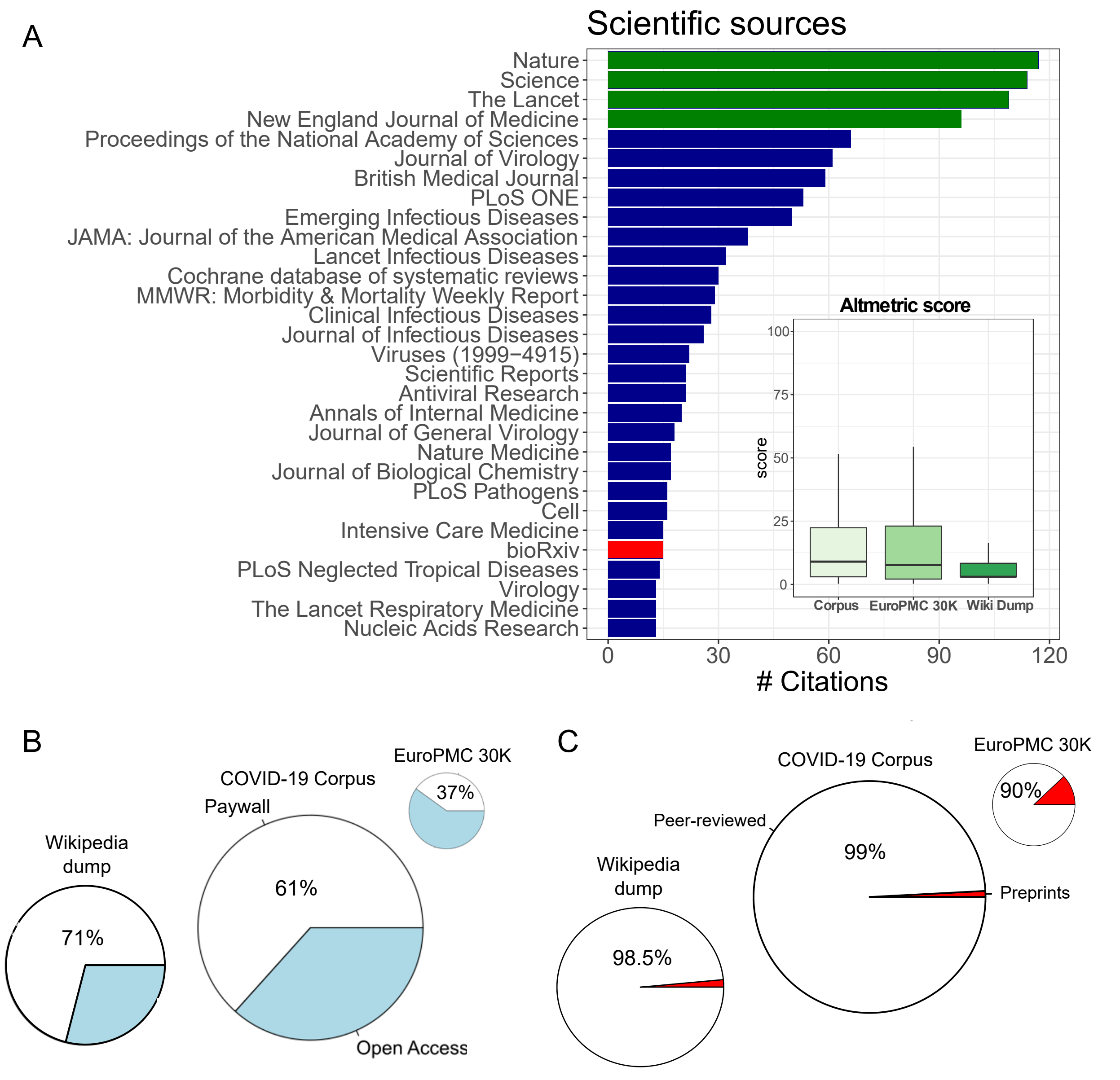

Figure 1. Characterization of scientific sources of the Wikipedia COVID-19 corpus. A) Bar plot of the most cited academic sources. Top journals are highlighted in green and preprints are represented in red. Bottom right: boxplot of Altmetrics score of the three sets: the Wikipedia COVID-19 corpus, the EuroPMC COVID-19 search, and the full Wikipedia dump as of May 2020. Comparison of the occurrence of B) open-access sources and C) preprints (MedRxiv and BioRxiv) in the three sets.

Remarkably, despite a surge in COVID-19 research being uploaded to preprint servers, we found that only a fraction of this new output was cited on Wikipedia – less than 1 percent, or 27 bioRxiv or medRxiv preprints were referenced (Figure 1C, Table S1). Among the COVID-19 preprints cited on Wikipedia was an early study on *Remdesivir* [25], a study on the mortality rate of elderly individuals [26], research on COVID-19 transmission in Spain [27] and New York [28], and research into how Wuhan's health system attempted to contain the virus [29]. This shows how non-peer-reviewed studies touched on medical, health and social aspects of the virus – with two of the preprints, for example, focusing on the benefits of contact tracing [30, 31]. The number of overall preprints was slightly lower than the general representation of preprints in Wikipedia (1.5%), but much lower than would be expected considering the fact that our academic database of EuroPMC papers had almost 3,700 preprints – 12.3 percent of the roughly 30,000 COVID-19 related papers in May 2020. Thus, in contrast to the high enrichment of preprints in COVID-19 research, Wikipedia's editors overwhelmingly preferred peer-reviewed papers to preprints. In other words, Wikipedia generally cited preprints more than it was found to on the topic of COVID-19, while COVID-19 articles cited open-access paper by more 10% (from 29% to 39%). Taken together with the bias towards high-impact journals, our data suggest that open access papers contributed significantly to Wikipedia's ability to stay both up to date and to maintain high academic standards, allowing editors to cite peer-reviewed research despite other alternatives being available.

We next focused on non-academic sources. Popular media, we found, played a substantial role in our corpus. Over 80 percent of all the references used in the COVID-19 corpus were non-academic, being either general media or websites (Figure 2A). In fact, a mere 16 percent of the over 21,000 references supporting the COVID-19 content were from academic journals. Among the general media sources used (Figure 2B-D), there was a high representation for what is termed legacy media outlets, like the *New York Times* and the *BBC*, alongside widely syndicated news agencies like *Reuters* and the *Associated Press*, and official sources like *WHO.org* and *gov.UK*. Among the most cited websites, for example, there was an interesting representation of local media outlets from countries hit early and hard by the virus, with the Italian *La Repubblica* and the *South China Morning Post* being among the most cited sites. The World Health Organization was one of the most cited publishers in the corpus of relevant articles, with more than 150 references.

A Scientific Score for Gauging Scientificness

To distinguish between the role scientific research and popular media played, we created a "scientific score" for Wikipedia articles (1). The metric is based on the ratio of academic as opposed to non-academic references any article includes. This score attempts to rank the *scientificness* of any given Wikipedia article based solely on its list of references. Ranging from 1 to 0, an article's scientific score is calculated according to the ratio of its sources that are academic (i.e. contain DOIs), so that an article with a score of 1 will have 100 percent academic references, while that with none will have a score of 0. Technically, as all of our corpus of coronavirus-related Wikipedia articles had at least one academic source in the form of a DOI, their scientific scores will always be greater than 0.

In effect, this score puts forth a metric for gauging the prominence of academic texts in any given article's reference list. Out of our 231 Wikipedia articles, 15 received a perfect scientific score of 1 (Supplementary Figure S2A). High scoring articles included the enzymes of "Furin" and "TMPRSS2" – whose inhibitor has been proposed as a possible treatment for COVID-19; "C30 Endopeptidase" – a group of enzymes also known as the "SARS coronavirus main proteinase"; and "SHC014-CoV" – a form of COVID-19 that affects the Chinese rufous horseshoe bat.

In contrast to the articles with scientific topics and even biographical articles about scientists themselves, which both had high scientific scores, those with the lowest scores (Supplementary Figure S2B) seemed to focus almost exclusively on social aspects of the pandemic or its immediate outcome. For example, the articles with the lowest scores dealt directly with the pandemic in a hyper-local context, including articles about the pandemic in Canada, North America, Indonesia, Japan or even Jersey, to name a few. Others focused on different ramifications of the pandemic, for example the "Impact of the COVID-19 pandemic on the arts and cultural heritage" or "Travel restrictions related to the COVID-19 pandemic". One of the articles with the lowest scientific score was the "Trump administration communication during the COVID-19 pandemic" which made scarce use of coronavirus-related research to inform its content, citing a single academic paper (related to laws regulating quarantine) among its 244 footnotes.

The Price of Remaining Up to Date on COVID-19

During the pandemic, there were over tens of thousands of edits to the site, with thousands of new articles being created and scores of existing ones being re-edited and recast in wake of new developments. Therefore, one could expect a rapid growth of articles on the topic, as well as a possible overall increase in the number of citations of all kinds. We sought to explore the temporal axis of Wikipedia's coverage of the pandemic to see how COVID-19 articles and their academic references developed over time and were affected by the outbreak.

First, we laid out our corpus of 231 articles across a timeline according to each article's respective date of creation (Supplementary Figure S3). An article count starting from 2001, when Wikipedia was first launched, and up until May 2020, shows that for many years there was a relatively steady growth in the number of articles that would become part of our corpus – until the pandemic hit, causing a massive peak at the start of 2020 (Figure 3A). As the pandemic spread, the total number of Wikipedia articles dealing with COVID-19 and supported by scientific literature almost doubled – with a comparable number of articles being created before and after 2020 (134 and 97, respectively), (Figure 3A, Supplementary Figure S3) .

The majority of the pre-2020 articles were created relatively early – between 2003 and 2006, likely linked to a general uptick in creation of articles on Wikipedia during this period. For example, the article for (the non-novel) "coronavirus" has existed since 2003, the article for the medical term "Transmission" and that of "Mathematical modeling of infectious diseases" from 2004, and the article for the "Coronaviridae" classification from 2005. Articles opened in this early period tended to focus on scientific concepts – for example those noted above or others like "Herd immunity". Conversely, the articles created post-pandemic during 2020 tended to be hyper-local or hyper-focused on the virus' effects and social ramifications. Therefore, we collectively term the first group Wikipedia's "scientific infrastructure", as they allowed new scientific information to be added into existing articles, alongside the creation of new ones focusing on the pandemic's social significance.

The pre-pandemic articles tended to have a high scientific

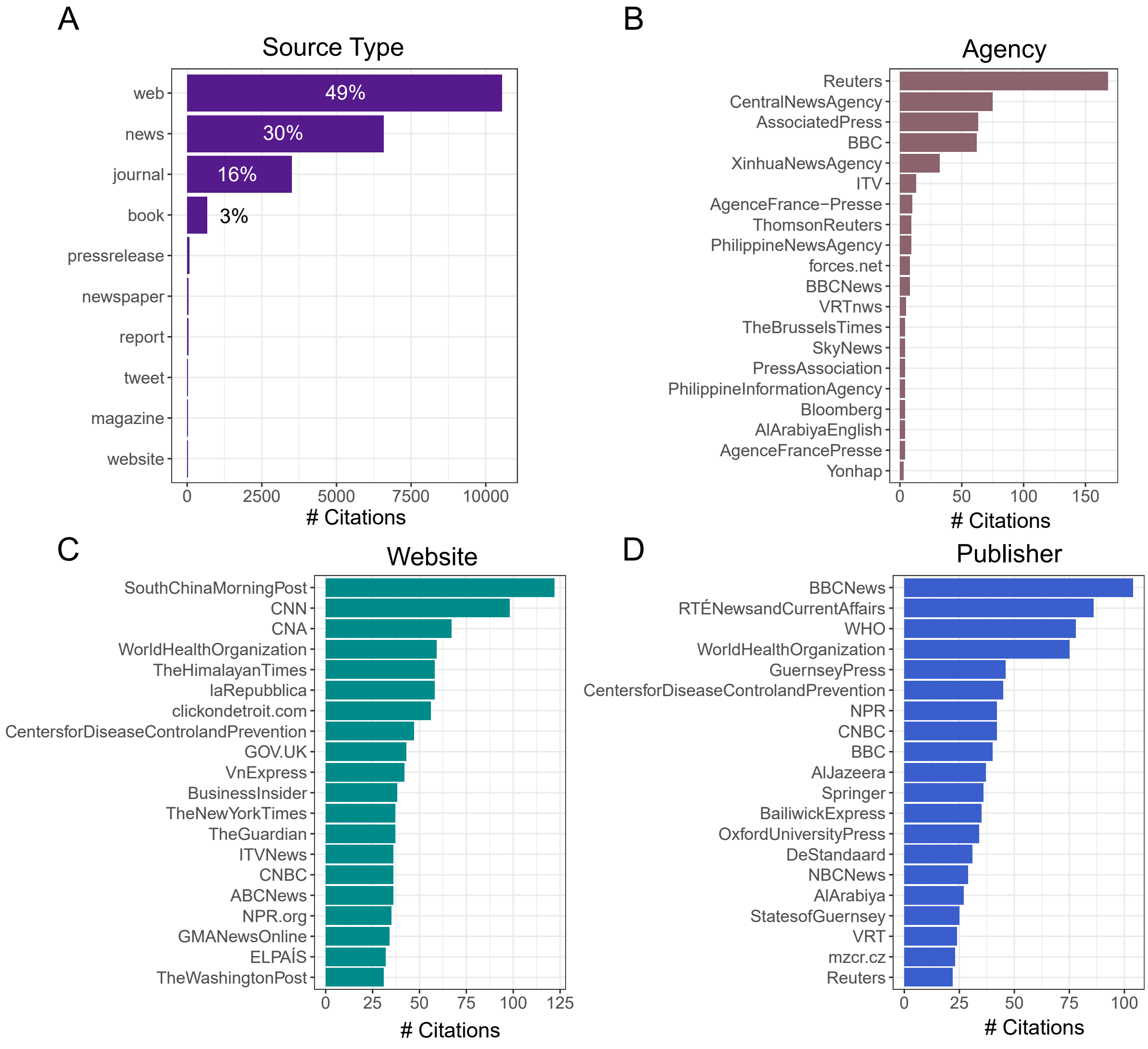

Figure 2. Top sources used in the Wikipedia COVID-19 corpus: A) source types, B) news agencies, C) websites, and D) publishers form the COVID-19 corpus sources (per Wikipedia's citation template terminology).

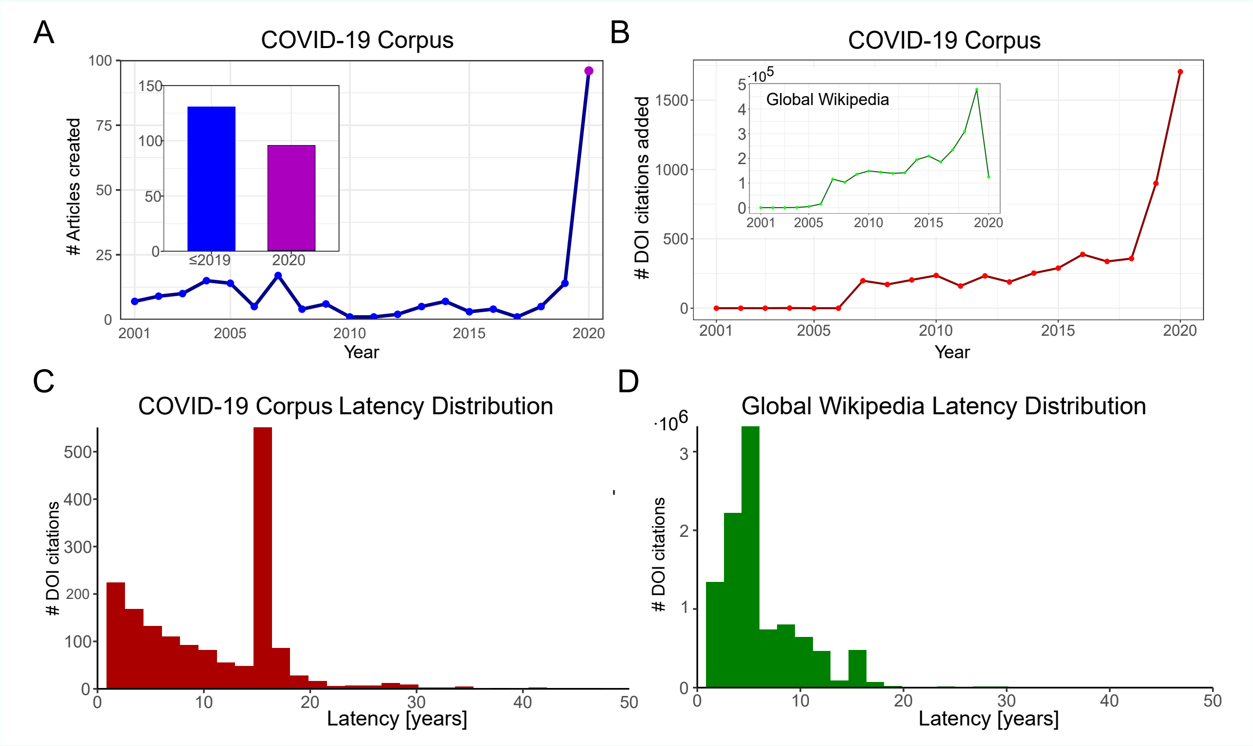

Figure 3. Historical perspective of the Wikipedia COVID-19 corpus. A) COVID-19 article creation per year; inset: number of articles created before and after 2020. B) Scientific citations added per year to the COVID-19 corpus and globally in Wikipedia (inset). Latency distribution of scientific papers C) in the COVID-19 corpus and D) the Wikipedia dump. See Supplementary FigureS3 and here for an interactive version of the timeline.

score - for example, "Chloroquine", which has been examined as a possible treatment for COVID-19. This article is one of many that underwent a shift in content in wake of the pandemic, seeing both a surge in traffic and a surge in editorial activity (Supplementary Figure S4). Per a subjective reading of this article's content and the editorial changes it underwent during this period, much of the scientific content that was present pre-pandemic was found to have remained intact, with new coronavirus-related information being integrated into the framework provided by existing content. The same occurred with many social concepts retroactively affiliated with COVID-19. Among these we can note the articles for "Herd immunity", "Social distancing" and the "SARS conspiracy theory" that also existed prior to the outbreak and served as part of Wikipedia's scientific infrastructure, allowing new information to be contextualized.

In addition to the dramatic rise in article creation during the pandemic, there was also a rise in the overall number of references in articles affiliated with COVID-19 on Wikipedia (Figure 3B). In fact, the number of DOIs added to these articles grew almost six-fold post-2020 - from roughly 250 to almost 1,500 citations. Though most of the citations added were not just academic ones, with URLs overshadowing DOIs as the leading type of citation added, the general rise in citations can be seen as indicative of scientific literature's prominent role in COVID-19 when taking into account that general trend in Wikipedia: The growth rate of references on COVID-19 articles was generally static until the outbreak; but on Wikipedia writ large references were on a rise since 2006. The post-2020 surge in citations was thus both academic and non-academic (Supplementary Figure S5A).

One could hypothesize that a rapid growth of articles dedicated to coronavirus would translate to an overall decrease in the presence of academic sources, as Wikipedia can create newer articles faster than academic research can be published on current events.

Examining the date of publication of the peer-reviewed studies referenced on Wikipedia shows that new COVID-19 research was cited alongside papers from previous years and even the previous century, the oldest being a 1923 paper titled the "The Spread of Bacterial Infection. The Problem of Herd-Immunity." [32]. Overall, among the papers referenced on Wikipedia were highly cited studies, some with thousands of citations (Table 2), but most had relatively low citation counts (median of citation count for a paper in the corpus was 5). Comparing between a paper's date of publication and its citation count reveals there is low anti-correlation (-0.2) but highly significant between the two (Pearson's product-moment correlation test p-value $< 10^{-15}$, Figure S5B). This suggest that on average older scientific papers have a higher citation count; unsurprisingly, the more time that has passed since publication, the bigger the chances a paper will be cited.

Comparing the pre-and post-2020 articles' scientific score reveals that on average, the new articles had a mean score of 0.14, compared to the pre-2020 group's mean of 0.48 and the overall average of 0.3 (Supplementary Figure S5C). Reading the titles of the 2020 articles to glean their topic and reviewing their respective scientific score can also point to a generalization: the more scientific an article is in topic, the more scientific its references are - even during the pandemic. This means that despite the dilution at a general level during the first months of 2020, articles with scientific topics created during this period did not pay that heavy of an academic price to stay up to date.

How did Wikipedia manage to maintain the quality of aca-

demic sourcing throughout the first wave of the pandemic? One possible explanation is that among the academic papers added to Wikipedia in 2020 were also papers published prior to this year if not a long time before. To investigate this hypothesis we used the latency metric (namely, the lag between a paper's publication and its integration into Wikipedia, see equation (2)). We found the mean latency of Wikipedia's COVID-19 content to be 10.2 years (Figure 3C), slower than Wikipedia's overall mean of 8.7 (Figure 3D). In fact, in the coronavirus corpus we observed a peak in latency of ~17 years - with over 500 citations being added to Wikipedia 17 years after their initial academic publication - almost twice as slow as Wikipedia's average. Interestingly, this time frame corresponds to the SARS outbreak (SARS-CoV-1) in 2002-2004, which yielded a boost of scientific literature regarding coronaviruses. This suggests that while there was a surge in editing activity during this pandemic that saw papers published in 2020 added to the COVID-19 articles, a large and even prominent role was still permitted for older literature. Viewed in this light, older papers played a similar role to pre-pandemic articles, giving precedence to existing knowledge in ordering the integration new knowledge on scientific topics.

Comparing the articles' scientific score to their date of creation portrays Wikipedia's scientific infrastructure and its dynamics during the pandemic (Supplementary Figure S5C). It reveals that despite maintaining high academic standards, citing papers published in prestigious and high impact factor journals, the need to stay up to date with COVID-19 research did come at some cost: most of the highest scoring articles were ones created pre-pandemic (especially during 2005-2010) and newer articles generally had a lower scientific score (Supplementary Figure S5C).

Networks of COVID-19 Knowledge

To further investigate Wikipedia's scientific sources and the infrastructure it provided, we built a network of Wikipedia articles linked together based on their shared academic (DOI) sources. We filtered the list of papers (extracted DOIs) in order to keep those which were cited in at least two different Wikipedia articles, and found 179 that fulfilled this criteria and were mapped to 136 Wikipedia articles in 454 different links (Figure 4, supplementary data (2)). This allowed us to map how scientific knowledge related to COVID-19 played a role not just in specific articles created during or prior to the pandemic, but actually formed a web of knowledge that proved to be an integral part of Wikipedia's scientific infrastructure. Similar to the timeline described earlier and as a subset of our COVID-19 corpus, Wikipedia articles belonging to this network included those dealing with people, institutions, regional outcomes of the pandemic and scientific concepts - for example those regarding the molecular structure of the virus or the mechanism of infection ("C30 Endopeptidase", "Coronaviridae", and "Airborne disease"). It also included a number of articles regarding the search for a potential drug to combat the virus or other possible interventions against it, with topics like social distancing, vaccine development and drugs in current clinical trials.

Interestingly, we observed six prominent Wikipedia articles as key nodes in this network. These shared multiple citations with many other pages through DOI connections (nodes with an elevated degree). Four of these six major nodes had a distinct and broad topic: "Coronavirus", which focused on the virus writ large; "Coronavirus disease 2019", which focused on the pandemic; and "COVID-19 drug repurposing research" and "COVID-19 drug development." The first two articles were key players in how Wikipedia presented its coverage of the pandemic to readers: both were linked to from the main coronavirus article ("Coronavirus disease 2019") which was placed on the English Wikipedia's homepage in a community-led process known as "In the News", which showcases select articles on current events on the website's homepage. Later on, alongside this process led by the volunteers of the WikiProject COVID-19 task force, the Wikimedia Foundation (the WMF is the non-profit that oversees the Wikipedia project) also issued a directive to place a special banner referring to the "Coronavirus disease 2019" article on the top of every single article in English, driving millions to the article and to subsequent articles linking out from it. As noted, these articles - "Coronavirus disease 2019" and the articles linking out from it - were part of our DOI network. The fact that this central article shared citations with other articles that linked out from it, as described in our network, highlights the interconnecting role academic citations played on Wikipedia's COVID-19 coverage, allowing academic sources to support both popular and scientific articles and providing the public with access to high-quality sources in different contexts.

The two remaining nodes were similar and did not prove to be distinctly independent concepts, but rather interrelated ones, with the articles for "Severe acute respiratory syndrome-related coronavirus" and "Severe acute respiratory syndrome coronavirus" each appearing as their own node despite their thematic overlap. It is also interesting to note that four of the six Wikipedia articles that served as the respective centers of these groups were locked to public editing as part of the protected page status (see supplementary data (3)). These were all articles linked to the WPM or, at a later stage, to the specific offshoot project set up as a task force to deal with COVID-19.

The main themes that emerge from the network is that of COVID-19 related drugs and of the disease itself. Unlike articles relating to the effect of the pandemic, which as shown above were predominantly based on popular media, these two were topics that did require scientific basing to be reliable. Reliability in this context is defined on Wikipedia as accordance with its MEDRS policy - shorthand for "MEDical Reliable Sources". The sourcing policy, which is Wikipedia's most rigid, bans primary sources. Instead, MEDRS demands medical and health claims cite meta-analysis or secondary sources that provide an overview of existing research or multiple-case-study clinical trials [33]. This policy is facilitated by the WPM's aforementioned partnership with the Cochrane Library. The fact that popular articles like "Coronavirus disease 2019" or "COVID-19 drug development" shared academic citations with other articles underscores the important role academic publications play on Wikipedia, creating the web of knowledge our network describes. Furthermore, it highlights how the editing community's centralized efforts (both articles were locked (supplementary dataset (3)) and fell under the oversight of Wikipedia's volunteer-run COVID-19 task force) allowed certain academic studies to find a role both in popular articles and in scientific articles linking out from them.

In our network analysis, an additional smaller group of nodes (with a lower degree) was also found. It had to do almost exclusively with China-related issues. As such, it exemplified how Wikipedia's sourcing policy - which has an explicit bias towards peer-reviewed studies and is enforced exclusively by the community - may play a key role in fighting disinformation. For example, the academic paper that was most cited in Wikipedia's COVID-19 articles was a paper published in Nature in 2020, titled "A pneumonia outbreak associated with a new coronavirus of probable bat origin" (Table

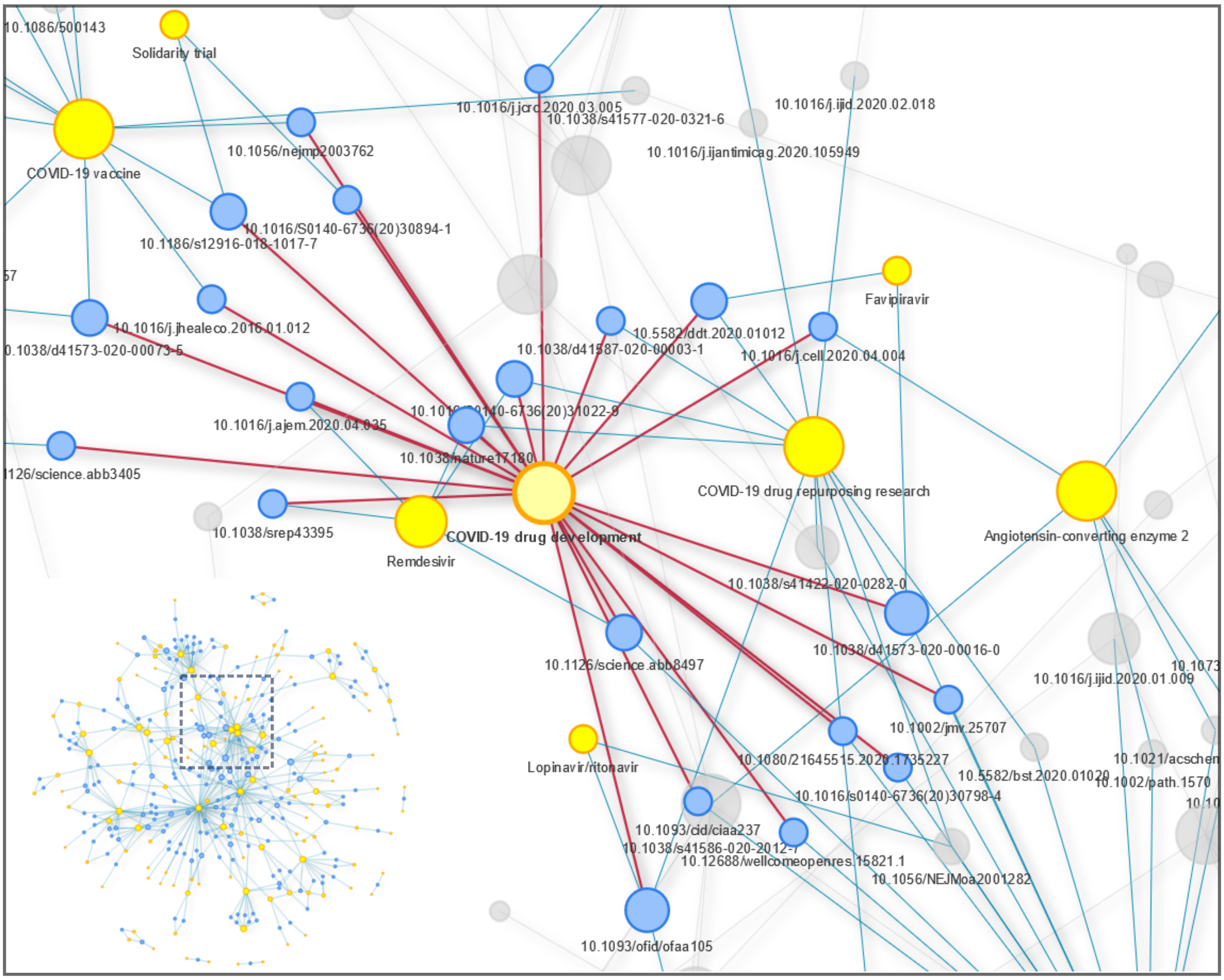

Figure 4. Network of articles-scientific papers (DOI) in the Wikipedia COVID-19 corpus. A network mapping scientific papers (with DOIs) cited in more than one article in the Wikipedia COVID-19 corpus was constructed. This network is composed of 454 edges, 179 DOIs (blue) and 136 Wikipedia articles (yellow). Nodes represent articles and their size is proportional to the number of connections. A zoom in on the cluster of Wikipedia articles dealing with COVID-19 drug development is depicted here for illustrative purposes. For clarity, edges marked in red indicate those connecting the DOIs cited directly in the "COVID-19 drug development" article and edges marked in blue for those connecting these DOIs to other articles citing them. See here for an interactive version of the network. See Supplementary dataset (2).

3). This paper was referenced in eight different Wikipedia articles, two among which dealt directly with scientific topics – "Angiotensin-converting enzyme 2" and "Severe acute respiratory syndrome coronavirus 2" – and two dealing with what can be termed para-scientific terms linked to COVID-19 – the "Wuhan Institute of Virology" and "Shi Zhengli". This serves to highlight how contentious issues with a wide interest for the public – in this case, the origin of the virus – receive increased scientific support on Wikipedia, perhaps as result of editors attempting to fend off misinformation supported by lesser, non-academic sources. Of the five most cited papers inside the COVID-19 corpus (Table 3) three focused specifically on either bats or the virus' animal origins, and another focused on its spread from Wuhan. Interestingly, one of the 27 preprints cited (Table 1) was also the first study to suggest the virus' origin lay with bats [34].

Taken together with the previous findings regarding high quality academic sources, centralized efforts in the form of locking articles did not just allow the enforcement of a rigid sourcing policy by the task force's editors, but also created a filtered knowledge funnel of sorts, which harnessed Wikipedia' preexisting infrastructure of articles, mechanisms and policies to allow a regulated intake of new information as well as the creation of new articles, both based on existing research.

Discussion

In the wake of COVID-19 pandemic, characterizing scientific research on English-language Wikipedia and understanding the role it plays is both important and timely. Millions of people – both medical professionals and the general public – read about health online [1]. Research has shown traffic to Wikipedia articles follows topics covered in the news [35] – a dynamic which played out during the pandemic's first wave [12]. Moreover, scientometric research has shown that academic research follows a similar pattern – with a surge of new studies during a pandemic followed by a decrease after it wanes [36]. During a pandemic the risk of disinformation on Wikipedia's content is more severe, as was during the Zika and SARS outbreaks [37]. Thus, throughout the outbreak of the COVID-19 pandemic, the threat was hypothetically increased: as a surge in traffic to Wikipedia articles, research has found, often translates into an increase in vandalism [38]. Moreover, research into medical content on Wikipedia found that people who read health articles on the open encyclopedia are more likely to hover over or thus possibly read their references [39].

Particularly in the case of the coronavirus outbreak, the content on Wikipedia could have taken on potentially lethal consequences as the pandemic was deemed to be an *infodemic*, and false information related to the virus was deemed a real threat to public health by the UN and WHO [8]. So far, most research into Wikipedia has revolved either around the quality, readership or editorship of its health articles – or about references and sourcing in general. Meanwhile, research on Wikipedia and COVID-19 has focused almost exclusively on editing patterns and users behaviors [12], or the representativity of academic citations [13]. Therefore, we deployed a comprehensive bibliometric analyses of COVID-19-related Wikipedia articles – focusing on articles' text and sources, their growth over time and their network relations.

Perhaps counter-intuitively, we found that despite the traffic surge, these articles relied on high quality sources, from both popular media and academic literature. Though the proportion of academic references in newly created articles did decrease in comparison to the period before the pandemic (resulting in a lower scientific score), we found that they still played a prominent role and that high editorial standards were generally maintained, utilizing several unique solutions which we will now attempt to outline and discuss.

One possible key to Wikipedia's success had to do with the existence of centralized oversight mechanisms by the community of editors that could be quickly and efficiently deployed. In this case, the existence of the WikiProject Medicine – one of Wikipedia's oldest community projects – and the formation of a specific COVID-19 task force in the form of WikiProject COVID-19, helped harness exiting editors and practices like locking articles to safeguard quality across large swaths of articles and thus enforce a relatively unified sourcing policy on those dealing with both popular and scientific aspects of the virus.

In general, all factual claims on Wikipedia need to be supported by a verifiable source. Specifically, biomedical articles affiliated with the WPM are bound by a specific policy known as MEDRS (which requires meta-analysis or secondary sources for medical content [33]). However, the mere existence of this policy does not necessarily mean it is respected. Our findings indicate that this policy, aided by the infrastructure provided by the community to enforce it, likely played a key role in regulating the quality of coronavirus articles. One mechanism used generally by the WPM to enforce the MEDRS sourcing standards and specifically deployed by the COVID-19 task force during the pandemic was locking articles to public editing (protected pages, supplementary dataset (3)). This is a technique that is used to prevent vandalism on Wikipedia [40] and is commonly used when news events drive large amounts of new readers to specific Wikipedia articles, increasing the risk of substandard sources and content being added into the article by editors unversed in Wikipedia's standards. This ad hoc measure of locking an article, deployed by a community vote on specific articles for specific amounts of time, prevents anonymous editors from being able to contribute directly to an article's text and forces them to work through an experienced editor, thus ensuring editorial scrutiny. This measure is in line with our findings that many of the COVID-19 network central nodes were locked articles.

Another possible key to Wikipedia's ability to maintain high quality sources during the pandemic was the existence of a specific infrastructure related directly to sourcing that could be enforced by the volunteer task force. The WPM has formed institutional-level partnerships to provide editors with access to reputable secondary sources that are in line with the MEDRS policy on medical and health topics – namely through its cooperation with the Cochrane Library. The Cochran Reviews' database is available to Wikipedia's medical editors and it offers them access to systematic literature reviews and meta-analyses summarizing the results of multiple medical research studies [41]. As well as the existence of this database on medical content, the practice of providing access to high-quality sources was also deployed specifically in regards to coronavirus in the form a list of "trusted" sources provided to volunteers by the task force on its project page. Alongside Cochrane studies, the WHO, for example, was given special status and preference [42]. This was evident in our results, with Cochrane sourcing being prominent, and the WHO being found to be among the most cited publishers on the COVID-19 articles. Also among the most cited scientific sources were others that were promoted by the task force as preferable sourcing for COVID-19 content: for example, *Science*, *Nature* and *The Lancet*. This indicates that the list of sources recommended by the task force were actually utilized by the volunteers and thus underscores the connection

between our findings and the existence of a centralized community effort.

This was also true for non-academic sources: Among general media sources that the task force endorsed were *Reuters* and the *New York Times*, which were also prominently represented in our findings. As each new edit to any locked COVID-19 article needed to be vetted by an experienced volunteer from the task force before it could go online within the body of an article's text, the influx of new information being added was slowed down and regulated; the source list thus allowed an especially strict sourcing policy to be rigorously implemented across thousands of articles. This was true despite the fact that there is no academic verification for volunteers - in fact, research suggests that less than half of Wikipedia's editors focused on health and medical issues are medical professionals [3, 4] - meaning that the task forces and its list of sources allowed non-experts to enforce academic-level standards.

This dynamic was also evident within articles with purely scientific content. Despite a deluge of preprints (both in general in recent years and specifically during the pandemic [43, 44]), in our analysis, non-peer-reviewed academic sources did not play a key role on Wikipedia's coronavirus content, while open access papers did. Therefore, one could speculate that our finding that open-access papers were disproportionately cited may provide an explanation - with academic quality trumping speed, and editors opting against preprints and preferring published studies instead. Previous research has found open-access papers are more likely to be cited on Wikipedia by 47 percent [10] and nearly one-third of the Wikipedia citations link to an open-access source [45]. Here we also saw that open-access was prevalent in Wikipedia and even more so on COVID-19 articles. This, we suggest, allowed Wikipedia's editors (expert or otherwise) to keep articles up to date without reverting to non-peer-reviewed academic content. This, one could suggest, was likely facilitated or at least aided by the decision by academic publications' like *Nature* and *Science* to lift their paywall and open public access to all of their COVID-19-related research papers, both past and present.

In addition to the communal infrastructure's ability to regulate the addition of new information and maintain quality standards over time, another facet we found to contribute to Wikipedia's ability to stay accurate during the pandemic is what we term its scientific infrastructure. Research on Wikipedia articles' content has shown that the initial structuring of information on a given article tends to dictate its development in later stages, and that substantial reorganizations gradually decrease over time [46]. A temporal review of our articles and their citations showed that the best-sourced articles - those with the highest scientific score that formed the scientific backbone of Wikipedia's COVID-19 content - were those created from 2005 and until 2010. These, we argue, formed Wikipedia's scientific infrastructure, which regulated the intake of new knowledge into Wikipedia.

Our network analysis reflects the pivotal role preexisting content played in contextualizing the science behind many popular concepts or those made popular by the pandemic. Preexisting content in the form of Wikipedia articles, policies, practices, and academic research served as a framework that helped regulate the deluge of new information, allowing newer findings to find a place within Wikipedia's existing network of knowledge. Future work on this topic could focus on the question of whether this dynamic changed as 2020 progressed and, at a later time, on how contemporary peer-reviewed COVID-19-related research that was published during the pandemic's next waves would be integrated into these articles.

Previous research has suggested that in terms of content errors Wikipedia is on par with academic and professional sources even in fields like medicine [6]. A recent meta-analysis of studies about medical content on Wikipedia found that despite the prominent role Wikipedia plays for the general public, health practitioners, patients and medical students, the academic discourse around Wikipedia within the context of health is still limited [7]. This indicates that academic publications and scientists are lagging on embracing it and its benefits. A change in this regard could help improve Wikipedia's content and even introduce new editors with academic background into the fold, which would further improve quality and timeliness.

"Open" science practices that go beyond open access, for instance citizens scientists and open data, can be translated to other contexts. In this regard, much like citizen scientists help support institutional science [47], Wikipedia's editors may be regarded as citizen encyclopedists [11]. Viewed as such, Wikipedia's citizen encyclopedists can play the same role communicating science that citizen scientists play in creating science. As previous citizen science projects have taught us [48], for that to work, citizens need scientists to provide the framework for non-expert contributions [49, 50]. As this study indicates, a similar infrastructure can be seen to exist on Wikipedia for encyclopedic (as opposed to scientific) work. Thus, should the cooperation between the scientific and Wikipedia communities increase, it could be utilized for other contexts as well.

Our findings outline ways in which Wikipedia managed to fend off disinformation and stay up to date. With Facebook and other social media giants struggling to implement both technical and human-driven solutions against medical disinformation from the top down, it seems Wikipedia's dual usage of established science and an open community of volunteers, provides a possible model for how this can be achieved - a valuable goal during an infodemic. Some have already suggested that the American Center for Disease Control should adopt Wikipedia's model to help communicate medical knowledge [51]. In October 2020, the WHO and WMF announced they would cooperate to make critical public health information available via an open licence. This means that in the near future, the quality of Wikipedia's coverage of the pandemic will very likely increase just as its role as central node in the network of knowledge transference to the general public becomes increasingly clear.

Wikipedia's main advantage is in many ways its largest disadvantage: its open format which allows a large community of editors of varying degrees of expertise to contribute. This can lead to large discrepancies in article quality and inconsistencies in the way editors add references to articles' text [45]. We tried to address these limitations using technical solutions, such as regular expressions for extracting URLs, hyprelinks, DOIs and PMIDs. In this study, which was limited to English, we retrieved most of our scientific literature metadata using Altmetrics [52, 17], EuroPMC [19] and CrossRef [18] R APIs. However the content of the underlying databases is not always accurate, and at a technical level, this method was not without limitations. For example, we could not retrieve all of the extracted DOIs' metadata. Moreover, information regarding open access (among others) varied with quality between the APIs [53]. In addition, our preprint analysis was mainly focused on MedRxiv and BioRxiv which have the benefit of having a distinct DOI prefix. These collections make up the majority of preprints. However, others may also exist. Unfortunately, we found no better solution to annotate preprints from the extracted DOIs.

Preprint servers do not necessarily use the DOI system [54] (i.e ArXiv) and others share DOI prefixes with published papers (for instance the preprint server used by The Lancet). Moreover, we developed a parser for general citations (news outlets, websites, publishers), and we could not avoid redundant entries (i.e "WHO", "World Health Organisation"). In addition, our method to delimit the COVID-19 corpus focused on medical content (EuroPMC search) and may explain why we found predominately biomedical and health studies. Using DOI filtering on Wikipedia's coronavirus articles should have equally led us to find papers from the social sciences - should those have been cited in this context. However, it seems that as these socially focused articles do not fall under the MEDRS sourcing policy, there was less if any use of academic studies, resulting in a low scientific score, thus further highlighting the importance of this policy in enforcing academic standards on the open encyclopedia's articles.

Finally, as Wikipedia is constantly changing, some of our conclusions are bound to change. Our study is limited to focus on the pandemic's first wave and its history on English Wikipedia alone, a crucial arena for examining the dynamics of knowledge online at a pivotal time frame. As these findings regarding the first wave were the result of a robust community effort that utilized English Wikipedia's policies and mechanisms to safeguard existing content and regulate the creation of new content, it may be specific to English Wikipedia and its community. Nonetheless, it seems safe to speculate that at least on English Wikipedia, similar processes will continue to take place in the future as new textual additions are made to the open encyclopedia. In fact, one could speculate that as more time passes from the first wave, the newer post-pandemic articles that had low scientific scores will undergo a form of review and have their sources improved as newer research becomes more readily available. Studying the second wave - for example, shifts in the scientific score overtime - and understanding how encyclopedic content written during the first wave changed over the next year could very instructive. Analyses of coronavirus articles indicated that at least on science, medical and health topics - especially those in the news and driving public interest - Wikipedia's methods for safeguarding its standards withstood the test. Perhaps as more academic research regarding the virus passes review and is published in 2021 and in the coming years, the ability of Wikipedia to reduce latency on this topic without having to compromise its scientificness will increase. Moreover, our findings hint that should journals open access to research in other fields, it may help Wikipedia cite even more peer reviewed research instead of media sources or preprints. Thus, with the help of community enforcement, like that seen during the first wave of the pandemic, Wikipedia should be able to succeed in other fields as well.

In summary, our findings reveal a trade off between timeliness and scientificness in regards to scientific literature. Most of Wikipedia's COVID-19 content was supported by references from highly trusted sources - but with the pandemic's breakout, these were more from the general media than from academic publications. That Wikipedia's COVID-19 articles were based on respected sources in both the academic and popular media was found to be true even as the pandemic and number of articles about it grew. Our investigation further demonstrates that despite a surge in preprints about the virus and their promise of cutting-edge information, Wikipedia preferred published studies, giving a clear preference to open-access studies. A temporal and network analyses of COVID-19 articles indicated that remaining up-to-date did come at a cost in terms of quality. It also showed how preexisting content - both in the form of pre-pandemic articles and papers - helped regulate the flow of new information into existing articles. In future work, we hope the tools and methods developed here will be used to examine how these same articles fared over the entire span of 2020, as well as helping others use them for research into other topics on Wikipedia. We observed how Wikipedia used volunteer-editors to enforce a rigid sourcing standards - and future work may continue to provide insight into how this unique method can be used to fight disinformation and to characterize the knowledge infrastructure in other arenas.

Acknowledgments

J.S. is a recipient of the Placide Nicod foundation, and R.A. is a recipient of the Azrieli Foundation fellowship. We are grateful for their financial support.

References

1. Heilman JM, West AG. Wikipedia and medicine: quantifying readership, editors, and the significance of natural language. Journal of medical Internet research 2015;17(3):e62.
2. Lavsa SM, Corman SL, Culley CM, Pummer TL. Reliability of Wikipedia as a medication information source for pharmacy students. Currents in Pharmacy Teaching and Learning 2011;3(2):154–158.
3. Allahwala UK, Nadkarni A, Sebaratnam DF. Wikipedia use amongst medical students–new insights into the digital revolution. Medical teacher 2013;35(4):337–337.
4. Heilman JM, Kemmann E, Bonert M, Chatterjee A, Ragar B, Beards GM, et al. Wikipedia: a key tool for global public health promotion. Journal of medical Internet research 2011;13(1):e14.
5. Herbert VG, Frings A, Rehatschek H, Richard G, Leithner A. Wikipedia–challenges and new horizons in enhancing medical education. BMC medical education 2015;15(1):32.
6. Jemielniak D. Wikipedia: Why is the common knowledge resource still neglected by academics? GigaScience 2019;8(12):giz139.
7. Smith DA. Situating Wikipedia as a health information resource in various contexts: A scoping review. PloS one 2020;15(2):e0228786.
8. WHO, Novel Coronavirus (2019-nCoV): situation report, 13. World Health Organization (WHO); 2020.
9. Wikipedia, Wikipedia:2020 Top 50 Report; 2020. https://en.wikipedia.org/wiki/Wikipedia:2020_Top_50_Report.
10. Teplitskiy M, Lu G, Duede E. Amplifying the impact of open access: Wikipedia and the diffusion of science. Journal of the Association for Information Science and Technology 2017;68(9):2116–2127.
11. Benjakob O, Aviram R. A Clockwork Wikipedia: From a Broad Perspective to a Case Study. Journal of Biological Rhythms 2018;33(3):233–244.
12. Chrzanowski J, Sołek J, Jemielniak D. Assessing Public Interest Based on Wikipedia's Most Visited Medical Articles During the SARS-CoV-2 Outbreak: Search Trends Analysis. Journal of medical Internet research 2021;23(4):e26331.
13. Colavizza G. COVID-19 research in Wikipedia. Quantitative Science Studies 2020;p. 1–32.
14. JA S, Beta version of WikiCitationHistoRy R package. Github; 2021.
15. Oliver K, WikipediR, An R API wrapper for MediaWiki, optimised for the Wikimedia Foundation MediaWiki instances, such as Wikipedia. CRAN; 2017.
16. Aaron H, mwcite: Extract academic citations from

Wikipedia. Github; 2015.

17. Ram K. rAltmetric: Retrieves altmetrics data for any published paper from altmetrics.com; 2012, http://CRAN.R-project.org/package=rAltmetric, r package version 0.3.
18. Lammey R. Using the Crossref Metadata API to explore publisher content. Sci Ed 2016;3(3):109–11.
19. Levchenko M, Gou Y, Graef F, Hamelers A, Huang Z, Ide-Smith M, et al. Europe PMC in 2017. Nucleic acids research 2018;46(D1):D1254–D1260.
20. JA S, Interactive timeline Wikipedia COVID-19. Github; 2021.
21. JA S, Interactive network Wikipedia COVID-19. Github; 2021.
22. JA S, Zenodo repository Wikipedia COVID-19. Zenodo; 2021.
23. JA BOARS, Supporting data for "Citation needed? Wikipedia bibliometrics during the first wave of the COVID pandemic". GigaScience Database; 2021.
24. Wikipedia, Wikipedia:Core content policies; 2020. https://en.wikipedia.org/wiki/Wikipedia:Core_content_policies.
25. Williamson BN, Feldmann F, Schwarz B, Meade-White K, Porter DP, Schulz J, et al. Clinical benefit of remdesivir in rhesus macaques infected with SARS-CoV-2. BioRxiv 2020;.
26. Ioannidis JP, Axfors C, Contopoulos-Ioannidis DG. Population-level COVID-19 mortality risk for non-elderly individuals overall and for non-elderly individuals without underlying diseases in pandemic epicenters. medRxiv 2020;.
27. Fuertes FD, Caballero MI, Monzón S, Jiménez P, Varona S, Cuesta I, et al. Phylodynamics of SARS-CoV-2 transmission in Spain. bioRxiv 2020;.
28. Gonzalez-Reiche AS, Hernandez MM, Sullivan MJ, Ciferri B, Alshammary H, Obla A, et al. Introductions and early spread of SARS-CoV-2 in the New York City area. Science 2020;.
29. Ming W, Huang J, Zhang C. Breaking down of the healthcare system: Mathematical modelling for controlling the novel coronavirus (2019-nCoV) outbreak in wuhan, chinadoi: 10.1101/2020.01. 27.922443. URL https://doi org/101101% 2F2020 2020;1:922443.
30. Silverman JD, Hupert N, Washburne AD. Using ILI surveillance to estimate state-specific case detection rates and forecast SARS-CoV-2 spread in the United States. medRxiv 2020;.
31. Kendall M, Parker M, Fraser C, Nurtay A, Wymant C, Bonsall D, et al. Quantifying SARS-CoV-2 transmission suggests epidemic control with digital contact tracing. Science 2020;.
32. Topley W, Wilson G. The spread of bacterial infection. The problem of herd-immunity. Epidemiology & Infection 1923;21(3):243–249.
33. Wikipedia, Wikipedia:Identifying reliable sources (medicine); 2021. https://en.wikipedia.org/wiki/Wikipedia:Identifying_reliable_sources_(medicine).
34. Zhou P, Yang XL, Wang XG, Hu B, Zhang L, Zhang W, et al. Discovery of a novel coronavirus associated with the recent pneumonia outbreak in humans and its potential bat origin. BioRxiv 2020;.
35. Keegan B, Gergle D, Contractor N. Hot off the wiki: Structures and dynamics of Wikipedia's coverage of breaking news events. American Behavioral Scientist 2013;57(5):595–622.
36. Kagan D, Moran-Gilad J, Fire M. Scientometric trends for coronaviruses and other emerging viral infections. GigaScience 2020;9(8):giaa085.
37. Generous N, Fairchild G, Deshpande A, Del Valle SY, Priedhorsky R. Global disease monitoring and forecasting with Wikipedia. PLoS Comput Biol 2014;10(11):e1003892.
38. Wu Q, Irani D, Pu C, Ramaswamy L. Elusive Vandalism Detection in Wikipedia: A Text Stability-Based Approach. In: Proceedings of the 19th ACM International Conference on Information and Knowledge Management CIKM '10, New York, NY, USA: Association for Computing Machinery; 2010. p. 1797–1800. https://doi.org/10.1145/1871437.1871732.
39. Maggio LA, Steinberg RM, Piccardi T, Willinsky JM. Meta-Research: Reader engagement with medical content on Wikipedia. Elife 2020;9:e52426.
40. Yasseri T, Sumi R, Rung A, Kornai A, Kertész J. Dynamics of conflicts in Wikipedia. PloS one 2012;7(6):e38869.
41. Joorabchi A, Doherty C, Dawson J. 'WP2Cochrane', a tool linking Wikipedia to the Cochrane Library: Results of a bibliometric analysis evaluating article quality and importance. Health Informatics Journal 2020;26(3):1881–1897.
42. Wikipedia, Wikipedia Project COVID-19: Reference sources; 2021. https://en.wikipedia.org/wiki/Wikipedia:WikiProject_COVID-19/Reference_sources.
43. Fu DY, Hughey JJ. Meta-Research: Releasing a preprint is associated with more attention and citations for the peer-reviewed article. Elife 2019;8:e52646.
44. Fraser N, Brierley L, Dey G, Polka JK, Pálfy M, Coates JA. Preprinting a pandemic: the role of preprints in the COVID-19 pandemic. bioRxiv 2020;.
45. Pooladian A, Borrego Á. Methodological issues in measuring citations in Wikipedia: a case study in Library and Information Science. Scientometrics 2017;113(1):455–464.
46. Verma AA, Dubey N, Iyengar SRS, Setia S. In: Tracing the Factoids: The Anatomy of Information Re-Organization in Wikipedia Articles New York, NY, USA: Association for Computing Machinery; 2021. p. 572–579. https://doi.org/10.1145/3442442.3452342.
47. Greshake Tzovaras B, Angrist M, Arvai K, Dulaney M, Estrada-Galiñanes V, Gunderson B, et al. Open Humans: A platform for participant-centered research and personal data exploration. GigaScience 2019;8(6):giz076.
48. Sobel J, Henry L, Rotman N, Rando G. BeerDeCoded: the open beer metagenome project. F1000Research 2017;6.
49. Conrad CC, Hilchey KG. A review of citizen science and community-based environmental monitoring: issues and opportunities. Environmental monitoring and assessment 2011;176(1):273–291.
50. McGowan ML, Choudhury S, Juengst ET, Lambrix M, Settersten RA, Fishman JR. "Let's pull these technologies out of the ivory tower": The politics, ethos, and ironies of participant-driven genomic research. BioSocieties 2017;12(4):494–519.
51. DiResta R. Institutional Authority Has Vanished. Wikipedia Points to the Answer. The Atlantic 2021;July(4):494–519.
52. Kwok R. Research impact: Altmetrics make their mark. Nature 2013;500(7463):491–493.
53. Meschede C, Siebenlist T. Cross-metric compatability and inconsistencies of altmetrics. Scientometrics 2018;115(1):283–297.
54. Paskin N. Digital object identifier (DOI®) system. Encyclopedia of library and information sciences 2010;3:1586–1592.

Supplementary information

Table 1. Preprints cited within the Wikipedia COVID-19 Corpus

Title	DOI	Author	Year
Isolation and Characterization of 2019-nCoV-like Coronavirus from Malayan Pangolins	10.1101/2020.02.17.951335	Xiao K, Zhai J, Feng Y, Zhou N, Zhang X, Zou J, Li N, Guo Y, Li X, Shen X, Zhang Z, Shu F, Huang W, Li Y, Zhang Z, Chen R, Wu Y, Peng S, Huang M, Xie W, Cai Q, Hou F, Liu Y, Chen W, Xiao L, Shen Y.	2020
Evidence of recombination in coronaviruses implicating pangolin origins of nCoV-2019	10.1101/2020.02.07.939207	Wong MC, Javornik Cregeen SJ, Ajami NJ, Petrosino JF.	2020
Spike mutation pipeline reveals the emergence of a more transmissible form of SARS-CoV-2	10.1101/2020.04.29.069054	Korber B, Fischer W, Gnanakaran S, Yoon H, Theiler J, Abfalterer W, Foley B, Giorgi E, Bhattacharya T, Parker M, Partridge D, Evans C, Freeman T, de Silva T, LaBranche C, Montefiori D, on behalf of the Sheffield COVID-19 Genomics Group.	2020
Global profiling of SARS-CoV-2 specific IgG/IgM responses of convalescents using a proteome microarray	10.1101/2020.03.20.20039495	Jiang H, Li Y, Zhang H, Wang W, Men D, Yang X, Qi H, Zhou J, Tao S.	2020
Novel coronavirus 2019-nCoV: early estimation of epidemiological parameters and epidemic predictions	10.1101/2020.01.23.20018549	Read JM, Bridgen JR, Cummings DA, Ho A, Jewell CP.	2020
Aerodynamic Characteristics and RNA Concentration of SARS-CoV-2 Aerosol in Wuhan Hospitals during COVID-19 Outbreak	10.1101/2020.03.08.982637	Liu Y, Ning Z, Chen Y, Guo M, Liu Y, Gali NK, Sun L, Duan Y, Cai J, Westerdahl D, Liu X, Ho K, Kan H, Fu Q, Lan K.	2020
Correlation Analysis Between Disease Severity and Inflammation-related Parameters in Patients with COVID-19 Pneumonia	10.1101/2020.02.25.20025643	Gong J, Dong H, Xia SQ, Huang YZ, Wang D, Zhao Y, Liu W, Tu S, Zhang M, Wang Q, Lu F.	2020
Estimation of COVID-2019 burden and potential for international dissemination of infection from Iran	10.1101/2020.02.24.20027375	Tuite AR, Bogoch I, Sherbo R, Watts A, Fisman DN, Khan K.	2020
Explaining national differences in the mortality of COVID-19: individual patient simulation model to investigate the effects of testing policy and other factors on apparent mortality.	10.1101/2020.04.02.20050633	Michaels JA, Stevenson MD.	2020
Saliva is more sensitive for SARS-CoV-2 detection in COVID-19 patients than nasopharyngeal swabs	10.1101/2020.04.16.20067835	Wyllie AL, Fournier J, Casanovas-Massana A, Campbell M, Tokuyama M, Vijayakumar P, Geng B, Muenker MC, Moore AJ, Vogels CBF, Petrone ME, Ott IM, Lu P, Lu-Culligan A, Klein J, Venkataraman A, Earnest R, Simonov M, Datta R, Handoko R, Naushad N, Sewanan LR, Valdez J, White EB, Lapidus S, Kalinich CC, Jiang X, Kim DJ, Kudo E, Linehan M, Mao T, Moriyama M, Oh JE, Park A, Silva J, Song E, Takahashi T, Taura M, Weizman O, Wong P, Yang Y, Bermejo S, Odio C, Omer SB, Dela Cruz CS, Farhadian S, Martinello RA, Iwasaki A, Grubaugh ND, Ko AI.	2020
Neutralizing antibody responses to SARS-CoV-2 in a COVID-19 recovered patient cohort and their implications	10.1101/2020.03.30.20047365	Wu F, Wang A, Liu M, Wang Q, Chen J, Xia S, Ling Y, Zhang Y, Xun J, Lu L, Jiang S, Lu H, Wen Y, Huang J.	2020
Estimation of SARS-CoV-2 Infection Prevalence in Santa Clara County	10.1101/2020.03.24.20043067	Yadlowsky S, Shah N, Steinhardt J.	2020
Population-level COVID-19 mortality risk for non-elderly individuals overall and for non-elderly individuals without underlying diseases in pandemic epicenters	10.1101/2020.04.05.20054361	Ioannidis JPA, Axfors C, Contopoulos-Ioannidis DG.	2020
Respiratory disease and virus shedding in rhesus macaques inoculated with SARS-CoV-2	10.1101/2020.03.21.001628	Munster VJ, Feldmann F, Williamson BN, van Doremalen N, Pérez-Pérez L, Schulz J, Meade-White K, Okumura A, Callison J, Brumbaugh B, Avanzato VA, Rosenke R, Hanley PW, Saturday G, Scott D, Fischer ER, de Wit E.	2020
Clinical benefit of remdesivir in rhesus macaques infected with SARS-CoV-2	10.1101/2020.04.15.043166	Williamson BN, Feldmann F, Schwarz B, Meade-White K, Porter DP, Schulz J, Doremalen Nv, Leighton I, Yinda CK, Pérez-Pérez L, Okumura A, Lovaglio J, Hanley PW, Saturday G, Bosio CM, Anzick S, Barbian K, Cihlar T, Martens C, Scott DP, Munster VJ, Wit Ed.	2020
Discovery of a novel coronavirus associated with the recent pneumonia outbreak in humans and its potential bat origin	10.1101/2020.01.22.914952	Zhou P, Yang X, Wang X, Hu B, Zhang L, Zhang W, Si H, Zhu Y, Li B, Huang C, Chen H, Chen J, Luo Y, Guo H, Jiang R, Liu M, Chen Y, Shen X, Wang X, Zheng X, Zhao K, Chen Q, Deng F, Liu L, Yan B, Zhan F, Wang Y, Xiao G, Shi Z.	2020

Breaking down of the healthcare system: Mathematical modelling for controlling the novel coronavirus (2019-nCoV) outbreak in Wuhan, China	10.1101/2020.01.27.922443	Ming W, Huang J, Zhang CJP.	2020
Introductions and early spread of SARS-CoV-2 in the New York City area	10.1101/2020.04.08.20056929	Gonzalez-Reiche AS, Hernandez MM, Sullivan M, Ciferri B, Alshammary H, Obla A, Fabre S, Kleiner G, Polanco J, Khan Z, Alburquerque B, van de Guchte A, Dutta J, Francoeur N, Melo BS, Oussenko I, Deikus G, Soto J, Sridhar SH, Wang Y, Twyman K, Kasarskis A, Altman DR, Smith M, Sebra R, Aberg J, Krammer F, Garcia-Sarstre A, Luksza M, Patel G, Paniz-Mondolfi A, Gitman M, Sordillo EM, Simon V, van Bakel H.	2020
Phylodynamics of SARS-CoV-2 transmission in Spain	10.1101/2020.04.20.050039	Díez-Fuertes F, Iglesias-Caballero M, Monzón S, Jiménez P, Varona S, Cuesta I, Zaballos Á, Thomson MM, Jiménez M, García Pérez J, Pozo F, Pérez-Olmeda M, Alcamí J, Casas I.	2020
Using ILI surveillance to estimate state-specific case detection rates and forecast SARS-CoV-2 spread in the United States	10.1101/2020.04.01.20050542	Silverman JD, Hupert N, Washburne AD.	2020
Quantifying SARS-CoV-2 transmission suggests epidemic control with digital contact tracing	10.1101/2020.03.08.20032946	Ferretti L, Wymant C, Kendall M, Zhao L, Nurtay A, Abeler-Dorner L, Parker M, Bonsall DG, Fraser C.	2020
Adoption and impact of non-pharmaceutical interventions for COVID-19	10.12688/wellcomeopenres.15808.1	Imai N, Gaythorpe KA, Abbott S, Bhatia S, van Elsland S, Prem K, Liu Y, Ferguson NM.	2020
Aberrant pathogenic GM-CSF+ T cells and inflammatory CD14+CD16+ monocytes in severe pulmonary syndrome patients of a new coronavirus	10.1101/2020.02.12.945576	Zhou Y, Fu B, Zheng X, Wang D, Zhao C, qi Y, Sun R, Tian Z, Xu X, Wei H.	2020
SARS-CoV-2 invades host cells via a novel route: CD147-spike protein	10.1101/2020.03.14.988345	Wang K, Chen W, Zhou Y, Lian J, Zhang Z, Du P, Gong L, Zhang Y, Cui H, Geng J, Wang B, Sun X, Wang C, Yang X, Lin P, Deng Y, Wei D, Yang X, Zhu Y, Zhang K, Zheng Z, Miao J, Guo T, Shi Y, Zhang J, Fu L, Wang Q, Bian H, Zhu P, Chen Z.	2020
Functional assessment of cell entry and receptor usage for lineage B ß-coronaviruses, including 2019-nCoV	10.1101/2020.01.22.915660	Letko M, Munster V.	2020
Broad anti-coronaviral activity of FDA approved drugs against SARS-CoV-2 in vitro and SARS-CoV in vivo	10.1101/2020.03.25.008482	Weston S, Coleman CM, Haupt R, Logue J, Matthews K, Frieman MB.	2020
Global and Temporal Patterns of Submicroscopic Plasmodium falciparum Malaria Infection	10.1101/554311	Whittaker C, Slater H, Bousema T, Drakeley C, Ghani A, Okell L.	2019

Table 2. Most cited scientific papers in the scientific literature within COVID-19 Wikipedia corpus

Title	Year	Journal	Authors	Citation Count
Understanding the Warburg effect: the metabolic requirements of cell proliferation.	2009	Science	Vander Heiden MG, Cantley LC, Thompson CB.	4927
The MIQE guidelines: minimum information for publication of quantitative real-time PCR experiments.	2009	Clin Chem	Bustin SA, Benes V, Garson JA, Hellemans J, Huggett J, Kubista M, Mueller R, Nolan T, Pfaffl MW, Shipley GL, Vandesompele J, Wittwer CT.	4809
Isolation of a cDNA clone derived from a blood-borne non-A, non-B viral hepatitis genome.	1989	Science	Choo QL, Kuo G, Weiner AJ, Overby LR, Bradley DW, Houghton M.	3672
Isolation of a T-lymphotropic retrovirus from a patient at risk for acquired immune deficiency syndrome (AIDS).	1983	Science	Barré-Sinoussi F, Chermann JC, Rey F, Nugeyre MT, Chamaret S, Gruest J, Dauguet C, Axler-Blin C, Vézinet-Brun F, Rouzioux C, Rozenbaum W, Montagnier L.	3016
The American-European Consensus Conference on ARDS. Definitions, mechanisms, relevant outcomes, and clinical trial coordination.	1994	Am J Respir Crit Care Med	Bernard GR, Artigas A, Brigham KL, Carlet J, Falke K, Hudson L, Lamy M, Legall JR, Morris A, Spragg R.	2904
Toll-like receptors.	2003	Annu Rev Immunol	Takeda K, Kaisho T, Akira S.	2872
The acute respiratory distress syndrome.	2000	N Engl J Med	Ware LB, Matthay MA.	2720
Network biology: understanding the cell's functional organization.	2004	Nat Rev Genet	Barabási AL, Oltvai ZN.	2697
Surviving sepsis campaign: international guidelines for management of severe sepsis and septic shock: 2012.	2013	Crit Care Med	Dellinger RP, Levy MM, Rhodes A, Annane D, Gerlach H, Opal SM, Sevransky JE, Sprung CL, Douglas IS, Jaeschke R, Osborn TM, Nunnally ME, Townsend SR, Reinhart K, Kleinpell RM, Angus DC, Deutschman CS, Machado FR, Rubenfeld GD, Webb SA, Beale RJ, Vincent JL, Moreno R, Surviving Sepsis Campaign Guidelines Committee including the Pediatric Subgroup.	2461
A comprehensive analysis of protein-protein interactions in Saccharomyces cerevisiae.	2000	Nature	Uetz P, Giot L, Cagney G, Mansfield TA, Judson RS, Knight JR, Lockshon D, Narayan V, Srinivasan M, Pochart P, Qureshi-Emili A, Li Y, Godwin B, Conover D, Kalbfleisch T, Vijayadamodar G, Yang M, Johnston M, Fields S, Rothberg JM.	2416

Table 3. Most cited scientific papers in COVID-19 Wikipedia corpus

DOI	Authors	OA	Journal	Year	Source	Title	Wiki	Sci.lit
10.1038/s41586-020-2012-7	Zhou P, Yang XL, Wang XG, Hu B, Zhang L, Zhang W, Si HR, Zhu Y, Li B, Huang CL, Chen HD, Chen J, Luo Y, Guo H, Jiang RD, Liu MQ, Chen Y, Shen XR, Wang X, Zheng XS, Zhao K, Chen QJ, Deng F, Liu LL, Yan B, Zhan FX, Wang YY, Xiao GF, Shi ZL.	Y	Nature	2020	MED	A pneumonia outbreak associated with a new coronavirus of probable bat origin.	8	940
10.3390/v11020174	Wong ACP, Li X, Lau SKP, Woo PCY.	Y	Viruses	2019	MED	Global Epidemiology of Bat Coronaviruses.	6	28
10.1016/j.ijid.2020.01.009	Hui DS, I Azhar E, Madani TA, Ntoumi F, Kock R, Dar O, Ippolito G, Mchugh TD, Memish ZA, Drosten C, Zumla A, Petersen E.	Y	Int J Infect Dis	2020	MED	The continuing 2019-nCoV epidemic threat of novel coronaviruses to global health - The latest 2019 novel coronavirus outbreak in Wuhan, China.	5	228
10.1016/j.jmii.2020.03.013	Lau H, Khosrawipour V, Kocbach P, Mikolajczyk A, Ichii H, Schubert J, Bania J, Khosrawipour T.	Y	J Microbiol Immunol Infect	2020	MED	Internationally lost COVID-19 cases.	5	5
10.1038/d41586-020-00548-w	Cyranoski D.	N	Nature	2020	MED	Mystery deepens over animal source of coronavirus.	5	8
10.1038/s41591-020-0820-9	Andersen KG, Rambaut A, Lipkin WI, Holmes EC, Garry RF.	Y	Nat Med	2020	MED	The proximal origin of SARS-CoV-2.	5	147
10.3390/v2081803	Woo PC, Huang Y, Lau SK, Yuen KY.	Y	Viruses	2010	MED	Coronavirus genomics and bioinformatics analysis.	5	109
10.1007/978-1-4939-2438-7_1	Fehr AR, Perlman S.	Y	Methods Mol Biol	2015	MED	Coronaviruses: an overview of their replication and pathogenesis.	4	195
10.1007/s00134-020-05991-x	Ruan Q, Yang K, Wang W, Jiang L, Song J.	Y	Intensive Care Med	2020	MED	Clinical predictors of mortality due to COVID-19 based on an analysis of data of 150 patients from Wuhan, China.	4	66
10.1038/d41573-020-00016-0	Li G, De Clercq E.	N	Nat Rev Drug Discov	2020	MED	Therapeutic options for the 2019 novel coronavirus (2019-nCoV).	4	105
10.1038/s41422-020-0282-0	Wang M, Cao R, Zhang L, Yang X, Liu J, Xu M, Shi Z, Hu Z, Zhong W, Xiao G.	Y	Cell Res	2020	MED	Remdesivir and chloroquine effectively inhibit the recently emerged novel coronavirus (2019-nCoV) in vitro.	4	474
10.1093/cid/ciaa149	To KK, Tsang OT, Chik-Yan Yip C, Chan KH, Wu TC, Chan JMC, Leung WS, Chik TS, Choi CY, Kandamby DH, Lung DC, Tam AR, Poon RW, Fung AY, Hung IF, Cheng VC, Chan JF, Yuen KY.	Y	Clin Infect Dis	2020	MED	Consistent detection of 2019 novel coronavirus in saliva.	4	94
10.1093/ofid/ofaa105	McCreary EK, Pogue JM.	Y	Open Forum Infect Dis	2020	MED	Coronavirus Disease 2019 Treatment: A Review of Early and Emerging Options.	4	7
10.1126/science.aba9757	Chinazzi M, Davis JT, Ajelli M, Gioannini C, Litvinova M, Merler S, Pastore Y Piontti A, Mu K, Rossi L, Sun K, Viboud C, Xiong X, Yu H, Halloran ME, Longini IM, Vespignani A.	Y	Science	2020	MED	The effect of travel restrictions on the spread of the 2019 novel coronavirus (COVID-19) outbreak.	4	69
10.1093/jtm/taaa030	Rocklöv J, Sjödin H, Wilder-Smith A.	Y	J Travel Med	2020	MED	COVID-19 outbreak on the Diamond Princess cruise ship: estimating the epidemic potential and effectiveness of public health countermeasures.	3	25
10.1101/2020.02.07.939207	Wong MC, Javornik Cregeen SJ, Ajami NJ, Petrosino JF.	N	NA	2020	PPR	Evidence of recombination in coronaviruses implicating pangolin origins of nCoV-2019	3	15
10.1101/2020.02.17.951335	Xiao K, Zhai J, Feng Y, Zhou N, Zhang X, Zou J, Li N, Guo Y, Li X, Shen X, Zhang Z, Shu F, Huang W, Li Y, Zhang Z, Chen R, Wu Y, Peng S, Huang M, Xie W, Cai Q, Hou F, Liu Y, Chen W, Xiao L, Shen Y.	N	NA	2020	PPR	Isolation and Characterization of 2019-nCoV-like Coronavirus from Malayan Pangolins	3	24
10.1111/j.1600-0668.2007.00469.x	Xie X, Li Y, Chwang AT, Ho PL, Seto WH.	N	Indoor Air	2007	MED	How far droplets can move in indoor environments-revisiting the Wells evaporation-falling curve.	3	167
10.1111/tmi.13383	Velavan TP, Meyer CG.	Y	Trop Med Int Health	2020	MED	The COVID-19 epidemic.	3	70
10.1126/science.1118391	Li W, Shi Z, Yu M, Ren W, Smith C, Epstein JH, Wang H, Crameri G, Hu Z, Zhang H, Zhang J, McEachern J, Field H, Daszak P, Eaton BT, Zhang S, Wang LF.	N	Science	2005	MED	Bats are natural reservoirs of SARS-like coronaviruses.	3	967

SI datasets

(1) Table of scientific paper form europmc COVID-19 cited in wikipedia
(2) Table of Wikipedia article-DOI network
(3) Table of protected wikipedia COVID-19 articles

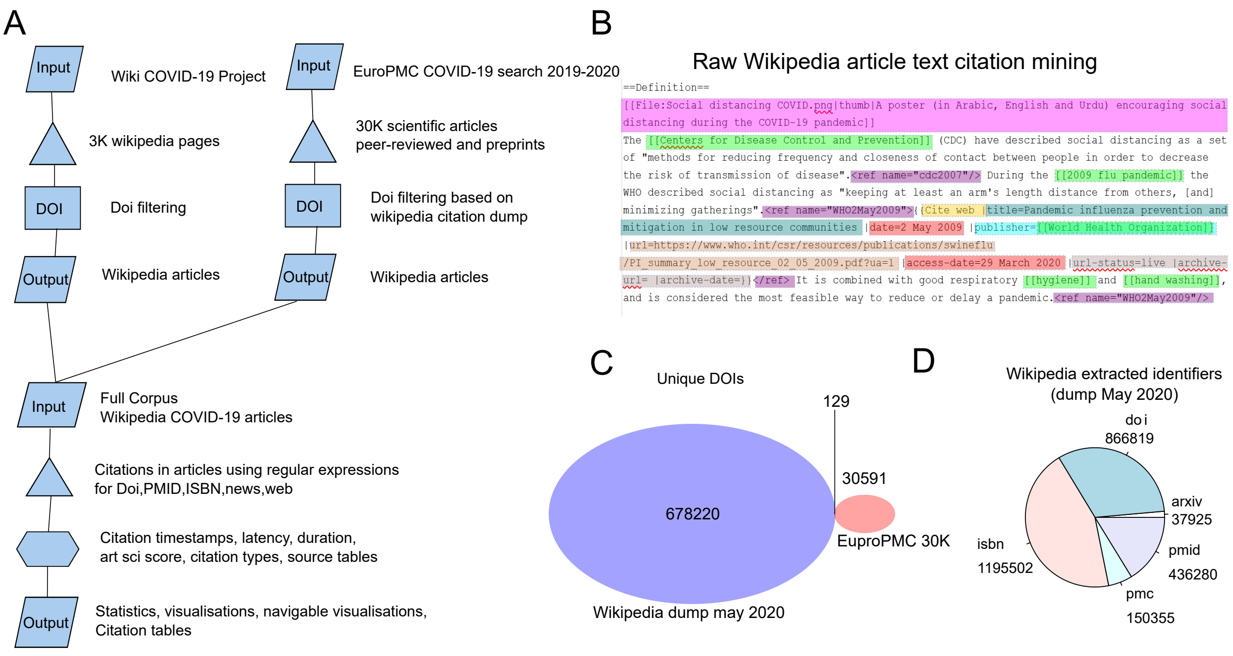

Figure S1. Corpus identification and citation extraction pipeline. A) Scheme of the corpus delimitation rational and citation extraction. To delimit our corpus of Wikipedia articles containing Digital Object Identifier (DOI), we applied two different strategies. First we scraped every Wikipedia pages from the COVID-19 Wikipedia project (about 3K pages) and we filtered them to keep only articles containing DOI citations (149 Wikipedia articles). For our second strategy, we searched the EuroPMC database for COVID-19, SARS-CoV2, SARS-nCoV19 - yielding 30,000 scientific papers, reviews and preprints. These were then compared to the citations extracted from the English Wikipedia dump of May 2020 (860,000 DOIs). Searching Wikipedia with the resulting set led us to identify an additional 91 Wikipedia articles containing at least one citation from the EuroPMC set. Taken together, from the resulting corpus of 231 Wikipedia articles, we extracted DOIs, PMIDs, ISBNs, websites and URLs using a set of regular expressions, as described in the methods. Subsequently, we computed several statistics for each Wikipedia article and we retrieved Atmetics, CrossRef and EuroPMC information for each of their cited papers' DOI. Finally, we produced tables of annotated citations and extracted information from each Wikipadia articles such as books, websites, newspapers. In addition, a timeline of Wikipedia articles and a network of Wikipedia articles linked by their shared scientific sources was produced. B) Example of raw Wikipedia text from the "Social distancing" article, highlighted with several parsed items from a reference. Pink: a hyperlink to an image file, green: Wikipedia hyperlinks, purple: reference, yellow: citation type, dark green: citation title, red: citation date, orange: citation URL. C) Overlap between DOIs from the Wikipedia dump and the 30K EuroPMC COVID-19-related scientific papers and preprints. D) Number of extracted citations with *mwcite* from the English Wikipedia dump of May 2020.

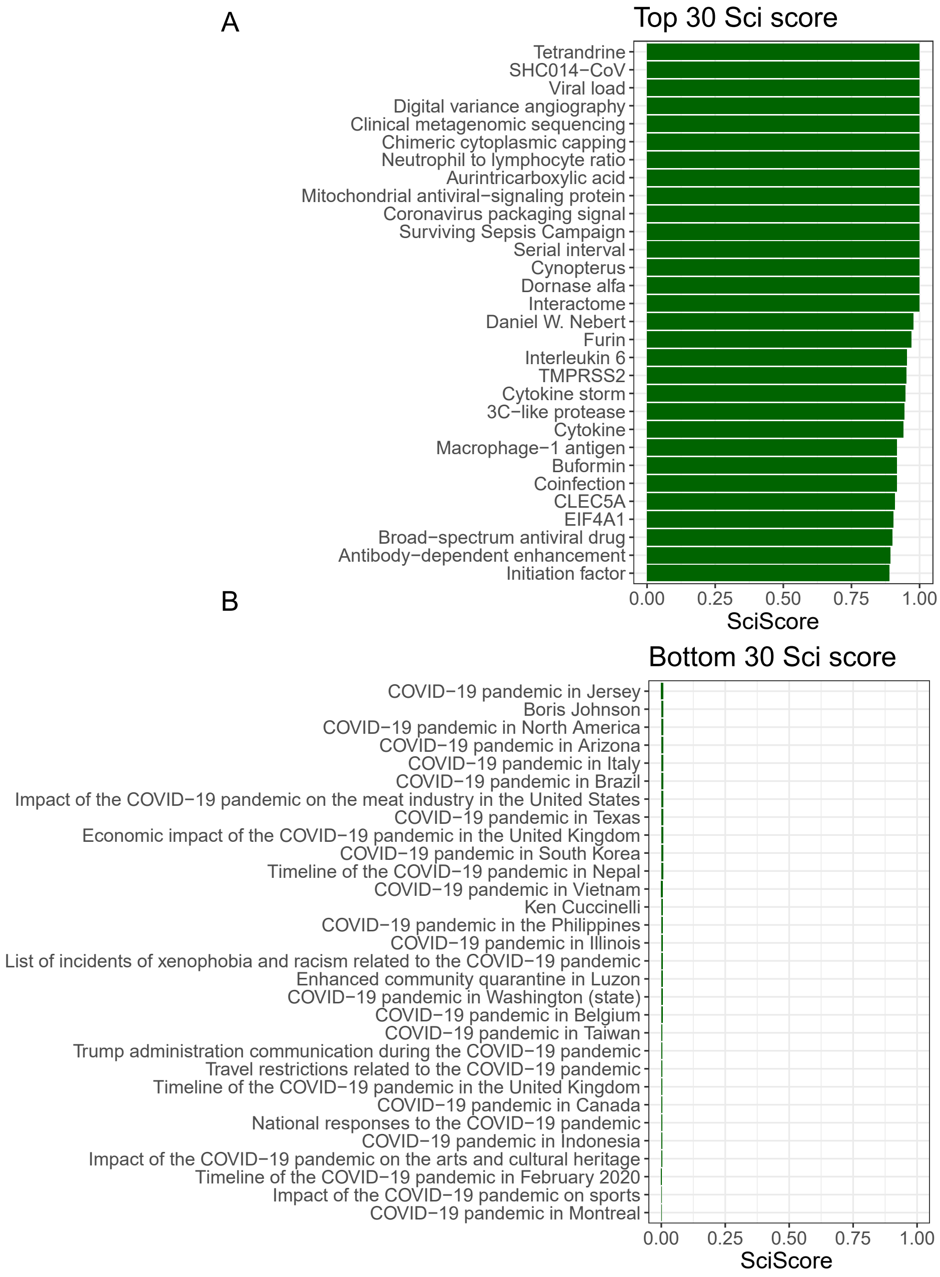

Figure S2. Articles from the Wikipedia COVID-19 corpus with A) the highest and B) lowest scientific scores. The scientific score was computed based on the reference content of each article, as defined in the methods section.

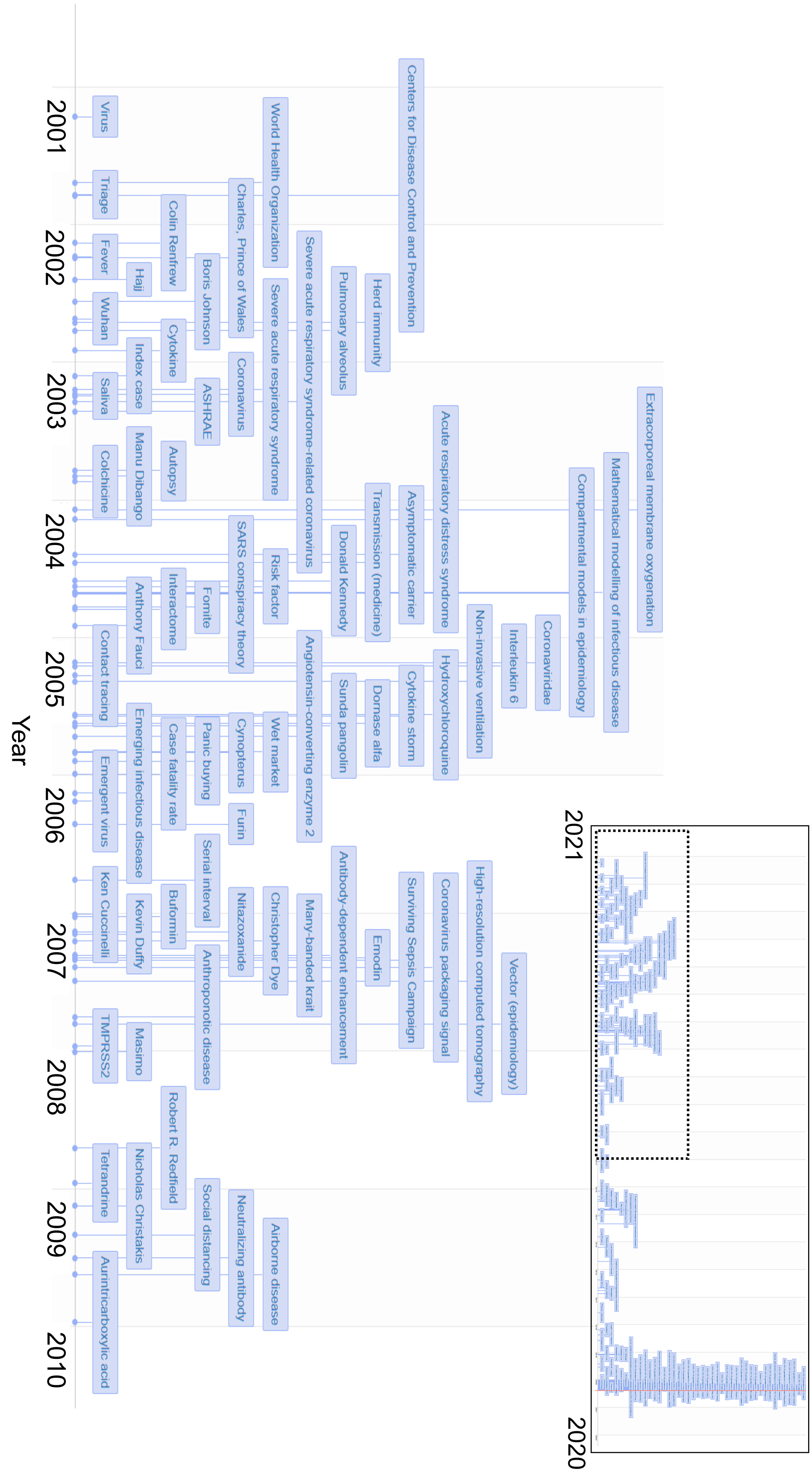

Figure S3. Timeline of the Wikipedia COVID-19 corpus articles, based on date of creation. See here for an interactive version of the timeline.

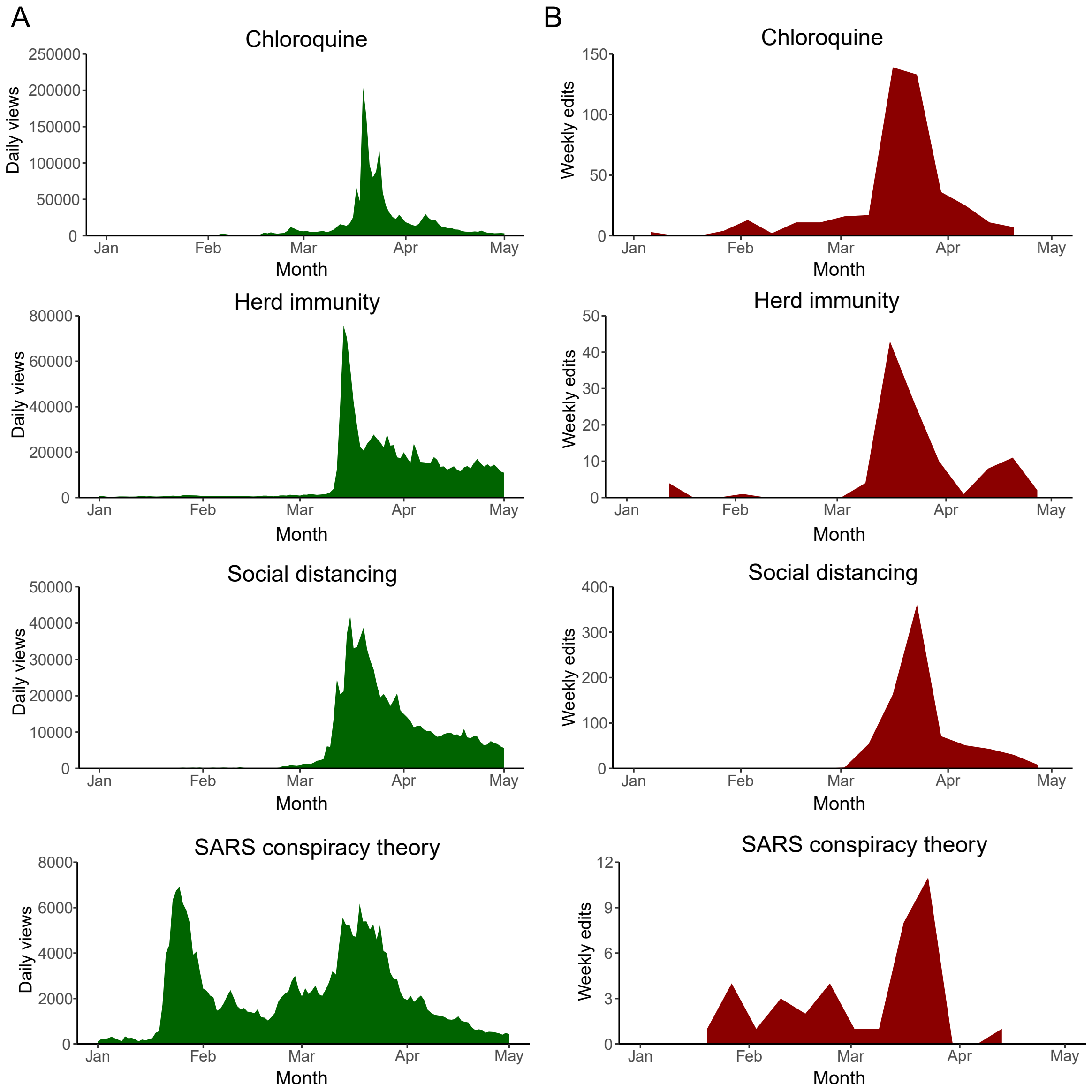

Figure S4. Selected articles' A) page views and B) edit counts during the first wave of COVID-19 pandemic (January-May of 2020).

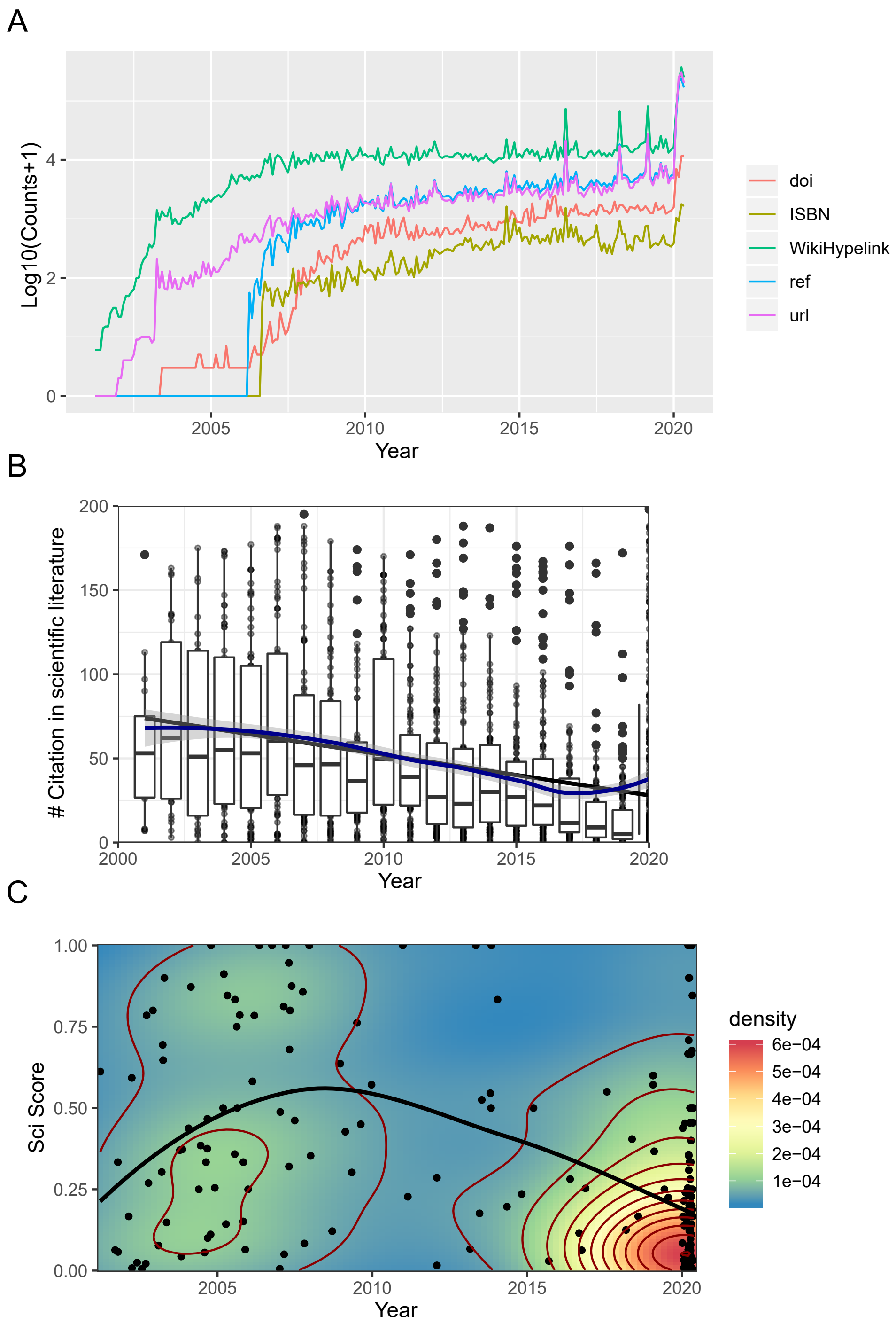

Figure S5. Historical characterization of citations in the COVID-19 corpus. A)Number of references on Wikipedia throughout time, parsed by different type of sources (doi, isbn, hyperlink, url). B) Number of citations in scientific literature as a function of the papers' publication year. C) Scientific score as a function of the creation date of Wikipedia article in the COVID-19 corpus.

Figure 1

Click here to access/download;Figure;Fig1.pdf

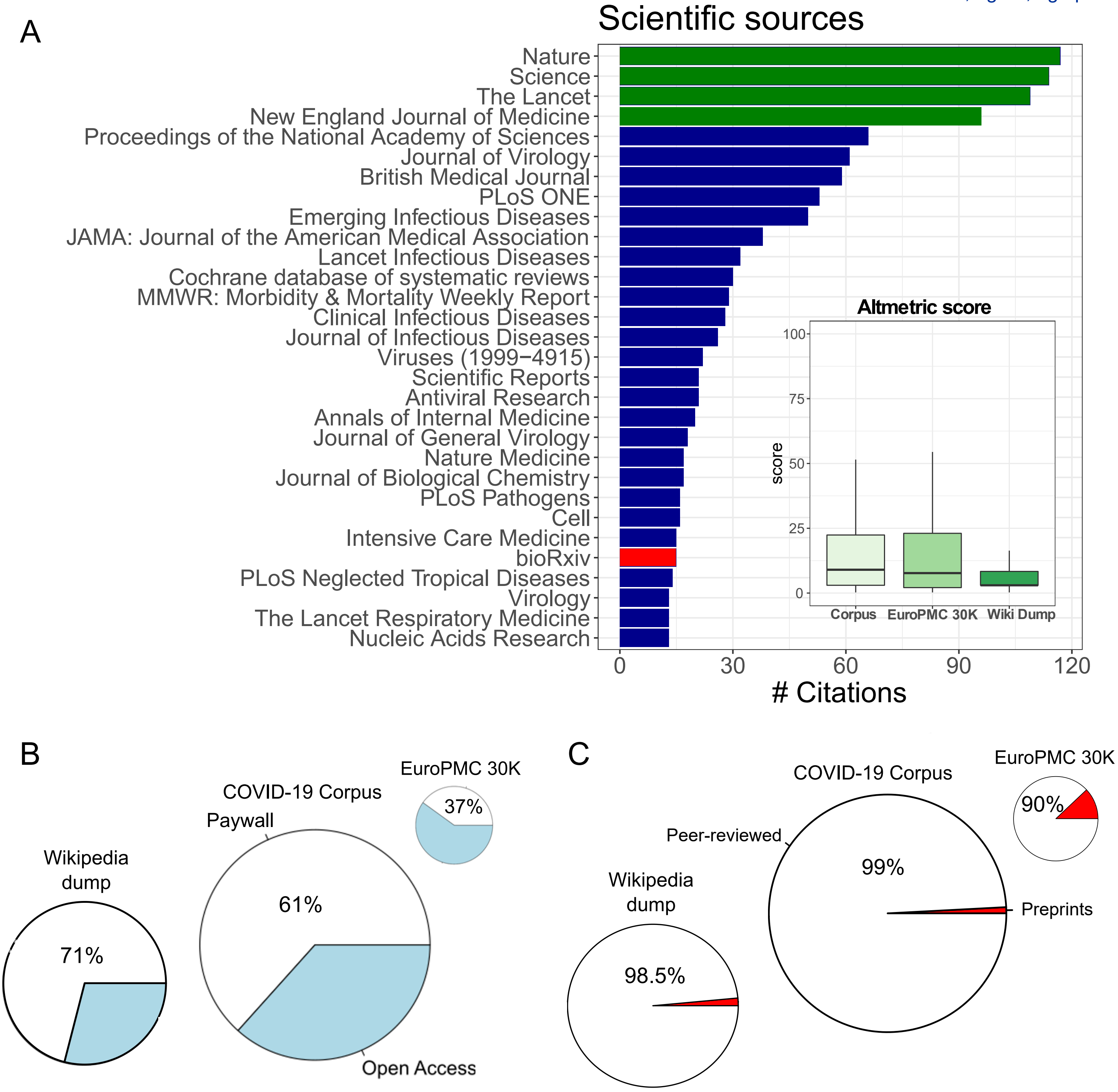

Figure 2

Click here to access/download;Figure;Fig2.pdf

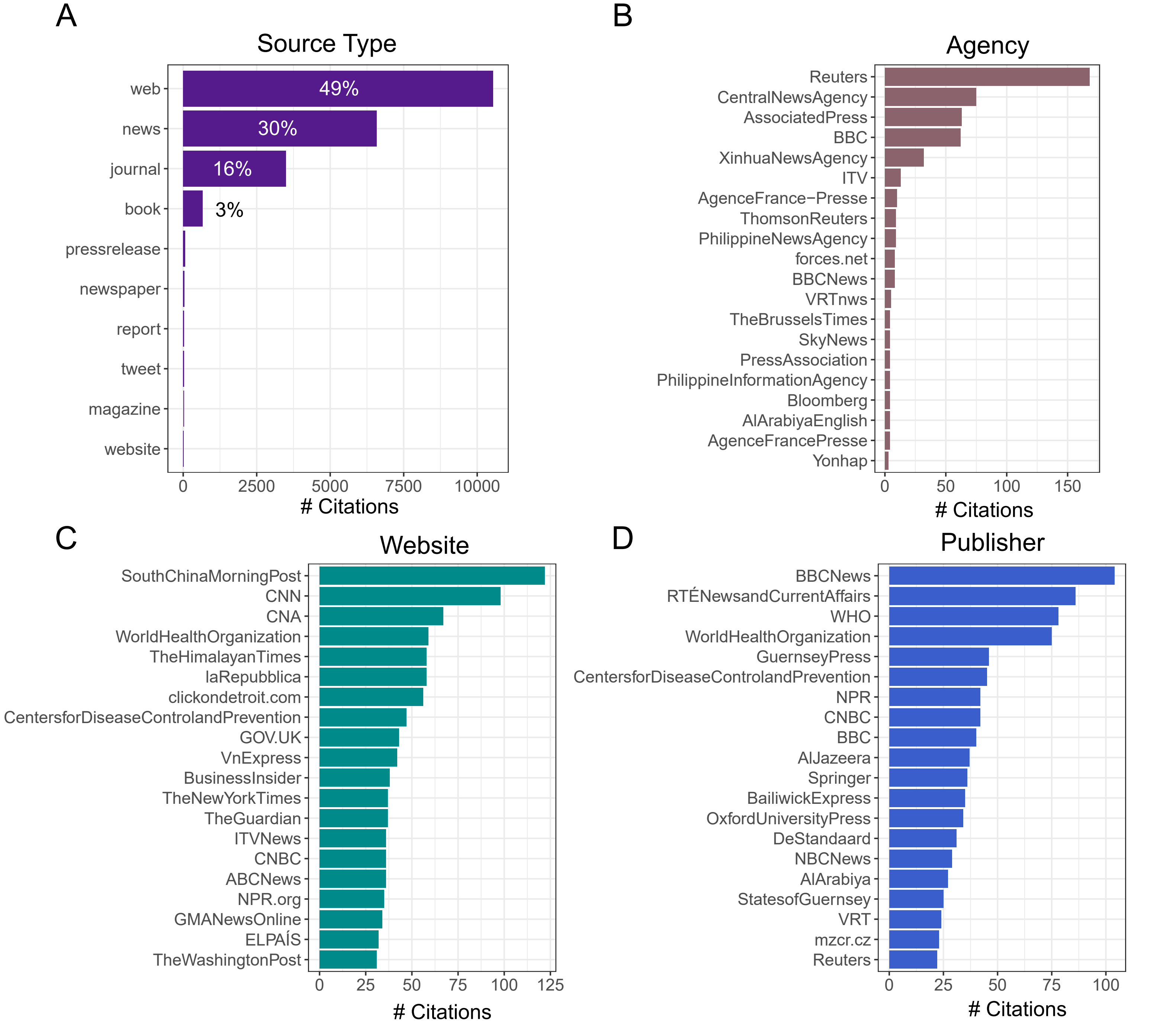

Figure 3

Click here to access/download;Figure;Fig3.pdf

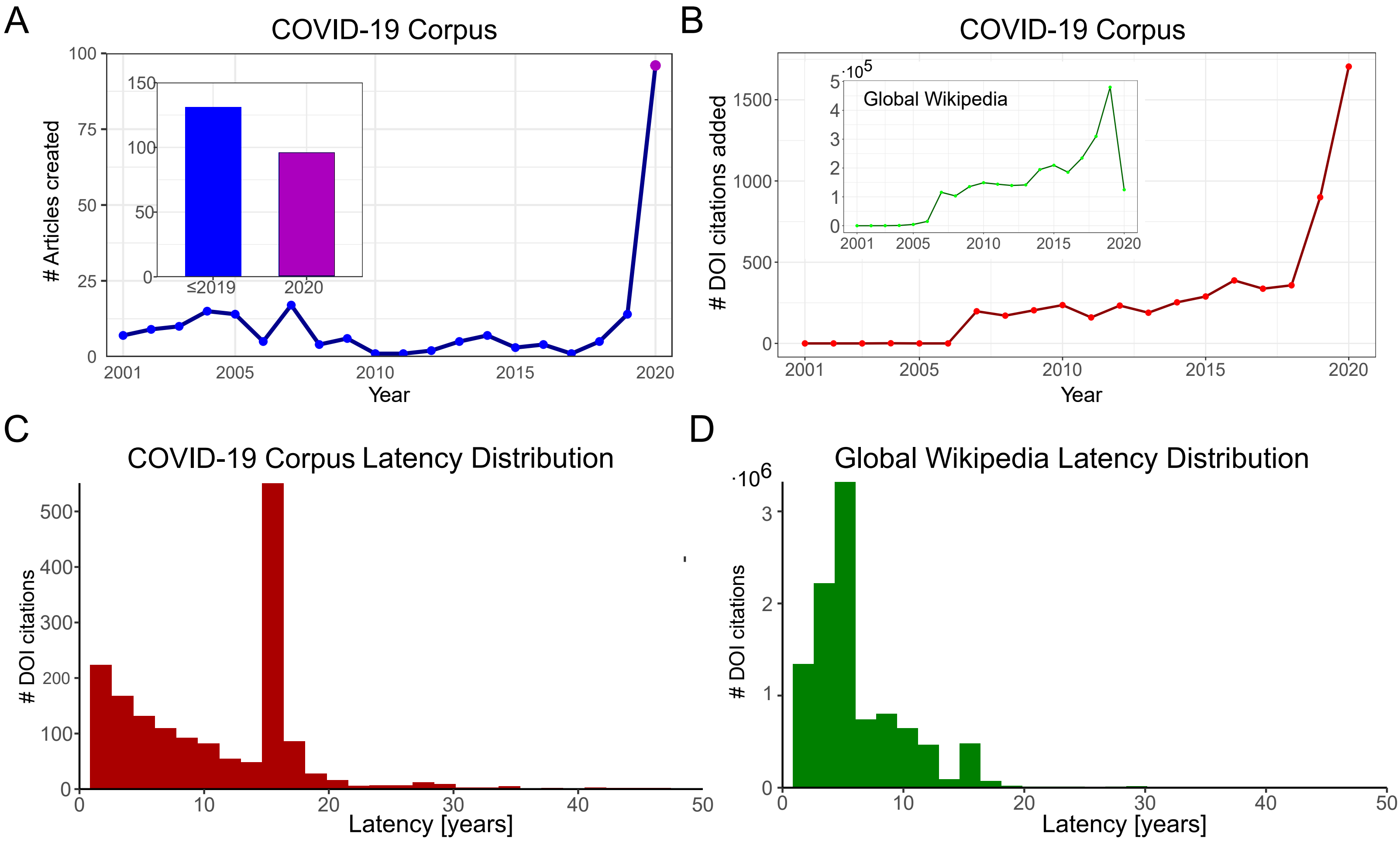

Figure 4

Click here to access/download;Figure;Fig4.pdf

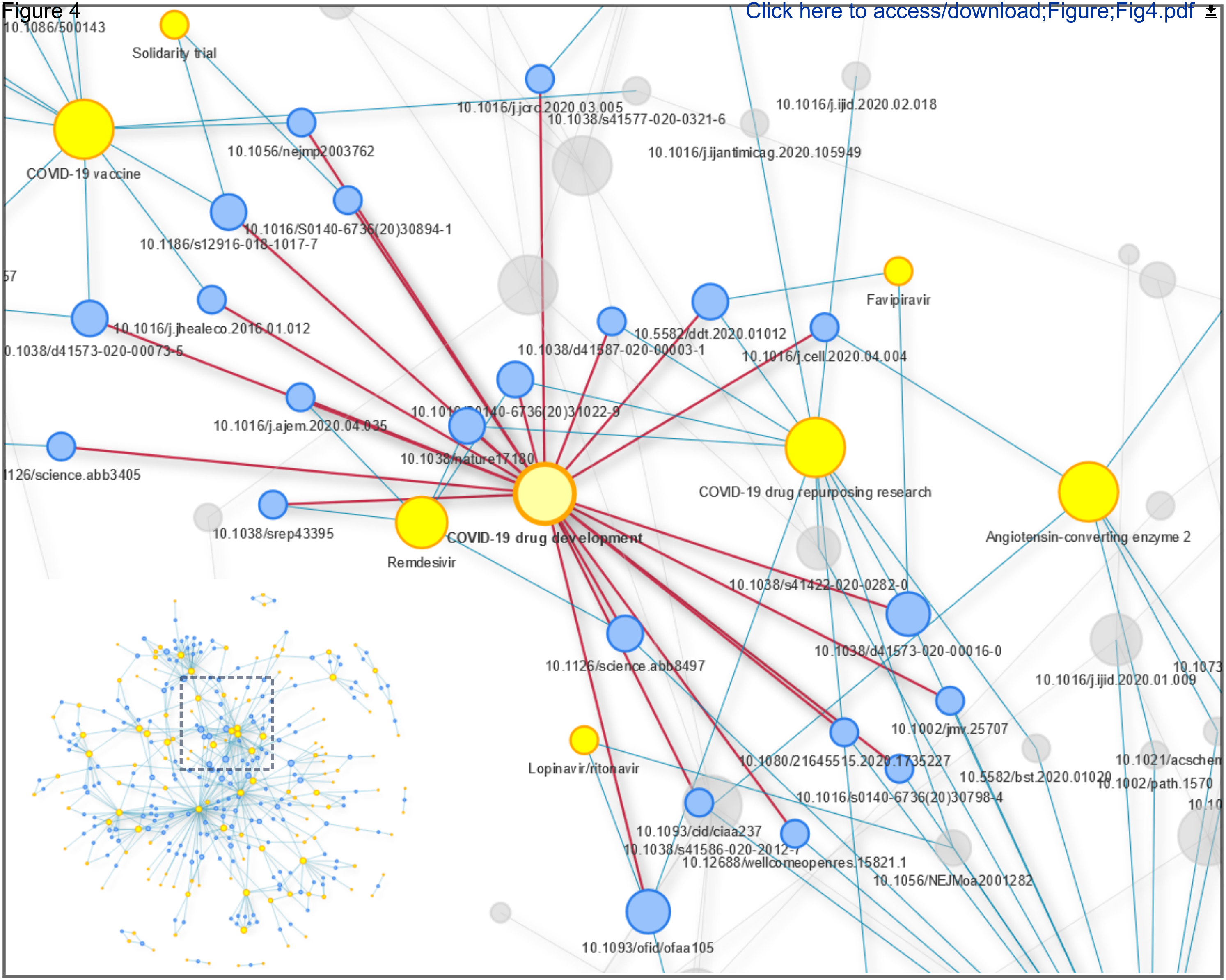

Click here to access/download;Figure;FigS1.pdf

Figure S1

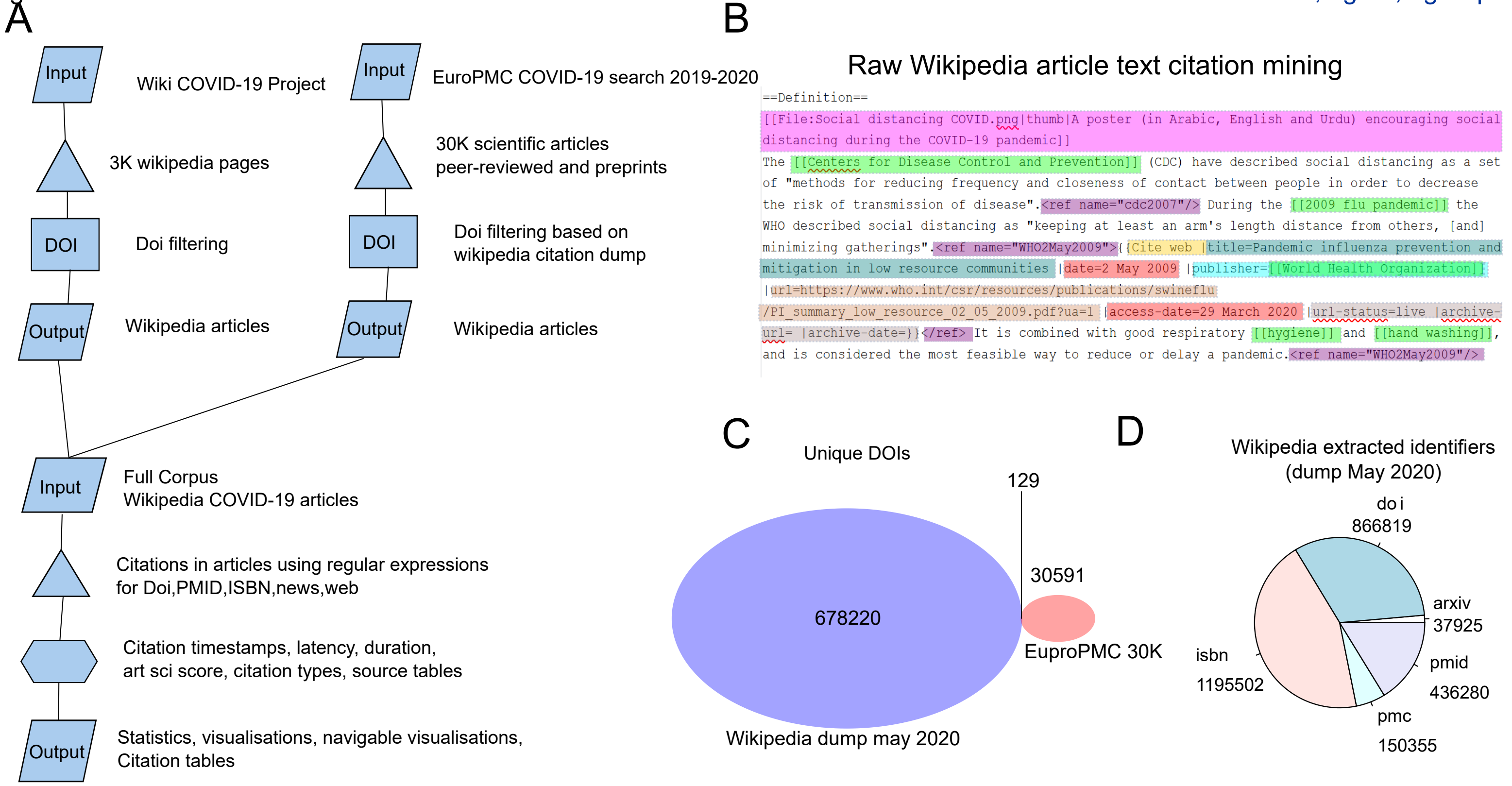

Figure S2

Click here to access/download;Figure;FigS2.pdf

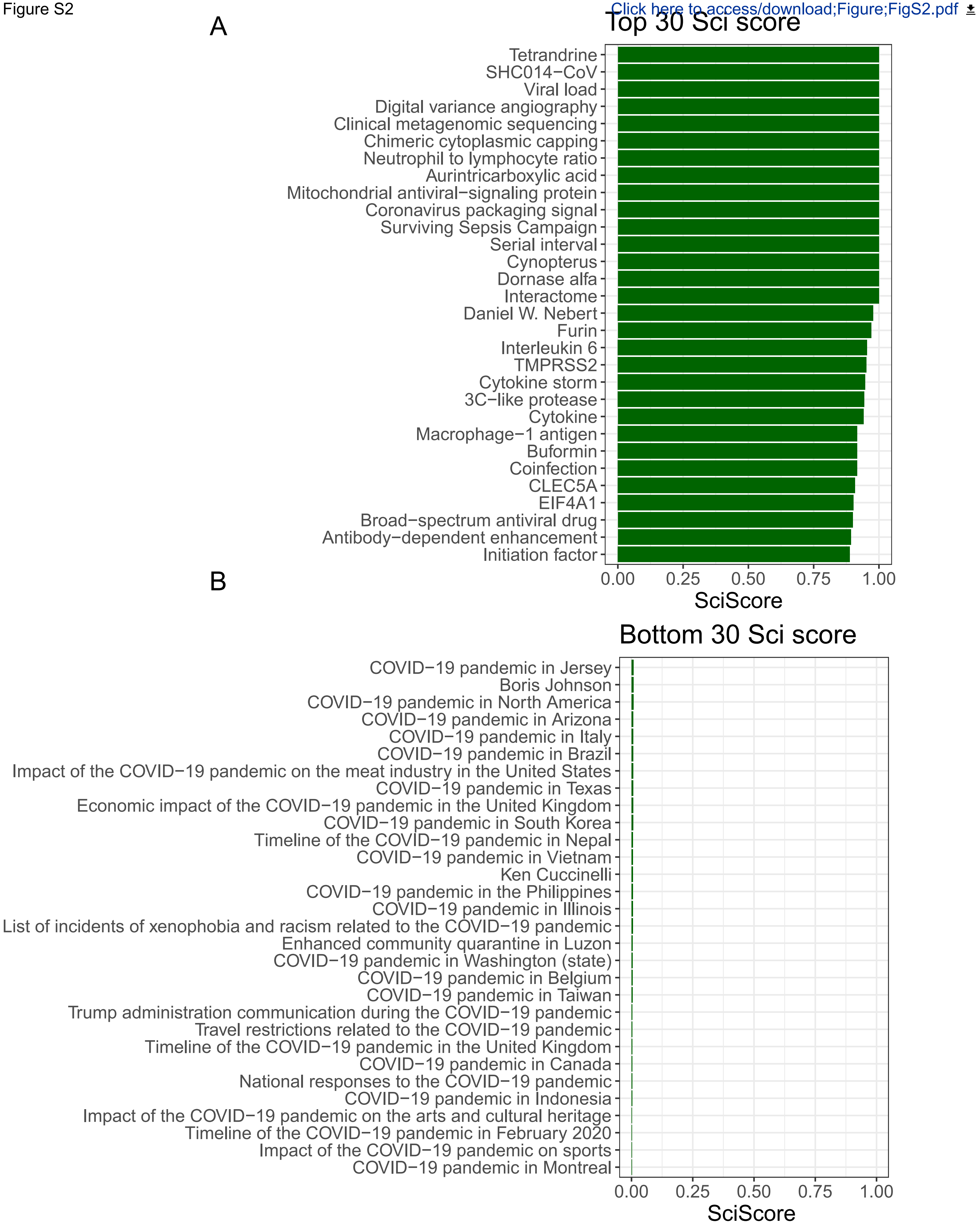

Figure S4

Click here to access/download;Figure;FigS4.pdf

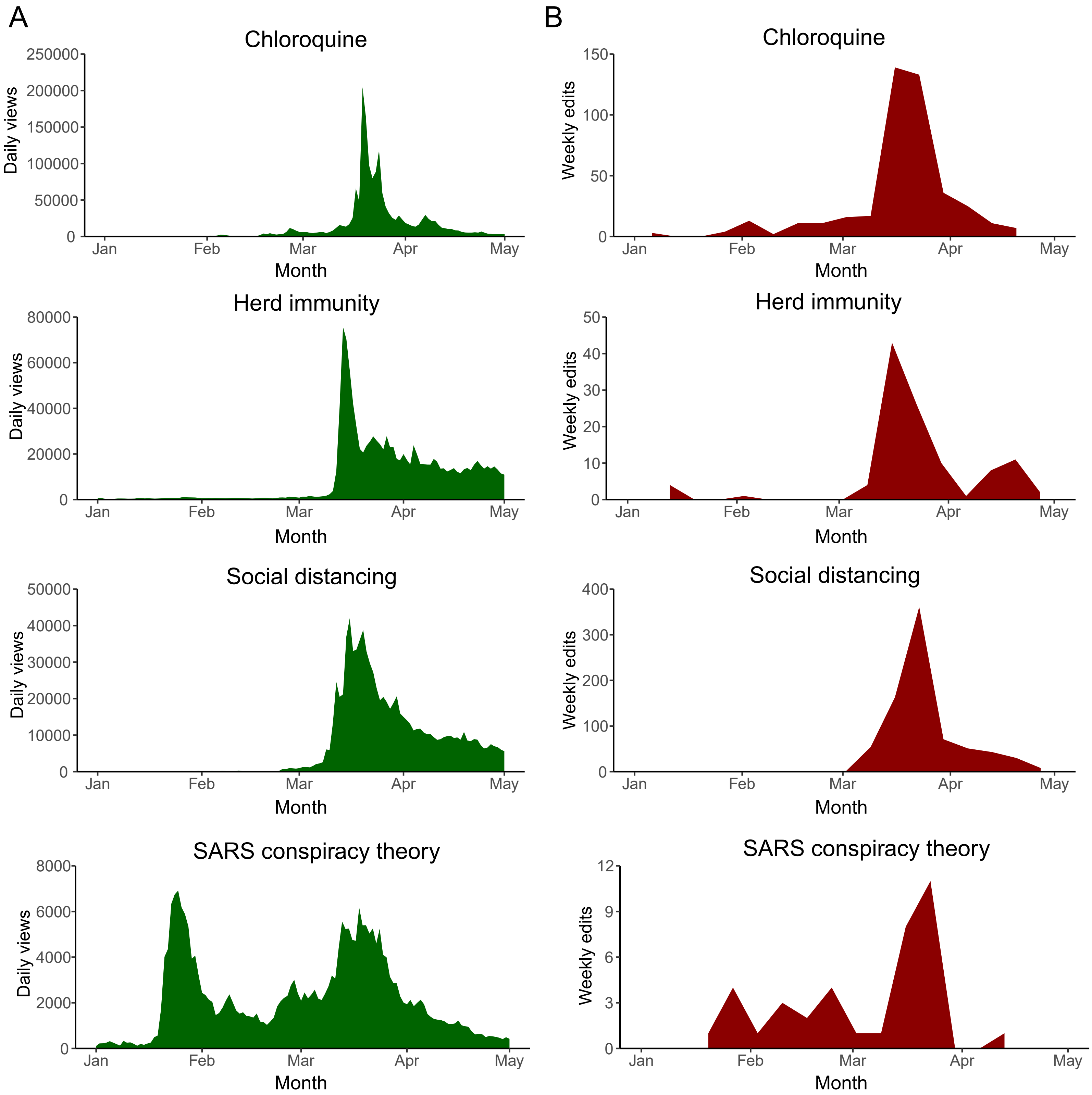

Figure S3

Click here to access/download;Figure;FigS3.pdf

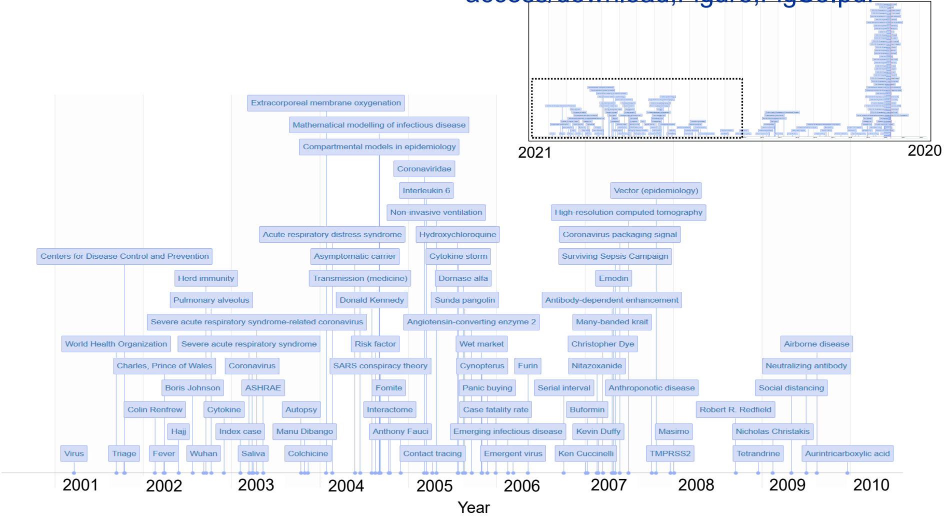

Manuscript
Click here to access/download;Figure;FigS5.pdf

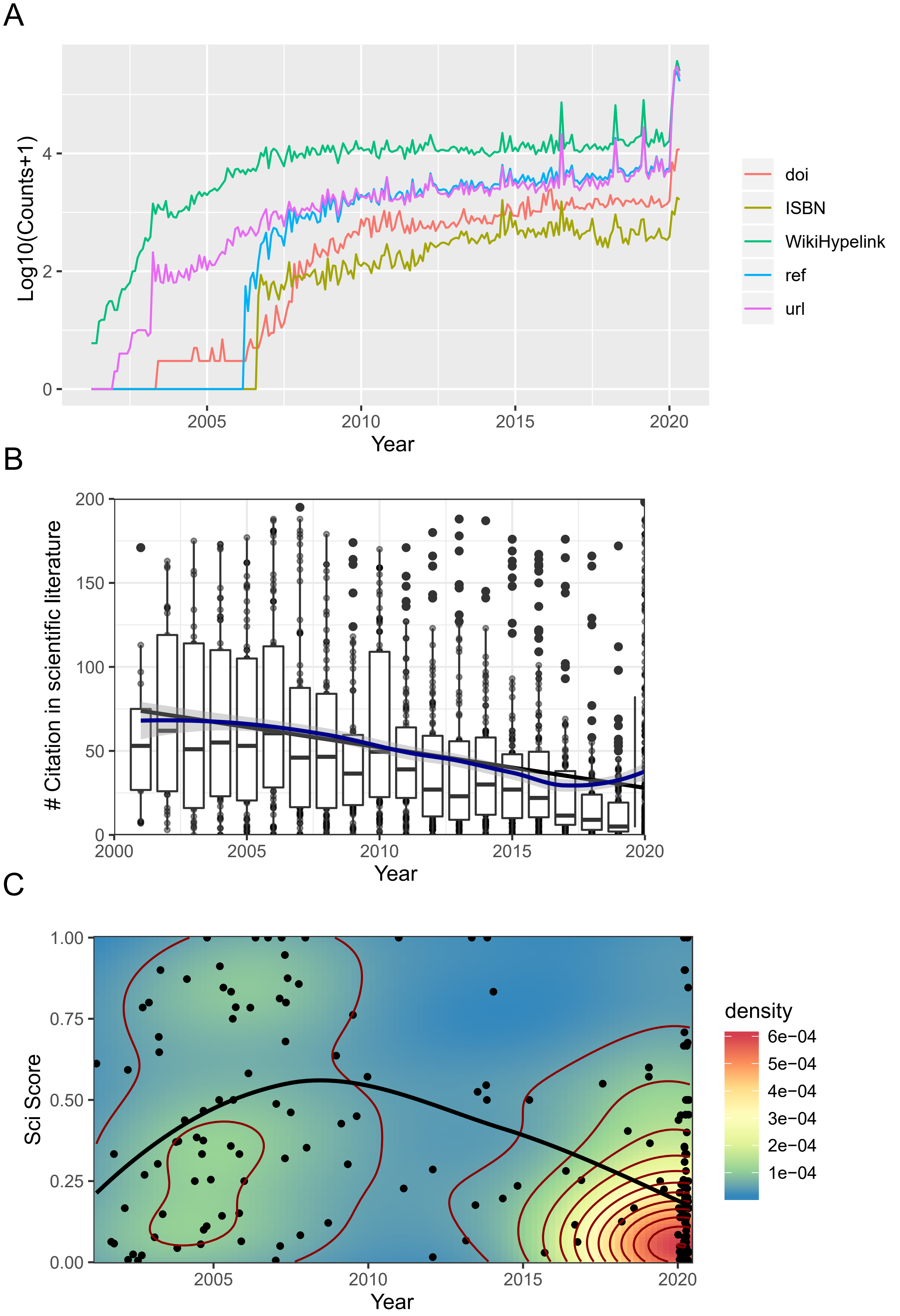

supp. data 2

Click here to access/download
Supplementary Material
Supp_data_Network.csv

supp. data 3

Click here to access/download
Supplementary Material
Supp_data_protected_corpus.csv

supp. data 1

Click here to access/download
Supplementary Material
supp_data_intesect_europmc_search_dump_doi_annotated.xlsx

Cover letter

Click here to access/download;Personal Cover;Cover_gigascience_071221.docx

Haifa, 07/12/2021

Dear editor,
With the present mail, we humbly resubmit for publication our manuscript, entitled: “Citation needed? Wikipedia bibliometrics during the first wave of the COVID pandemic.”

As required, we rearrange our data availability section to add the reference to the GigaDB repository and we made minor corrections to the main text. We arranged the order of some supplementary figure panels and tables to match better the flow of our main text.
Finally, per the suggestion of reviewer 3 we performed intense proofing of our main text.

Respectfully yours,
Omer Benjakob, Dr. Rona Aviram and Dr. Jonathan Sobel
