# Supplementary material for: Citation needed? Wikipedia bibliometrics during the first wave of the COVID-19 pandemic
Source: Gigascience. 2022 Jan 12;11:giab095. doi: 10.1093/gigascience/giab095 (PMC8756189; doi:10.1093/gigascience/giab095)
Supplement: giab095_Supplemental_Files [file giab095_supplemental_files.zip › supplementary_figures_and_tables.pdf]

Supplementary information

Table 1. Preprints cited within the Wikipedia COVID-19 Corpus

Title	DOI	Author	Year
Isolation and Characterization of 2019-nCoV-like Coronavirus from Malayan Pangolins	10.1101/2020.02.17.951335	Xiao K, Zhai J, Feng Y, Zhou N, Zhang X, Zou J, Li N, Guo Y, Li X, Shen X, Zhang Z, Shu F, Huang W, Li Y, Zhang Z, Chen R, Wu Y, Peng S, Huang M, Xie W, Cai Q, Hou F, Liu Y, Chen W, Xiao L, Shen Y.	2020
Evidence of recombination in coronaviruses implicating pangolin origins of nCoV-2019	10.1101/2020.02.07.939207	Wong MC, Javornik Cregeen SJ, Ajami NJ, Petrosino JF.	2020
Spike mutation pipeline reveals the emergence of a more transmissible form of SARS-CoV-2	10.1101/2020.04.29.069054	Korber B, Fischer W, Gnanakaran S, Yoon H, Theiler J, Abfalterer W, Foley B, Giorgi E, Bhattacharya T, Parker M, Partridge D, Evans C, Freeman T, de Silva T, LaBranche C, Montefiori D, on behalf of the Sheffield COVID-19 Genomics Group.	2020
Global profiling of SARS-CoV-2 specific IgG/ IgM responses of convalescents using a proteome microarray	10.1101/2020.03.20.20039495	Jiang H, Li Y, Zhang H, Wang W, Men D, Yang X, Qi H, Zhou J, Tao S.	2020
Novel coronavirus 2019-nCoV: early estimation of epidemiological parameters and epidemic predictions	10.1101/2020.01.23.20018549	Read JM, Bridgen JR, Cummings DA, Ho A, Jewell CP.	2020
Aerodynamic Characteristics and RNA Concentration of SARS-CoV-2 Aerosol in Wuhan Hospitals during COVID-19 Outbreak	10.1101/2020.03.08.982637	Liu Y, Ning Z, Chen Y, Guo M, Liu Y, Gali NK, Sun L, Duan Y, Cai J, Westerdahl D, Liu X, Ho K, Kan H, Fu Q, Lan K.	2020
Correlation Analysis Between Disease Severity and Inflammation-related Parameters in Patients with COVID-19 Pneumonia	10.1101/2020.02.25.20025643	Gong J, Dong H, Xia SQ, Huang YZ, Wang D, Zhao Y, Liu W, Tu S, Zhang M, Wang Q, Lu F.	2020
Estimation of COVID-2019 burden and potential for international dissemination of infection from Iran	10.1101/2020.02.24.20027375	Tuite AR, Bogoch I, Sherbo R, Watts A, Fisman DN, Khan K.	2020
Explaining national differences in the mortality of COVID-19: individual patient simulation model to investigate the effects of testing policy and other factors on apparent mortality.	10.1101/2020.04.02.20050633	Michaels JA, Stevenson MD.	2020
Saliva is more sensitive for SARS-CoV-2 detection in COVID-19 patients than nasopharyngeal swabs	10.1101/2020.04.16.20067835	Wyllie AL, Fournier J, Casanovas-Massana A, Campbell M, Tokuyama M, Vijayakumar P, Geng B, Muenker MC, Moore AJ, Vogels CBF, Petrone ME, Ott IM, Lu P, Lu-Culligan A, Klein J, Venkataraman A, Earnest R, Simonov M, Datta R, Handoko R, Naushad N, Sewanan LR, Valdez J, White EB, Lapidus S, Kalinich CC, Jiang X, Kim DJ, Kudo E, Linehan M, Mao T, Moriyama M, Oh JE, Park A, Silva J, Song E, Takahashi T, Taura M, Weizman O, Wong P, Yang Y, Bermejo S, Odio C, Omer SB, Dela Cruz CS, Farhadian S, Martinello RA, Iwasaki A, Grubaugh ND, Ko AI.	2020
Neutralizing antibody responses to SARS-CoV-2 in a COVID-19 recovered patient cohort and their implications	10.1101/2020.03.30.20047365	Wu F, Wang A, Liu M, Wang Q, Chen J, Xia S, Ling Y, Zhang Y, Xun J, Lu L, Jiang S, Lu H, Wen Y, Huang J.	2020
Estimation of SARS-CoV-2 Infection Prevalence in Santa Clara County	10.1101/2020.03.24.20043067	Yadlowsky S, Shah N, Steinhardt J.	2020
Population-level COVID-19 mortality risk for non-elderly individuals overall and for non-elderly individuals without underlying diseases in pandemic epicenters	10.1101/2020.04.05.20054361	Ioannidis JPA, Axfors C, Contopoulos-Ioannidis DG.	2020
Respiratory disease and virus shedding in rhesus macaques inoculated with SARS-CoV-2	10.1101/2020.03.21.001628	Munster VJ, Feldmann F, Williamson BN, van Doremalen N, Pérez-Pérez L, Schulz J, Meade-White K, Okumura A, Callison J, Brumbaugh B, Avanzato VA, Rosenke R, Hanley PW, Saturday G, Scott D, Fischer ER, de Wit E.	2020
Clinical benefit of remdesivir in rhesus macaques infected with SARS-CoV-2	10.1101/2020.04.15.043166	Williamson BN, Feldmann F, Schwarz B, Meade-White K, Porter DP, Schulz J, Doremalen Nv, Leighton I, Yinda CK, Pérez-Pérez L, Okumura A, Lovaglio J, Hanley PW, Saturday G, Bosio CM, Anzick S, Barbian K, Cihlar T, Martens C, Scott DP, Munster VJ, Wit Ed.	2020
Discovery of a novel coronavirus associated with the recent pneumonia outbreak in humans and its potential bat origin	10.1101/2020.01.22.914952	Zhou P, Yang X, Wang X, Hu B, Zhang L, Zhang W, Si H, Zhu Y, Li B, Huang C, Chen H, Chen J, Luo Y, Guo H, Jiang R, Liu M, Chen Y, Shen X, Wang X, Zheng X, Zhao K, Chen Q, Deng F, Liu L, Yan B, Zhan F, Wang Y, Xiao G, Shi Z.	2020

Breaking down of the healthcare system: Mathematical modelling for controlling the novel coronavirus (2019-nCoV) outbreak in Wuhan, China	10.1101/2020.01.27.922443	Ming W, Huang J, Zhang CJP.	2020
Introductions and early spread of SARS-CoV-2 in the New York City area	10.1101/2020.04.08.20056929	Gonzalez-Reiche AS, Hernandez MM, Sullivan M, Ciferri B, Alshammary H, Obla A, Fabre S, Kleiner G, Polanco J, Khan Z, Alburquerque B, van de Guchte A, Dutta J, Francoeur N, Melo BS, Oussenko I, Deikus G, Soto J, Sridhar SH, Wang Y, Twyman K, Kasarskis A, Altman DR, Smith M, Sebra R, Aberg J, Krammer F, Garcia-Sarstre A, Luksza M, Patel G, Paniz-Mondolfi A, Gitman M, Sordillo EM, Simon V, van Bakel H.	2020
Phylodynamics of SARS-CoV-2 transmission in Spain	10.1101/2020.04.20.050039	Díez-Fuertes F, Iglesias-Caballero M, Monzón S, Jiménez P, Varona S, Cuesta I, Zaballos Á, Thomson MM, Jiménez M, García Pérez J, Pozo F, Pérez-Olmeda M, Alcamí J, Casas I.	2020
Using ILI surveillance to estimate state-specific case detection rates and forecast SARS-CoV-2 spread in the United States	10.1101/2020.04.01.20050542	Silverman JD, Hupert N, Washburne AD.	2020
Quantifying SARS-CoV-2 transmission suggests epidemic control with digital contact tracing	10.1101/2020.03.08.20032946	Ferretti L, Wymant C, Kendall M, Zhao L, Nurtay A, Abeler-Dorner L, Parker M, Bonsall DG, Fraser C.	2020
Adoption and impact of non-pharmaceutical interventions for COVID-19	10.12688/wellcomeopenres.15808.1	Imai N, Gaythorpe KA, Abbott S, Bhatia S, van Elsland S, Prem K, Liu Y, Ferguson NM.	2020
Aberrant pathogenic GM-CSF+ T cells and inflammatory CD14+CD16+ monocytes in severe pulmonary syndrome patients of a new coronavirus	10.1101/2020.02.12.945576	Zhou Y, Fu B, Zheng X, Wang D, Zhao C, qi Y, Sun R, Tian Z, Xu X, Wei H.	2020
SARS-CoV-2 invades host cells via a novel route: CD147-spike protein	10.1101/2020.03.14.988345	Wang K, Chen W, Zhou Y, Lian J, Zhang Z, Du P, Gong L, Zhang Y, Cui H, Geng J, Wang B, Sun X, Wang C, Yang X, Lin P, Deng Y, Wei D, Yang X, Zhu Y, Zhang K, Zheng Z, Miao J, Guo T, Shi Y, Zhang J, Fu L, Wang Q, Bian H, Zhu P, Chen Z.	2020
Functional assessment of cell entry and receptor usage for lineage B ß-coronaviruses, including 2019-nCoV	10.1101/2020.01.22.915660	Letko M, Munster V.	2020
Broad anti-coronaviral activity of FDA approved drugs against SARS-CoV-2 in vitro and SARS-CoV in vivo	10.1101/2020.03.25.008482	Weston S, Coleman CM, Haupt R, Logue J, Matthews K, Frieman MB.	2020
Global and Temporal Patterns of Submicroscopic Plasmodium falciparum Malaria Infection	10.1101/554311	Whittaker C, Slater H, Bousema T, Drakeley C, Ghani A, Okell L.	2019

Table 2. Most cited scientific papers in the scientific literature within COVID-19 Wikipedia corpus

Title	Year	Journal	Authors	Citation Count
Understanding the Warburg effect: the metabolic requirements of cell proliferation.	2009	Science	Vander Heiden MG, Cantley LC, Thompson CB.	4927
The MIQE guidelines: minimum information for publication of quantitative real-time PCR experiments.	2009	Clin Chem	Bustin SA, Benes V, Garson JA, Hellemans J, Huggett J, Kubista M, Mueller R, Nolan T, Pfaffl MW, Shipley GL, Vandesompele J, Wittwer CT.	4809
Isolation of a cDNA clone derived from a blood-borne non-A, non-B viral hepatitis genome.	1989	Science	Choo QL, Kuo G, Weiner AJ, Overby LR, Bradley DW, Houghton M.	3672
Isolation of a T-lymphotropic retrovirus from a patient at risk for acquired immune deficiency syndrome (AIDS).	1983	Science	Barré-Sinoussi F, Chermann JC, Rey F, Nugeyre MT, Chamaret S, Gruest J, Dauguet C, Axler-Blin C, Vézinet-Brun F, Rouzioux C, Rozenbaum W, Montagnier L.	3016
The American-European Consensus Conference on ARDS. Definitions, mechanisms, relevant outcomes, and clinical trial coordination.	1994	Am J Respir Crit Care Med	Bernard GR, Artigas A, Brigham KL, Carlet J, Falke K, Hudson L, Lamy M, Legall JR, Morris A, Spragg R.	2904
Toll-like receptors.	2003	Annu Rev Immunol	Takeda K, Kaisho T, Akira S.	2872
The acute respiratory distress syndrome.	2000	N Engl J Med	Ware LB, Matthay MA.	2720
Network biology: understanding the cell's functional organization.	2004	Nat Rev Genet	Barabási AL, Oltvai ZN.	2697
Surviving sepsis campaign: international guidelines for management of severe sepsis and septic shock: 2012.	2013	Crit Care Med	Dellinger RP, Levy MM, Rhodes A, Annane D, Gerlach H, Opal SM, Sevransky JE, Sprung CL, Douglas IS, Jaeschke R, Osborn TM, Nunnally ME, Townsend SR, Reinhart K, Kleinpell RM, Angus DC, Deutschman CS, Machado FR, Rubenfeld GD, Webb SA, Beale RJ, Vincent JL, Moreno R, Surviving Sepsis Campaign Guidelines Committee including the Pediatric Subgroup.	2461
A comprehensive analysis of protein-protein interactions in Saccharomyces cerevisiae.	2000	Nature	Uetz P, Giot L, Cagney G, Mansfield TA, Judson RS, Knight JR, Lockshon D, Narayan V, Srinivasan M, Pochart P, Qureshi-Emili A, Li Y, Godwin B, Conover D, Kalbfleisch T, Vijayadamodar G, Yang M, Johnston M, Fields S, Rothberg JM.	2416

Table 3. Most cited scientific papers in COVID-19 Wikipedia corpus

DOI	Authors	OA	Journal	Year	Source	Title	Wiki	Sci.lit
10.1038/s41586-020-2012-7	Zhou P, Yang XL, Wang XG, Hu B, Zhang L, Zhang W, Si HR, Zhu Y, Li B, Huang CL, Chen HD, Chen J, Luo Y, Guo H, Jiang RD, Liu MQ, Chen Y, Shen XR, Wang X, Zheng XS, Zhao K, Chen QJ, Deng F, Liu LL, Yan B, Zhan FX, Wang YY, Xiao GF, Shi ZL.	Y	Nature	2020	MED	A pneumonia outbreak associated with a new coronavirus of probable bat origin.	8	940
10.3390/v11020174	Wong ACP, Li X, Lau SKP, Woo PCY.	Y	Viruses	2019	MED	Global Epidemiology of Bat Coronaviruses.	6	28
10.1016/j.ijid.2020.01.009	Hui DS, I Azhar E, Madani TA, Ntoumi F, Kock R, Dar O, Ippolito G, Mchugh TD, Memish ZA, Drosten C, Zumla A, Petersen E.	Y	Int J Infect Dis	2020	MED	The continuing 2019-nCoV epidemic threat of novel coronaviruses to global health - The latest 2019 novel coronavirus outbreak in Wuhan, China.	5	228
10.1016/j.jmii.2020.03.013	Lau H, Khosrawipour V, Kocbach P, Mikolajczyk A, Ichii H, Schubert J, Bania J, Khosrawipour T.	Y	J Microbiol Immunol Infect	2020	MED	Internationally lost COVID-19 cases.	5	5
10.1038/d41586-020-00548-w	Cyranoski D.	N	Nature	2020	MED	Mystery deepens over animal source of coronavirus.	5	8
10.1038/s41591-020-0820-9	Andersen KG, Rambaut A, Lipkin WI, Holmes EC, Garry RF.	Y	Nat Med	2020	MED	The proximal origin of SARS-CoV-2.	5	147
10.3390/v2081803	Woo PC, Huang Y, Lau SK, Yuen KY.	Y	Viruses	2010	MED	Coronavirus genomics and bioinformatics analysis.	5	109
10.1007/978-1-4939-2438-7_1	Fehr AR, Perlman S.	Y	Methods Mol Biol	2015	MED	Coronaviruses: an overview of their replication and pathogenesis.	4	195
10.1007/s00134-020-05991-x	Ruan Q, Yang K, Wang W, Jiang L, Song J.	Y	Intensive Care Med	2020	MED	Clinical predictors of mortality due to COVID-19 based on an analysis of data of 150 patients from Wuhan, China.	4	66
10.1038/d41573-020-00016-0	Li G, De Clercq E.	N	Nat Rev Drug Discov	2020	MED	Therapeutic options for the 2019 novel coronavirus (2019-nCoV).	4	105
10.1038/s41422-020-0282-0	Wang M, Cao R, Zhang L, Yang X, Liu J, Xu M, Shi Z, Hu Z, Zhong W, Xiao G.	Y	Cell Res	2020	MED	Remdesivir and chloroquine effectively inhibit the recently emerged novel coronavirus (2019-nCoV) in vitro.	4	474
10.1093/cid/ciaa149	To KK, Tsang OT, Chik-Yan Yip C, Chan KH, Wu TC, Chan JMC, Leung WS, Chik TS, Choi CY, Kandamby DH, Lung DC, Tam AR, Poon RW, Fung AY, Hung IF, Cheng VC, Chan JF, Yuen KY.	Y	Clin Infect Dis	2020	MED	Consistent detection of 2019 novel coronavirus in saliva.	4	94
10.1093/ofid/ofaa105	McCreary EK, Pogue JM.	Y	Open Forum Infect Dis	2020	MED	Coronavirus Disease 2019 Treatment: A Review of Early and Emerging Options.	4	7
10.1126/science.aba9757	Chinazzi M, Davis JT, Ajelli M, Gioannini C, Litvinova M, Merler S, Pastore Y Piontti A, Mu K, Rossi L, Sun K, Viboud C, Xiong X, Yu H, Halloran ME, Longini IM, Vespignani A.	Y	Science	2020	MED	The effect of travel restrictions on the spread of the 2019 novel coronavirus (COVID-19) outbreak.	4	69
10.1093/jtm/taaa030	Rocklöv J, Sjödin H, Wilder-Smith A.	Y	J Travel Med	2020	MED	COVID-19 outbreak on the Diamond Princess cruise ship: estimating the epidemic potential and effectiveness of public health countermeasures.	3	25
10.1101/2020.02.07.939207	Wong MC, Javornik Cregeen SJ, Ajami NJ, Petrosino JF.	N	NA	2020	PPR	Evidence of recombination in coronaviruses implicating pangolin origins of nCoV-2019	3	15
10.1101/2020.02.17.951335	Xiao K, Zhai J, Feng Y, Zhou N, Zhang X, Zou J, Li N, Guo Y, Li X, Shen X, Zhang Z, Shu F, Huang W, Li Y, Zhang Z, Chen R, Wu Y, Peng S, Huang M, Xie W, Cai Q, Hou F, Liu Y, Chen W, Xiao L, Shen Y.	N	NA	2020	PPR	Isolation and Characterization of 2019-nCoV-like Coronavirus from Malayan Pangolins	3	24
10.1111/j.1600-0668.2007.00469.x	Xie X, Li Y, Chwang AT, Ho PL, Seto WH.	N	Indoor Air	2007	MED	How far droplets can move in indoor environments-revisiting the Wells evaporation-falling curve.	3	167
10.1111/tmi.13383	Velavan TP, Meyer CG.	Y	Trop Med Int Health	2020	MED	The COVID-19 epidemic.	3	70
10.1126/science.1118391	Li W, Shi Z, Yu M, Ren W, Smith C, Epstein JH, Wang H, Crameri G, Hu Z, Zhang H, Zhang J, McEachern J, Field H, Daszak P, Eaton BT, Zhang S, Wang LF.	N	Science	2005	MED	Bats are natural reservoirs of SARS-like coronaviruses.	3	967

SI datasets

(1) Table of scientific paper form europmc COVID-19 cited in wikipedia
(2) Table of Wikipedia article-DOI network
(3) Table of protected wikipedia COVID-19 articles

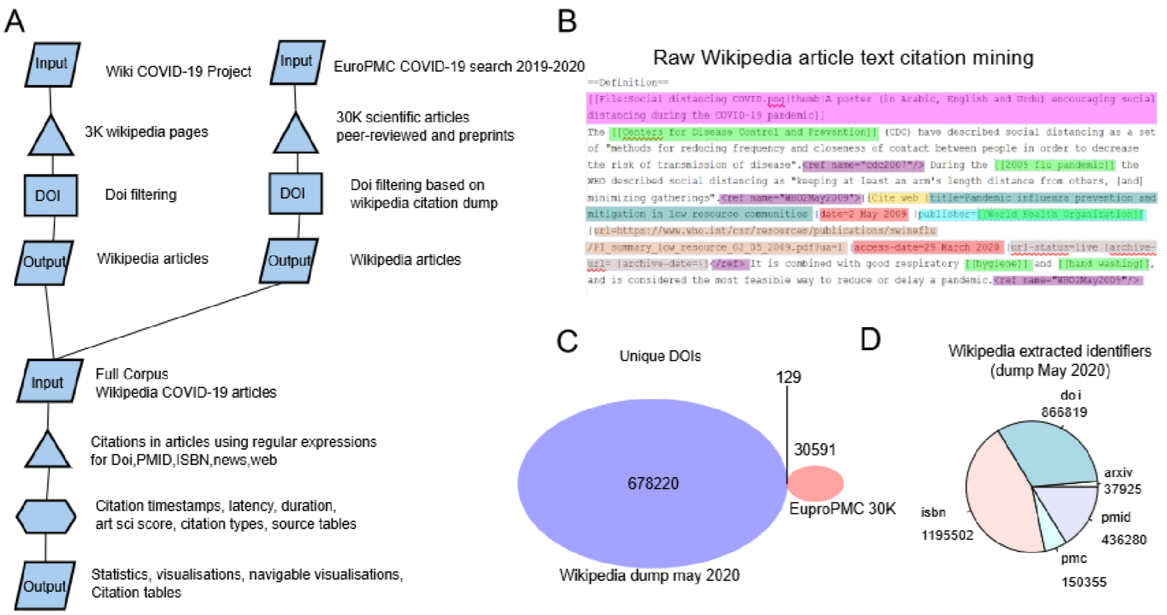

Figure S1. Corpus identification and citation extraction pipeline. A) Scheme of the corpus delimitation rational and citation extraction. To delimit our corpus of Wikipedia articles containing Digital Object Identifier (DOI), we applied two different strategies. First we scraped every Wikipedia pages from the COVID-19 Wikipedia project (about 3K pages) and we filtered them to keep only articles containing DOI citations (149 Wikipedia articles). For our second strategy, we searched the EuroPMC database for COVID-19, SARS-CoV2, SARS-nCoV19 - yielding 30,000 scientific papers, reviews and preprints. These were then compared to the citations extracted from the English Wikipedia dump of May 2020 (860,000 DOIs). Searching Wikipedia with the resulting set led us to identify an additional 91 Wikipedia articles containing at least one citation from the EuroPMC set. Taken together, from the resulting corpus of 231 Wikipedia articles, we extracted DOIs, PMIDs, ISBNs, websites and URLs using a set of regular expressions, as described in the methods. Subsequently, we computed several statistics for each Wikipedia article and we retrieved Atmetics, CrossRef and EuroPMC information for each of their cited papers' DOI. Finally, we produced tables of annotated citations and extracted information from each Wikipadia articles such as books, websites, newspapers. In addition, a timeline of Wikipedia articles and a network of Wikipedia articles linked by their shared scientific sources was produced. B) Example of raw Wikipedia text from the "Social distancing" article, highlighted with several parsed items from a reference. Pink: a hyperlink to an image file, green: Wikipedia hyperlinks, purple: reference, yellow: citation type, dark green: citation title, red: citation date, orange: citation URL. C) Overlap between DOIs from the Wikipedia dump and the 30K EuroPMC COVID-19-related scientific papers and preprints. D) Number of extracted citations with *mwcite* from the English Wikipedia dump of May 2020.

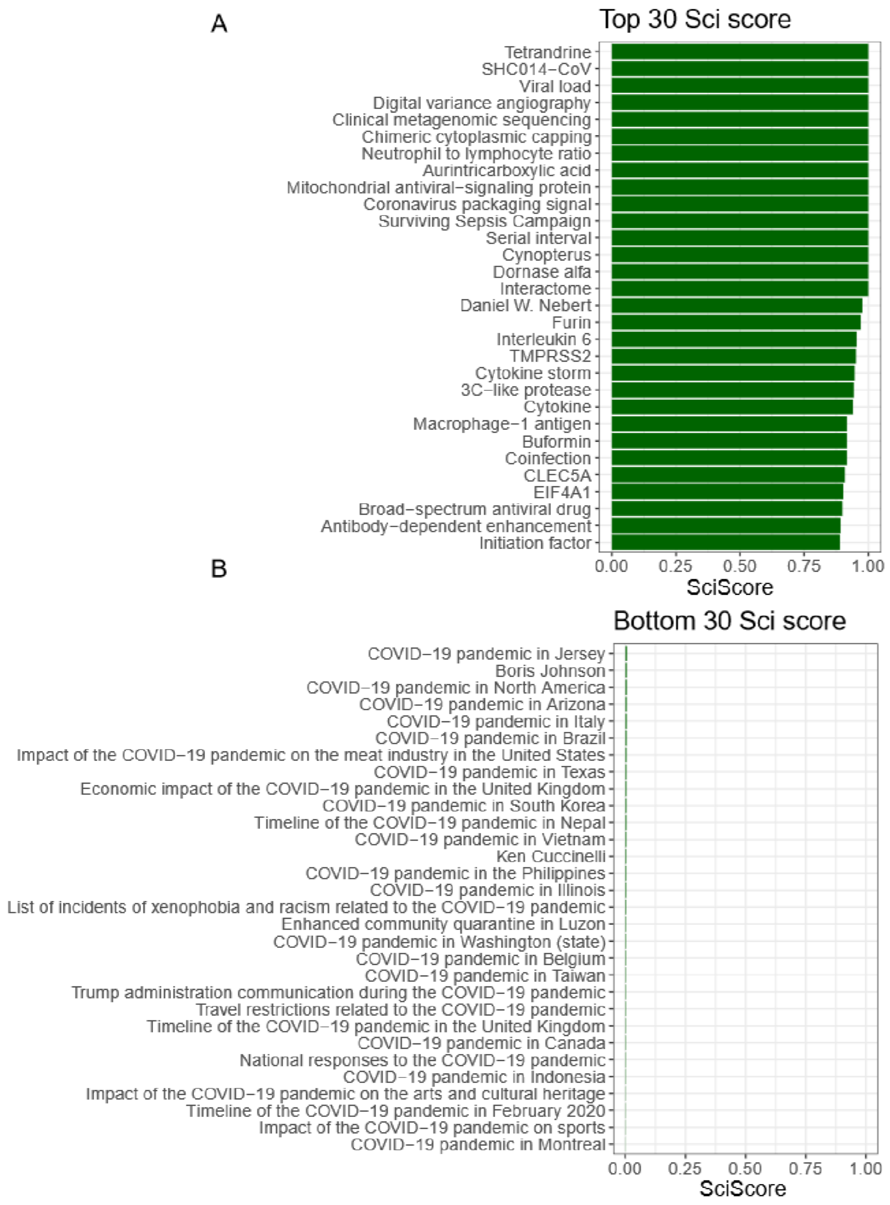

Figure S2. Articles from the Wikipedia COVID-19 corpus with A) the highest and B) lowest scientific scores. The scientific score was computed based on the reference content of each article, as defined in the methods section.

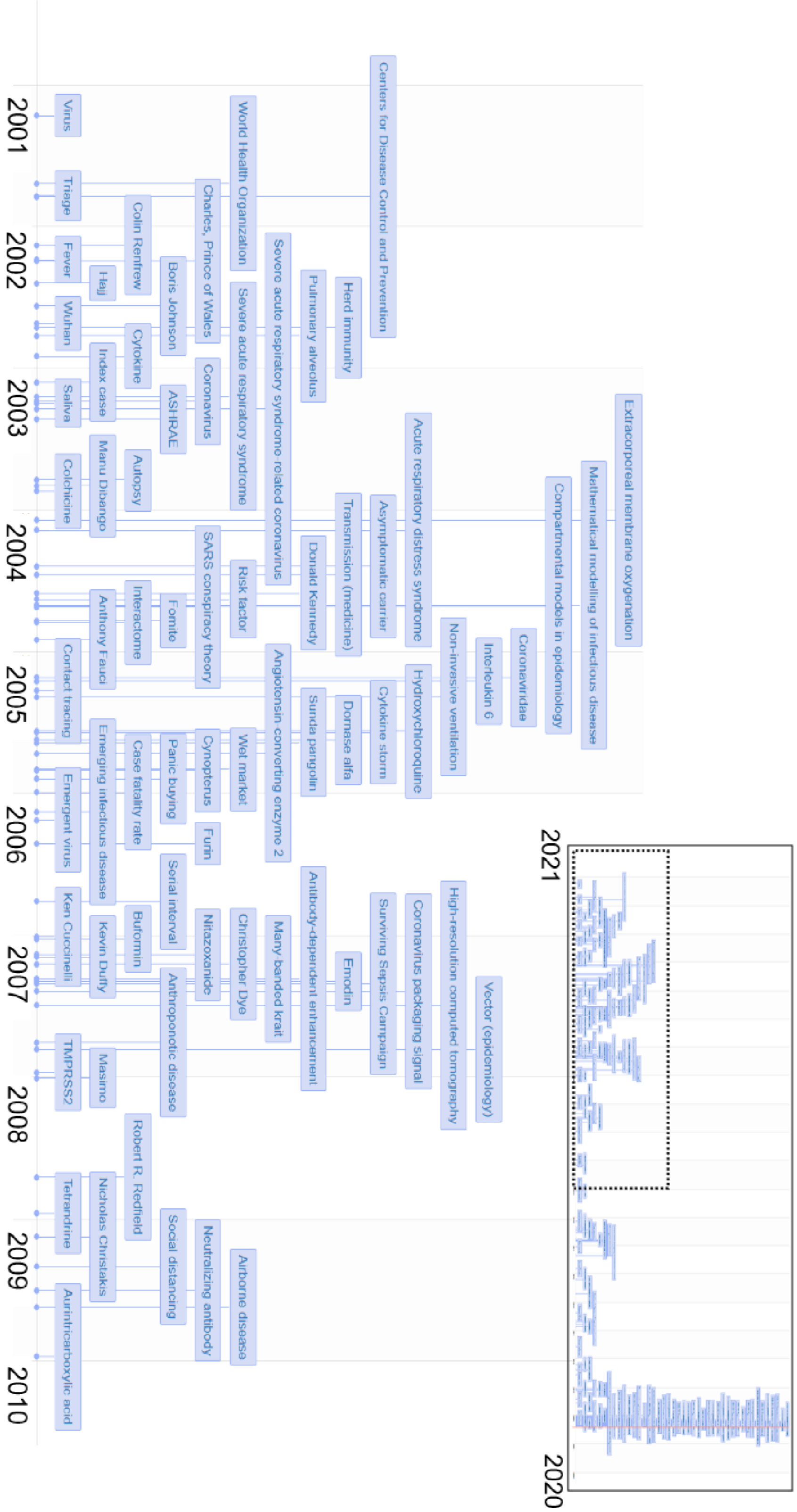

Figure S3. Timeline of the Wikipedia COVID-19 corpus articles, based on date of creation. See here for an interactive version of the timeline.

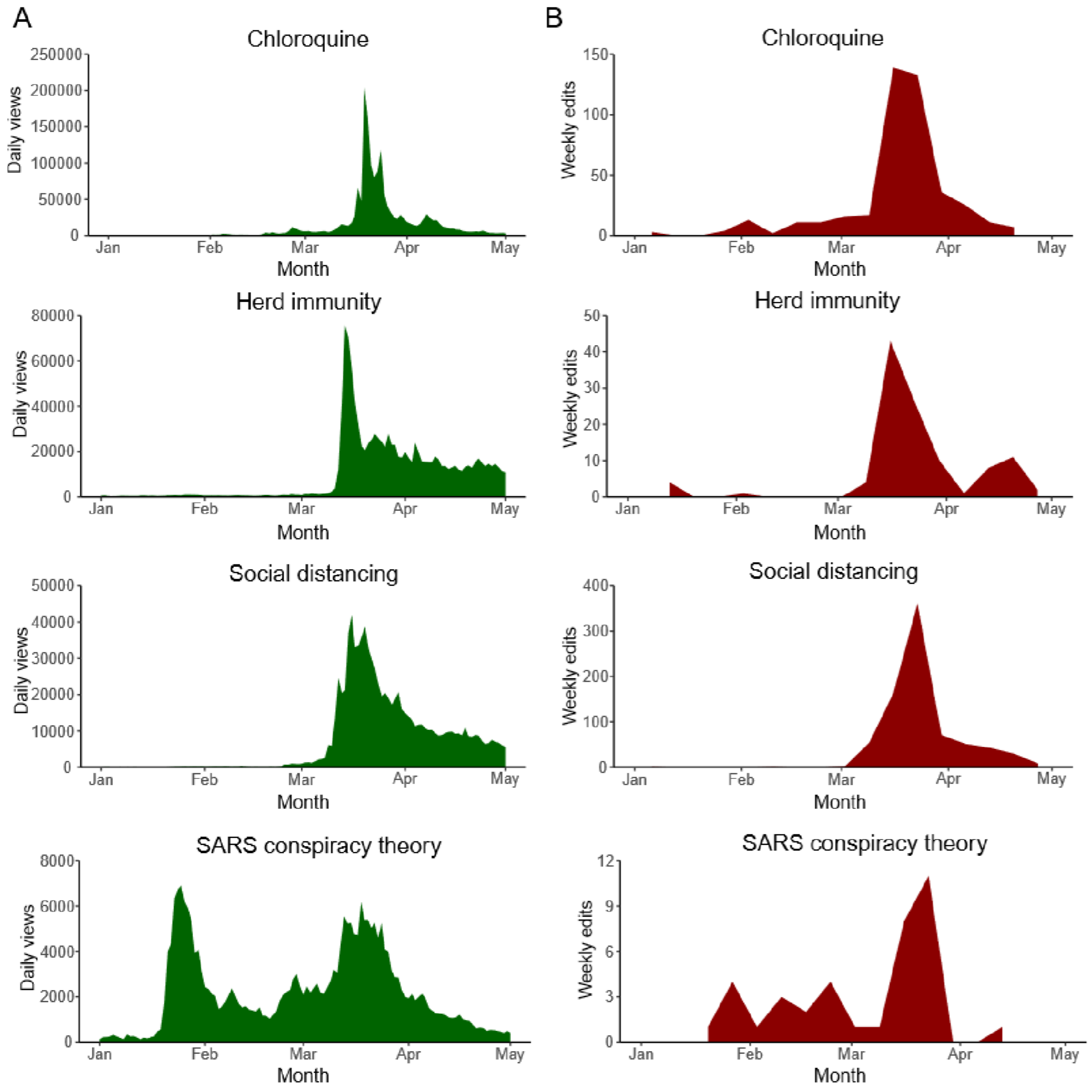

Figure S4. Selected articles' A) page views and B) edit counts during the first wave of COVID-19 pandemic (January–May of 2020).

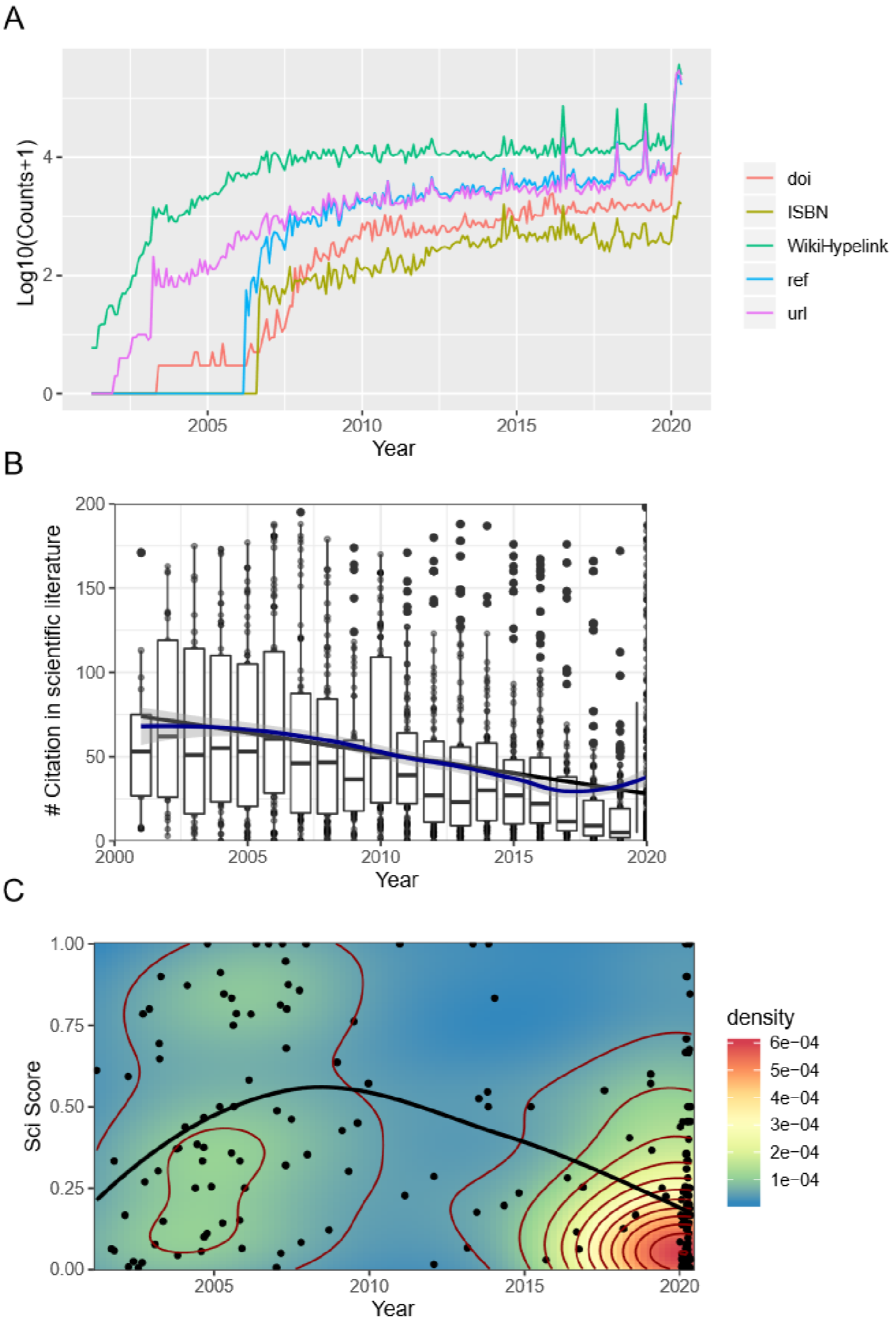

Figure S5. Historical characterization of citations in the COVID-19 corpus. A)Number of references on Wikipedia throughout time, parsed by different type of sources (doi, isbn, hyperlink, url). B) Number of citations in scientific literature as a function of the papers' publication year. C) Scientific score as a function of the creation date of Wikipedia article in the COVID-19 corpus.
