# Supplementary material for: Citation needed? Wikipedia bibliometrics during the first wave of the COVID-19 pandemic
Source: Gigascience. 2022 Jan 12;11:giab095. doi: 10.1093/gigascience/giab095 (PMC8756189; doi:10.1093/gigascience/giab095)
Supplement: giab095_Response_to_Reviewer_Comments_Revision_2 [file giab095_response_to_reviewer_comments_revision_2.pdf]

# Author's Response To Reviewer Comments

Close

Dear editor,
With the present mail, we humbly resubmit for publication our manuscript, entitled: “Citation needed? Wikipedia bibliometrics during the first wave of the COVID pandemic.”

Please see our point by points response:

The Data Availability section needs to be moved after the Discussion and before the Acknowledgements.

As required, we have moved the section at the right place.

This will require the references to be reordered and there are a number that need to be updated (probably manually) too. These are as follows:
The three Zenodo references (references 20-22) are missing their DOI details, as is the GigaDB reference (23) which should be cited like this with the full DOI:
Benjakob O; Aviram R; Sobel JA (2021): Supporting data for "Citation needed? Wikipedia bibliometrics during the first wave of the COVID pandemic" GigaScience Database.

We have manually corrected the above-mentioned references. As some of these were cited in the methods section, their numbering remains the same.

The preprints (e.g. 25-27, 30, 34) are lacking their DOIs making them hard to disambiguate provide accurate metadata so these should be updated too. Some of these have been published in journals (25, 26, 34) so depending on the context on how you are referring to them (in the context of a preprint or from a perspective of their content) you might want to update the citations to the final version.

In this work we would like to keep the citation to the preprints as is due to the point we are making about preprint usage in Wikipedia. We hope that you will agree with us.

Respectfully yours,
Omer Benjakob, Dr. Rona Aviram and Dr. Jonathan Sobel

Close
