# Supplementary material for: Citation needed? Wikipedia bibliometrics during the first wave of the COVID-19 pandemic
Source: Gigascience. 2022 Jan 12;11:giab095. doi: 10.1093/gigascience/giab095 (PMC8756189; doi:10.1093/gigascience/giab095)
Supplement: giab095_Reviewer_1_Report_Original_Submission — Dariusz Jemielniak -- 5/13/2021 Reviewed [file giab095_reviewer_1_report_original_submission.pdf]

Reviewer Report

Title: Citation needed? Wikipedia bibliometrics during the first wave of the COVID-19 pandemic.

Version: Original Submission **Date:** 5/13/2021

Reviewer name: Dariusz Jemielniak

Reviewer Comments to Author:

This is a very solid article on a timely topic. I also commend you for the thorough and meticulous methodology.

One thing that I believe you could amplify on is what would your proposed solution to the "trade off between timeliness and scientificness"? After all, Wikipedia relies on the sources that are reliable, verifiable, but foremostly... available. At the time when there are no academic journal articles published (yet) the chosen modus operandi does not appear to be a trade-off, it is basically the only logical solution. A trade-off would occur if the less valuable sources were not replaced when more academic ones appear, and this is not the case.

I believe you should mention the fact that Wikipedia has an agreement with Cochrane database, which likely affects the popularity of this source.

Additionally, I think that the literature review needs to be expanded. There are already some publications about Wikipedia and COVID-19, as well as about medical coverage on Wikipedia (some non-exhaustive references added below). Moreover, Wikipedia has been a topic covered in GigaScience and it would be reasonable to reflect on the previous conversations in the journal in your publication.

Chrzanowski, J., SoÅ,ek, J., & Jemielniak, D. (2021). Assessing Public Interest Based on Wikipedia's Most Visited Medical Articles During the SARS-CoV-2 Outbreak: Search Trends Analysis. Journal of medical Internet research, 23(4), e26331.

Colavizza, G. (2020). COVID-19 research in Wikipedia. Quantitative Science Studies, 1-32.

Jemielniak, D. (2019). Wikipedia: Why is the common knowledge resource still neglected by academics?. GigaScience, 8(12), giz139.

Jemielniak, D., Masukume, G., & Wilamowski, M. (2019). The most influential medical journals according to Wikipedia: quantitative analysis. Journal of medical Internet Research, 21(1), e11429.

Kagan, D., Moran-Gilad, J., & Fire, M. (2020). Scientometric trends for coronaviruses and other emerging viral infections. GigaScience, 9(8), giaa085.

Methods

Are the methods appropriate to the aims of the study, are they well described, and are necessary controls included? Choose an item.

Conclusions

Are the conclusions adequately supported by the data shown? Choose an item.

Reporting Standards

Does the manuscript adhere to the journal's guidelines on minimum standards of reporting? Choose an item.

Choose an item.

Statistics

Are you able to assess all statistics in the manuscript, including the appropriateness of statistical tests used? Choose an item.

Quality of Written English

Please indicate the quality of language in the manuscript: Choose an item.

Declaration of Competing Interests

Please complete a declaration of competing interests, considering the following questions:

- Have you in the past five years received reimbursements, fees, funding, or salary from an organisation that may in any way gain or lose financially from the publication of this manuscript, either now or in the future?
- Do you hold any stocks or shares in an organisation that may in any way gain or lose financially from the publication of this manuscript, either now or in the future?
- Do you hold or are you currently applying for any patents relating to the content of the manuscript?
- Have you received reimbursements, fees, funding, or salary from an organization that holds or has applied for patents relating to the content of the manuscript?
- Do you have any other financial competing interests?
- Do you have any non-financial competing interests in relation to this paper?

If you can answer no to all of the above, write 'I declare that I have no competing interests' below. If your reply is yes to any, please give details below.

I am a non-paid, volunteer member of the Board of Trustees at Wikimedia Foundation, the non-profit publisher of Wikipedia.

I agree to the open peer review policy of the journal. I understand that my name will be included on my report to the authors and, if the manuscript is accepted for publication, my named report including any attachments I upload will be posted on the website along with the authors' responses. I agree for my report to be made available under an Open Access Creative Commons CC-BY license (http://creativecommons.org/licenses/by/4.0/). I understand that any comments which I do not wish to be included in my named report can be included as confidential comments to the editors, which will not be published.

Choose an item.

To further support our reviewers, we have joined with Publons, where you can gain additional credit to further highlight your hard work (see: https://publons.com/journal/530/gigascience). On publication of this paper, your review will be automatically added to Publons, you can then choose whether or not to claim your Publons credit. I understand this statement.

Yes Choose an item.
