# Supplementary material for: Citation needed? Wikipedia bibliometrics during the first wave of the COVID-19 pandemic
Source: Gigascience. 2022 Jan 12;11:giab095. doi: 10.1093/gigascience/giab095 (PMC8756189; doi:10.1093/gigascience/giab095)
Supplement: giab095_Reviewer_2_Report_Original_Submission — Dean Giustini -- 5/30/2021 Reviewed [file giab095_reviewer_2_report_original_submission.pdf]

Reviewer Report

Title: Citation needed? Wikipedia bibliometrics during the first wave of the COVID-19 pandemic.

Version: Original Submission **Date:** 5/30/2021

Reviewer name: Dean Giustini

Reviewer Comments to Author:

This is a well-written manuscript. The methods are well-described. I've confined my comments to improving the reporting of your methods, some comments about the paper's structure, and a few about the readability of the figures and tables (which I think in general are too small, and difficult to read). Here are my main comments for your consideration as you work to improve your paper:
1) Title of manuscript - the title of your paper seems inadequate to me, and doesn't really convey its content. A more descriptive title that includes the idea of the "first wave" might be useful from my point of view as a reader who scans titles to see if I am interested. I'd recommend including words in the title that refer to your methods. What type of research is this - a quantitative analysis of citations? Title words say a lot about the robust nature of your methods. As you consider whether to keep your title as is, keep mind that title words will aid readers in understanding your research at a glance, and provide impetus to read your abstract (and one hopes the entire manuscript). These words will help researchers find the paper later as well via the Internet's many search engines (i.e., Google Scholar).
2) Abstract - The abstract is well-written. Could the aims of your research be more obvious? and clearly articulated? How about using a statement such as "This research aims to" or similar? I also don't understand the sentence that begins with "Using references as a readout". What is meant by a "readout" in this context? Do you mean to read a print-out of references later? Lower down, you introduce the concept of Wikipedia's references as a "scientific infrastructure", and place it in quotations. Why is it in quotations? I wondered what the concept was on first reading it. A recurring web of papers in Wikipedia constitutes a set of core references - but would I call them a scientific infrastructure? Not sure; they are a mere sliver of the scientific corpus. Not sure I have any suggestions to clarify the use of this phrase.
3) Introduction - This is an excellent introduction to your paper, and it provides a lot of useful context and background. You make a case for positioning Wikipedia as a trusted source of information based on the highly selective literature cited by the entries. However, I would only caution that some COVID-19 entries cite excellent research but the content is contested, and vice versa. One suggestion I had for this section was the possibility of tying citizen science (part of open science) to the rise of Wikipedia's medwiki volunteers. Wikipedia provides all kinds of ways for citizens to get involved in science. As an open science researcher, I appreciated all of the open aspects you mention. Clearly, open access to Wikipedia in all languages is a driving force in combatting misinformation generally, and the COVID "infodemic" specifically. I admit I struggled to understand the point of the section that begins, "Here, we asked what role does scientific literature, as opposed to general media, play in supporting the encyclopedia's coverage of the COVID-19 as the pandemic spread." The opening sentence articulates your a priori research question, always welcome for readers. Would some of the information that

follows in this section around your methods be better placed in the following section under the "Material and Methods"? I found it jarring to read that "....after the pandemic broke out we observed a drop in the overall percentage of academic references in a given coronavirus article, used here as a metric for gauging scientificness in what we term an article's Scientific Score." These two ideas are introduced again later, but I had no idea on reading them here what they signified or whether they were related to research you were building on. You might consider adding a parenthetical statement that they will be described later, and that the idea of a score is your own.
4) Material and methods - Your methods section might benefit from writing a preamble to prepare your readers. As already mentioned, consider taking some of the previous section and recasting it as an introduction to your methods. Consider adding some information to orient readers, and elaborating in a sentence or two about why identifying COVID-19 citations / information sources is an important activity. By the way, what is meant by this: "To delimit the corpus of Wikipedia articles containing DOIs"? Do you mean "identify" Wikipedia articles with DOIs in their references? As I mentioned (apologies in advance for the repetition), it strikes me as odd that you don't refer to this research as a form of citation analysis (isn't that what it is?). Instead you characterize it as "citation counting". If your use of words has been intentional, is there a distinction you are making that I simply do not understand? Also: bibliometricians and/or scientometricians might wonder why you avoid the phrase citation analysis. Further to your methods which are primarily quantitative and statistical - what are the qualitative methods used throughout the paper to analyze the data? How did you carry out this qualitative work? (On page 10, you state "we set out to examine in a temporal, qualitative and quantitative manner, the role of references in articles linked directly to the pandemic as it broke.") That part of your methods seems to be a bit under-developed, and may be worth reconsidering as you work to improve your reporting in the manuscript.
5) Table 1. I am not sure what this table adds to the methods given it leads off your visuals. Do you really need it? It doesn't reveal anything to me and could be in a supplemental file. I also have difficulties in properly seeing table 1; perhaps you could make it larger and more readable?
6) Figure 1. This is the most informative visual in the paper but it is hard to read and crowded. It deserves more space or the information it provides is not fully understood.
7) Figure 3. This is very bulky as a figure, although informative. Again, I'm not sure all of it needs inclusion. Perhaps select part of it, and include other parts in a supplement.
7) Limitations - The paper does not adequately address its limitations. A more fulsome evaluation of limitations would be beneficial to me as a reader, as it would place your work in a larger context. For example, consider asking whether the results are indicative of Wikipedia's other medical or scientific entries? Or are the results not generalizable at all? In other works, are they indicative of something very limited based on the timeframe that you examined? I found myself disagreeing with: "....the mainstream output of scientific work on the virus predated the pandemic's outbreak to a great extent". Is this still true? and what might its significance be now that we are in 2021? Would it be helpful to say that most of the foundational research re: the family of coronaviruses was published pre-2020, but entries about COVID-19 disease and treatment entries are now distinctly different in terms of papers cited, especially going forward. Wiki editors identify relevant papers over time but are not adept at identifying emerging evidence in my experience, or at incorporating important papers early; it's strange given that recency is one of its true calling cards. For me, the most confounding aspect of the infodemic is the constant shifts

of evidence, and how to respond in a way that is prudent and evidence-based. As you point out, Wikipedia has a 8.7 year latency in citing highly relevant papers - and, it seem likely that many important COVID-19 papers were neglected in Wikipedia in the first wave especially about the disease. As you point out, this will form part of future research, which I hope you and your team will pursue.

8) Reference 31 lacks a source: Amit Arjun Verma and S. Iyengar. Tracing the factoids: the anatomy of information reorganization in wikipedia articles. 2021.

Good luck with the next stages in improving your manuscript for publication. I believe it adds to our understanding of Wikipedia's role in promoting sources of information.

Methods

Are the methods appropriate to the aims of the study, are they well described, and are necessary controls included? Choose an item.

Conclusions

Are the conclusions adequately supported by the data shown? Choose an item.

Reporting Standards

Does the manuscript adhere to the journal's guidelines on minimum standards of reporting? Choose an item.

Choose an item.

Statistics

Are you able to assess all statistics in the manuscript, including the appropriateness of statistical tests used? Choose an item.

Quality of Written English

Please indicate the quality of language in the manuscript: Choose an item.

Declaration of Competing Interests

Please complete a declaration of competing interests, considering the following questions:

- Have you in the past five years received reimbursements, fees, funding, or salary from an organisation that may in any way gain or lose financially from the publication of this manuscript, either now or in the future?
- Do you hold any stocks or shares in an organisation that may in any way gain or lose financially from the publication of this manuscript, either now or in the future?
- Do you hold or are you currently applying for any patents relating to the content of the manuscript?

- Have you received reimbursements, fees, funding, or salary from an organization that holds or has applied for patents relating to the content of the manuscript?
- Do you have any other financial competing interests?
- Do you have any non-financial competing interests in relation to this paper?

If you can answer no to all of the above, write 'I declare that I have no competing interests' below. If your reply is yes to any, please give details below.

I declare that I have no competing interests.

I agree to the open peer review policy of the journal. I understand that my name will be included on my report to the authors and, if the manuscript is accepted for publication, my named report including any attachments I upload will be posted on the website along with the authors' responses. I agree for my report to be made available under an Open Access Creative Commons CC-BY license (http://creativecommons.org/licenses/by/4.0/). I understand that any comments which I do not wish to be included in my named report can be included as confidential comments to the editors, which will not be published.

Choose an item.

To further support our reviewers, we have joined with Publons, where you can gain additional credit to further highlight your hard work (see: https://publons.com/journal/530/gigascience). On publication of this paper, your review will be automatically added to Publons, you can then choose whether or not to claim your Publons credit. I understand this statement.

Yes Choose an item.
