# Supplementary material for: Citation needed? Wikipedia bibliometrics during the first wave of the COVID-19 pandemic
Source: Gigascience. 2022 Jan 12;11:giab095. doi: 10.1093/gigascience/giab095 (PMC8756189; doi:10.1093/gigascience/giab095)
Supplement: giab095_Reviewer_3_Report_Original_Submission — Daniel Mietchen -- 6/14/2021 Reviewed [file giab095_reviewer_3_report_original_submission.pdf]

Reviewer Report

Title: Citation needed? Wikipedia bibliometrics during the first wave of the COVID-19 pandemic.

Version: Original Submission **Date:** 6/14/2021

Reviewer name: Daniel Mietchen

Reviewer Comments to Author:

Summary
The present manuscript provides an overview of how the English Wikipedia incorporated COVID-19-related information during the first months of the ongoing COVID-19 pandemic.
It focuses on information supported by academic sources and considers how specific properties of the sources (namely their status with respect to open access and preprints) correlate with their incorporation into Wikipedia, as well as the role of existing content and policies in mediating that incorporation.
No aspect of the manuscript would justify a rejection but there are literally lots of opportunities for improvements, so "Major revision" appears to be the most appropriate recommendation at this point.
General comments
The main points that need to be addressed better:
(1) documentation of the computational workflows;
(2) adaptability of the Wikipedia approach to other contexts;
(3) descriptions of or references to Wikipedia workflows;
(4) linguistic presentation.
Ad 1: while the code used for the analyses and for the visualizations seems to be shared rather comprehensively, it lacks sufficient documentation as to what was done in what order and what manual steps were involved. This makes it hard to replicate the findings presented here or to extend the analysis beyond the time frame considered by the authors.
Ad 2: The authors allude to how pre-existing Wikipedia content and policies - which they nicely frame as Wikipedia's "scientific infrastructure" or "scientific backbone" - "may provide insight into how its unique model may be deployed in other contexts" but that potentially most transferrable part of the manuscript - which would presumably be of interest to many of its readers - is not very well developed, even though that backbone is well described for Wikipedia itself.
Ad 3: there is a good number of cases where the Wikipedia workflows are misrepresented (sometimes ever so slightly), and while many of these do not affect the conclusions, some actually do, and overall comprehension is hampered. I highlighted some of these cases, and others have been pointed out in community discussions, notably at
https://en.wikipedia.org/w/index.php?title=Wikipedia_talk:WikiProject_COVID-19&oldid=1028476999#Review_of_Wikipedia's_coverage_of_COVID and
http://bluerasberry.com/2021/06/review-of-paper-on-wikipedia-and-covid/ . Some resources particularly relevant to these parts of the manuscript have not been mentioned, be it scholarly ones like https://arxiv.org/abs/2006.08899 and https://doi.org/10.1371/journal.pone.0228786 or Wikimedia

ones like https://en.wikipedia.org/wiki/Wikipedia_coverage_of_the_COVID-19_pandemic and https://commons.wikimedia.org/wiki/File:Wikimedia_Policy_Brief_-_COVID-19_-_How_Wikipedia_helps_us_through_uncertain_times.pdf . Likewise essentially missing - although this is a common feature in academic articles about Wikipedia - is a discussion of how valid the observations made for the English Wikipedia are in the context of other language versions (e.g. Hebrew). On that basis, it is understandable that no attempt is made to look beyond Wikipedia to see how coverage of the pandemic was handled in other parts of the Wikimedia ecosystem (e.g. Wikinews, Wikisource, Wikivoyage, Wikimedia Commons and Wikidata), but doing so might actually strengthen the above case for deployability of the Wikipedia approach in other contexts. Disclosure: I am closely involved with WikiProject COVID-19 on Wikidata too, e.g. as per https://doi.org/10.5281/zenodo.4028482 .
Ad 4: The relatively high number of linguistic errors - e.g. typos, grammar, phrasing and also things like internal references or figure legends - needlessly distracts from the value of the paper. The inclusion of figures - both via the text body and via the supplement - into the narrative is also sometimes confusing and would benefit from streamlining.
While GigaScience has technically asked me to review version 3 of the preprint (available via https://www.biorxiv.org/content/10.1101/2021.03.01.433379v3 and also via GigaScience's editorial system), that version was licensed incompatibly with publication in GigaScience, so I pinged the authors on this (via https://twitter.com/EvoMRI/status/1393114202349391872), which resulted (with some small additional changes) in the creation of version 4 (available via https://www.biorxiv.org/content/10.1101/2021.03.01.433379v4) that I concentrated on in my review. Production of that version 4 - of which I eventually used both the PDF and the HTML, which became avaailable to me at different times - took a while, during which I had a first full read of the manuscript in version 3.
In an effort to explore how to make the peer review process more transparent than simply sharing the correspondence, I recorded myself while reading the manuscript for the second time, commenting on it live. These recordings are available via https://doi.org/10.5281/zenodo.4909923 .
In terms of specific comments, I annotated version 4 directly using Hypothes.is, and these annotations are available via https://via.hypothes.is/https://www.biorxiv.org/content/10.1101/2021.03.01.433379v4.full .

Methods

Are the methods appropriate to the aims of the study, are they well described, and are necessary controls included? Choose an item.

Conclusions

Are the conclusions adequately supported by the data shown? Choose an item.

Reporting Standards

Does the manuscript adhere to the journal's guidelines on [minimum standards of reporting?] Choose an item.

Choose an item.

Statistics

Are you able to assess all statistics in the manuscript, including the appropriateness of statistical tests used? Choose an item.

Quality of Written English

Please indicate the quality of language in the manuscript: Choose an item.

Declaration of Competing Interests

Please complete a declaration of competing interests, considering the following questions:

- Have you in the past five years received reimbursements, fees, funding, or salary from an organisation that may in any way gain or lose financially from the publication of this manuscript, either now or in the future?
- Do you hold any stocks or shares in an organisation that may in any way gain or lose financially from the publication of this manuscript, either now or in the future?
- Do you hold or are you currently applying for any patents relating to the content of the manuscript?
- Have you received reimbursements, fees, funding, or salary from an organization that holds or has applied for patents relating to the content of the manuscript?
- Do you have any other financial competing interests?
- Do you have any non-financial competing interests in relation to this paper?

If you can answer no to all of the above, write 'I declare that I have no competing interests' below. If your reply is yes to any, please give details below.

I am an active participant in WikiProject COVID-19, which the present manuscript discusses.

I agree to the open peer review policy of the journal. I understand that my name will be included on my report to the authors and, if the manuscript is accepted for publication, my named report including any attachments I upload will be posted on the website along with the authors' responses. I agree for my report to be made available under an Open Access Creative Commons CC-BY license (http://creativecommons.org/licenses/by/4.0/). I understand that any comments which I do not wish to be included in my named report can be included as confidential comments to the editors, which will not be published.

Choose an item.

To further support our reviewers, we have joined with Publons, where you can gain additional credit to further highlight your hard work (see: https://publons.com/journal/530/gigascience). On publication of this paper, your review will be automatically added to Publons, you can then choose whether or not to claim your Publons credit. I understand this statement.

Yes Choose an item.
