# Supplementary material for: Citation needed? Wikipedia bibliometrics during the first wave of the COVID-19 pandemic
Source: Gigascience. 2022 Jan 12;11:giab095. doi: 10.1093/gigascience/giab095 (PMC8756189; doi:10.1093/gigascience/giab095)
Supplement: giab095_Reviewer_3_Report_Revision_1 — Daniel Mietchen -- 11/17/2021 Reviewed [file giab095_reviewer_3_report_revision_1.pdf]

Reviewer Report

Title: Citation needed? Wikipedia bibliometrics during the first wave of the COVID-19 pandemic.

Version: Revision 1 **Date:** 11/17/2021

Reviewer name: Daniel Mietchen

Reviewer Comments to Author:

I welcome the changes the authors have made - both to the manuscript itself (of which I read the bioRxiv version 5) and to the WikiCitationHistoRy repo - in response to the reviewer comments. I also noticed comments they chose not to address, but as stated before, none of these would be ground for rejection. What I am irritated about is whether the proofreading has actually happened before the current version 5 was posted. For instance, reference 44 seems missing (some others are missing in the bioRxiv version, but I suspect that's not the authors' fault), while lots of linguistic issues in phrases like "to provide a comprehensive bibliometric analyses of english Wikipedia's COVID-19 articles" would still benefit from being addressed.
At this point, I thus recommend that the authors
(a) update the existing Zenodo repository such that there is some more structure in the way the files are shared
(b) archive a release of WikiCitationHistoRy on Zenodo

Methods

Are the methods appropriate to the aims of the study, are they well described, and are necessary controls included? Choose an item.

Conclusions

Are the conclusions adequately supported by the data shown? Choose an item.

Reporting Standards

Does the manuscript adhere to the journal's guidelines on [minimum standards of reporting?](#) Choose an item.

Choose an item.

Statistics

Are you able to assess all statistics in the manuscript, including the appropriateness of statistical tests used? Choose an item.

Quality of Written English

Please indicate the quality of language in the manuscript: Choose an item.

Declaration of Competing Interests

Please complete a declaration of competing interests, considering the following questions:

- Have you in the past five years received reimbursements, fees, funding, or salary from an organisation that may in any way gain or lose financially from the publication of this manuscript, either now or in the future?
- Do you hold any stocks or shares in an organisation that may in any way gain or lose financially from the publication of this manuscript, either now or in the future?
- Do you hold or are you currently applying for any patents relating to the content of the manuscript?
- Have you received reimbursements, fees, funding, or salary from an organization that holds or has applied for patents relating to the content of the manuscript?
- Do you have any other financial competing interests?
- Do you have any non-financial competing interests in relation to this paper?

If you can answer no to all of the above, write 'I declare that I have no competing interests' below. If your reply is yes to any, please give details below.

I contribute to the Wikipedia ecosystem's coverage of COVID-19 and related topics. I received a stipend from Wikimedia Germany to attend Wikimania 2018 and funding from the Alfred P. Sloan Foundation and Wellcome to improve Wikidata-based scholarly profiling software and coverage of clinical trials.

I agree to the open peer review policy of the journal. I understand that my name will be included on my report to the authors and, if the manuscript is accepted for publication, my named report including any attachments I upload will be posted on the website along with the authors' responses. I agree for my report to be made available under an Open Access Creative Commons CC-BY license (http://creativecommons.org/licenses/by/4.0/). I understand that any comments which I do not wish to be included in my named report can be included as confidential comments to the editors, which will not be published.

Choose an item.

To further support our reviewers, we have joined with Publons, where you can gain additional credit to further highlight your hard work (see: https://publons.com/journal/530/gigascience). On publication of this paper, your review will be automatically added to Publons, you can then choose whether or not to claim your Publons credit. I understand this statement.

Yes Choose an item.
